# A protocol for identifying suitable biomarkers to assess fish health: A systematic review

**DOI:** 10.1371/journal.pone.0174762

**Published:** 2017-04-12

**Authors:** Frederieke Kroon, Claire Streten, Simon Harries

**Affiliations:** 1 Australian Institute of Marine Science, Townsville, Queensland, Australia; 2 Australian Institute of Marine Science, Arafura Timor Research Facility, Brinkin, Northern Territory, Australia; Northwest Fisheries Science Center, UNITED STATES

## Abstract

**Background:**

Biomarkers have been used extensively to provide the connection between external levels of contaminant exposure, internal levels of tissue contamination, and early adverse effects in organisms.

**Objectives:**

To present a three-step protocol for identifying suitable biomarkers to assess fish health in coastal and marine ecosystems, using Gladstone Harbour (Australia) as a case study.

**Methods:**

Prior to applying our protocol, clear working definitions for biomarkers were developed to ensure consistency with the global literature on fish health assessment. First, contaminants of concern were identified based on the presence of point and diffuse sources of pollution and available monitoring data for the ecosystem of interest. Second, suitable fish species were identified using fisheries dependent and independent data, and prioritised based on potential pathways of exposure to the contaminants of concern. Finally, a systematic and critical literature review was conducted on the use of biomarkers to assess the health of fish exposed to the contaminants of concern.

**Results/Discussion:**

We present clear working definitions for bioaccumulation markers, biomarkers of exposure, biomarkers of effect and biomarkers of susceptibility. Based on emission and concentration information, seven metals were identified as contaminants of concern for Gladstone Harbour. Twenty out of 232 fish species were abundant enough to be potentially suitable for biomarker studies; five of these were prioritised based on potential pathways of exposure and susceptibility to metals. The literature search on biomarkers yielded 5,035 articles, of which 151met the inclusion criteria. Based on our review, the most suitable biomarkers include bioaccumulation markers, biomarkers of exposure (CYP1A, EROD, SOD, LPOX, HSP, MT, DNA strand breaks, micronuclei, apoptosis), and biomarkers of effect (histopathology, TAG:ST).

**Conclusion:**

Our protocol outlines a clear pathway to identify suitable biomarkers to assess fish health in coastal and marine ecosystems, which can be applied to biomarker studies in aquatic ecosystems around the world.

## Introduction

Globally, the coastal environment is the ultimate sink for many contaminants and their associated breakdown products [1]. Contaminants can enter the coastal environment via aquatic and atmospheric transport from point sources such as shipping ports, industrial waste, sewage outfalls, and stormwater drains, and from diffuse sources such as agricultural and urban runoff [1]. The presence of contaminants has the potential to affect the quality and uses of the coastal environment including direct uses such as fisheries, tourism, and recreation [2].

Assessing the impacts of contaminants on the health of aquatic organisms and ecosystems is challenging due to the presence of multiple stressors and the complexity of ecosystems [3, 4]. Biomarkers have been used extensively to provide the connection between external levels of contaminant exposure, internal levels of tissue contamination, and early adverse effects in organisms [5–7]. As such, they are considered ‘*early warning*’ signals that have the potential to detect an effect in target biota prior to one being observed at the population, community or ecosystem level [3, 5, 7]. Hence, the use of biomarkers can be a critical line of evidence to understand relationships between stressors and effects on coastal resources, and to prevent detrimental impacts of contamination on ecosystem structure and function [3–5].

Fish species are generally considered to be one of the key elements for the assessment of the quality of aquatic ecosystems [5]. First, fish are ubiquitous in almost all aquatic environments with resident fish comprising a critical component of the community exposed to contaminants [5]. Second, fish have high ecological relevance in the aquatic environment due to their influence on food web structure, nutrient cycling and energy transfer. Fish are also an important protein source for humans, and the exposure and effects of contaminants on this food source is of general interest to consumers. Third, the taxonomy, basic life history, and physiology of fish are generally well understood, allowing for targeted studies on internal levels of tissue contamination and early adverse effects. Importantly, however, considerable variation exists among fish species with regards to their contaminant exposure patterns, their basic physiological features, and ultimately their response to environmental contaminants [6]. Hence, fish species used for aquatic health assessment need to be selected based on the potential pathways of exposure to the contaminant of concern, and the biological response that is measured or proposed as a biomarker [6].

**Fig 1:**
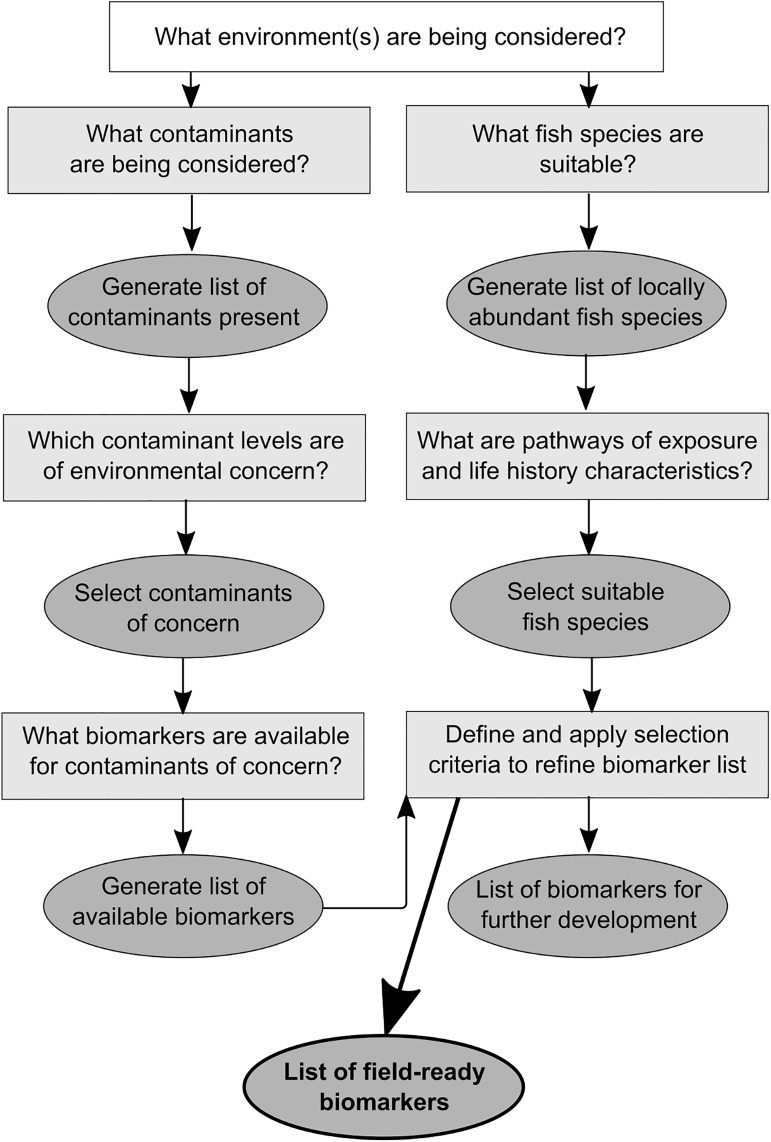
Conceptual diagram outlining a three-step protocol for identifying suitable biomarkers to assess fish health. Rectangles represent literature reviews, ovals represent data lists generated. The protocol is applied using Gladstone Harbour (Australia) as a case study.

In this study, we present a protocol for identifying suitable biomarkers to assess fish health in coastal and marine ecosystems (Fig 1). We outline three steps that are required as part of this protocol, using Gladstone Harbour (Australia) as a case study. First, we identify potential contaminants of concern based on the presence of point and diffuse sources of pollution and available monitoring data for the ecosystem of interest. Second, we identify the most appropriate fish species suitable for biomarker studies based on abundance records from fisheries dependent and independent studies, combined with species-specific biological information that may affect exposure pathways to the contaminants of concern. Next, we conduct a systematic and critical literature review [8] of the biomarkers available to assess the health of fish exposed to the contaminants of concern. Combined, our protocol outlines a clear pathway suitable for biomarker studies that can be used to assess health of aquatic organisms in ecosystems around the world.

## Materials and methods

### Definition of biomarkers

Various definitions of biomarkers are used in the scientific literature resulting in confusion about their meanings [5, 6, 9]. To ensure that our use of the term ‘biomarker’ is consistent with that used in fish health assessments worldwide [5, 6], we reviewed the literature in Web of Science^TM^ for definitions of biomarkers used in assessments of fish health specifically, and aquatic ecosystem health more broadly. Based on our findings, we developed clear working definitions of biomarkers for this study.

### Study area

Gladstone Harbour (Fig. 3.1 in [10]) is located near the City of Gladstone in central Queensland, Australia. The harbour is exposed to various point and diffuse sources of pollution, including shipping, industrial waste, sewage outfalls, stormwater drains, and agricultural and urban runoff. The Harbour is the location of the Port of Gladstone, a major bulk commodity port with 1,648 ships visiting and 100 MT total throughput in the financial year 2014/15 (http://www.gpcl.com.au/). The Port of Gladstone is one of the three major hubs for the export of coal in Queensland [11], and services major industries in the Gladstone region, including mining, engineering, construction, and manufacturing. The City of Gladstone had a population of 66,097 in 2014; the potential impact of stormwater, and urban runoff more broadly, on Gladstone Harbour is currently unknown [10]. The Gladstone area is serviced by seven sewage treatment plants (STP), of which two discharge into watercourses connected to Gladstone Harbour [12]. The two river basins discharging into Gladstone Harbour, the Calliope and the Boyne, contribute additional suspended sediment, nutrients, and pesticides, derived from upstream agricultural land uses [13, 14].

### Contaminants of concern in water and sediment

To identify potential contaminants of concern in water and sediment of Gladstone Harbour, we considered point and diffuse sources of pollution and assessed available monitoring data. First, we examined the National Pollutant Inventory (NPI, [15]) which records annual emissions and transfers of 93 pollutants from facilities around Australia under the National Environment Protection Measures legislation. We extracted facilities, as well as pollution emission and transfer records, for the Gladstone region for 2014/2015. Potential pollutants of concern for Gladstone Harbour were identified based on the NPI inventory and volumes released into water each year. The NPI data do not provide information on the level of exposure, toxicity or the fate of these pollutants in the environment. To evaluate if these NPI pollutants are of environmental concern we reviewed publicly available monitoring and environmental assessment reports from 2005 onwards [10, 16–31]. Where possible, contaminant concentrations in water and sediment were compared against Australian guidelines for water and sediment quality [32–34]. Concentrations of contaminants detected in water and/or sediment in Gladstone Harbour, other than the NPI ones, were also reviewed.

### Selection of suitable fish species

To identify fish species suitable for biomarker studies that could be used to assess fish health, we considered both fisheries dependent [35–39] and independent [40–44] data published from 2005 onwards to generate a list of fish species present for Gladstone Harbour. Commercial fishing data [37] were extracted for two fishing areas in and around Gladstone Harbour, namely S30 and S31 [39]. In addition, we examined catch data from the Queensland Government shark control program [44] from locations nearby Gladstone Harbour (Tannum Sand). Using this list, we identified 20 fish species that are abundant in Gladstone Harbour year-round. To ensure consistency with the latest nomenclature, the names of these 20 fish species were checked against the most recent accepted synonym for scientific name in FishBase (ver 01.2016) (http://www.fishbase.org/), and the accepted common name in Australian Fish Names Standard AS 5300–2015 [45]. Relevant information for identifying the most suitable fish species for future biomarker studies was subsequently collated for each of the 20 fish species from established references [46–48] and internet resources (FishBase ver 01.2016, http://www.fishbase.org/; Australian Museum, http://australianmuseum.net.au/), including (i) potential pathways of exposure to contaminants of concern (e.g. habitat preferences, temporal movement and/or migration patterns, feeding mode, and trophic level in the food web), and (ii) life history characteristics, such as age, size, weight and sex, that may affect such exposure pathways as well as the responses to the contaminants of concern.

### Selection of suitable fish biomarkers

**Fig 2:**
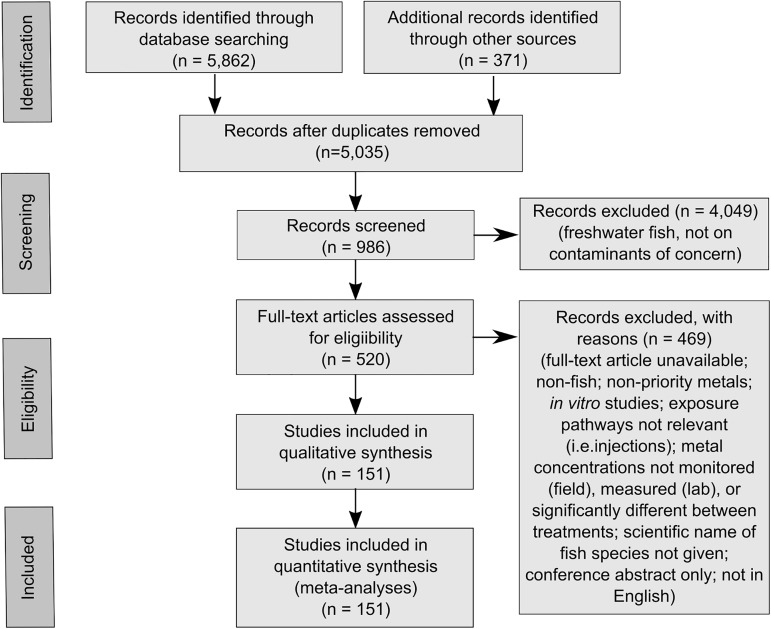
PRISMA flowchart providing the steps of data collection for the systematic review of fish biomarkers to assess fish health. The review focussed on the contaminants of concern identified for Gladstone Harbour (Australia).

To conduct a systematic review and meta-analyses of the global literature of the use of biomarkers in fish health assessment, we followed an established protocol (PRISMA [8], Fig 2). Specifically, we conducted a thorough literature search to develop a database of fish biomarkers in order to assess their potential use in Gladstone Harbour. The search was performed in Web of Science™ in August 2016 and covered the years 1980–2016. The search included the following terms: fish, biomarker*, pollut*, health, condit*, bioaccumul*, metal*, alumin*, cadmium, copper, gallium, lead, selenium, and zinc. Additional records were identified through other sources (e.g. reports in the grey literature, etc.). Following the removal of duplicate records, the remaining publications were screened based on study organisms and contaminants of concern. Records that did not examine the effects of contaminants of concern on coastal and/or marine fish were subsequently removed. Studies on freshwater fish species were excluded given that the speciation and behaviour of metals, and consequently the uptake pathways and sites of bioaccumulation, differ markedly in fresh and marine waters [49–51]. Full text articles were obtained for the remaining records where possible, and assessed for eligibility for inclusion in the qualitative synthesis of the potential use of fish biomarkers for assessing fish health in Gladstone Harbour. Criteria for exclusion included non-fish, non-priority contaminants, *in vitro* studies, pathways of metal application not environmentally relevant (i.e. injections), metal concentrations not monitored (field), measured (lab), or significantly different between treatments, scientific name of fish species not given, full text unavailable, conference abstract only, and text not in English. To identify potential suitable biomarkers for fish health assessment in Gladstone Harbour, the findings of eligible papers were tabulated against individual fish species for the contaminants of concern.

## Results and discussion

### Definition of biomarkers

Based on our review of the international literature [7, 52–59], we agree with previous authors that definitions of biomarkers are rather diffuse (S1 Table) [5, 6, 9]. For example, many definitions of *biomarkers of exposure* include both body burden as well as early response to contaminants. Similarly, definitions for *biomarkers of effects* generally refer to biochemical and physiological alterations resulting from exposure to a contaminant, but sometimes also include changes ranging from molecular, behavioural, and up to ecosystem level. To ensure consistency with the global literature on fish health assessment, we developed the following clear working definitions of biomarkers for this study (see also Figure 3 in [5]):

**Bioaccumulation markers** = Analytical/chemical indicators inside an organism or its products (also referred to as **body burden**) [5],

**Biomarkers of exposure** = Markers which indicate an early biochemical response has occurred following exposure of an individual or organism to a contaminant [3, 59],

**Biomarker of effect** = Measurable biochemical, physiological or other alterations within tissues or body fluids of an organism that can be recognized as associated with an established or possible health impairment or disease [3, 5],

**Biomarker of susceptibility** = Inherent or acquired ability of an organism to respond to the challenge of exposure to a specific xenobiotic substance, including genetic factors and changes in receptors which alter the susceptibility of an organism to that exposure [5].

In our systematic review, we specifically focus on bioaccumulation markers, biomarkers of exposure, and biomarkers of effect.

### Contaminants of concern in water and sediment

Twenty-seven facilities listed their emissions in the NPI database in the Gladstone region in 2014/2015 (S2 Table); the water treatment plant in Agnes Water is the only one not located around Gladstone Harbour and is not further considered. Of the 93 substances that are required to be reported in the NPI, 46 are emitted into the air (44), water (22), and land (14), and 25 are transferred by these 26 facilities (S3 Table). Based on the NPI inventory and volumes released into water each year, potential contaminants of concern for Gladstone Harbour include nutrients (ammonia; total nitrogen, TN; total phosphorus, TP), heavy/trace metals (arsenic, As; cadmium, Cd; chromium, Cr; copper, Cu; lead, Pb; manganese, Mn; nickel, Ni; and zinc, Zn), chlorine, cyanide, and fluoride (Table 1).

**Table 1:** Potential contaminants of concern in Gladstone Harbour based on emission and monitoring reports. Contaminants in bold are those prioritised as contaminants of concern for Gladstone Harbour, and are further considered in identifying suitable fish biomarkers for assessing fish health.

Potential contaminant of concern	Information		References
	Emission	Monitoring	
Ammonia	X	X	[10, 15, 21, 22, 24]
Total nitrogen (TN)	X	X	[10, 15, 21, 22, 24]
Total phosphorus (TP)	X	X	[10, 15, 21, 22, 24]
Chlorophyll a		X	[24]
Turbidity		X	[10, 21, 22, 24]
Chlorine	X		[15]
Cyanide	X	X	[15, 18, 19, 26]
Fluoride	X	X	[15, 18, 19, 26, 27]
**Aluminium (Al)**		X	[10, 17–24, 26, 27]
Arsenic (As)	X	X	[10, 15, 17–24, 26, 27]
**Cadmium (Cd)**	X	X	[10, 15–24, 26, 27]
Chromium (Cr)	X	X	[15, 17–24, 26, 27]
Cobalt (Co)		X	[17, 21, 22, 24, 27]
**Copper (Cu)**	X	X	[10, 15–24, 26, 27]
**Gallium (Ga)**		X	[17, 21, 22, 24]
Iron (Fe)		X	[17–19, 21–24, 26, 27]
**Lead (Pb)**	X	X	[10, 15, 17–19, 21–24, 26, 27]
Manganese (Mn)	X	X	[10, 15–18, 21, 22, 24, 25, 27]
Mercury (Hg)		X	[17–19, 21–24, 26, 27]
Nickel (Ni)	X	X	[10, 15–24, 26, 27]
**Selenium (Se)**	X	X	[15, 17–22, 24, 26, 27]
**Zinc (Zn)**	X	X	[10, 15–20, 22–24, 26, 27]
Tributyltin (TBT)		X	[18, 19, 23, 26, 27, 31]
Polycyclic aromatic hydrocarbon compounds (PAHs)		X	[10, 18, 19, 23, 24, 26, 27]
Polychlorinated biphenyls (PCBs)		X	[18, 23, 27, 31]
Chlorinated hydrocarbons		X	[18, 23, 27, 31]
Semi volatile organic compounds (SVOCs)		X	[18, 23, 27, 31]
Total petroleum hydrocarbons (TPHs)		X	[18, 27, 31]
Benzene, toluene, ethylbenzene and xylene (BTEX)		X	[18, 27, 31]
Organochlorine and organophosphorus pesticides		X	[18, 27, 31]
Herbicides, carbamate pesticides, and insecticides		X	[18, 27, 31]

Monitoring information for Gladstone Harbour water and/or sediment was available for the following contaminants: nutrients; heavy/trace metals; tributyltin (TBT); chlorine, cyanide and fluoride; polycyclic aromatic hydrocarbons (PAHs); polychlorinated biphenyls (PCBs); chlorinated hydrocarbons; semi-volatile organic compounds (SVOC); total petroleum hydrocarbons (TPHs); benzene, toluene, ethylbenzene and xylene (BTEX); organochlorine and organophosphorus pesticides; and herbicides, carbamate pesticides and insecticides (Table 1) [10, 16–31]. In contrast, we were unable to find monitoring information for pharmaceuticals and personal care products. These contaminants were not further assessed to identify contaminants of concern for this review, but if present in Gladstone Harbour may contribute to fish biomarker responses [60, 61].

In Gladstone Harbour, aqueous concentrations of nutrients (e.g. TN, TP) and associated indicators (e.g. turbidity and Chl *a*) have been reported above national guideline values [10, 21, 24, 62]. While potential contaminants of concern for the Gladstone Harbour environment (Table 1), we did not further consider nutrients given that potential impacts likely manifest themselves through changes in aquatic food web structure rather than eco-toxicological pathways. Furthermore, monitoring information on specific N and P constituents rather than TN and TP is required to assess potential eco-toxicological effects (e.g. nitrite [63], nitrate [64]).Based on emission and concentration information in the environment (S3–S5 Tables), the metals aluminium (Al), Cd, Cu, gallium (Ga), Pb, selenium (Se), and Zn were prioritised as contaminants of concern for Gladstone Harbour (Table 1). Concentrations of these metals in water and/or sediment were generally elevated in inner harbour sites compared to reference sites, with those of Al, Zn, and Cu on occasion exceeding their respective national guideline values (S4 and S5 Tables) [16, 17, 19–24, 27–31]. In contrast, elevated concentrations of As, cobalt (Co), Cr, iron (Fe), mercury (Hg), Mn, and Ni are thought to be derived from natural rather than anthropogenic sources [10, 16–27], and are not considered contaminants of concern. In addition, national guideline values for Hg were exceeded in only two out of 1,295 sediment samples over seven years of monitoring [17, 19, 23, 27, 29–31].

Tributyltin (TBT) concentrations in Gladstone Harbour sediment have exhibited a generally decline between 2001 and 2012 to levels well below the national guideline values (S4 and S5 Tables) [19, 27, 31]. Since 2005, TBT has been detected in only five out of 870 sediment samples with concentrations well below the national guideline values [27, 31]. Concentrations of TBT in the water column have not been measured since 2002 [19], because the environmental risk of TBT was expected to decline with the chemical being phased out in the 2000s [18]. Hence, for this study TBT was not considered a contaminant of concern (Table 1).Fluoride, chlorine and cyanide are emitted by industry around Gladstone Harbour (S3 Table), but monitoring information is only available for fluoride and cyanide (S4 and S5 Tables). These inorganic elements are not considered a contaminant of concern for this study (Table 1), as aqueous concentrations of cyanide were all below reporting limits, while those of fluoride were considered to have a low likelihood of adverse ecological effects [19, 27].

Sediment PAHs concentrations in Gladstone Harbour were considerably lower than their low national guideline values, and showed no exceedances of these values in four studies from 2005 to 2012 (S6 Table) [19, 23, 27, 31]. The most recent study detected 11 individual PAHs in 12 to 75% of sediment samples, respectively, with the maximum sediment concentration of total PAHs 59 times lower than the low sediment guideline value [27]. The highest total PAHs concentration reported for Gladstone Harbour sediment was still a magnitude lower than the low national sediment guideline value [31]. Hence, PAHs were not considered a contaminant of concern for this study (Table 1).

Polychlorinated biphenyls (PCBs), chlorinated hydrocarbons and SVOCs were not detected in Gladstone Harbour sediment in surveys conducted in 2009 (S7 Table) [31]. Long chain TPHs were more frequently detected than short chain TPHs, but total TPHs concentrations were almost four times below the low sediment guideline value (S8 Table) [27, 31]. Very few detections of BTEX have been reported (S8 Table) [27, 31]. Combined, the low prevalence and concentrations of these compounds excluded them as a contaminant of concern for fish health assessments (Table 1).

Finally, organochlorine and organophosphorus pesticides, herbicides, carbamate pesticides and insecticides have not been detected in the sediments of Gladstone Harbour (S9–S11 Table) [27, 31]. These two studies analysed more than a 1,000 sediment samples for over 60 different pesticides. Hence, for this study these contaminants were not considered contaminants of concern (Table 1).

### Selection of suitable fish species

A total of 232 fish species were identified for locations in and around Gladstone Harbour from both fisheries dependent [35–39] and independent [40–44] studies, as well as from catch data from the Queensland Government shark control program [44] (S12 Table). Scientific names were not available for some species, potentially resulting in a higher number of species listed than actually present. Based on catch and abundance records, 20 fish species were identified as potentially suitable for biomarker studies (Table 2). This list includes Sand Whiting (*Sillago ciliata*), as earlier surveys report this as the most prevalent fish caught by recreational fishers between 1990 and 2004 [65].

**Table 2:** Exposure and life history characteristics of twenty fish species abundant in and around Gladstone Harbour. Abbreviations: TL = total length, SL = standard length, FL = fork length, ID = insufficient data.

Fish species		Exposure characteristics				Life history characteristics		
Scientific name	Common Name	Habitat	Migration	Movement	Feeding Mode / Trophic level	Size	Weight and Age	Sex
*Acanthopagrus australis*	Yellowfin Bream	Coastal, estuarine	Diadromous, pre-spawning from river to coast	Schooling, demersal	Carnivore (worms, molluscs, crustaceans, echinoderms, ascidians, and small fish)	max 65cm TL	max 3.7–4.5kg; 14 yrs	Protandrous. Sexually mature at 3–4 yrs and 22cm TL
*Acanthopagrus berda*	Pikey Bream	Marine, freshwater, brackish	Oceanodromous	Demersal	Carnivore (worms, molluscs, crustaceans, echinoderms, and small fish)	max 90cm TL; common 35cm TL	max 3.2kg; 14 yrs	Protandrous. Sex change at 19.1 TL and 1.95 yrs
*Ambassis marianus*	Maclaey's Glassfish	Freshwater, brackish	ID	Schooling, demersal	ID	max 10cm SL; common 6cm SL	ID	ID
*Carcharhinus melanopterus*	Blacktip Reef Whaler	Marine, brackish	Amphidromous	Reef associated pelagic	Carnivore (Prefers fishes but also crustaceans, cephalopods and other molluscs)	max 200cm TL	ID (mis-reported as 13.5 kg in FishBase)	Viviparous, placental, mature at 90-120cm TL
*Eleutheronema tetradactylum*	Blue Threadfin	Freshwater, inshore, estuarine, marine	Amphidromous	Loose schools, pelagic-neritic	Carnivore (mainly on ponyfish, other fish, crustaceans, molluscs)	max 200cm TL; common 50cm TL	max 145kg	Protandrous, males at 24–47 cm FL, intersex at 25–46 cm FL and females at 28–72 cm FL
*Epinephelus coioides*	Goldspotted Rockcod	Brackish, marine, rocky sea beds, coral reefs	ID	Solitary, demersal	Carnivore (small fishes, shrimps, cephalopods and crabs)	max 120cm TL	max 15kg; 22 yrs	Protogynous. Sexually mature (25–30 cm)
*Epinephelus quoyanus*	Longfin Rockcod	Marine, reef-associated	ID	Solitary, demersal	Carnivore (shrimp, small fishes, worms and crabs)	max 40cm TL	ID	ID
*Herklotsichthys castelnaui*	Southern Herring	Estuarine, marine	Coastal waters to upper estuaries	Schools, pelagic-neritic	ID	max 20cm SL, common 14cm SL	ID	Oviparous
*Lates calcarifer*	Barramundi	Freshwater, estuarine, coastal	Diadromous, freshwater to estuaries (males)	Demersal	Carnivore (fishes, shrimps, crayfish, crabs and aquatic insects)	max 200cm TL, common 150cm TL	max 60kg; 20 yrs	Protandrous. Sexually mature at 55 cm TL and 3–5 yrs (males), 5 yrs (females)
*Leiognathus equulus*	Common Ponyfish	Freshwater, brackish, marine	Amphidromous	Schooling, demersal	Carnivore (polychaetes, small crustaceans, small fishes and worms)	Max 28cm TL, common 20cm TL	ID	ID
*Lethrinus laticaudis*	Grass Emperor	Marine, brackish, reef-associated	Non-migratory	Schooling, demersal	Carnivore (fish and crustaceans).	max 56cm TL, common 35cm TL	ID	ID
*Liza argentea*	Goldspot Mullet	Freshwater, brackish, marine	Catadromous	Schooling, demersal	Omnivorous filter feeder (detritus, micro-algae, filamentous algae, and benthic organisms)	max 45cm TL, common 18.5cm TL	ID	Oviparous
*Lutjanus argentimaculatus*	Mangrove Jack	Freshwater, estuarine, marine, reef-associated	Oceanodromous	Demersal	Carnivore (fishes, crustaceans)	max 150 cm TL, common 80 cm TL	max 14.5 kg, 39 yrs	ID
*Lutjanus carponotatus*	Stripey Snapper	Coastal, marine, reef-associated	ID	Schooling, demersal	Carnivore (zoobenthos, benthic crustaceans, fish)	max 40cm TL, common 30cm TL	max 20 yrs	Multiple spawner
*Mugil cephalus*	Sea Mullet	Freshwater, estuarine, marine	Coastal spawning migrations	Schooling, benthopelagic	Omnivorous filter feeder (phytoplankton, macroalgae, detritus, and benthic organisms)	max 100cm SL, common 50cm SL	max 12kg, 16 yrs	Sexually mature at 3 to 4 yrs
*Platycephalus fuscus*	Dusky Flathead	Estuarine, marine	ID	Demersal, regular contact with bottom	Active foragers, ambush predators, (small fish, small crustaceans, cephalopods, and polychaete worms)	max 120cm TL	at least 15kg	Gonochoristic or protandrous. Sexual mature at 1.2 yrs and 47cm (males), and at 2–5 yrs and 56.8cm (females)
*Platycephalus indicus*	Bartail Flathead	Brackish, marine, reef associated	Oceanodromous	Demersal, regular contact with bottom	Carnivore (fish, benthic crustaceans)	max 100cm TL, common 60cm TL	max 3.5kg	Mature at 40cm TL
*Pomadasys kaakan*	Barred Javelin	Estuarine, inshore, marine, reef-associated	Spawners form shoals near river mouths during the winter	Demersal	Carnivorous (fish, crustaceans)	max 80.0 cm TL, common 50.0 cm TL	max 6kg	Oviparous, length at maturity 35cm TL
*Scomberomorus queenslandicus*	School Mackerel	Coastal	Oceanodromous seasonal inshore migration	Schooling, pelagic	Carnivore (zooplankton, fish, benthic crustaceans, cephalopods)	max 100cm FL, common 50.0 to 80.0 m FL	max 12.2 kg	ID
*Sillago ciliata*	Sand Whiting	Estuarine, coastal, marine	Non-migratory	Schooling, demersal	Carnivores (benthic polychaetes, crustaceans, and molluscs)	max 51cm TL	max 1.4 kg, 22 yrs	Sexually mature at 24 cm FL (males) and 26 cm FL (females)

The potential pathways of exposure to contaminants of concern are likely to differ for at least some of these 20 fish species, based on habitat use, migration and movement patterns, and feeding modes and trophic levels (Table 2) [46–48] (http://www.fishbase.org/; http://australianmuseum.net.au/). Only one of these 20 species, namely *Scomberomorus queenslandicus*, appears to be restricted to one habitat (coastal), with all other species occurring in two or more habitats. This multi-habitat use is reflected in the migration patterns of the 15 species for which this information is known, with amphidromous, catadromous, diadromous, and oceanodromous patterns all being represented. Only *Lethrinus laticaudis* and *S*. *ciliata* are considered non-migratory. Movements and feeding modes of all but five species, namely *Carcharhinus melanopterus*, *Eleutheronema tetradactylum*, *Herklotsichthys castelnaui*, *Mugil cephalus*, and *S*. *queenslandicus*, are demersal. All 18 species for which the trophic level is known are carnivores, except for *Liza argentea* and *M*. *cephalus*.

Information on life history characteristics was not as readily available for these 20 fish species (Table 2) [46–48] (http://www.fishbase.org/; http://australianmuseum.net.au/). Maximum sizes for individual species ranged from 20 cm Standard Length (SL) (*Ambassis marianus*) to 200 cm Total length (TL) (*C*. *melanopterus*, *E*. *tetradactylum* and *Lates calcarifer*). Many of the 14 species for which weight and/or age information is available have maximum ages of 10 years or more. Labile patterns of sexual development such as protandry and protogony occur in at least five of the 20 species. This information, combined with our systematic review on fish biomarkers (see next section), was used to identify fish species suitable for biomarker studies that could be used to assess fish health in Gladstone Harbour.

### Selection of suitable fish biomarkers

Our systematic review of the literature identified a total of 5,862 publications, including 832 duplicate records (Fig 2; S1 Text). Following screening of the 5,030 records on study organisms and contaminants of concern, 981 records remained for further assessment of eligibility. Based on availability of full text articles and other eligibility criteria, a total of 151 publications were included in the qualitative synthesis of fish biomarkers. In general, the response of fish biomarkers are assessed using three different types of exposure studies, namely (i) in fish harvested from contaminated compared to reference field sites, (ii) in caged fish exposed to contaminated versus reference field conditions, and (iii) in fish exposed to controlled laboratory conditions (including toxicity testing) of contaminated water, sediment or food items.

#### Bioaccumulation markers

Our literature review identified a total of 118 publications on bioaccumulation markers for the identified contaminants of concern (Al, Cd, Cu, Ga, Pb, Se, and Zn) in coastal and marine fish (S13 Table). This includes bioaccumulation studies for seven out of the 20 fish species suitable for biomarker studies in Gladstone Harbour (Table 2), namely *A*. *australis*, *A*. *berda*, *E*. *coioides*, *L*. *equulus*, *M*. *cephalus*, *P*. *fuscus* and *P*. *indicus* [66–75]. Of these 118 studies, 72 were field studies with wild or caged fish collected from contaminated and reference sites, and 46 were controlled laboratory exposure studies. Bioaccumulation was determined primarily in gill, liver and muscle tissues, with whole fish bodies and other tissues such as brains, intestines, and gonads used less often. The majority of field studies (45 studies) examined bioaccumulation in muscle tissue, mostly to determine potential risks of fish consumption to humans. Surprisingly, a large number of field studies (47 studies) did not provide information on the specific life history stages examined. In contrast, in laboratory studies liver tissue (29 studies) and juveniles (33 studies) were most commonly analysed, to examine detoxification, metabolism and secretion of metals in life history stages considered most vulnerable to contaminant exposure. The exposure pathways most commonly examined were sediment contamination in field studies (59 studies) and aqueous contamination in laboratory studies (33 studies). The most common analytical techniques to measure metal bioaccumulation in fish tissues were inductively coupled plasma mass spectrometry (ICP-MS), inductive coupled plasma atomic emission spectrometry (ICP-AES) (also referred to as inductively coupled plasma optical emission spectrometry, ICP-OES), and atomic absorption spectroscopy (AAS).

Bioaccumulation factors (BAFs) for individual fish species, calculated as the metal concentration in the organism (or tissue) divided by that in the sediment or water, were reported in only six out of the 118 studies [76–81]; none of these were on fish species identified as suitable for biomarker studies in Gladstone Harbour. Bioaccumulation factors for sediment (BAF_sed_) and water (BAF_water_) were mostly determined for Cd, Cu and Zn, with BAF_sed_ values for Cd ranging from 0 in *Cathorops spixii* [76] to 521 in *Liza microlepis* [79], for Cu from 0.13 for *Stromateoides argenteus* [81] to 304 for *Liza saliens* [80], and for Zn from 0.21 in *Johnius belengeri* [78] to 447 in *L*. *saliens* [80]. The values for BAF_water_ for Cd ranged from 1,048 in *Cynoglossus sinicus* to 21,333 in *Trypauchen vagina*, for Cu from 987 for *Argyrosomus argentatus* to 2,242 in *Setipinna taty*, and for Zn from 371 in *Johnius belengeri* to 1,651 in *Leiognathus rivulatus* [78]. The high values for BAF_sed_ and BAF_water_ for Cd, Cu and Zn suggest a high bioaccumulation potential for these metals, in fish muscle and liver tissue in particular.

Of the seven contaminants of concern, bioaccumulation of Cd, Cu, Pb and Zn in fish tissues was most often examined, in both field and laboratory studies. In contrast, bioaccumulation of Al and Se was examined much less frequently, or in the case of Ga not at all. Metal bioaccumulation, however, does not always reflect environmental metal concentrations [66, 67, 83, 84], and can vary with fish species [67, 74, 82, 85–87], tissue [66, 67, 73, 86], and life history stage [70, 82], with exposure pathways [82, 88, 89], with season [90, 91], and can be influenced by metal speciation and bioavailability [73, 85, 92]. Hence, when using biomarkers to assess fish health, metal bioaccumulation should be used in conjunction with other biomarkers to indicate whether biological responses are related to bioaccumulation.

#### Biomarkers of exposure

**Biotransformation enzymes: Phase I.** Biotransformation is the process by which an organism alters a xenobiotic compound to enable its excretion. Phase I reactions are the major pathway for the biotransformation of lipophilic compounds and include oxidative, reductive, and hydrolytic reactions where a polar group is either introduced or unmasked (via the addition of an oxygen atom), rendering the xenobiotic molecule less active and more water-soluble and allowing it to be more readily excreted. Most Phase I reactions are catalysed within the microsomal mono-oxygenase (MO) enzyme system and are dependent on the heme protein cytochrome P450 (cyt P450). These heme proteins are located predominantly in the liver, but also present in other fish organelles and tissues [5]. Cyt P450 isozyme induction is triggered when specific xenobiotic compounds bind to the aromatic hydrocarbon (Ah) receptor protein complex and the heat-shock protein 90 (HSP 90), causing HSP 90 to be released.

*Total cytochrome P450 (cyt P450)*. Exposure to certain xenobiotic compounds, such as PAHs and PCBs, can show a strong and highly specific induction in the cyt P450 isozymes and a significant corresponding elevation in total cyt P450 levels (S14 Table). In *L*. *calcarifer*, exposure to different metal concentrations in sediment did not result in differences in cyt P450 levels [93]. The use of total cyt P450 as a biomarker, however, has been somewhat superseded by techniques that target specific isozymes such as cytochrome P450 1A (CYP1A) which allow more specific discrimination of causal agents. Hence, total cyt P450 was not considered a suitable biomarker for fish health assessments in Gladstone Harbour.

*Cytochrome P450 1A (CYP1A)*. Cytochrome P450 1A is a class of cyt P450 isozymes responsible for the biotransformation of a number of compounds including PAHs, halogenated aromatic hydrocarbons (HAHs), PCBs, and dioxins. CYP1A protein levels respond to specific xenobiotic compounds and are generally more responsive to contaminants than other CYP isozymes. CYP1A protein levels can be determined cheaply via immunological tests such as enzyme-linked immunosorbent assays (ELISA) or via histochemical techniques. Xenobiotic-related elevations in CYP1A proteins are usually preceded by an increase in CYP1A mRNA which may also be used as a biomarker.

Four different methods have been used to assess cytochrome P450 1A (CYP1A) activity as a biomarker of fish health when exposed to metal contamination; all of these studies have used sediment or water as the toxicant pathway (S14 Table). CYP1A activity has been measured in liver, gonads, gills and skin. CYP1A activity in gonads and skin did not change in response to metal contamination [86, 94]. A single study measuring CYP1A activity (using immunoreactivity) in gills found that enzyme activity varied with cell type and not site [95]. Assessment of CYP1A activity in the liver using the same method also revealed no significant differences in CYP1A activity between sites contaminated with metals, even though an upregulation of the cyt P450 system was detected in the same tissue using the EROD bioassay [95]. The authors [95] proposed this difference was due to immunochemical assays being less sensitivity than catalytic assays.

Ten studies measured CYP1A in the liver using catalytic activity (8 studies) and mRNA concentrations (2 studies) (S14 Table). Seven studies identified a significant positive or negative change in CYP1A activity when fish were exposed to contaminated sediment. CYP1A activity in fish studied *in situ* was higher when exposed to sediments with elevated metal concentrations; the differential activity of this enzyme could be used to separate effects of organic contamination from metal contamination [96, 97]. The changes in CYP1A activity, however, did not necessarily correlate with metals levels in the water column or metals accumulated in fish tissue [86], as shown by the mixed response observed in some studies [97, 98]. The absence of a relationship between CYP1A activity and metal concentrations is possibly due to the sediments also being contaminated with organic contaminants which are known to induce the CYP1A enzyme due to its role in detoxification of PAHs [96, 97, 99].

Alternatively, the absence of a correlation between cytochrome c system and total concentrations of all metals in the sediment may be due to this enzyme having metal specific responses. For example, it is suppressed by Hg and Cd [100, 101], while Cu induces the system [95]. This complex relationship between CYP1A activity and metals means the suitability of this enzyme as a biomarker is dependent on the ability to resolve responses to individual metals from the suite of metals present in the ecosystem, as is the case in Gladstone Harbour. In addition, CYP1A activity is influenced by season, tissue, life stage and sex and importantly, salinity if estuarine fish species are the target species. Therefore fish health assessments based on changes in the activity of this enzyme would have to employ a strict criteria for sampling to ensure this variability was accounted for [94, 97].

*Ethoxyresorufin O-deethylase (EROD) and aryl hydrocarbon hydroxylase (AHH)*. Ethoxyresorufin O-deethylase (EROD) activity is a measure of catalytic response in fish, and is a sensitive indicator of the inductive response of the cyt P450 system. EROD activity is indicated by changes in the fluorescence of the metabolic product resorufin. Aryl hydrocarbon hydrolase (AHH) catalytic activity can be used as a measure of the response of the CYP1A isoenzyme, by determining the hydroxylation of benzo[a]pyrene. Both EROD and AHH are sensitive biomarkers when measured in the livers of fish, especially when used in conjunction with other biomarkers such as CYP1A and CYP1A mRNA. The other sub units of the cytochrome 450 system are measured using the following assays pentoxyresorufin-O-depentylase (PROD) measures the catalysis of cytochrome P450 CYP2B, 7-benzyloxyresorufin-O-depentylase (BROD) measures the catalytic activity of the cytochrome P450 subfamily CYP3A and 7-methoxyresorufin-O-methoxyresorufin (MROD) cytochrome P450 CYP1A2.

A single study assessed EROD activity in the gills of fish (*Solea senegalensis*) exposed to sediment contaminated with metals and PAHs showed a significant decrease, correlated to heavy metals in preference to PAHs [95]. Although this is a single study, these results suggest that this combination of tissue and biomarker may be suitable for assessing fish health in relation to metals in systems also containing PAHs.

EROD activity in the liver of fish significantly increased as a result of metals exposure in 13 of the 21 of the studies reviewed, while six reported no response and six a mixed response (S14 Table). EROD activity in the liver is heavily influenced by PAHs, due to it being a detoxification pathway for these chemicals [96, 97, 99, 100]. The majority of studies reporting a positive response in EROD activity, however, were also contaminated with organics. Therefore, these findings do not necessarily confirm EROD activity is indicative of metal contamination. For example, Oliva *et al*. [95] reported a significant correlation between Cu labile organic fraction in water and EROD, but also reported a significant correlation between EROD activity and phenanthrene-type metabolites in liver in the same fish. Other studies identified a mixed response, where EROD activity was not necessarily highest at the site with the highest metal concentrations [97, 101–103]. This highlights the complexity of elucidating which particular contaminants are driving EROD activity in systems affected by multiple contaminants, particularly combinations of metals and PAHs. To address this complexity, Fonseca et al. [101] applied generalised linear models (GLMs) to describe the biomarker responses, and was able to separate Hg from the other metals as influencing EROD activity [101]. Finally, similar to CYP1A, EROD activity is influenced by life history characteristics such as sex [97]. Hence, EROD is a potentially a suitable biomarker for assessing fish health in Gladstone Harbour, including *L*. *calcarifer* [93], but will require careful examination in a combination of field and laboratory studies, and analysis to interpret the responses.

Aryl hydrocarbon hydrolase (AHH) catalytic activity was not used in any of the studies reviewed, and is not further considered. The activity of CYP2B and CYP1A2, but not CYP3A, in liver of *Scophthalmus maximus* increased following exposure to sediment contaminated with Cu, Pb and Zn [99]. In contrast, CYP3A levels increased in liver tissue of fish exposed to sediment contaminated with a suite of metals and PAHs [104]. This discrepancy may be due in part to the different methods of analyses used (BROD vs real time PCR analysis of liver mRNA). The limited number of studies applying MROD, BROD, and PROD to measure biomarker response following metal exposure precludes a recommendation for their use in assessing fish health in Gladstone Harbour.

*Cytochrome b5 (cyt b5)*. Cytochrome b5 (Cyt b5) is ‘*involved in the cyt P450-mediated biotransformations through electron donation by NADH via cytochrome b5 reductase*’ [5]. This biomarker is not further considered as none of the studies reviewed used cyt b5.

*NADPH cytochrome P450 reductase (P450 RED)*.According to Van der Oost et al. [5], ‘*Cyt P450 activity depends on reduction of the heme iron by electron transfer from the flavoprotein P450 RED*, *and in some cases from cyt b5*’. P450 RED supplies electrons from NADPH for use in monooxygenase reaction whereas cyt b5 utilises NADP as the electron source [105]. P450 RED activity was not used in any of the studies reviewed, and is not further considered.

**Biotransformation enzymes: Phase II enzymes and co-factors.** Phase II biotransformation involves the attachment (conjugation) of a polar molecule to the xenobiotic parent molecule. The resultant molecule is more water soluble, of higher molecular weight and is more effectively excreted. Depending on the type of xenobiotic compound, certain molecules may be directly metabolised by the phase II system; others may require biotransformation via phase I enzymes first.

*Reduced and oxidised glutathione (GSH and GSSG)*.Reduced glutathione (GSH) and oxidised glutathione (GSSG) provide the major pathways for conjugation of electrophilic compounds and metabolites. GSH plays a key role in the detoxification of xenobiotic compounds by reacting with compounds, replacing hydrogen, chlorine and nitro groups among others. Thiol status is the ratio between reduced and oxidised glutathione (GSH/GSSG) and has been used as an indicator of oxidative stress.

Five studies investigated GSH and GSSH as biomarkers for assessing impact of metals on fish health (S15 Table). Results from field studies vary with no change in GSH/GSSH, a significant decrease, and no response/a negative response in *M*. *cephalus* depending on the age of the fish [106–108]. In laboratory toxicity tests, GSH/GSSH in gills and in whole fish (including *M*. *cephalus*) increased following Pb exposures [69], while GSH/GSSG levels varied with tissue and life stage following Cd exposure [109, 110]. Specifically, results from the latter two studies indicate GSH/GSSG is biphasic, with increasing activity occurring up until a certain concentration after which levels decline [109, 110]. The return to normal GSH/GSSG levels may be due to compensation by other enzymes such as superoxide dismutase (SOD). This means the activity of this enzyme does not necessarily reflect contamination levels after a prolonged exposure time. Based on the variability in GSH/GSSH response in relation to metal exposure concentration and time, fish age and tissue type, as well as the conflicting field and laboratory results for *M*. *cephalus*, we consider this biomarker currently unsuitable for assessing fish health in Gladstone Harbour.

*Glutathione S-transferase (GST)*. Glutathione S-transferase (GST) is a catalyst required for the conjugation of electrophilic compounds by GSH [111]. GST provides essential functions in intracellular transport as well as defence against oxidative damage and peroxidative DNA products.

Twenty studies have examined the response of GST activity in fish to elevated metals levels in water or sediment (S15 Table). The response of GST activity was variable, with six reporting no response and the remaining 14 reporting positive (9 studies) or negative (5 studies) responses. Additionally, in one study different responses were reported when the same fish population were exposed to the same sediment but under either laboratory or field conditions [112]. This variability is likely due to species-specific responses [83, 101], tissue-specific responses [113], seasonal and annual variations [114] and responses to contaminants other than metals (e.g. organics [97, 98]). For example, GST activity was generally higher in liver tissue in *Cynoglossus arel* from polluted locations, but not so in *Acanthopagrus latus* even though hepatic metal bioaccumulation was confirmed [83]. The confirmed metal contamination of marine sediment suggest that these differences are due to different feeding habits, with *C*. *arel* a bottom dwelling fish and *A*. *latus* a more pelagic species [83]. In addition, tissue-specific responses are potentially related to differential metal accumulation with the liver accumulating 30 times more Cu than the kidney, and twice the concentration of Zn [113]. The site of accumulation is related to exposure route for the metals, with the liver mainly representing metals accumulated through diet while the kidney would generally reflect metals taken up through the gills [113]. Even within the same tissue (e.g. gills), however, GST levels have shown an increase [115], a decrease [84], and no significant response [83] to environmental metal concentrations.

Five studies examined GST response under toxicity testing conditions with Cu only, as well as two with Cd and one with Pb (S15 Table). Similar to field studies the responses to Cu and to Cd, respectively, varied with tissue type and showed a biphasic response meaning it could not be used to confirm exposure to higher metal concentrations [109, 110, 116]. In contrast, Pb exposure resulted in a negative response in GST levels in gills and whole juveniles of two different fish species under all concentrations [69]. The absence of a biphasic response to Pb may be due to the metal concentrations not reaching threshold levels for this protein.

Overall, GST activity does respond to metal contamination but this response can vary significantly with species, tissue, season, and the presence of other contaminants, under both field and toxicity testing conditions. Importantly, the GST response to contaminants can be low (~2 fold), making it difficult to differentiate increased activity due to contaminant exposure from natural variability in the fish species of interest [97]. Hence, with many factors influencing the induction of GST activity, but in particular the relatively low response of this enzyme when exposed to contaminants, this biomarker was considered unsuitable for assessing fish health in Gladstone Harbour.

*UDP-glucuronyl transferases (UDPGTs)*. Uridine 5’-diphosphoglucuronic acid—glucuronyl transferases (UDPGT) are the catalysts responsible for the addition of glucuronic acid to a substrate, which is a key pathway for the detoxification and excretion of xenobiotic and endogenous substances. This biomarker is not further considered as none of the studies reviewed used UDPGT.

*Total glutathione*. Five studies used total glutathione to assess fish health when exposed to metals [100, 106, 112, 113, 115] (S15 Table). The response of total glutathione varied with tissue, with no response in the kidney despite confirmed metal accumulation, a positive response in the gills, and a negative one in the liver [113, 115]. The response in either gill or liver was not related with metal accumulation in these tissues [113, 115]. Like GST, total glutathione levels exhibited different responses when the same species was exposed to the same sediment under caged field conditions and controlled conditions [112]. Two other studies found that total glutathione decreased with increasing metal concentrations in the environment, with the same response observed in fish at different life stages [100, 106]. The variability in total glutathione response with exposure conditions and tissues, combined with the low number of studies to assess, would suggest that at this stage total glutathione is not a suitable biomarker for assessing fish health in Gladstone Harbour.

**Oxidative stress parameters.** Oxidative stress parameters are a suite of measures of the oxygen toxicity being suffered by an organism. Oxygen toxicity is caused by reactive oxygen species (ROS) and oxygen free radicals, and is a result of either increased production of these oxidising species, or a significant decrease in production of antioxidants (such as glutathione). Severe oxygen toxicity can lead to enzyme deactivation, lipid peroxidation, DNA damage and eventually apoptosis or cell necrosis. ROS may be produced endogenously, or as a result of the reduction of xenobiotic compounds such as aromatic diols, quinones and hydroxylamines, nitroaromatics, bipyridyls and some transition metal chelates [5].

Superoxide dismutase (SOD), catalase (CAT), and glutathione peroxidase (GPOX), are groups of antioxidant enzymes that form part of a cellular defence system that inhibit and detoxify oxyradical formation. SODs are metalloenzymes that catalyse the reaction of superoxide anions into hydrogen peroxide (which is also an ROS). Resultant hydrogen peroxide is then detoxified by CAT and GPOX. CATs enable the conversion of hydrogen peroxide to release molecular oxygen and water and are involved in fatty acid metabolism as well as detoxifying xenobiotic ROS. GPOXs facilitate the reduction of a range of peroxides (unlike CAT which only acts on hydrogen peroxide) to alcohols. Where CAT uses a donor hydrogen peroxide molecule in the reduction of another, GPOX requires an additional reductant to achieve this (such a selenium-dependant tetrameric cytosolic enzyme), and commonly utilises reduced glutathione (GSH) as a cofactor. GPOX is crucial in protecting cell membranes from damage via lipid peroxidase (LPOX).

*Superoxide dismutase (SOD)*. Nineteen studies have examined the influence of metals on SOD activity in fish (S16 Table). One study used a real-time PCR assay and found no significant response of SOD in fish gonads in response to metal contamination [94]. All other studies used bioassays to measure SOD activity in liver, gills, muscle, intestine, kidney, red blood cells, blood, spleen and whole fish. SOD activity in gill tissue decreased in all studies, irrespective of exposure to mixed metals in field studies or single metal in laboratory toxicity tests [49, 84, 110, 117]. Following Cd exposure in toxicity tests, responses in different tissues have been consistent across various tissues (e.g. gill, liver, spleen [117]), as well as inconsistent (e.g. negative in gill, positive in liver [110]). The response of SOD activity to Cd exposure has been described as biphasic [109] as well as potentially polyphasic [110]. Controlled Cu exposures have resulted in decreased SOD activity in gills and liver [116], although responses can differ between tissues in one and the same species (e.g. no response in liver, negative response in gills and intestines) [49]. Activity of SOD in muscle tissue did not change significantly following Cu exposure [116].

In field studies, SOD activity in fish tissue is responsive, albeit variably, to metal concentrations in sediment and water (S16 Table). Four studies reported no significant change in SOD activity, six showed a mixed response and four reported significant positive responses. For example, SOD activity in the muscle of fish increased when exposed to sediment contaminated with As, Cd, Cu and Pb [118]. In *M*. *cephalus*, SOD activity was lower in individuals exposed to contaminated water [72]. A range of contaminants, however, can affect SOD activity of different species exposed in the same environment, such as Cd, Cu and Zn in *Dicentrarchus labrax* and PAHs in *S*. *senegalensis* and *Pomatoschistus microps* [102]. Despite the general variable results in both field and laboratory studies, the consistent results for SOD activity in gill tissue suggest that this biomarker may be suitable to assess fish health in Gladstone Harbour.

*Catalase (CAT)*. Twenty eight studies have assessed the response of CAT activity to metal contamination, with seven being toxicity tests and 22 field assessments (S16 Table). Following controlled Cu exposure CAT activity did not change in gill tissues [49, 116]. In contrast, controlled Cd exposure resulted in a decrease in CAT activity in gill, liver and spleen tissue in *Synechogobius hasta* [117], but no response in gill, kidney or liver tissue in *Paralicthys olivaceus* [110]. These differences may be due in part to different Cd concentrations being examined rather than species-specific responses. Notwithstanding, differences in CAT activity were identified between *M*. *cephalus* and *Terapon jarbua* following controlled Pb exposure [69]. Results from these toxicity tests show that CAT activity can be species, tissue, metal and concentration dependent [49, 69, 110, 116, 117].

Field studies demonstrate a variety of responses in CAT activity (S16 Table). Sixteen of these studies used bioassays to assess CAT activity in liver tissue, of which only ten reported either a significant increase or decrease in CAT activity at contaminated sites [100, 106, 107, 112, 113, 115, 118–120]. For example, CAT activity in the liver of *D*. *labrax* was correlated with Cd, Cu and Zn in sediment [102]. In *M*. *cephalus*, hepatic CAT activity showed a significant negative response to contaminated water [72], but was also shown to be influenced by season [114]. In addition to season, the variability in CAT responses documented in field studies is likely due to salinity, location, and species [98, 102, 103, 121, 122]. Duration of exposure also affects CAT activity, with response in the liver of *S*. *maximus* correlated with Cd, Hg, Cr, Mn, Ni, Pb and V on day 7 of exposure to sediment, but with Cd, Mn, Ni, Pb and V by day 21 [103]. The variable response of CAT to specific metals or combinations of metals under both field and laboratory conditions suggests that this biomarker is not suitable to assess fish health in Gladstone Harbour.

*Glutathione peroxidase (GPOX)*. Twelve studies assessed glutathione peroxidase (GPOX) activity in fish in response to increased metals concentrations (S16 Table). Controlled laboratory exposures showed that GPOX activity decreased significantly in the gills, liver and spleen but not the kidneys following exposure to Cd [110, 117]. Of the 13 field studies, five reported no change in hepatic GPOX in different fish exposed to metals, four showed a mixed response, two a positive response, and two showed negative response [72, 83, 96, 102, 106, 112, 113, 115, 123]. In *M*. *cephalus*, hepatic GPOX activity decreased significantly following exposure to contaminated water [72]. Like many biomarkers GPOX exhibits seasonal variation and species-specific responses [83, 113, 115]. In addition, GPOX activity in kidneys appears to respond preferentially to Cu concentrations in the sediment [113], which may reduce its effectiveness as a biomarker in cases where Cu is not a contaminant of concern. Based on its inconsistent responses to metals, in combination with seasonal and species variability, this biomarker is not recommended for assessing fish health in Gladstone Harbour.

*Glutathione reductase (GRED)*. Glutathione reductase (GRED) maintains homeostasis between the reduced and oxidised forms of glutathione, namely GSH and GSSG, particularly under oxidative stress conditions. GRED uses the oxidation of NADPH to NADP^+^ to catalyse the reduction of GSSH to GSH. NADPH levels can be measured spectrometrically and can therefore be used as a proxy for GRED activity. Twelve studies in our review examined the response of GRED activity to different metal concentrations (S16 Table). In the one toxicity study, GRED activity showed no response in muscle and gill tissue, and a mixed, biphasic response in liver tissue following Cu exposure [116]. In most of the fish species studied in the field, GRED activity did not significantly change when exposed to contaminated sediment [83, 106, 108, 113]. Only a few field studies reported a significant correlation between hepatic GRED activity and metal concentrations in the environment, including for *M*. *cephalus* [72, 122]. In species showing a mixed response there was no correlation between the GRED activity and metal concentrations [83]. A few studies found significant responses to accumulation, such as higher GRED activity in gill tissues with activity levels correlated with Zn, Cd and Cu accumulation on gill tissue [115]. In cases where changes in GRED activity were reported, the response varied with tissue type, exposure time, and seasonality [104, 113, 115]. Based on these mixed findings, GRED was considered unsuitable as a biomarker for assessing fish health in Gladstone Harbour.

*Non-enzymatic antioxidants*. Non-enzymatic antioxidants such as vitamins have been used as biomarkers although relevant studies are very scarce [5]. Indeed, none of the studies reviewed used non-enzymatic antioxidants and these biomarkers are not further considered.

*Biochemical indices of oxidative damage*. Lipid peroxidation (LPOX) is the oxidation of polyunsaturated fatty acids as a consequence of oxidative stress, and can be quantified by the measurement of degradation products such as aldehydes, acetone and malondialdehyde. Twenty three studies using different methods assessed changes in LPOX activity in fish in relation to metal concentrations (S16 Table). Five field studies identified no response in LPOX activity of fish when exposed to contaminated sediment [102, 118, 124–126]. Several field studies have shown a significant increase in LPOX activity in the blood of *S*. *senegalensis*, *Lutjanus russellii* and *Pomadasys hasta* when exposed to contaminated sediment [120, 127]. This relationship was not observed, however, when the fish were exposed to the same sediment under laboratory conditions [127]. Other field studies showed species- and tissue-dependent responses of LPOX activity, with differences in gill and liver tissues between the pelagic *A*. *latus* and the demersal *C*. *arel* collected from the same locations [83, 128]. For example, hepatic LPOX activity showed a mixed response to contaminant levels in *A*. *latus* but no response in *C*. *arel* [83]. Seasonal variation in LPOX activity has also been detected in various field studies [115, 122, 124]. The influence of life history stage was observed in *Anguilla anguilla* with glass eels showing no response and yellow eels showing a significant positive response when exposed to the same contaminated sediment [106].

Laboratory toxicity tests showed that LPOX activity in gills, liver or spleen significantly increased when fish were exposed to Pb, Cu or Cd [69, 116, 117]. LPOX activity, however, can vary with tissue, with an increase in gill and liver tissue following exposure to Cd or mixed metals in sediment, but not in kidneys [110, 113]. Similar to field studies, LPOX activity varies with life stage following Cd exposure, with juveniles showing a more sensitive response than various larva stages [109]. The relatively consistent changes in LPOX activity in response to metal exposures in a number of studies, including for *M*. *cephalus* [69, 72, 114], suggest this enzyme is a potential biomarker for assessing fish health in Gladstone. However, its suitability will depend on whether species-specific and seasonal responses can be differentiated from those resulting from metal contamination.

Selenium-dependent glutathione peroxidase (Se-GPx) was assessed in four studies (S16 Table). Two field studies showed either a lower Se-GPx activity in *M*. *cephalus* at sites with contaminated sediment [114], or no response in Se-GPx activity in three estuarine fish species [102]. Exposure of *A*. *anguilla* to contaminated sediment in the laboratory resulted in a significant increase in Se-GPx activity in two different studies [100, 112]. However, Se-GPx activity decreased following exposure of the same fish to the same sediment under field conditions [112]. Due to the limited number of studies assessing the effect of metals on Se-GPx activity, combined with conflicting results between laboratory and field tests, this biomarker is not recommended for assessing fish health in Gladstone Harbour.

Reactive oxygen species (ROS) were only assessed in a single study (S16 Table). Following controlled Cu exposure in the laboratory, ROS activity in the estuarine guppy (*Poecilia vivipara*) increased in gill and liver tissue, but showed no response in muscle tissue [116]. The low number of studies precludes recommendation of this biomarker for Gladstone Harbour.

Total oxyradical scavenging capacity (TOSC) is a standardised assay that provides a method of assessing the capability of samples to neutralise ROS. By introducing different ROS to a sample at a constant rate and assessing the efficiency of antioxidants, its scavenging capacity can be determined. Two studies, both on *A*. *anguilla*, used TOSC to assess fish health when exposed to metals (S16 Table). Exposure to contaminated sediment results in no response under field conditions, but a mixed response under laboratory conditions [112]. In contrast, TOSC activity showed a negative response in the same fish species under field conditions [100]. Hence, this biomarker was not recommended for Gladstone Harbour due to inconsistent and insufficient data.

Antioxidant capacity against peroxyl radicals (ACAP) were only assessed in a single study, on the estuarine guppy (*P*. *vivipara*) (S16 Table). Following controlled Cu exposure, ACAP response varied with tissue with no response in gills, a positive response in liver and a negative response in muscle [116]. Due to lack of data, this biomarker was not recommended for Gladstone Harbour.

Glucose-6-phosphate dehydrogenase (G6PDH) contributes to maintaining the level of the co-enzyme NADPH, thereby maintaining the level of glutathione to protect cells such as erythrocytes from oxidative damage. Only a single study, on *Symphodus melops*, used G6PDH to assess the effect of metals and found no response in females but a negative response in males [108]. Due to a lack of data, and the likely interaction of sex and G6PDH response, this biomarker was not recommended for assessing fish health in Gladstone Harbour.

Additional oxidative stress biomarkers were examined in a few studies only, namely Acyl-CoA oxidase; methemoglobin; nitric oxide synthase; hypoxia inducible factor, lipid hydroperoxide, conjugated diene; carbonyl proteins; 2-Keto-4-methiolbutyric acid, oxidised proteins and DT-diaphorase (S8 Table). Due to the limited data for these biomarkers they were not recommended for Gladstone Harbour.

**Biotransformation products.** This biomarker is not further considered as none of the studies reviewed used biotransformation products.

**Stress proteins, metallothioneins and multixenobiotic resistance.** *Stress proteins or heat shock proteins (HSP)*. Stress proteins protect and regenerate cells in response to stress and harmful conditions. The group consists of heat shock proteins (HSPs), which respond (by increasing synthesis) to heat and other physical and chemical stresses, the glucose-regulated proteins (GRPs) which respond to oxygen or glucose deficiency, and the stressor-specific stress proteins which include metallothioneins (MTs) and heme oxygenase proteins.

The eight studies that assessed the response of HSP70 to metal contamination used a range of techniques (S17 Table). Two studies using real-time PCR assays of samples from gonads, skin or liver did not observe a response when fish were exposed to contaminated sediment [86, 94]. In contrast, two other studies using real-time PCR saw upregulation of HSP70 in liver or gills of fish exposed to contaminated sediments, which was confirmed using immunohistochemistry and *in situ* hybridisation [104, 129]. This indicates the suitability of all these methods to asses HSP70 activity. In five of the eight studies HSP70 activity was upregulated, with four assessing the response of fish to contaminated sediment and one examining the response after fish had been exposed to Cd [104, 129–132]. This suggest that this biomarker is suitable for Gladstone Harbour, but further field studies are required to validate its use given the reported seasonal variation in HSP70 activity [133], in combination with the strong influence of climate on HSP70 [5].

The two studies that assessed the response of HSP90 to metal contamination used real-time PCR (S17 Table) [86, 96]. One of these studies reported a response of HSP90 in the skin of fish exposed to contaminated sediment but not the liver [86], while the other found no difference between clean and contaminated fish [96]. Based on the relatively low number of studies on HSP90 and metal contamination, this particular biomarker is not considered a priority for assessing fish health in Gladstone Harbour. However, the reported findings for HSPs in general are promising and these biomarkers could be examined in further detail to assess their suitability in the Gladstone context.

*Metallothioneins (MT)*. Metallothioneins (MTs) are a family of proteins essential for the regulation, sequestration and detoxification of metals including Cu, Zn, Cd, Hg and have been shown to be induced by Co, Ni, Bi and Ag. MTs act by intercepting and binding metal ions as they are being taken up by the cell and also by the removal of metals by non-thionein ligands. MTs can be measured by quantitative assays of the protein via IEC-AAS, metal substitution assays and polarographic and immunochemical techniques. In fish, MT activity is greatest in tissues that are most closely involved in uptake, storage and excretion (such as the gills, liver and small intestine).

MTs are one of the most studied biomarkers for assessing the effect of metal contamination in fish, with a total of 32 studies reviewed (S17 Table). Thirty-one different MT assays were performed on livers from fish exposed to metals in field samples; most of these studies (83%) reported no change or a mixed response. Only a few studies reported a significant change in hepatic MT levels after exposure to contaminated sediment or water [87, 102, 124, 134], and in some of these the response was not observed in all fish exposed to the same contaminants [87, 102]. In contrast, MT levels exhibited a significant positive response in the gills of three different fish species [128, 129, 135], and in the muscle in two other studies [128, 132]. In laboratory toxicity tests, MT levels increased in *Aphanius fasciatus*, *Atherinops affnis*, *Gobius niger*, *Sparus aurata* and *T*. *jarbua* with increasing Cd concentrations [94, 130, 136–139]. However, upregulation of MTs was not sustained over the entire exposure period, with some MTs levels returning to normal levels by the last day of the toxicity test [130, 136–139]. Reported changes in MT levels following Cu toxicity testing were not directly related to Cu concentrations [116, 140]. Controlled exposures to Zn via food resulted in a significant increase in whole juveniles, carcass and viscera of *T*. *jarbua* and *A*. *latus* but not in their gills [141]. Combined, these results suggest that MT activity in tissues other than liver may be a suitable biomarker to assess fish health in Gladstone Harbour.

The copper transporter gene (Ctr1) mediates the transport of copper into eukaryotes for metabolic processes such as a cofactor for enzymes. The single study identified in our review showed that this biomarker in *S*. *aurata* did not change following aqueous exposure to Cu, and the response varied with tissue following dietary uptake of Cu [140]. Hence, Ctr1 was not considered a suitable biomarker for Gladstone Harbour.

*Multixenobiotic resistance (MXR)*. Multixenobiotic resistance (MXR) prevents the accumulation of both xenobiotic and endogenous compounds inside the cell, by removing them via an energy-dependent transport protein, Transmembrane P-glycoprotein (PGP). This biomarker is not further considered as none of the studies reviewed used MXR.

**Haematological parameters.** Haematological parameters offer the ability to conduct non-lethal or non-destructive tests. Haematological parameters may be less chemical specific than some other biomarkers, but provide other insights into the general health and physiology of the organism. A number of haematological parameters have been used to monitor pollution induced stress in fish, including plasma enzyme activity, erythrocyte counts, haemoglobin content and haematocrit (S18 Table).

*Serum transaminases*. Measurement of enzymes in the serum (extracellular fluid or plasma) is considered a sensitive indicator of cellular damage. Alanine transferases (ALT or GPT) and aspartate transaminase (AST or GOT) are a group of enzymes responsible for the conversion of amino acids and α-ketoacids. One of the two studies identified in our review (S18 Table) showed no change in ALT/AST levels of *Gadus morhua* and a mixed response in *Platichthys flesus* when exposed to the same sediment in the field [97]. The other laboratory study reported a significant increase in ALT/AST levels following dietary exposure to Cd in *Rachycentro canadum* [142]. The lower number of studies, combined with their inconsistent results, means that these biomarker are not recommended for assessing fish health in Gladstone Harbour.

*Other haematological parameters*. The delta-aminolevulinic acid dehydratase (ALAD) gene encodes for the δ-aminolevulinic acid dehydratase enzyme also known as porphobilinogen synthase [143]. The ALAD enzyme catalyses the second step in heme synthesis. ALAD is expressed in all tissues, but the highest levels of expression are found in erythrocytes and the liver. This biomarker is not further considered as none of the studies reviewed used ALAD.

Sodium (Na) and chloride (Cl) are the main ions in the extracellular fluid. Maintaining these ion concentrations is necessary so that membrane potential is maintained across the cell. Membrane potential is critical for generating energy, transmission of nerve impulses and muscle movement. Only one study measured Na and Cl in fish after metal exposure and found no significant change [144] (S18 Table). Hence this biomarker is not further considered for Gladstone Harbour.

Haematocrit is the percentage by volume of red blood cells in the blood, whereas erythrocyte counts refer to the absolute number of red blood cells in given volume of blood. Haemoglobin is an iron based oxygen transport molecule that makes up a large proportion of the red blood cell, it can be easily measured and is expressed as g/dL of blood. All of these parameters have been shown to change in the presence of certain contaminants and can be used as indicators of stress or toxicity (S18 Table). Haematocrit results in two field studies were inconsistent though, with one study reporting no response to metal exposure and the other a significant increase [97, 108]. Following dietary exposure in the laboratory, a significant change was observed when fish were fed Cd but not for Se [142, 145]. Erythrocyte counts did not change following exposure to sediment or water contaminated with metals [97, 127, 142]. In contrast, haemoglobin levels in fish increased significantly following exposure to Cu in water [126]. Due to the limited number of studies using haematocrit, erythrocyte counts and haemoglobin as biomarkers, and the inconsistent results for haematocrit and erythrocyte counts, these biomarkers are not recommended for assessing fish health in Gladstone Harbour.

Alkaline phosphatase (ALP) is an enzyme that catalyses the dephosphorylation (hydrolysis of phosphate group) of a range of molecules such as nucleotides and proteins. Two studies used ALP activity in fish (S18 Table), with a mixed response in ALP levels in a field study [97], and a significant increase in ALP levels of fish fed Cd [142]. Due to the limited data this biomarker was not further considered for assessing fish health in Gladstone Harbour.

Intermediary metabolism parameters in the serum, such as glucose, albumin and total protein, are frequently analysed to examine the nutritional status of fish. Two field studies reported different responses for all three biomarkers to metal exposure [97, 120] (S18 Table). Omar et al. [120] identified a significant decrease in total protein and albumin in fish exposed to contaminants. Beyer et al. [97] identified species specific responses in albumin levels and albumin/protein ratio, and reported no significant change in glucose levels in either *G*. *morhua* or *P*. *flesus* when exposed to water containing metals. Due to the limited data on these biomarkers, combined with apparent species-specific responses, they are not recommended for assessing fish health in Gladstone Harbour.

**Immunological parameters.** Immunological parameters have not been used often as biomarkers in fish exposed to metal contamination, with the most common ones being proliferating cell nuclear antigen (PCNA) [120, 129, 146], calcium binding proteins (CBP), cell respiratory burst (RB) and total complementary activity of serum (TCAS) [126, 129] (S18 Table). Levels of PCNA in fish from contaminated sites varied across studies, with higher levels recorded in *Coris julis* [129] and *D*. *labrax* [146], but lower in *P*. *hasta* [120]. Levels of CBP were lower in fish exposed to contaminated sediment while TCAS and RB levels did not show a response [126, 129]. Fasulo et al. [129] concluded that PCNA and CBP were most likely due to physiological adaptation and not a specific response to contaminants. Levels of RB and TCAS *D*. *labrax* did not respond to Cu exposure (in antifouling nets) [126]. Other biomarkers did not respond either [126] suggesting a low toxicity of the antifouling nets rather than insensitivity of the RB and TCAS. The inconsistent results of these few studies suggest that these biomarkers are not suitable to assess fish health in Gladstone Harbour.

Additional immunological parameters that have been used include % leukocytes and % thrombocytes [97, 127], stability of lysosomes 1 and 2 [100, 147], lysozyme activity [103, 112, 148], macrophage aggregate activity (MAM), macrophage aggregates (MA), proteomics serum (Pro-S), cytokine transforming growth factor (β1 9TCF-b1), melanomacrophage centres (MMC), thymus volume (TV) and cell viability (CV) (S18 Table). The response of lysozyme activity following metal exposure varied across studies, with a decline in juvenile *A*. *anguilla*, an increase in *P*. *flesus*, and a mixed response in *S*. *maximus* [103, 112, 148]. Similarly, the response of % leukocytes and % thrombocytes differed in *G*. *morhua* and *S*. *senegalensis* following exposure to sediment contaminated with metals, including when exposed to the same sediment under different conditions [97, 127]. In contrast, both the stability of lysosomes 1 and 2 and MMM showed consistent responses, with the former declining following metal exposure [100, 147], and the latter increasing following exposure to contaminated sediment in the field and Cd exposure in laboratory toxicity testing [117, 148]. Only one study each measured changes in TV, TCF-b1, Pro-S, MAM, MA and CV [72, 103, 145, 147, 149]. Due to the limited number of studies using these immunological parameters these biomarkers were not recommended for Gladstone Harbour.

**Neurotoxic parameters.** Cholinesterase (ChE) are enzymes relevant to neural functions; those with a high affinity for acetylcholin (AChE), and those with affinity for butyrylcholin (BChE) [5]. Fish muscle contains both, while fish brains only contain the former. Nine studies used ChE as a biomarker in fish exposed to metals under field conditions [93, 98, 100, 104, 106, 123, 128, 146, 147], with variable responses reported (S19 Table). Most studies measuring ChE activity in the liver did not identify a response in fish exposed to contaminated sediment [100, 112, 147], while other studies showed a mixed or negative response [98, 106]. Activity of ChE in *L*. *calcarifer* muscle differed across rivers with different levels of contamination, but was not clearly linked with aqueous or sediment metal concentrations [93]. The response of ChE can vary with tissue following exposure to contaminated sediment, with an increase in *S*. *senegalensis* in gill and muscle tissue, but no response in brain tissue [128]. The response of ChE levels in muscle and gill tissue was also variable between studies [93, 123, 128, 146]. This variability in ChE response to metal contamination suggests that it is not suitable as a biomarker to assess fish health in Gladstone Harbour.

The neurotransmitters choline acetyltransferase (ChAT) and serotonin, as well as the enzymes tyrosine hydroxylase (TH), involved dopamine synthesis and the serotonin receptor (5-HT3) have been used as fish biomarkers [129, 146] (S19 Table). Levels of both ChAT and serotonin declined, and those of TH increased, in *D*. *labrax* exposed to sediment contaminated with metals and hydrocarbons [146]. A decline in serotonin levels was also observed in *C*. *julis* exposed to contaminated sediment [129]. The response of 5-HT3 in these two studies, however, varied with an increase in *C*. *julis* but no response in *D*. *labrax* [129, 146]. Given the limited number of studies these biomarkers were not recommended for Gladstone Harbour.

**Reproductive and endocrine parameters.** Pollution stress has well-known effects on reproductive and endocrine parameters in fish [150], however, only a few studies were identified in our review that specifically examined these biomarkers (S19 Table). Vitellogenin (VTG) is a precursor egg yolk protein found in the blood or haemolymph in females of nearly all oviparous species [151]. VTG levels are highly sensitive to endocrine disrupting chemicals (EDCs), and are affected by exposure to some xenobiotics including PCBs and Cd [5]. Only two studies used VTG as a biomarker in fish exposed to metals. Hepatic *vtg* transcripts assessed on real-time PCR were higher in male *A*. *fasciatus* collected from a site contaminated with heavy metals compared to a more pristine site [94]. In contrast, plasma VTG levels in male *P*. *flesus* varied considerable between sites including reference sites [98]. The species-specific gene sequence for *vtg* has been isolated for one of the priority species identified for Gladstone Harbour, namely *L*. *calcarifer* [152–154]. Hence, examining the suitability of this biomarker for Gladstone Harbour may be appropriate if a relationship with fish health can be demonstrated.

The endocrine receptor, thyroid receptor alpha (Trα) has also been used as a biomarker to assess fish health but the levels varied with exposure time [104]. Given that this biomarker was only used in one study it was not recommended for Gladstone Harbour.

**Genotoxic parameters.** Genotoxic parameters refer to any changes to the genetic material of an organism caused by exposure to a contaminant. A number of genotoxic parameters have been used to monitor pollution induced stress in fish, including DNA adducts, secondary DNA modifications, and irreversible genotoxic events (S20 Table).

*DNA adducts*. A DNA adduct is a form of DNA damage caused by the binding of a chemical to a segment of DNA. The two studies identified in our review showed different responses in DNA adducts in *P*. *flesus* exposed to metals, namely an increase [148] and no change [98] (S20 Table). These inconsistent results in a low number of studies preclude recommendation of this biomarker for fish health assessments in Gladstone Harbour.

*Secondary DNA modifications*. Secondary DNA modifications including changes in DNA strand breaks, DNA unwinding, as well as programmed cell death (i.e. apoptosis). DNA strand breaks can be assessed using the comet assay and has been examined in 14 studies [96, 99, 100, 116, 127, 132, 133, 137, 155, 158–162] (S20 Table). Eight out of the eleven field studies reported a significant increase in DNA damage following metal exposure [99, 127, 132, 155, 159–162], including two of the potential suitable fish species (Table 2) namely *E*. *coioides* [159] and *M*. *cephalus* [160]. DNA damage was similar following exposure to metals or to organic pollutants [96]. Seasonal variation in DNA damage has been reported, with the highest damage observed in the post-reproductive season [133]. No change in DNA damage was observed in *S*. *senegalensis* following Cd exposure in toxicity testing conditions [137]. Given the field results, assessing DNA damage using the comet assay may be a suitable biomarker for assessing fish health in Gladstone Harbour. However, its specificity for metal contamination as well as potential seasonal variation with reproductive status [133] will need to be further examined.

DNA unwinding was assessed in two field studies only, showing no effect of metal contamination in *P*. *flesus* [147], but higher DNA unwinding in *L*. *calcarifer* exposed to contaminated sediment [93] (S20 Table). The low number of studies and inconsistent results precluded recommendation of this biomarker for assessing fish health in Gladstone Harbour.

Apoptosis, also known as programmed cell death, is ‘*a physiological and irreversible process in tissue homeostasis that leads to DNA fragmentation of multiples of 180/200 base pairs*’ [5]. All four studies reviewed reported increased apoptosis in four different fish species exposed to metals in the field and laboratory [96, 129, 130, 136] (S20 Table). As such, apoptosis may be appropriate as a biomarker, but further species-specific work is required to determine its suitability to assess fish health in Gladstone Harbour.

The Fas ligand is a type II transmembrane protein involved in apoptosis, and has been used as a biomarker in three of the studies reviewed [129, 132, 146] (S20 Table). The Fas ligand protein increased in both *C*. *julis* and *D*. *labrax* exposed to contaminated sediments [129, 146]. However, no changes were detected when using bioassay in the same study [146]. Due to only three studies using Fas ligand, it was not further considered as a potential biomarker for fish health assessments in Gladstone Harbour.

*Irreversible genotoxic events*. Micronucleus and nuclear abnormalities in fish erythrocytes were examined in twelve studies [84, 90, 96, 100, 112, 114, 116, 127, 132, 158, 160, 162] (S20 Table). Eleven of these identified abnormalities when exposed to contaminated sediment in the field or to Cu in toxicity testing [90, 96, 100, 112, 114, 116, 127, 132, 158, 160, 162], including in *M*. *cephalus* [114, 160]. The effect of metals in *M*. *cephalus* could be identified throughout the year despite seasonal variation in micronuclei frequency [114]. Hence, micronuclei in fish erythrocytes could be a potential biomarker for Gladstone Harbour, but the specificity of this biomarker to certain contaminants needs to be examined.

**Other biomarkers of exposure.** A range of other biomarkers of exposure have been used to assess the response in fish following metal exposure, including osmoregulatory and respiratory markers, and carbohydrate and aerobic metabolism (S21 Table).

*Osmoregulatory and respiratory markers*. Na+ / K+ ATPase (NKA) is an enzyme in the plasma membrane involved in ion transport and osmoregulation, and has been used in five studies to assess the response to metal exposure [84, 106, 135, 144, 163](S21 Table). Exposure to contaminated sediment resulted in an increase in NKA in *A*. *anguilla* [106] but not in *D*. *labrax* [135]. Activity of NKA showed seasonal variation in fish from Brazilian estuaries likely related to ionic changes in the water rather than changes in metal contamination [84]. In laboratory toxicity testing, NKA activity increased in *Fundulus heteroclitus* exposed to Zn [163] but not in *Squalus acanthias* exposed to Pb [144]. Given the variable response of NKA to metal contamination in these studies, this biomarker is not recommended for use in Gladstone Harbour.

The enzyme lactate dehydrogenase (LDH) catalyses the conversion of pyruvate to lactate in anaerobic glycolysis, and has been used in five studies [49, 106, 117, 123, 128] (S21 Table). Four of these reported no change in LDH activity following metal exposure in either the field or under toxicity testing conditions [49, 117, 123, 128]. The other study found that life history stage affected the response of LDH to metal contamination [106]. Lactate, the end product of anaerobic glycolysis, has also been used to as a biomarker, but no effects on lactate levels were reported [49, 144]. Due to the absence in all but one study of a response in LDH activity and lactate levels these biomarkers were not recommended for Gladstone Harbour.

Single studies were identified using other osmoregulatory and respiratory markers, such as aquaporins (AQPs), Ca^2+^-ATPase, trimethylamine oxide (TMAO), urea, PaO_2_, PaCO_2_, arterial pH, K, Ca, NH_4_, Na and Cl [135, 144, 163] (S21 Table). These were not further considered for Gladstone Harbour.

*Carbohydrate and aerobic metabolism*. Pyruvate kinase (PK) is involved in the generation of energy for the cell due to its role in transferring a phosphate to ADP in the final step of glycolysis. Two laboratory toxicity studies examined the effects of Cd exposure (S22 Table), with PK activity increasing in the intestine, but not in hepatic tissue of *F*. *heteroclitus* [49], whereas hepatic PK activity increased in *S*. *hasta* [117]. These inconsistent results of only two studies suggest that PK activity may not be a suitable biomarker for Gladstone Harbour.

Single studies were identified using other carbohydrate and aerobic metabolism biomarkers, namely succinate dehydrogenase (SDH), malic dehydrogenase (MD), hepatic lipase (HL), lipoprotein lipase (LPL), isocitrate dehydrogenase (IDH), hexokinase (HK), citrate synthase (CS), cytochrome c oxidase (COX), 9-cis retinic acid receptor (RXRα) and nuclear receptor binds xenochemical compounds termed estrogen like molecules (Erα) [49, 104, 117, 123] (S22 Table). These were not further considered for Gladstone Harbour.

#### Biomarkers of effect

**Histopathology.** Histopathology of a range of fish tissues following metal exposure has been assessed in 19 studies [69, 84, 96, 98, 117, 129, 131, 135, 139, 145, 148, 160, 164–170] (S23 Table). Fourteen of these reported an increase in histological alterations when fish were exposed to metals in the field (10 studies) and under toxicity testing conditions (4 studies) [69, 96, 117, 129, 131, 135, 139, 148, 160, 164, 166, 168–170]. This includes three of the 20 species identified as potentially suitable for biomarker studies in Gladstone Harbour, namely *M*. *cephalus*, *L*. *calcarifer* and *E*. *coioides* [69, 160, 164, 166]. While histological changes have been observed following metal exposure, this relationship is not necessarily linear or specific to a particular set of contaminants [169, 170]. Therefore, the suitability of histopathology to assess fish health in Gladstone Harbour will need to be examined for specificity and sensitivity.

**Gross indices.** Gross indices reflect fish condition as determined by morphology, appearance and other gross characteristics [5]. These biomarkers have been used extensively to examine the response in fish exposed to metals both in field and laboratory studies (S23 Table).

*Liver somatic index (LSI)*. The hepatic somatic index (HIS) or liver somatic index (LSI) is used to identify possible liver diseases and is estimated using the equation;

LSI=liver weight/body weight×100

Eleven studies examined changes in HSI/LSI in fish exposed to metals [83, 93, 97, 98, 108, 117, 120, 145, 148, 161, 171], with most of these reporting no change [83, 93, 97, 98, 108, 148, 161]. This includes a study on one of the potential suitable fish species *L*. *calcarifer* [93]. Seasonal and intra-specific variation likely confounds any potential effect of metal contamination on LSI [83, 171]. The lack of change in LSI in most studies suggests that LSI is not a suitable biomarker for Gladstone Harbour.

*Gonadosomatic index (GSI)*. The gonadosomatic index (GSI) is used as an indicator of reproductive condition and is estimated using the equation;

GSI=gonad weight/body weight×100

Six of the studies reviewed used GSI to assess fish health following metal exposure [97, 98, 108, 114, 156, 161]. The reported results ranged from a significant decrease in GSI, including in *M*. *cephalus* [108, 114, 161], to no change in GSI [97, 98, 156]. These inconsistent results precluded the inclusion of GSI in the suite of potential biomarkers for Gladstone Harbour.

*Spleen somatic index (SSI)*. The spleen somatic index (SSI) is an indicator of overall spleen health and is calculated using the equation;

SSI=spleen weight/body weight×100

Two studies used SSI to assess fish health following metal exposure, with a decline reported for *Symphodus melops* and no response for *G*. *morhua* [97, 108]. The low number of studies, and inconsistent results meant that this biomarker was not further considered for Gladstone Harbour.

*Visceral somatic index VSI*. The visceral somatic index is an indicator of the overall health of a fish’s viscera. VSI is calculated using the equation;

VSI=spleen weight/body weight×100

One toxicity test used VSI and reported no change in fish exposed to Cd [117]. Hence, this biomarker was not further considered for Gladstone harbour.

*Condition factor (CF)*. The condition factor (CF) is used to assess the general condition of fish, and is estimated using the following equation;

CF=body weight/length×100

Eleven out of the fifteen studies that used CF to assess fish health following metal exposure reported no change or a mixed response [83, 94, 97, 98, 114, 117, 138, 148, 160, 161, 167–169]. Given this inconsistency in results, this biomarker was not further considered for Gladstone Harbour.

*Fulton’s condition index (K index)*. The Fulton’s condition index (K index) is a measure of fish health based on standard weight, and is estimated using the equation;

$$K=100\left(W/L^{3}\right)$$

Nine studies used the K index to assess fish health following exposure to sediment contaminated with metals [93, 101, 106, 124, 125, 139, 172–175]. Five of the 12 fish species sampled in these studies, including *L*. *calcarifer*, showed no change in their K index [93, 101, 106, 124]. One study reported an increase in the K index following exposure to contaminants [125]. Six fish species showed a decrease in the K index in response to metal contamination, of which three were exposed to sediment from the same location [172, 175]. Changes in the K factor are related to exposure time [175], and also vary with life history stage [106]. The variable response of the K index in these metal exposure studies suggests that its suitability and specificity as a biomarker for fish health in Gladstone would have to be assessed in more detail.

*Growth index*. Specific growth rates (in % day) in length and weight have been used to estimate growth indices for both length and weight, using the following equations;

$$GL=100\left({\mathrm{ln L}}_{2}–L_{1}\right)/t_{2}–t_{1}$$

$$GW=100\left({\mathrm{ln W}}_{2}–W_{1}\right)/t_{2}–t_{1}$$

Only one field study used the growth index showing a significant decrease in juvenile *D*. *labrax*, but not in *S*. *maximus*, caged in a polluted harbour [172]. A controlled laboratory exposure to the same sediment resulted in a mixed response in *S*. *maximus* [175]. These inconsistent results suggest that the growth index is not a suitable biomarker for Gladstone Harbour.

*RNA*: *DNA ratio*. The RNA:DNA ratio is used as an indicator of nutritional condition, but its response to metal exposure is highly variable [93, 98, 101, 102, 125, 172, 174, 175]. Some studies have correlated the response in RNA:DNA ratios to metals under certain scenarios [102, 172]. Two other studies reported a decrease in RN:DNA ratio following exposure to contaminated sediment for two estuarine goby species [101] and in *L*. *calcarifer* [93]. In addition, species-specific responses have been observed for the RNA: DNA ratio [102, 172]. Given this variation in response, this biomarker was not recommended for Gladstone Harbour.

**Other biomarkers of effect.** A range of other biomarkers of effect have been used to assess the response in fish following metal exposure, including metabolism and nutrition related biomarkers, parasites, neuromasts in lateral line tissue, prey capture and food intake, phototactic responses and epidermal diseases (S23 Table).

*Metabolism and nutrition*. The lipid storage index has been used to assess fish health based on ratio of the quantity of triacylglycerols (TAG; reserve lipids) to the quantity of sterols (ST; structural lipids) (TAG:ST) [172, 175]. Following exposure to contaminated sediments, TAG:ST declined in juvenile *S*. *maximus* under controlled laboratory conditions [103], in juvenile *D*. *labrax* and *S*. *maximus* caged in a polluted harbour [175], and in *P*. *flesus* in field conditions [173]. The total lipid concentration were analysed in three studies [102, 117, 173], with the results in field studies ranging from species-specific responses [102] to a decline [173]. In controlled toxicity tests, the total lipid concentration increased in *S*. *hasta* following Cd exposure [117], but declined in *E*. *coioides* following Cu toxicity [164]. Total protein concentration in muscle or liver was used in three studies that reported a mixed response [98, 102, 144], while total muscle glycogen levels only started to decline at high Pb concentrations [144]. Total polyunsaturated fatty acids decreased, and monounsaturated fatty acids and total saturated fatty acid increased in juvenile *E*. *coioides* following Cu exposure [164]. In the same study, the digestive enzymes protease, amylase and lipase in the liver, stomach and intestine decreased [164]. Collagen levels were higher in *A*. *fasciatus* deformed following Cd exposure, compared to normal fish under field conditions, and corresponded with increased collagen fibres [139]. In contrast, collagen levels did not differ in non-deformed fish from reference and contaminated sites [139]. Overall, these results suggest that out of all the metabolism and nutrition biomarkers TAG:ST may be the most suitable to assess fish health in Gladstone Harbour.

*Parasites*. The abundance of parasites has been assessed as a fish biomarker of sediment metal concentrations in the North Sea [147, 167]. The parasite community on *P*. *flesus* was influenced by sediment metal concentrations, with the abundance of several parasites negatively correlated with the concentration of individual metals [147]. Parasite abundance and diversity correlated significantly with several other biomarkers, including EROD, Choline (brain) activity, macrophage aggregate activity (MAM), hepatic lysosomal stability (LY2) and plasma lysosomal activity (Lys) [147]. No changes in the abundance of the one parasite examined was reported in the second study [167]. The apparent metal specificity of parasite abundance in fish [147], and the role of parasites in assessing fish health more broadly, suggests that this may be a suitable biomarker for Gladstone Harbour. However, given that only one study examined responses in parasite community more work needs to be conducted to assess its suitability.

*Lateral line tissue*. The lateral line of fish is composed of neuromasts and is involved in their behaviour such as detecting predators and avoiding obstacles [176]. The one study that examined the response of neuromasts to metal exposure reported localised damage in *D*. *labrax*, resulting in a 10% decrease in fish escape rate [176]. Damage to lateral line tissue is thus a potential indicator of detrimental impacts at higher biological levels. However, only one study examined this biomarker and it is not recommended for use in Gladstone Harbour.

*Food intake and prey capture*. Food intake was examined in one laboratory toxicity study, showing a decrease with increasing Cd concentrations in *A*. *affinis* [136]. Prey capture is a behavioural biomarker that is associated with reduced fish growth [177]. *Fundulus heteroclitus* captured in contaminated sites showed a lower prey capture ability in laboratory experiments than fish from reference sites [177, 178].The reduced prey capture ability was strongly correlated with metal accumulation in liver tissue [177, 178]. Given the low number of studies on food intake and prey capture, and the requirement of experimental studies to assess their response, these biomarkers were not recommended for Gladstone Harbour.

*Phototactic response*. The phototactic response of fish larvae can be used as indicator of their physical condition [89]. This biomarker was examined in one laboratory study and showed a lower response in larvae hatched from eggs exposed to contaminated sediments compared to control larvae [89]. Given that this biomarker requires experimental studies to assess its response it was not recommended for use in Gladstone Harbour.

*Epidermal diseases*. The incidence of epidermal diseases in fish exposed to contaminants in mesocosm experimental systems increased in both contaminated and clean mesocosms tanks [148]. However, lymphocystis was higher in the contaminated mesocosm tank [148]. Given that only one study examined this biomarker, with unclear results, it was not recommended for use in Gladstone Harbour.

#### Biomarkers integrating exposure and effect

Traditionally, a suite of individual biomarkers has been used to assess biological impacts of contaminants on fish. New techniques such as microarray, proteomics and RNA sequencing (RNASeq) take fish health assessments beyond individual biomarkers to a global analysis of how all genes or proteins within an individual fish respond to contaminants [3, 149, 156]. Approaches such as microarray and proteomics not only extend the number of biomarkers analysed but also provide knowledge of exposure and pathways of injury, and potentially facilitate the identification of new more suitable individual biomarkers for routine monitoring programs [3, 156]. On the other hand, these approaches have the disadvantage of generating a wealth of information which can be difficult to interpret when there is no obvious toxic effect to the fish [3].

**Microarray and proteomics.** Our systematic review identified four papers which assessed fish health using microarray or proteomics [104, 149, 155, 156]. Microarray analysis of *P*. *flesus* exposed to reference and contaminated sediments showed significant changes in the hepatic abundance of 241 transcripts and 18 metabolites [155], and differential expression of 57 hepatic genes [156]. Endocrine microarray identified upregulation of five genes and downregulation of six genes, including CYP4501A and CYP450, in *S*. *aurata* exposed to contaminated sediment [104]. Based on proteomic analysis of plasma proteins from *S*. *aurata* exposed to Cu under laboratory conditions, 10 proteins were differentially expressed between controls and Cu exposed fish [149]. The ten differentially expressed proteins included the traditional biomarker proteins COX, ALT and GST, but also three new potential biomarkers of Cu exposure: growth hormone receptor, DNA recombinase complex and warm acclimation physiological response proteins [149]. Hence microarray or proteomics may be suitable for assessing fish health in Gladstone Harbour and reveal additional pathway impacted by contamination. However, specific relationships between gene transcripts or metabolic profiles and sediment contamination could not always be established [155], potentially due to the contaminants being present in a non-bioavailable form. Combined with the low number of studies to assess their effectiveness, this precludes the inclusion of these biomarkers for Gladstone Harbour until clear relationships have been established between contaminant exposure, changes in transcripts, metabolites and protein abundance, and early adverse effects in fish.

**RNASeq.** RNASeq is based on measuring the abundance of transcripts, but unlike microarray, is not limited to commercially available array chips or customising expensive individual chips, and does not require pre-existing knowledge of the fish genome [179]. This means that RNASeq can be used to assess responses to contaminants in non-model fish species [179], such as potential suitable fish species in Gladstone Harbour like *L*. *calcarifer* [157]. Our review, however, did not identify any papers that applied RNASeq to assess effects of metals on fish health. RNASeq may be a suitable biomarker for assessing fish health in Gladstone Harbour particularly given the existing knowledge of the *L calcarifer* transcriptome [157]. However, the application of this biomarker needs to be further development to provide clear connections between external levels of contaminant exposure, changes in transcripts abundances, and early adverse effects in organisms.

### Assessment of fish health biomarkers for Gladstone Harbour

**Fig 3:**
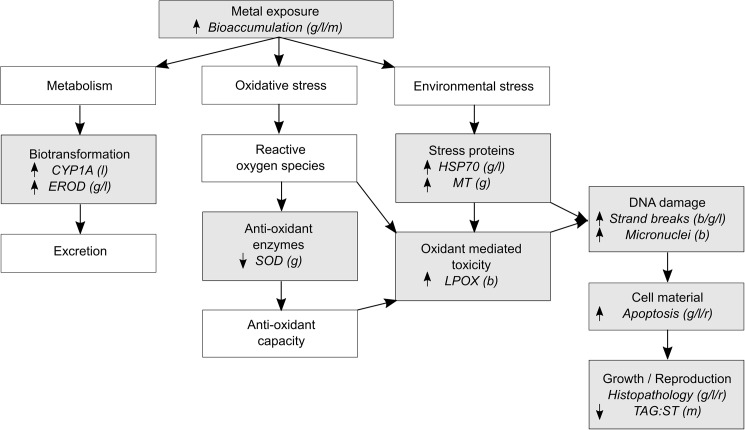
Flow diagram showing chemical, biochemical, physiological and other alterations in response to metal exposure. Biomarkers that have been identified in our systematic review as potentially suitable for fish health assessment in Gladstone Harbour are included in italics. For each biomarker, the arrow presents likely up- or downregulation following metal exposure; suitable tissues are given in between brackets: b = blood, g = gill, l = liver, m = muscle, r = gonads. Modified from [180] in [3].

Based on the metals being identified as contaminant of concern (Table 1), our review identified several biomarkers that would be suitable to assess fish health in Gladstone Harbour. These include bioaccumulation markers, biomarkers of exposure (e.g. CYP1A, EROD, SOD, LPOX, HSP70, MT, DNA strand breaks, micronucleus and nuclear abnormalities, apoptosis), and biomarkers of effect (e.g. histopathology, TAG:ST) (Fig 3). In addition, several other potential biomarkers were highlighted as having potential, but generally the number of studies was too low to provide a strong recommendation. These include biomarkers of exposure (e.g. VTG), biomarkers of effect (e.g. K index), and, in particular biomarkers that integrate exposure and effects, (e.g. RNASeq). Many biomarkers, however, exhibit variable responses depending on fish species, type of tissue analysed, location and time of sampling, and in some cases life history characteristics such as age and sex. Hence, targeted field assessments and controlled laboratory experiments need to be conducted with the species and biomarkers identified to verify their applicability and specificity for fish health assessments in Gladstone Harbour.

Our review highlighted that the pathway of metal exposure is critical in causing an effect, particularly whether metals are taken up through the water or the sediment. Hence, fish species recommended for Gladstone Harbour need to include both pelagic and demersal species. In addition, several biomarkers have already been developed for certain species identified as potentially suitable (Table 2), albeit not necessarily in the context of metal contamination. These include (i) CYP1A for *L*. *calcarifer* [93, 181] and *L*. *carponotatus* [182, 183]; (ii) EROD for *A*. *berda* [184], *L*. *calcarifer* [93, 181], *L*. *carponotatus* [182, 183], and *M*. *cephalus* [185, 186]; (iii) micronucleus test for *M*. *cephalus* [185]; (iv) VTG for *L*. *calcarifer* [152–154]; (v) transcriptome for *L*. *calcarifer* [157], and (vi) various gross indices for *L*. *calcarifer* [93] and *M*. *cephalus* [185]. Combined information on life history characteristics relevant to metal contamination (Table 2), and fish genera used in fish biomarker studies globally we recommend that five fish species be further considered for the assessment of fish health in Gladstone Harbour. These are the demersal *Acanthopagrus australis* (Yellowfin Bream), *Lates calcarifer* (Barramundi), and *Mugil cephalus* (Sea Mullet), and the pelagic *Eleutheronema tetradactylum* (Blue Threadfin), and *Scomberomorus queenslandicus* (School Mackerel).

## Conclusion

In this study, we present a protocol for identifying suitable biomarkers to assess fish health in coastal and marine ecosystems, using Gladstone Harbour (Australia) as a case study (Fig 1). To ensure that our use of the term ‘biomarker’ is consistent with that used in fish health assessments worldwide [5, 6], we formulated and used clear working definitions of biomarkers based on a review of the global literature [5–7, 9, 52–59] (S1 Table). We identified metals, specifically aluminium (Al), cadmium (Cd), copper (Cu), gallium (Ga), lead (Pb), selenium (Se), and zinc (Zn), as contaminants of concern in water and sediment of Gladstone Harbour (Table 1), based on point and diffuse sources of pollution [15] (S2 and S3 Tables) and available monitoring data[10, 16–31] (S4–S11 Tables). These definitions and contaminants of concern were subsequently used to inform our systematic literature review [8] (Fig 2; S1 Text), structure our findings, and assess the suitability of different biomarkers for monitoring fish health in Gladstone Harbour.

Our systematic review has identified various biomarkers that could be suitable for assessing fish health in Gladstone Harbour (Fig 3). In addition, our review has identified five fish species suitable for such biomarker studies, based on (i) abundance information from fisheries dependent [35–39] and independent [40–44] (S12 Table), (ii) exposure and life history information relevant to metal contamination [46–48] (http://www.fishbase.org/; http://australianmuseum.net.au/) (Table 2), and (iii) existence of suitable biomarkers [93, 152–154, 157, 181–186]. These five fish species are *Acanthopagrus australis* (Yellowfin Bream), *Lates calcarifer* (Barramundi), *Mugil cephalus* (Sea Mullet), *Eleutheronema tetradactylum* (Blue Threadfin), and *Scomberomorus queenslandicus* (School Mackerel). For two of these species (*L*. *calcarifer* and *M*. *cephalus*), many of the potentially suitable biomarkers have already been developed but will need to be verified for Gladstone Harbour conditions. For the other fish species, the potentially suitable biomarkers will need to be developed and subsequently verified for Gladstone Harbour.

Our protocol outlines a clear pathway to identify suitable biomarkers to assess fish health in coastal and marine ecosystems, which could be applied to biomarker studies in aquatic ecosystems around the world. Our results demonstrate that, while biomarkers have been used extensively in coastal and marine fish (S13–S23 Tables), none of the biomarkers reviewed have been specifically linked to adverse effects in organisms (i.e. growth, reproduction, and mortality) and populations (i.e. Adverse Outcome Pathways; [187, 188]. Thus, following the identification of suitable biomarkers and prioritised fish species, further targeted field studies and controlled laboratory experiments will need to be conducted to verify their applicability and specificity for fish health assessments in the ecosystem of interest.

## Supporting information

S1 Table Definitions of biomarkers. (DOCX)

S2 Table Facilities in the Gladstone region. (DOCX)

S3 Table Total emissions of 46 substances from 26 facilities. (DOCX)

S4 Table Inorganic elements, organometallics, metals and metalloids concentrations in Gladstone Harbour water. (DOCX)

S5 Table Inorganic elements, organometallics, metals and metalloids concentrations in Gladstone Harbour sediment. (DOCX)

S6 Table Polycyclic aromatic hydrocarbon (PAHs) concentrations in Gladstone Harbour sediment. (DOCX)

S7 Table Polychlorinated biphenyls (PCBs), chlorinated hydrocarbons and semi-volatile organic compounds (SVOCs) concentrations in Gladstone Harbour sediment. (DOCX)

S8 Table Total petroleum hydrocarbons (TPHs) and benzene, toluene, ethylbenzene and xylene (BTEX) concentrations in Gladstone sediment. (DOCX)

S9 Table Organochlorine pesticide concentrations in Gladstone Harbour sediment. (DOCX)

S10 Table Organophosphorus pesticide concentrations in Gladstone Harbour sediment. (DOCX)

S11 Table Herbicides, carbamate pesticides and insecticides concentrations in Gladstone Harbour sediment. (DOCX)

S12 Table Fish species in Gladstone Harbour. (DOCX)

S13 Table Summary of bioaccumulation studies. (DOCX)

S14 Table Summary of biomarker of exposure studies: biotransformation enzymes, Phase I. (DOCX)

S15 Table Summary of biomarker of exposure studies: biotransformation enzymes, Phase II. (DOCX)

S16 Table Summary of biomarker of exposure studies: oxidative stress parameters. (DOCX)

S17 Table Summary of biomarker of exposure studies: bio transformational products, stress proteins, metallothioneins and metal proteins. (DOCX)

S18 Table Summary of biomarker of exposure studies: haematological and immunological parameters. (DOCX)

S19 Table Summary of biomarker of exposure studies: reproductive, endocrine and neurotoxic parameters. (DOCX)

S20 Table Summary of biomarker of exposure studies: genotoxic parameters. (DOCX)

S21 Table Summary of biomarker of exposure studies: osmoregulatory and respiratory parameters. (DOCX)

S22 Table Summary of biomarker of effect studies: carbohydrate and aerobic metabolism. (DOCX)

S23 Table Summary of biomarker of effect studies: histopathology and gross indices. (DOCX)

S1 Text PRISMA Checklist for Systematic Review. (DOCX)
