# Supplementary material for: A protocol for identifying suitable biomarkers to assess fish health: A systematic review
Source: PLoS One. 2017 Apr 12;12(4):e0174762. doi: 10.1371/journal.pone.0174762 (PMC5389625; doi:10.1371/journal.pone.0174762)
Supplement: S1 Table — (DOCX) [file pone.0174762.s001.docx]

**S1 Table. Definitions of biomarkers used in the global scientific literature for assessments of fish health specifically, and aquatic ecosystem health more broadly.** General definitions of biomarkers are given, as well as those for biomarkers of exposure, of effect and of susceptibility, respectively.

| **Biomarker definitions** | **References** |
| --- | --- |
| **Biomarker (general)** |  |
| Detectable biochemical and tissue‐level changes that indicate altered physiology. | [1] (from [2]) |
| Detectable biochemical and tissue responses that represent changes in organisms after exposure to pollutants. | [2] (from [3]) |
| A biological response to a chemical or chemicals that gives a measure of exposure, and sometimes, also of toxic effect. | [4] (from [5]) |
| Biochemical, anatomical, physiological and behavioural responses that signal exposure to and/or adverse effects of anthropogenic chemicals and radiations. | [4] (see [6]) |
| Any measurement reflecting an interaction between a biological system and a potential hazard, which may be chemical, physical or biological. | [7](from [8]) |
| A change in a biological response (ranging from molecular through cellular and physiological responses to behavioral changes) which can be related to exposure to or toxic effects of environmental chemicals. | [7]( (from[6]) |
| Any biological response to an environmental chemical at the sub-individual level, measured inside an organism or in its products (urine, faeces, hair, feathers, etc.), indicating a deviation from the normal status that cannot be detected in the intact organism. | [7] (from [9]) |
| All biological (biochemical, physiological, histological and morphological) indicators measured inside an organism or its products. | [7] |
| A xenobiotically-induced variation in cellular or biochemical components or processes, structures, or functions that is measurable in a biological system or sample. | [10] (modified based on [11]) |
| A biological response (ranging from the molecular to community structure and even to the function and structure of ecosystems) to a chemical or chemicals that gives a measure of exposure and sometimes, also of toxic effect. | [6] |
| A biological response to a chemical or chemicals that gives a measure of exposure and sometimes, also of toxic effect. The types of 'biological responses' that can be considered range from the molecular to species composition. | [5] |
| Any biological response to an environmental chemical at the below-individual level, measured inside an organism or in its products (urine, faeces, hairs, feathers, etc.), indicating a departure from the normal status, that cannot be detected from the intact organism. Restrict the term 'biomarker' to biochemical, physiological, histological and morphological (including appearance, pigmentation, surface deformation, etc.) measurements of 'health' and exclude behavioural effects. | [9] |
| Almost any measurement reflecting an interaction between a biological system and a potential hazard, which may be chemical, physical or biological. The measured response may be functional and physiological, biochemical at the cellular level, or a molecular interaction. | [8] |
| Biochemical, physiological, or histological indicators of either exposure to, or effects of, xenobiotic chemicals at the suborganismal or organismal level. | [12] |
| Quantifiable biochemical, physiological, or histological measures that relate in a dose-dependent manner the degree of dysfunction that the contaminant has produced. | [3] |
| Indicators signalling events in biological systems or samples. | [11] |

| **Biomarker of exposure** |  |
| --- | --- |
| Show an early response to contaminants and are typically specific to a particular class of contaminants. | [1] |
| The internal dose or bioavailability of a particular xenobiotic or its metabolite in an organism. Should be well-characterized responses that take into account pharmacodynamic and physicochemical properties of the biological organism and agent, respectively (e.g. CYP1A, EROD). | [13] |
| Markers which indicate that exposure of an individual or organism to a xenobiotic has occurred and to what extent (e.g. particular metabolites or adducts indicating *interaction* with the *biological system)*. | [14] |
| The detection and measurement of an exogenous substance or its metabolite or the product of an interaction between a xenobiotic agent and some target molecule or cell that is measured in a compartment within an organism. | [7](from [11], [8]) |
| An exogenous substance or its metabolite or the product of an interaction between a xenobiotic agent and some target molecule or cell that is measured in a compartment within an organism. | [8] |
| The identification of an exogenous substance within the system, the interactive product between a xenobiotic compound and endogenous components, or other event in the biological system related to the exposure. | [11] |
| **Biomarker of effect** (also called **biomarker of response**) |  |
| Indicators of physiological or biochemical changes as a consequence of exposure. | [1] |
| Measurable biochemical, physiological or other alterations within tissues or body fluids of an organism that can be recognized as associated with an established or possible health impairment or disease. | [7](from [11], [8]) |
| Markers that can be measured at any point along the continuum from the molecular to the ecosystem level and may vary tremendously in their specificity (e.g. HSPs). | [13] |
| Indicators of biochemical change of actual or potential toxicological importance to an organism resulting from exposure to a xenobiotic (e.g. HSPs, cytochrome P450) | [14] |
| A measurable biochemical, physiological, behavioural or other alteration within an organism that, depending upon the magnitude, can be recognized as associated with an established or possible health impairment or disease. | [8] |
| An indicator of an endogenous component of the biological system, a measure of the functional capacity of the system, or an altered state of the system that is recognized as impairment or disease. | [11] |
| **Biomarker of susceptibility** |  |
| The inherent or acquired ability of an organism to respond to the challenge of exposure to a specific xenobiotic substance, including genetic factors and changes in receptors which alter the susceptibility of an organism to that exposure. | [7](from [11], [8]) |
| In contrast to biomarkers of effect or exposure, biomarkers of susceptibility do not represent stages along the dose-effect continuum, but are conditions that increase the rate of transition between the steps. | [13] |
| Markers which indicate that an organism may be more or less susceptible to adverse effects following exposure to a particular xenobiotic. | [14] |
| An indicator of an inherent or acquired ability of an organism to respond to the challenge of exposure to a specific xenobiotic substance. | [8] |
| An indicator that the health of the system is especially sensitive to the challenge of exposure to a xenobiotic compound (a compound originating outside the organism). | [11] |

# References

1. Hook SE, Gallagher EP, Batley GE. The Role of Biomarkers in the Assessment of Aquatic Ecosystem Health. Integr Environm Ass Manag. 2014; 10: 327-41. doi: 10.1002/ieam.1530 PMID: 000338133500004
2. Smit MGD, Bechmann RK, Hendriks AJ, Skadsheim A, Larsen BK, Baussant T, et al. Relating biomarkers to whole-organism effects using species sensitivity distributions: a pilot study for marine species exposed to oil. Environ Toxicol Chem. 2009; 28: 1104-9.
3. Mayer FL, Versteeg DJ, McKee MJ, l.C. F, R.L. G, D.C. M, et al. Physiological and nonspecific biomarkers. In: R.J. H, Kimerle RR, Mehrle PMJ, Bergman HL, editors. Biomarkers: Biochemical, Physiological, and Histological Markers of Anthropogenic Stress. Boca Raton, Fl, USA: Lewis Publishers; 1992. p. 5-85.
4. Handy RD, Galloway TS, Depledge MH. A proposal for the use of biomarkers for the assessment of chronic pollution and in regulatory toxicology. Ecotoxicol. 2003; 12: 331-43. doi: 10.1023/a:1022527432252 PMID: 000181129000028
5. Peakall DB, Walker CH. The role of biomarkers in environmental assessment (3). Vertebrates. Ecotoxicol. 1994; 3: 173-9.
6. Peakall DB. The role of biomarkers in environmental assessment (1). Introduction. Ecotoxicol. 1994; 3: 157-60.
7. van der Oost R, Beyer J, Vermeulen NPE. Fish bioaccumulation and biomarkers in environmental risk assessment: a review. Environ Toxicol Pharmacol. 2003; 13: 57-149. doi: 10.1016/s1382-6689(02)00126-6 PMID: 000180555100001
8. World Health Organization International Programme on Chemical Safety. Biomarkers and risk assessment: concepts and principles Geneva, Switzerland: World Health Organization; 1993 [cited 2016 25 July]. Available from: http://www.inchem.org/documents/ehc/ehc/ehc155.htm#SectionNumber:1.2
9. Van Gestel CA, Van Brummelen TC. Incorporation of the biomarker concept in ecotoxicology calls for a redefinition of terms. Ecotoxicol. 1996; 5: 217-25. doi: 10.1007/BF00118992
10. McCarty LS, Power M, Munkittrick KR. Bioindicators versus biomarkers in ecological risk assessment. Hum Ecol Risk Ass. 2002; 8: 159-64. PMID: 000173786800014
11. Committee on Biological Markers of the National Research Council. Biological markers in environmental health research. Environ Health Perspect. 1987; 74: 3-9.
12. Huggett RJ, Stegeman JJ, Page DS, Parker KR, Woodin B, Brown JS. Biomarkers in fish from Prince William Sound and the Gulf of Alaska: 1999-2000. Environ Sci Technol. 2003; 37: 4043-51. doi: 10.1021/es0342401 PMID: 000185326700005
13. Schlenk D. Necessity of Defining Biomarkers for Use in Ecological Risk Assessments. Mar Pollut Bull. 1999; 39: 48-53. doi: 10.1016/S0025-326X(99)00015-6.
14. Editor. Editorial. Biomarkers. 1996; 1: 1-2. doi: 10.3109/13547509609079340.
