# Supplementary material for: A protocol for identifying suitable biomarkers to assess fish health: A systematic review
Source: PLoS One. 2017 Apr 12;12(4):e0174762. doi: 10.1371/journal.pone.0174762 (PMC5389625; doi:10.1371/journal.pone.0174762)
Supplement: S2 Table — (DOCX) [file pone.0174762.s002.docx]

**S2 Table. Facilities in the Gladstone region with emissions reported in the National Pollutant Inventory.** Data from the National Pollutant Inventory ([www.npi.gov.au](http://www.npi.gov.au)).

| **Registered Business Name** | **Main activities** | **Facility Name** | **Suburb** |
| --- | --- | --- | --- |
| Aurizon Operations Ltd | Locomotive fuelling | [Barney Point Rail Fuelling Facility](http://www.npi.gov.au/npidata/action/load/individual-facility-detail/criteria/state/QLD/year/2015/jurisdiction-facility/Q042QRL017) | Gladstone |
| Aurizon Operations Ltd | Locomotive servicing and fuelling | [Callemondah Rail Yard (Fuelling Facility)](http://www.npi.gov.au/npidata/action/load/individual-facility-detail/criteria/state/QLD/year/2015/jurisdiction-facility/Q042QRL002) | Callemondah |
| Boyne Smelters Ltd | Aluminium smelting (from alumina) | [BSL](http://www.npi.gov.au/npidata/action/load/individual-facility-detail/criteria/state/QLD/year/2015/jurisdiction-facility/Q017BSL001) | Gladstone |
| BP Australia | Bulk petroleum storage facility | [Gladstone Terminal](http://www.npi.gov.au/npidata/action/load/individual-facility-detail/criteria/state/QLD/year/2015/jurisdiction-facility/Q022BPA001) | Gladstone |
| Caltex Australia petroleum Pty Ltd | Petroleum product wholesaling | [Caltex Terminal Gladstone](http://www.npi.gov.au/npidata/action/load/individual-facility-detail/criteria/state/QLD/year/2015/jurisdiction-facility/Q022CAL028) | Gladstone |
| Cement Australia (Queensland) Pty Ltd | Limestone mining, crushing and preparation of raw meal for cement manufacture | [East End Mine](http://www.npi.gov.au/npidata/action/load/individual-facility-detail/criteria/state/QLD/year/2015/jurisdiction-facility/Q050QCL003) | Mount Larcom |
| Cement Australia (Queensland) Pty Ltd | Cement Manufacture | [Fishermans Landing](http://www.npi.gov.au/npidata/action/load/individual-facility-detail/criteria/state/QLD/year/2015/jurisdiction-facility/Q050QCL002) | Gladstone |
| Central combined group Pty Ltd | Petroleum product wholesaling | [Gladstone](http://www.npi.gov.au/npidata/action/load/individual-facility-detail/criteria/state/QLD/year/2015/jurisdiction-facility/Q022CCG001) | Galdstone |
| Coogee chemicals Pty Ltd | Sulphuric acid terminal | [Coogee Chemicals Pty Ltd, Yarwun](http://www.npi.gov.au/npidata/action/load/individual-facility-detail/criteria/state/QLD/year/2015/jurisdiction-facility/Q038COO003) | Yarwun |
| Earth Commodities Gladstone Pty Ltd | Drill & Blast, Load & Haul, Crushing & Screening, Stockpiling and Loading Trucks | [Gladstone Quarry](http://www.npi.gov.au/npidata/action/load/individual-facility-detail/criteria/state/QLD/year/2015/jurisdiction-facility/Q056EAR001) | Yarwun |
| Elgas Ltd | n/a | [Gladstone AU123](http://www.npi.gov.au/npidata/action/load/individual-facility-detail/criteria/state/QLD/year/2015/jurisdiction-facility/Q022ELG008) | Gladstone |
| Gladstone Port Corporation Ltd | Wharf operation | [Port Central](http://www.npi.gov.au/npidata/action/load/individual-facility-detail/criteria/state/QLD/year/2015/jurisdiction-facility/Q080GPA005) | Gladstone |
| International Bunker Supplies Pty Ltd | Marine fuel oil storage facility only (do not burn any fuel, only store) | [INTERNATIONAL BUNKER SUPPLIES PTY LTD](http://www.npi.gov.au/npidata/action/load/individual-facility-detail/criteria/state/QLD/year/2015/jurisdiction-facility/Q022IBS001) | Gladstone |
| Jemena Queensland gas pipeline (1) Pty Ltd, and Jemena Queensland gas pipeline (2) Pty Ltd | Gas Pipeline | [Gladstone Meter Station (Queensland Gas Pipeline)](http://www.npi.gov.au/npidata/action/load/individual-facility-detail/criteria/state/QLD/year/2015/jurisdiction-facility/Q012DAO015) | Gladstone |
| Jemena Queensland gas pipeline (1) Pty Ltd, and Jemena Queensland gas pipeline (2) Pty Ltd | Natural Gas Metering | [Larcom Creek (Tee)Check Meter Station (Queensland Gas Pipeline)](http://www.npi.gov.au/npidata/action/load/individual-facility-detail/criteria/state/QLD/year/2015/jurisdiction-facility/Q012DAO014) | Mount Larcom |
| Jemena Queensland gas pipeline (1) Pty Ltd, and Jemena Queensland gas pipeline (2) Pty Ltd | Natural Gas Metering | [Orica Meter Station (Queensland Gas Pipeline)](http://www.npi.gov.au/npidata/action/load/individual-facility-detail/criteria/state/QLD/year/2015/jurisdiction-facility/Q012DAO021) | Gladstone |
| Jemena Queensland gas pipeline (1) Pty Ltd, and Jemena Queensland gas pipeline (2) Pty Ltd | Natural Gas Metering | [QAL + Boyne Meter Station (Queensland Gas Pipeline)](http://www.npi.gov.au/npidata/action/load/individual-facility-detail/criteria/state/QLD/year/2015/jurisdiction-facility/Q012DAO020) | Gladstone |
| Northern oil refineries Pty Ltd | Used oil recycling | [Northern Oil Refinery](http://www.npi.gov.au/npidata/action/load/individual-facility-detail/criteria/state/QLD/year/2015/jurisdiction-facility/Q013NOR001) | Yarwun |
| NRG Gladstone operating services Pty Ltd | Electricity generation using coal or coal derived products | [Gladstone Power Station](http://www.npi.gov.au/npidata/action/load/individual-facility-detail/criteria/state/QLD/year/2015/jurisdiction-facility/Q019NRG001) | Gladstone |
| Orica Australia Pty Ltd | Production & storage of:- Ammonium Nitrate, Nitric Acid, Sodium Cyanide, Chlorine, Hydrochloric Acid, Caustic Soda, Sodium Hypchlorite and Expanded Polystyrene Beads | [Yarwun Site](http://www.npi.gov.au/npidata/action/load/individual-facility-detail/criteria/state/QLD/year/2015/jurisdiction-facility/Q038ORI001) | Yarwun Via Gladstone |
| QC LNG Operating company Pty Ltd | LNG Manufacturing | [Curtis Island LNG Plant](http://www.npi.gov.au/npidata/action/load/individual-facility-detail/criteria/state/QLD/year/2015/jurisdiction-facility/Q012QGC015) | Curtis Island |
| QER Pty Ltd | Care and maintenance predominantly. Minimal shale mining, retorting using the Technology Demonstration Plant | [Stuart Project](http://www.npi.gov.au/npidata/action/load/individual-facility-detail/criteria/state/QLD/year/2015/jurisdiction-facility/Q020SEM001) | Gladstone |
| Queensland Alumina Ltd | Bauxite refining | [QAL](http://www.npi.gov.au/npidata/action/load/individual-facility-detail/criteria/state/QLD/year/2015/jurisdiction-facility/Q016QAL001) | Gladstone |
| RTA Yarwun PTY LTD | Alumina refining | [RTA Yarwun Pty Ltd](http://www.npi.gov.au/npidata/action/load/individual-facility-detail/criteria/state/QLD/year/2015/jurisdiction-facility/Q016COM001) | Gladstone |
| Sibelco Australia Ltd | (Extractive Industry) Mining, Crushing and Screening of Limestone to produce Agricultural, Concrete Aggregate and Roadbase Products | [Calliope Quarry](http://www.npi.gov.au/npidata/action/load/individual-facility-detail/criteria/state/QLD/year/2015/jurisdiction-facility/Q056UAL003) | Calliope |
| Trility Pty Ltd | Water Treatment | [Agnes Water / 1770 Water Treatment Plant](http://www.npi.gov.au/npidata/action/load/individual-facility-detail/criteria/state/QLD/year/2015/jurisdiction-facility/Q046UUA001) | Agnes Water |
| Wiggins Island Coal Export Terminal Pty Ltd | Coal export terminal | [WIGGINS ISLAND COAL EXPORT TERMINAL PTY LTD](http://www.npi.gov.au/npidata/action/load/individual-facility-detail/criteria/state/QLD/year/2015/jurisdiction-facility/Q080WIC001) | Callemondah |
