# Supplementary material for: A protocol for identifying suitable biomarkers to assess fish health: A systematic review
Source: PLoS One. 2017 Apr 12;12(4):e0174762. doi: 10.1371/journal.pone.0174762 (PMC5389625; doi:10.1371/journal.pone.0174762)
Supplement: S3 Table — (DOCX) [file pone.0174762.s003.docx]

**S3 Table. Total emissions of 46 substances into air, land and water from 26 facilities located around Gladstone Harbour in 2014/2015.** Data from the National Pollutant Inventory ([www.npi.gov.au](http://www.npi.gov.au)). Discharges from coastal river basins into Gladstone Harbour are not included in ammonia, total nutrient and total phosphorus emissions.

| **Contaminant** | **Total emissions (kg) for 2014/15** | | |
| --- | --- | --- | --- |
|  | **Air** | **Land** | **Water** |
| Acetaldehyde | 80,000 | 0 | 0 |
| Acetic acid (ethanoic acid) | 20 | 0 | 0 |
| Ammonia (total) | 46,000 | 0 | 5,300 |
| Antimony and compounds | 12 | 0 | 0 |
| Arsenic and compounds | 300 | 14 | 470 |
| Benzene | 35,000 | 0 | 0.0010 |
| Beryllium and compounds | 14 | 0.048 | 0 |
| Boron and compounds | 14,000 | 1,300 | 0 |
| 1,3-Butadiene (vinyl ethylene) | 0.31 | 0 | 0 |
| Cadmium and compounds | 100 | 0.079 | 26 |
| Carbon monoxide | 43,000,000 | 0 | 0 |
| Chlorine and compounds | 8 | 0 | 900 |
| Chromium (III) compounds | 1,400 | 0.11 | 21 |
| Chromium (IV) compounds | 15 | 0.34 | 0.67 |
| Cobalt and compounds | 120 | 6.8 | 0.097 |
| Copper and compounds | 2,800 | 1.0 | 430 |
| Cumene (1-methylethylbenzene) | 870 | 0 | 0 |
| Cyanide (inorganic) compounds | 110 | 0 | 17 |
| Cyclohexane | 770 | 0 | 0 |
| Ethylbenzene | 470 | 0 | 0.0020 |
| Fluoride compounds | 690,000 | 430 | 160,000 |
| Formaldehyde (methyl aldehyde) | 200,000 | 0 | 0 |
| n-hexane | 5,700 | 0 | 0 |
| Hydrochloric acid | 820,000 | 0 | 0 |
| Lead and compounds | 700 | 0.54 | 28 |
| Magnesium oxide fume | 10 | 0 | 0 |
| Manganese and compounds | 5,100 | 840 | 690 |
| Mercury and compounds | 160 | 0.052 | 0.42 |
| Methyl ethyl ketone | 300 | 0 | 0 |
| Nickel and compounds | 2,100 | 4.9 | 39 |
| Oxides of nitrogen | 46,000,000 | 0 | 0 |
| Particulate matter 10 µm | 3,600,000 | 0 | 0 |
| Particulate matter 2.5 µm | 750,000 | 0 | 0 |
| Polychlorinated dioxins and furans | 0.0010 | 0 | 0 |
| Polycyclic aromatic hydrocarbons (B[a]Peq) | 39 | 0 | 0.67 |
| Selenium and compounds | 60 | 0 | 0 |
| Styrene (ethenylbenzene) | 0.70 | 0 | 0 |
| Sulfur dioxide | 40,000,000 | 0 | 0 |
| Sulfuric acid | 320,000 | 0 | 0 |
| Toluene (methylbenzene) | 31,000 | 0 | 0.0050 |
| Total nitrogen | 0 | 0 | 155,100 |
| Total phosphorus | 0 | 0 | 14,950 |
| Total volatile organic compounds | 1,400,000 | 0 | 0 |
| Trichloroethylene | 23,000 | 0 | 0 |
| Xylenes (individual or mixed isomers) | 4,700 | 0 | 0.0080 |
| Zinc and compounds | 4,900 | 9.2 | 1,000 |
