# Supplementary material for: A protocol for identifying suitable biomarkers to assess fish health: A systematic review
Source: PLoS One. 2017 Apr 12;12(4):e0174762. doi: 10.1371/journal.pone.0174762 (PMC5389625; doi:10.1371/journal.pone.0174762)
Supplement: S4 Table — (DOCX) [file pone.0174762.s004.docx]

**S4 Table. Inorganic elements, organometallics, metals and metalloids concentrations (µg L^-1^) in Gladstone Harbour water based on publicly available water quality data.**

| **Contaminant** | **Guide-line value*** | **Apte *et al.* 2005 [1]** | | | | **Angel *et al.* 2010^±^ [2]** | | | | **Angel *et al*. 2012^±^ [3]** | | | | **DEHP 2012 [4]** | | | | **Kroon *et al*. 2015 [5]** | | | |
| --- | --- | --- | --- | --- | --- | --- | --- | --- | --- | --- | --- | --- | --- | --- | --- | --- | --- | --- | --- | --- | --- |
|  |  | # of samples | | Concentration | | # of samples | | Concentration | | # of samples | | Concentration | | # of samples | | Concentration | | # of samples | | Concentration | |
|  |  | Tested | > LOR | Min | Max | Tested | > LOR | Min | Max | Tested | > LOR | Min | Max | Tested | > LOR | Min | Max | Tested | > LOR | Min | Max |
| Chlorine | - |  |  |  |  |  |  |  |  |  |  |  |  |  |  |  |  |  |  |  |  |
| Cyanide | 4 | 25 | 0 |  |  |  |  |  |  |  |  |  |  |  |  |  |  |  |  |  |  |
| Fluoride | - | 100 | 100 | 1,110 | 1,320 |  |  |  |  |  |  |  |  |  |  |  |  |  |  |  |  |
| Tributyltin* | 0.006 | 7 | 5 | **0.009** | **0.021** |  |  |  |  |  |  |  |  |  |  |  |  |  |  |  |  |
| Aluminium | 24^¥^ | 100 | 11 | 1 | **34** |  |  |  |  | 32 | 32 | 1 | **330** | 244 | 39 | 10 | **61** | ns | ns | 2.5 | **560** |
| Antimony | - |  |  |  |  |  |  |  |  |  |  |  |  | 244 | 0 | nd | nd |  |  |  |  |
| Arsenic | - | 100 | 100 | 0.51 | 1.27 |  |  |  |  | 32 | 32 | 0.68 | 1.12 | 244 | 244 | 0.9 | 2.7 | ns | ns | 0.1 | 4.7 |
| Barium | - |  |  |  |  |  |  |  |  |  |  |  |  |  |  |  |  |  |  |  |  |
| Beryllium | - |  |  |  |  |  |  |  |  |  |  |  |  |  |  |  |  |  |  |  |  |
| Cadmium | 5.5 | 100 | 56 | 0.004 | 0.110 | 30 | 30 | 0.002 | 0.038 | 32 | 32 | 0.003 | 0.009 | 244 | 3 | 0.2 | 0.2 | ns | ns | 0.1 | 0.6 |
| Chromium | 24.7 | 100 | 0 | nd | nd |  |  |  |  | 32 | 0 | nd | nd | 244 | 4 | 0.5 | 0.9 | ns | ns | 0.5 | 3.1 |
| Cobalt | 1 |  |  |  |  |  |  |  |  | 32 | 32 | 0.022 | 0.171 | 244 | 23 | 0.2 | 0.4 | ns | ns | 0.5 | **1.3** |
| Copper | 1.3 | 100 | 100 | 0.05 | 1.18 | 30 | 30 | 0.019 | 0.650 | 32 | 32 | 0.588 | 1.060 | 244 | 29 | 1 | **2** | ns | ns | 0.11 | **6.9** |
| Gallium | - |  |  |  |  |  |  |  |  |  |  |  |  | 36 | 14 | 1 | 5.8 | ns | ns | 0.5 | 4.3 |
| Iron | - | 100 | 2 | 15 | 16 |  |  |  |  | 32 | 5 | 1.5 | 12 | 244 | 39 | 5 | 110 | ns | ns | 0.5 | 380 |
| Lead | 4.4 | 100 | 9 | 0.08 | 0.77 |  |  |  |  | 32 | 5 | 0.016 | 0.085 | 244 | 5 | 0.2 | 2.1 | ns | ns | 0.5 | 2.3 |
| Manganese | - | 100 | 35 | 0.6 | 7 | 30 | 21 | 0.400 | 7.000 | 32 | 29 | 0.2 | 6.5 | 244 | 226 | 0.5 | 82.3 | ns | ns | 0.3 | 400 |
| Mercury | 0.4 |  |  |  |  |  |  |  |  |  |  |  |  | 244 | 0 | nd | nd | ns | ns | 0.03 | 0.11 |
| Molybdenum | - |  |  |  |  |  |  |  |  |  |  |  |  | 244 | 244 | 0.4 | 32.4 | ns | ns | 0.5 | 41 |
| Nickel | 70 | 100 | 82 | 0.19 | 0.66 | 30 | 30 | 0.150 | 0.910 | 32 | 32 | 0.345 | 0.823 | 244 | 71 | 0.5 | 1.7 | ns | ns | 0.5 | 3.6 |
| Selenium | - | 100 | 100 | 0.04 | 0.38 |  |  |  |  |  |  |  |  | 244 | 3 | 2 | 2 | ns | ns | 0.5 | 6 |
| Silver | 1.4 |  |  |  |  |  |  |  |  |  |  |  |  | 244 | 6 | 0.1 | 0.2 | ns | ns | 0.3 | **5** |
| Strontium | - |  |  |  |  |  |  |  |  |  |  |  |  |  |  |  |  |  |  |  |  |
| Tin | - |  |  |  |  |  |  |  |  |  |  |  |  | 244 | 3 | 5 | 5 | ns | ns | 0.5 | 3.5 |
| Uranium | - |  |  |  |  |  |  |  |  |  |  |  |  |  |  |  |  | ns | ns | 0.5 | 5.6 |
| Vanadium | 100 |  |  |  |  |  |  |  |  |  |  |  |  | 244 | 244 | 0.9 | 6.8 | ns | ns | 0.5 | 11 |
| Zinc | 15 | 100 | 46 | 0.22 | 1.35 | 30 | 30 | 0.055 | 0.260 | 32 | 32 | 0.109 | 1.620 | 244 | 12 | 5 | **25** | ns | ns | 0.5 | **24** |

^*^ANZECC/ARMCANZ 2000 [6];^¥^ Guideline value from Golding et al. 2015 [7]; Bold font indicates value exceeds guideline value; * units are µg Sn L^-1^; # = number; ^±^ = samples from The Narrows and Port Curtis only. Abbreviations: LOR = limit of reporting; Min = minimum; Max = maximum; nd = not detected; ns = not specified.

# References

1. Apte S, Duivenvoorden L, Johnson R, Jones MA, Revill A, Simpson S, et al. Contaminants in Port Curtis: screening level risk assessment. Indooroopilly, Australia: Cooperative Research Centre for Coastal Zone, Estuary and Waterway Management, 2005.
2. Angel BM, Hales LT, Simpson SL, Apte SC, Chariton AA, Shearer DA, et al. Spatial variability of cadmium, copper, manganese, nickel and zinc in the Port Curtis Estuary, Queensland, Australia. Mar Freshw Res. 2010; 61: 170-83. doi: 10.1071/MF09046
3. Angel BM, Jarolimek CV, King JJ, Hales LT, Simpson SL, Jung RF, et al. Metal Concentrations in the Waters and Sediments of Port Curtis, Queensland. Sydney, Australia: CSIRO Wealth from Oceans Flagship, 2012.
4. Department of Environment and Heritage Protection. Update on the quality of sediment from Port Curtis and Tributaries. Brisbane, Australia: Department of Environment and Heritage Protection, 2012 Number 6, ISSN 1834-3910.
5. Kroon FJ, Berry KLE, Brinkman DL, Davis A, King O, Kookana R, et al. Identification, impacts, and prioritisation of emerging contaminants present in the GBR and Torres Strait marine environments. Final Report Project 1.10. Cairns, Australia: Report to the National Environmental Science Programme. Reef and Rainforest Research Centre Limited, 2015.
6. ANZECC/ARMCANZ (Australian and New Zealand Environment and Conservation Council and Agriculture and Resource Management Council of Australia and New Zealand). National Water Quality Management Strategy, Paper No. 4 - Australian and New Zealand Guidelines for Fresh and Marine Water Quality. Canberra, Australia: ANZECC/ARMCANZ, 2000.
7. Golding LA, Angel BM, Batley GE, Apte SC, Krassoi R, Doyle CJ. Derivation of a water quality guideline for aluminium in marine waters. Environ Toxicol Chem. 2015; 34: 141-51. doi: 10.1002/etc.2771
