# Supplementary material for: A protocol for identifying suitable biomarkers to assess fish health: A systematic review
Source: PLoS One. 2017 Apr 12;12(4):e0174762. doi: 10.1371/journal.pone.0174762 (PMC5389625; doi:10.1371/journal.pone.0174762)
Supplement: S5 Table — (DOCX) [file pone.0174762.s005.docx]

**S5 Table. Inorganic elements, organometallics, metals and metalloids concentrations (mg kg^-1^) in Gladstone Harbour sediment based on publicly available data.**

| **Contaminant** | **Guideline value^€^** | | **Apte *et al.* 2005 [1]** | |  |  | **Vicente-Beckett *et al.* 2006 [2]** | | | | **GHD *et al*. 2009 [3]** | |  |  | **DERM 2011 [4]** | |  |  |
| --- | --- | --- | --- | --- | --- | --- | --- | --- | --- | --- | --- | --- | --- | --- | --- | --- | --- | --- |
|  | **low** | **high** | **# of samples** | | **Concentration** | | **# of samples** | | **Concentration** | | **# of samples** | | **Concentration** | | **# of samples** | | **Concentration** | |
|  |  |  | **Tested** | **> LOR** | **Min** | **Max** | **Tested** | **> LOR** | **Min** | **Max** | **Tested** | **> LOR** | **Min** | **Max** | **Tested** | **> LOR** | **Min** | **Max** |
| Chlorine | - | - |  |  |  |  |  |  |  |  |  |  |  |  |  |  |  |  |
| Cyanide | - | - |  |  |  |  |  |  |  |  |  |  |  |  |  |  |  |  |
| Fluoride | - | - |  |  |  |  |  |  |  |  |  |  |  |  |  |  |  |  |
| Tributyltin* | 9.0 | 70 | 56 | 8 | **36** | **655** |  |  |  |  | 839 | 3 | 0.0031 | 0.9 |  |  |  |  |
| Aluminium | - | - |  |  |  |  | 152 | 152 | 14200 | 83551 | 1024 | 1044 | 530 | 20600 | 17 | 17 | 5900 | 43500 |
| Antimony | 2.0 | 25 | 100 | 96 | 0.24 | 1.09 | 82 | 82 | 0.312 | 0.75 | 1044 | 0 | nd | nd | 17 | 0 | nd | nd |
| Arsenic | 20 | 70 | 100 | 100 | 5 | **124** | 152 | 152 | 6.35 | **34.8** | 1044 | 976 | 1.08 | **49.5** | 17 | 17 | 7 | **42** |
| Barium | - | - |  |  |  |  |  |  |  |  |  |  |  |  | 17 | 17 | 11 | 380 |
| Beryllium | - | - |  |  |  |  |  |  |  |  |  |  |  |  | 17 | 17 | 0.11 | 0.83 |
| Cadmium | 1.5 | 10 | 100 | 2 | 0.11 | 0.24 | 152 | 152 | 0.022 | 0.226 | 1044 | 12 | 0.1 | 2.4 | 17 | 1 | 0.9 | 0.9 |
| Chromium | 80 | 370 | 100 | 100 | 8 | **251** | 152 | 152 | 14.4 | **90.5** | 1044 | 1044 | 1.1 | 33.1 | 17 | 17 | 8 | 42 |
| Cobalt | - | - |  |  |  |  |  |  |  |  | 1024 | 1024 | 0.6 | 77.4 | 17 | 17 | 4.5 | 16.0 |
| Copper | 65 | 270 | 100 | 100 | 3 | 45 | 152 | 152 | 8.1 | 54.2 | 1044 | 1041 | 1.2 | **171** | 17 | 14 | 3 | 49 |
| Gallium | - | - |  |  |  |  |  |  |  |  |  |  |  |  |  |  |  |  |
| Iron | - | - |  |  |  |  | 152 | 152 | 14600 | 51300 | 1024 | 1024 | 560 | 76000 |  | 17 | 13000 | 73000 |
| Lead | 50 | 220 | 100 | 100 | 3 | 18 | 152 | 152 | 5.75 | 22.60 | 1044 | 1006 | 1 | 43 | 17 | 17 | 4 | 9 |
| Manganese | - | - |  |  |  |  |  |  |  |  | 1044 | 1033 | 10 | 7680 | 17 | 17 | 82 | 1000 |
| Mercury | 0.15 | 1.0 | 100 | 100 | 0.9^¶^ | 55.3^¶^ | 82 | 82 | 0.020 | 0.065 | 1044 | 526 | 0.01 | **0.75** | 17 | 0 | nd | nd |
| Molybdenum | - | - |  |  |  |  |  |  |  |  |  |  |  |  | 17 | 14 | 0.5 | 2.8 |
| Nickel | 21 | 52 | 100 | 100 | 2 | **35** | 152 | 150 | 8.21 | **50.9** | 1044 | 1033 | 1.1 | **49** | 17 | 17 | 4 | **21** |
| Selenium | - | - |  |  |  |  |  |  |  |  | 1024 | 945 | 0.1 | 5.4 | 17 | 0 | nd | nd |
| Silver | 1.0 | 4.0 | 100 | 21 | 0.1 | 0.5 | 152 | 152 | 0.050 | 0.744 | 1024 | 11 | 0.1 | 0.6 |  |  |  |  |
| Strontium | - | - |  |  |  |  |  |  |  |  |  |  |  |  |  |  |  |  |
| Tin | - | - |  |  |  |  |  |  |  |  |  |  |  |  | 17 | 10 | 2 | 4 |
| Uranium | - | - |  |  |  |  |  |  |  |  |  |  |  |  |  |  |  |  |
| Vanadium | - | - |  |  |  |  |  |  |  |  | 1024 | 1022 | 5.6 | 287 | 17 | 17 | 25 | 160 |
| Zinc | 200 | 410 | 100 | 100 | 6 | 113 | 152 | 149 | 24.2 | 108 | 1044 | 1041 | 2 | 136 | 17 | 17 | 11 | 87 |

* Simpson et al. 2013 [5]; Bold font indicates value exceeds guideline value; ^#^ unit is µg Sn kg^-1^ and values are normalised to total organic carbon; ^¶^ unit is µg kg^-1^; Abbreviations: LOR = limit of reporting; Min = minimum; Max = maximum; nd = not detected; ns = not specified.

**S5 Table. Continued**

| **Contaminant** | **Guideline value^*^** | | **Angel *et al.* 2012^±^ [6]** | | | | **DEHP 2012 [4]** | | | | **Kroon *et al*. 2015 [7]** | | | |
| --- | --- | --- | --- | --- | --- | --- | --- | --- | --- | --- | --- | --- | --- | --- |
|  | **low** | **high** | **# of samples** | | **Concentration** | | **# of samples** | | **Concentration** | | **# of samples** | | **Concentration** | |
|  |  |  | **Tested** | **> LOR** | **Min** | **Max** | **Tested** | **> LOR** | **Min** | **Max** | **Tested** | **> LOR** | **Min** | **Max** |
| Chlorine | - | - |  |  |  |  |  |  |  |  |  |  |  |  |
| Cyanide | - | - |  |  |  |  |  |  |  |  |  |  |  |  |
| Fluoride | - | - |  |  |  |  | 31 | 31 | 50 | 280 |  |  |  |  |
| Tributyltin^#^ | 9.0 | 70 |  |  |  |  | 31 | 5 | 1.1 | 2.0 |  |  |  |  |
| Aluminium | - | - | 19 | 19 | 1720 | 26900 | 31 | 31 | 1920 | 20200 | ns | ns | 1100 | 38600 |
| Antimony | 2.0 | 25 | 19 | 19 | 0.08 | 0.35 | 31 | 0 | nd | nd |  |  |  |  |
| Arsenic | 20 | 70 | 19 | 19 | 7 | **54** | 31 | 31 | 3.45 | **25.7** | ns | ns | 2.5 | **34** |
| Barium | - | - | 19 | 19 | 9 | 82 |  |  |  |  |  |  |  |  |
| Beryllium | - | - | 19 | 19 | 0.22 | 0.79 |  |  |  |  |  |  |  |  |
| Cadmium | 1.5 | 10 | 19 | 19 | 0.11 | 0.44 | 31 | 0 | nd | nd | ns | ns | 0.3 | **3.7** |
| Chromium | 80 | 370 | 19 | 19 | 7 | 32 | 31 | 31 | 4.3 | 29.8 | ns | ns | 4 | 45 |
| Cobalt | - | - | 19 | 19 | 6 | 31 | 31 | 31 | 2.6 | 15.2 | ns | ns | 2 | 25 |
| Copper | 65 | 270 | 19 | 19 | 2 | 22 | 31 | 31 | 1.5 | 44.4 | ns | ns | 1 | 52 |
| Gallium | - | - | 19 | 19 | 2 | 10 |  |  |  |  | ns | ns | 0.3 | 17 |
| Iron | - | - | 19 | 19 | 12300 | 62600 | 31 | 31 | 8490 | 34300 | ns | ns | 3800 | 42700 |
| Lead | 50 | 220 | 19 | 19 | 3 | 13 | 31 | 31 | 1.6 | 11.4 | ns | ns | 1.1 | 26 |
| Manganese | - | - | 19 | 19 | 74 | 1330 | 31 | 31 | 82 | 766 | ns | ns | 21 | 1330 |
| Mercury | 0.15 | 1.0 | 19 | 3 | 0.02 | 0.05 | 31 | 14 | 0.01 | **1.37** | ns | ns | 0.1 | 0.1 |
| Molybdenum | - | - | 19 | 19 | 0.4 | 2 |  |  |  |  | ns | ns | 0.25 | 5.4 |
| Nickel | 21 | 52 | 19 | 19 | 4 | 16 | 31 | 31 | 2.1 | 16.9 | ns | ns | 1 | **31** |
| Selenium | - | - | 19 | 19 | 0.05 | 0.51 | 31 | 31 | 0.1 | 0.9 | ns | ns | 0.25 | 1.6 |
| Silver | 1.0 | 4.0 | 19 | 19 | 0.01 | 0.07 | 31 | 1 | 0.3 | 0.3 | ns | ns | 0.25 | 0.5 |
| Strontium | - | - | 19 | 19 | 28 | 576 |  |  |  |  |  |  |  |  |
| Tin | - | - |  |  |  |  |  |  |  |  | ns | ns | 0.25 | 2 |
| Uranium | - | - |  |  |  |  |  |  |  |  |  |  | 0.3 | 3.3 |
| Vanadium | - | - | 19 | 19 | 27 | 114 | 31 | 31 | 14.7 | 71 | ns | ns | 11 | 94 |
| Zinc | 200 | 410 | 19 | 19 | 18 | 57 | 31 | 31 | 7.5 | 78.2 | ns | ns | 3 | 100 |

* Simpson et al. 2013 [5]; Bold font indicates value exceeds guideline value; ^#^ unit is µg Sn kg^-1^ and values are normalised to total organic carbon; ^¶^ unit is µg kg^-1^; Abbreviations: LOR = limit of reporting; Min = minimum; Max = maximum; nd = not detected; ns = not specified.

# References

1. Apte S, Duivenvoorden L, Johnson R, Jones MA, Revill A, Simpson S, et al. Contaminants in Port Curtis: screening level risk assessment. Indooroopilly, Australia: Cooperative Research Centre for Coastal Zone, Estuary and Waterway Management, 2005.
2. Vicente-Beckett V, Shearer D, Munksgaard N, Hancock G, Morrison H. Metal and polycyclic aromatic hydrocarbon contaminants in benthic sediments of Port Curtis. Indooroopilly, QLD: Cooperative Research Centre for coastal zone, estuary and waterway management., 2006 Technical report 73.
3. GHD Pty Ltd. Gladstone Ports Corporation. Report for western basin dredging and disposal project. Sediment quality assessment. Brisbane, Australia: GHD Pty Ltd, 2009.
4. Queensland Department of Environment and Heritage Protection. Update on the quality of sediment from Port Curtis and Tributaries. 2012. ISSN 1834-3910.
5. Simpson SL, Batley GE, Chariton AA. Revision of the ANZECC/ARMCANZ Sediment Quality Guidelines. Sydney, Australia: CSIRO Land and Water, 2013. CSIRO Land and Water Science Report 08/07.
6. Angel BM, Jarolimek CV, King JJ, Hales LT, Simpson SL, Jung RF, et al. Metal Concentrations in the Waters and Sediments of Port Curtis, Queensland. Sydney, Australia: CSIRO Wealth from Oceans Flagship, 2012.
7. Kroon FJ, Berry KLE, Brinkman DL, Davis A, King O, Kookana R, et al. Identification, impacts, and prioritisation of emerging contaminants present in the GBR and Torres Strait marine environments. Final Report Project 1.10. Cairns, Australia: Report to the National Environmental Science Programme. Reef and Rainforest Research Centre Limited, 2015.
