# Supplementary material for: A protocol for identifying suitable biomarkers to assess fish health: A systematic review
Source: PLoS One. 2017 Apr 12;12(4):e0174762. doi: 10.1371/journal.pone.0174762 (PMC5389625; doi:10.1371/journal.pone.0174762)
Supplement: S6 Table — (DOCX) [file pone.0174762.s006.docx]

**S6 Table. Polycyclic aromatic hydrocarbon (PAHs) concentrations (µg kg^-1^) in Gladstone Harbour sediment based on publicly available data.**

| **Contaminant** | **Guideline value^*^** | | **Apte *et al.* 2005 [1]** | | | | **Vicente-Beckett *et al.* 2006 [2]** | | | | **GHD Pty Ltd 2009 [3]** | | | | **DEHP 2012 [4]** | | | |
| --- | --- | --- | --- | --- | --- | --- | --- | --- | --- | --- | --- | --- | --- | --- | --- | --- | --- | --- |
|  |  |  | **# of samples** | | **Concentration** | | **# of samples** | | **Concentration** | | **# of samples** | | **Concentration** | | **# of samples** | | **Concentration** | |
|  | **low** | **high** | **Tested** | **>LOR** | **Min** | **Max** | **Tested** | **>LOR** | **Min** | **Max** | **Tested** | **>LOR** | **Min** | **Max** | **Tested** | **>LOR** | **Min** | **Max** |
| 2-Methylnaphthalene |  |  |  |  |  |  |  |  |  |  | 1012 | 0 | nd | nd | 31 | 5 | 6 | 14 |
| Acenaphthene | 16 | 500 | 23 | 0 | nd | nd | 30 | 1 | 5 | 5 | 997 | 0 | nd | nd | 31 | 0 | nd | nd |
| Acenaphthylene | 44 | 640 | 23 | 0 | nd | nd | 30 | 0 | nd | nd | 997 | 1 | 4 | 4 | 31 | 0 | nd | nd |
| Anthracene | 85 | 1100 | 23 | 0 | nd | nd | 30 | 9 | 0.48 | 2 | 997 | 3 | 4 | 5 | 31 | 0 | nd | nd |
| Benz(a)anthracene | 261 | 1600 | 23 | 0 | nd | nd | 30 | 19 | 0.61 | 15 | 997 | 9 | 5 | 47 | 31 | 10 | 4 | 10 |
| Benzo(a)pyrene | 430 | 1600 | 23 | 0 | nd | nd | 30 | 17 | 2 | 15 | 997 | 13 | 4 | 42 | 31 | 8 | 5 | 10 |
| Benzo(e)pyrene |  |  |  |  |  |  | 30 | 19 | 0.48 | 19 | 1009 | 23 | 4 | 35 | 31 | 11 | 5 | 12 |
| Benzo(b)fluoranthene |  |  |  |  |  |  |  |  |  |  | 997 | 32 | 4 | 64 | 31 | 12 | 4 | 15 |
| Benzo(k)fluoranthene |  |  |  |  |  |  |  |  |  |  | 997 | 4 | 4 | 20 | 31 | 7 | 4 | 9 |
| Benzo(b,k)fluoranthene |  |  | 23 | 0 | nd | nd | 30 | 26 | 1 | 51 |  |  |  |  |  |  |  |  |
| Benzo(g,h,i)perylene |  |  | 23 | 0 | nd | nd | 30 | 21 | 0.71 | 13 | 997 | 19 | 4 | 21 | 31 | 6 | 5 | 10 |
| Chrysene | 384 | 2800 | 23 | 0 | nd | nd | 30 | 25 | 0.8 | 26 | 997 | 10 | 4 | 51 | 31 | 5 | 6 | 18 |
| Dibenzo(a,h)anthracene | 63 | 260 | 23 | 0 | nd | nd | 30 | 0 | nd | nd | 997 | 1 | 6 | 6 | 31 | 1 | 4 | 4 |
| Coronene |  |  |  |  |  |  |  |  |  |  | 1009 | 0 | nd | nd | 31 | 0 | nd | nd |
| Fluoranthene | 600 | 5100 | 23 | 0 | nd | nd | 30 | 26 | 1 | 42 | 997 | 19 | 4 | 55 | 31 | 14 | 4 | 17 |
| Fluorene | 19 | 540 | 23 | 0 | nd | nd | 30 | 13 | 0.61 | 8 | 997 | 0 | nd | nd | 31 | 0 | nd | nd |
| Indeno(1,2,3-c,d)pyrene |  |  | 23 | 0 | nd | nd | 30 | 7 | 0.61 | 2 | 997 | 10 | 4 | 18 | 31 | 1 | 4 | 4 |
| Naphthalene | 160 | 2100 | 23 | 0 | nd | nd | 30 | 19 | 2 | 7.8 | 997 | 3 | 6 | 7 | 31 | 23 | 5 | 15 |
| Perylene |  |  |  |  |  |  | 30 | 27 | 2 | 66.1 | 1009 | 246 | 4 | 1640 | 31 | 11 | 4 | 10 |
| Phenanthrene | 240 | 1500 | 23 | 0 | nd | nd | 30 | 23 | 2 | 30 | 997 | 5 | 8 | 31 | 31 | 13 | 6 | 34 |
| Pyrene | 665 | 2600 | 23 | 0 | nd | nd | 30 | 26 | 1 | 32 | 997 | 12 | 4 | 41 | 31 | 16 | 5 | 19 |
| Total PAHs | 10000^#^ | 50000^#^ |  |  | nd | nd | 22 | 22 | 5 | 266 | 997 | 263 | 4 | 1660 | 31 | 23 | 5 | 169 |

*ANZECC/ARMCANZ 2000 [5], except for ^#^ from Simpson et al. 2013 [6]; Abbreviations: LOR = limit of reporting; Min = minimum; Max = maximum; nd = not detected.

# References

1. Apte S, Duivenvoorden L, Johnson R, Jones MA, Revill A, Simpson S, et al. Contaminants in Port Curtis: screening level risk assessment. Indooroopilly, Australia: Cooperative Research Centre for Coastal Zone, Estuary and Waterway Management, 2005.
2. Vicente-Beckett V, Shearer D, Munksgaard N, Hancock G, Morrison H. Metal and polycyclic aromatic hydrocarbon contaminants in benthic sediments of Port Curtis. Indooroopilly, QLD: Cooperative Research Centre for coastal zone, estuary and waterway management., 2006 Technical report 73.
3. GHD Pty Ltd. Gladstone Ports Corporation. Report for western basin dredging and disposal project. Sediment quality assessment. Brisbane, Australia: GHD Pty Ltd, 2009.
4. Queensland Department of Environment and Heritage Protection. Update on the quality of sediment from Port Curtis and Tributaries. 2012. ISSN 1834-3910.
5. ANZECC/ARMCANZ (Australian and New Zealand Environment and Conservation Council and Agriculture and Resource Management Council of Australia and New Zealand). National Water Quality Management Strategy, Paper No. 4 - Australian and New Zealand Guidelines for Fresh and Marine Water Quality. Canberra, Australia: ANZECC/ARMCANZ, 2000.
6. Simpson SL, Batley GE, Chariton AA. Revision of the ANZECC/ARMCANZ Sediment Quality Guidelines. Sydney, Australia: CSIRO Land and Water, 2013. CSIRO Land and Water Science Report 08/07.
