# Supplementary material for: A protocol for identifying suitable biomarkers to assess fish health: A systematic review
Source: PLoS One. 2017 Apr 12;12(4):e0174762. doi: 10.1371/journal.pone.0174762 (PMC5389625; doi:10.1371/journal.pone.0174762)
Supplement: S7 Table — (DOCX) [file pone.0174762.s007.docx]

**S7 Table. Polychlorinated biphenyls (PCBs), chlorinated hydrocarbons and semi-volatile organic compounds concentrations (µg kg^-1^) in Gladstone Harbour sediment based on publicly available data.**

| **Contaminant group** | **Contaminant** | **Guideline value^*^** | | **GHD Pty Ltd 2009 [1]** | | | |
| --- | --- | --- | --- | --- | --- | --- | --- |
|  |  |  |  | **# of samples** | | **Concentration** | |
|  |  | **low** | **high** | **Tested** | **>LOR** | **Min** | **Max** |
| Polychlorinated biphenyls (PCBs) | Arochlor 1221 |  |  | 997 | 0 | nd | nd |
|  | Arochlor 1232 |  |  | 997 | 0 | nd | nd |
|  | Arochlor 1242 |  |  | 997 | 0 | nd | nd |
|  | Arochlor 1248 |  |  | 997 | 0 | nd | nd |
|  | Arochlor 1254 |  |  | 997 | 0 | nd | nd |
|  | Arochlor 1260 |  |  | 997 | 0 | nd | nd |
|  | Total PCBs | 34 | 280 | 997 | 0 | nd | nd |
| Chlorinated hydrocarbons | Hexachlorobenzene |  |  | 1029 | 0 | nd | nd |
| Semivolatile organic compounds (SVOC) | 2,4,6-trichlorophenol |  |  | 608 | 0 | nd | nd |
|  | 2,6-dichlorophenol |  |  | 608 | 0 | nd | nd |
|  | 2-nitrophenol |  |  | 608 | 0 | nd | nd |
|  | 4-chloro-3-methylphenol |  |  | 608 | 0 | nd | nd |
|  | Pentachlorophenol |  |  | 608 | 0 | nd | nd |

^*^Simpson et al. 2013 [2]; Abbreviations: LOR = limit of reporting; Min = minimum; Max = maximum; nd = not detected.

# References

1. GHD Pty Ltd. Gladstone Ports Corporation. Report for western basin dredging and disposal project. Sediment quality assessment. Brisbane, Australia: GHD Pty Ltd, 2009.
2. Simpson SL, Batley GE, Chariton AA. Revision of the ANZECC/ARMCANZ Sediment Quality Guidelines. Sydney, Australia: CSIRO Land and Water, 2013. CSIRO Land and Water Science Report 08/07.
