# Supplementary material for: A protocol for identifying suitable biomarkers to assess fish health: A systematic review
Source: PLoS One. 2017 Apr 12;12(4):e0174762. doi: 10.1371/journal.pone.0174762 (PMC5389625; doi:10.1371/journal.pone.0174762)
Supplement: S8 Table — (DOCX) [file pone.0174762.s008.docx]

**S8 Table. Total petroleum hydrocarbons and BTEX concentrations (mg kg^-1^) in Gladstone sediment based on publicly available data.**

| **Contaminant group** | **Contaminant** | **Guideline value^*^** | | **GHD Pty Ltd 2009 [1]** | | | | **DEHP 2012 [2]** | | | |
| --- | --- | --- | --- | --- | --- | --- | --- | --- | --- | --- | --- |
|  |  |  |  | **# of samples** | | **Concentration** | | **# of samples** | | **Concentration** | |
|  |  | **low** | **high** | **Tested** | **>LOR** | **Min** | **Max** | **Tested** | **>LOR** | **Min** | **Max** |
| Benzene, toluene, ethylbenzene and xylene (BTEX) | Benzene |  |  | 465 | 8 | 0.3 | 0.5 | 31 | 0 | nd | nd |
|  | Ethylbenzene |  |  | 465 | 8 | 0.3 | 0.5 | 31 | 0 | nd | nd |
|  | Toluene |  |  | 465 | 2 | 0.2 | 0.3 | 31 | 0 | nd | nd |
|  | Xylene (m & p) |  |  | 462 | 0 | nd | nd | 31 | 0 | nd | nd |
|  | Xylene (o) |  |  | 462 | 0 | nd | nd | 31 | 0 | nd | nd |
|  | Xylene total |  |  | 465 | 0 | nd | nd | 31 | 0 | nd | nd |
| Total petroleum hydrocarbons (TPH) | TPH C6 - C9 |  |  | 465 | 1 | 3 | 3 | 31 | 0 | nd | nd |
|  | TPH C10 - C14 |  |  | 465 | 1 | 6 | 6 | 31 | 1 | 4 | 4 |
|  | TPH C15 - C28 |  |  | 465 | 334 | 3 | 48 | 31 | 27 | 4 | 48 |
|  | TPH C29 - C36 |  |  | 465 | 307 | 5 | 42 | 31 | 20 | 6 | 37 |
|  | TPH + C10 - C36 | 280 | 550 | 465 | 349 | 7 | 73.5 | 31 | 27 | 4 | 89 |

^*^Simpson et al. 2013 [3]; Abbreviations: LOR = limit of reporting; Min = minimum; Max = maximum; nd = not detected.

# References

1. GHD Pty Ltd. Gladstone Ports Corporation. Report for western basin dredging and disposal project. Sediment quality assessment. Brisbane, Australia: GHD Pty Ltd, 2009.
2. Queensland Department of Environment and Heritage Protection. Update on the quality of sediment from Port Curtis and Tributaries. 2012. ISSN 1834-3910.
3. Simpson SL, Batley GE, Chariton AA. Revision of the ANZECC/ARMCANZ Sediment Quality Guidelines. Sydney, Australia: CSIRO Land and Water, 2013. CSIRO Land and Water Science Report 08/07.
