# Supplementary material for: A protocol for identifying suitable biomarkers to assess fish health: A systematic review
Source: PLoS One. 2017 Apr 12;12(4):e0174762. doi: 10.1371/journal.pone.0174762 (PMC5389625; doi:10.1371/journal.pone.0174762)
Supplement: S9 Table — (DOCX) [file pone.0174762.s009.docx]

**S9 Table. Organochlorine pesticide concentrations (µg kg^-1^) in Gladstone Harbour sediment based on publicly available data.**

| **Contaminant** | **Guideline value^*^** | | **GHD Pty Ltd 2009 [1]** | | | | **DEHP 2012 [2]** | | | |
| --- | --- | --- | --- | --- | --- | --- | --- | --- | --- | --- |
|  |  |  | **# of samples** | | **Concentration** | | **# of samples** | | **Concentration** | |
|  | **low** | **high** | **Tested** | **>LOR** | **Min** | **Max** | **Tested** | **>LOR** | **Min** | **Max** |
| 4,4-Dichlorodiphenyldichloroethylene (DDE) | 1.4 | 7 | 1029 | 0 | nd | nd | 31 | 0 | nd | nd |
| a-Hexachlorobenzene |  |  | 1029 | 0 | nd | nd | 31 | 0 | nd | nd |
| Aldrin |  |  | 1029 | 0 | nd | nd | 31 | 0 | nd | nd |
| b-Hexachlorobenzene |  |  | 1029 | 0 | nd | nd | 31 | 0 | nd | nd |
| Chlordane (cis) |  |  | 1029 | 0 | nd | nd | 31 | 0 | nd | nd |
| Chlordane (trans) |  |  | 1029 | 0 | nd | nd | 31 | 0 | nd | nd |
| d-Hexachlorobenzene |  |  | 1029 | 0 | nd | nd | 31 | 0 | nd | nd |
| Dichlorodiphenyldichloroethane (DDD) | 3.5 | 9 | 1029 | 0 | nd | nd | 31 | 0 | nd | nd |
| Dichlorodiphenyltrichloroethane (DDT) | 1.2 | 5 | 1029 | 0 | nd | nd | 31 | 0 | nd | nd |
| DDT + DDE + DDD |  |  | 1029 | 0 | nd | nd | 31 | 0 | nd | nd |
| Dieldrin | 2.8 | 7 | 1029 | 0 | nd | nd | 31 | 0 | nd | nd |
| Endosulfan I |  |  | 1029 | 0 | nd | nd | 317 | 0 | nd | nd |
| Endosulfan II |  |  | 1029 | 0 | nd | nd | 31 | 0 | nd | nd |
| Endosulfan sulphate |  |  | 1029 | 0 | nd | nd | 31 | 0 | nd | nd |
| Endrin | 2.7 | 60 | 1029 | 0 | nd | nd | 31 | 0 | nd | nd |
| Endrin aldehyde |  |  | 1029 | 0 | nd | nd | 31 | 0 | nd | nd |
| Endrin ketone |  |  | 1029 | 0 | nd | nd | 31 | 0 | nd | nd |
| Lindane | 0.9 | 1.4 | 1029 | 0 | nd | nd | 31 | 0 | nd | nd |
| Heptachlor |  |  | 1029 | 0 | nd | nd | 31 | 0 | nd | nd |
| Heptachlor epoxide |  |  | 1029 | 0 | nd | nd | 31 | 0 | nd | nd |
| Methoxychlor |  |  | 1029 | 0 | nd | nd | 31 | 0 | nd | nd |

* Simpson et al. [3]; Abbreviations: LOR = limit of reporting; Min = minimum; Max = maximum; nd = not detected.

# References

1. GHD Pty Ltd. Gladstone Ports Corporation. Report for western basin dredging and disposal project. Sediment quality assessment. Brisbane, Australia: GHD Pty Ltd, 2009.
2. Queensland Department of Environment and Heritage Protection. Update on the quality of sediment from Port Curtis and Tributaries. 2012. ISSN 1834-3910.
3. Simpson SL, Batley GE, Chariton AA. Revision of the ANZECC/ARMCANZ Sediment Quality Guidelines. Sydney, Australia: CSIRO Land and Water, 2013. CSIRO Land and Water Science Report 08/07.
