# Supplementary material for: A protocol for identifying suitable biomarkers to assess fish health: A systematic review
Source: PLoS One. 2017 Apr 12;12(4):e0174762. doi: 10.1371/journal.pone.0174762 (PMC5389625; doi:10.1371/journal.pone.0174762)
Supplement: S10 Table — (DOCX) [file pone.0174762.s010.docx]

**S10 Table. Organophosphorus pesticide concentrations (µg kg^-1^) in Gladstone Harbour sediment based on publicly available data.**

| **Contaminant** | **Guideline value^*^** | | **GHD Pty Ltd 2009 [1]** | | | | **DEHP 2012 [2]** | | | |
| --- | --- | --- | --- | --- | --- | --- | --- | --- | --- | --- |
|  |  |  | **# of samples** | | **Concentration** | | **# of samples** | | **Concentration** | |
|  | **low** | **high** | **Tested** | **>LOR** | **Min** | **Max** | **Tested** | **>LOR** | **Min** | **Max** |
| Azinophos methyl |  |  | 1009 | 0 | nd | nd | 31 | 0 | nd | nd |
| Bromophos |  |  | 1009 | 0 | nd | nd | 31 | 0 | nd | nd |
| Carbophenothion |  |  | 1009 | 0 | nd | nd | 31 | 0 | nd | nd |
| Chlorfenvinphos |  |  | 1009 | 0 | nd | nd | 31 | 0 | nd | nd |
| Chlorfenvinphos Z |  |  | 1009 | 0 | nd | nd | 31 | 0 | nd | nd |
| Chlorphyrifos |  |  | 1009 | 0 | nd | nd | 31 | 0 | nd | nd |
| Chlorphyrifos-methyl |  |  | 1009 | 0 | nd | nd | 31 | 0 | nd | nd |
| Demeton-S-methyl |  |  | 1009 | 0 | nd | nd | 31 | 0 | nd | nd |
| Diazinon |  |  | 1009 | 0 | nd | nd | 31 | 0 | nd | nd |
| Dichlorvos |  |  | 1009 | 0 | nd | nd | 31 | 0 | nd | nd |
| Dimethoate |  |  | 1009 | 0 | nd | nd | 31 | 0 | nd | nd |
| Ethion |  |  | 1009 | 0 | nd | nd | 31 | 0 | nd | nd |
| Fenamiphos |  |  | 1009 | 0 | nd | nd | 31 | 0 | nd | nd |
| Fenthion |  |  | 1009 | 0 | nd | nd | 31 | 0 | nd | nd |
| Malathion |  |  | 1009 | 0 | nd | nd | 31 | 0 | nd | nd |
| Methyl parathion |  |  | 1009 | 0 | nd | nd | 31 | 0 | nd | nd |
| Monocrotophos |  |  | 1009 | 0 | nd | nd | 31 | 0 | nd | nd |
| Parathion |  |  | 1009 | 0 | nd | nd | 31 | 0 | nd | nd |
| Pirimphos-ethyl |  |  | 1009 | 0 | nd | nd | 31 | 0 | nd | nd |
| Prothiofos |  |  | 1009 | 0 | nd | nd | 31 | 0 | nd | nd |

Abbreviations: LOR = limit of reporting; Min = minimum; Max = maximum; nd = not detected.

# References

1. GHD Pty Ltd. Gladstone Ports Corporation. Report for western basin dredging and disposal project. Sediment quality assessment. Brisbane, Australia: GHD Pty Ltd, 2009.
2. Queensland Department of Environment and Heritage Protection. Update on the quality of sediment from Port Curtis and Tributaries. 2012. ISSN 1834-3910.
