# Supplementary material for: A protocol for identifying suitable biomarkers to assess fish health: A systematic review
Source: PLoS One. 2017 Apr 12;12(4):e0174762. doi: 10.1371/journal.pone.0174762 (PMC5389625; doi:10.1371/journal.pone.0174762)
Supplement: S11 Table — (DOCX) [file pone.0174762.s011.docx]

**S11 Table. Herbicides, carbamate pesticides and insecticides concentrations (µg kg^-1^) in Gladstone Harbour sediment based on publicly available data.**

| **Contaminant group** | **Contaminant** | **GHD Pty Ltd 2009 [1]** | | | |
| --- | --- | --- | --- | --- | --- |
|  |  | **# of samples** | | **Concentration** | |
|  |  | **Tested** | **>LOR** | **Min** | **Max** |
| Carbamate | 3-Hydrox Carbofuran | 30 | 0 | nd | nd |
|  | Aldicarb | 30 | 0 | nd | nd |
|  | Bendiocarb | 30 | 0 | nd | nd |
|  | Carbaryl | 30 | 0 | nd | nd |
|  | Carbofuran | 30 | 0 | nd | nd |
|  | Methiocarb | 30 | 0 | nd | nd |
|  | Methomyl | 30 | 0 | nd | nd |
|  | Oxamyl | 30 | 0 | nd | nd |
|  | Thiodicarb | 30 | 0 | nd | nd |
| Herbicides | Atrazine | 30 | 0 | nd | nd |
|  | Pronamide | 4 | 0 | nd | nd |
|  | Simazine | 30 | 0 | nd | nd |
|  | 2,4,5-Trichlorophenoxyacetic acid | 30 | 0 | nd | nd |
|  | 2,4,5-Trichlorophenoxypropionic acid (fenoprop, silvex) | 30 | 0 | nd | nd |
|  | 2,4-Dichlorophenoxyacetic acid | 30 | 0 | nd | nd |
|  | 2,4-Dichlorophenoxybutyric acid | 30 | 0 | nd | nd |
|  | 4-Chlorophenoxy acetic acid | 30 | 0 | nd | nd |
|  | Clopyralid | 30 | 0 | nd | nd |
|  | Dicamba | 30 | 0 | nd | nd |
|  | Fluroxypyr | 30 | 0 | nd | nd |
|  | 2-methyl-4-chlorophenoxyacetic acid | 30 | 0 | nd | nd |
|  | 4-chloro-2-methylphenoxybutanoic acid | 30 | 0 | nd | nd |
|  | Mecoprop | 30 | 0 | nd | nd |
|  | Picioram | 30 | 0 | nd | nd |
|  | Triclopyr | 30 | 0 | nd | nd |

Abbreviations: LOR = limit of reporting; Min = minimum; Max = maximum; nd = not detected.

# References

1. GHD Pty Ltd. Gladstone Ports Corporation. Report for western basin dredging and disposal project. Sediment quality assessment. Brisbane, Australia: GHD Pty Ltd, 2009.
