# Supplementary material for: A protocol for identifying suitable biomarkers to assess fish health: A systematic review
Source: PLoS One. 2017 Apr 12;12(4):e0174762. doi: 10.1371/journal.pone.0174762 (PMC5389625; doi:10.1371/journal.pone.0174762)
Supplement: S12 Table — (DOCX) [file pone.0174762.s012.docx]

**S12 Table. Fish species reported in and around Gladstone harbour from 2005 to 2015.** Data were sourced from both fisheries dependent [1-5] and independent [6-10] studies, as well as from the Queensland Government shark control program [10], published from 2005 onwards.

| **Type of Study** | | **Fisheries Dependent** | | **Fisheries Independent** | | | | |
| --- | --- | --- | --- | --- | --- | --- | --- | --- |
| **References** | | **Commercial** [3, 5] | **Recreational** [1, 2] | [7] | [8] | [9] | [6] | [10] |
| **Scientific Name** | **Common Name** | **2005 / 2015** | **2006 / 2014** | **2003** | **??** | **2012** | **2014** | **2006 / 2015** |
| N/A | Australian Blacktip |  |  |  |  |  |  | 9 |
| N/A | Blacktip Reef Whaler |  |  |  |  |  |  | 360 |
| N/A | Bull Whaler |  |  |  |  |  |  | 42 |
| N/A | Common Blacktip Whaler |  |  |  |  |  |  | 62 |
| N/A | Creek Whaler |  |  |  |  |  |  | 1 |
| N/A | Dusky Whaler |  |  |  |  |  |  | 0 |
| N/A | Hammerhead Shark |  |  |  |  |  |  | 1 |
| N/A | Long Nose Whaler |  |  |  |  |  |  | 1 |
| N/A | Sandbar Whaler |  |  |  |  |  |  | 10 |
| N/A | Scalloped Hammerhead |  |  |  |  |  |  | 5 |
| N/A | Sharptooth Shark |  |  |  |  |  |  | 1 |
| N/A | Tawny Shark |  |  |  |  |  |  | 1 |
| N/A | Tiger Shark |  |  |  |  |  |  | 113 |
| N/A | Whaler |  |  |  |  |  |  | 1 |
| *Absalom radiatus* | Fringe-Finned Trevally |  |  | 2 |  |  |  |  |
| *Acanthopagrus australis* | Yellowfin Bream |  | x | 47 | x | 3 | 58 |  |
| *Acanthopagrus berda* | Pikey Bream |  | x |  | x | 1 | 32 |  |
| N/A | Bream | 18.1 | x |  |  |  |  |  |
| *Acanthurus fuliginosus* | Surgeonfish |  |  |  | x |  |  |  |
| *Acentronura tentaculata* | Shortpouch Pygmy Pipehorse |  |  |  | x |  |  |  |
| *Achlyopa nigra* | Black Sole |  |  | 1 |  |  |  |  |
| *Alepes kleini* | Razor Belly Scad |  |  |  | x |  |  |  |
| *Ambassis marianus* | Macleay's Glassfish |  |  | 244 | x | 1 | 266 |  |
| *Ambiserrula jugosa* | Flat Head |  |  |  | x |  |  |  |
| *Amniataba percoides* | Barred Grunter |  |  |  | x | 3 | 12 |  |
| *Anacanthus barbatus* | Bearded Leather Jack |  |  |  | x |  |  |  |
| *Anyperodon leucogrammicus* | Grouper |  |  |  | x |  |  |  |
| *Apogon fasciatus* | Striped Cardinalfish |  |  | 30 |  |  |  |  |
| *Apogon limenus* | Cardinal Fish |  |  |  | x |  |  |  |
| *Argyrosomus japonicus* | Jewfish |  |  | 2 |  |  |  |  |
| *Arius graeffei* | Blue Catfish |  |  |  | x | 5 |  |  |
| *Arothron hispidus* | Puffer Fish |  |  |  | x |  |  |  |
| *Arothron manilensis* | Narrow-Lined Toadfish |  |  | 6 |  |  |  |  |
| *Arrhamphus sclerolepis* | Snub-Nosed Garfish |  |  | 24 |  | 1 | 3 |  |
| *Aseraggodes normani* | Flat Fish |  |  |  | x |  |  |  |
| *Atherinomorus ogilbyi* | Common Hardyhead |  |  | 97 |  |  |  |  |
| *Auxis thazard* | Frigate Mackerel |  |  | 1 |  |  |  |  |
| *Bathyaploactis ornatissimus* | Velvet Fish |  |  |  | x |  |  |  |
| *Bathygobius fuscus* | Dusky Frillgoby |  |  |  | x |  |  |  |
| *Campichthys tryoni* | Tryon’s Pipefish |  |  |  | x |  |  |  |
| *Carangoides caeruleopinnatus* | Onion-Ring Trevally |  |  | 2 |  |  |  |  |
| *Carangoides fulvoguttatus* | Yellow Spotted Trevally |  |  |  | x |  |  |  |
| *Caranx ignobilis* | Giant Trevally |  |  |  | x |  |  |  |
| *Caranx melampygus* | Blue Fin Trevally |  |  |  | x |  |  |  |
| *Caranx sexfasciatus* | Big Eye Trevally |  |  |  | x |  |  |  |
| *Centropogon marmoratus* | Cobbler/Marbled Fortescue |  |  |  | x |  |  |  |
| *Chaetodon tricinctus* | Three-Band Coralfish |  |  | 2 |  |  |  |  |
| *Chanos chanos* | Milkfish |  |  |  |  |  | 2 |  |
| *Chelonodon patoca* | Milk-Spotted Puffer |  |  |  |  | 1 |  |  |
| *Choerodon cephalotes* | Purple Tuskfish |  |  | 1 |  |  |  |  |
| *Choeroichthys brachysoma* | Pacific Short-Bodied Pipefish |  |  |  | x |  |  |  |
| *Corythoichthys amplexus* | Fijian Banded Pipefish |  |  |  | x |  |  |  |
| *Corythoichthys flavofasciatu* | Reticulate Pipefish |  |  |  | x |  |  |  |
| *Corythoichthys haematopterus* | Reef-Top Pipefish |  |  |  | x |  |  |  |
| *Corythoichthys intestinalis* | Australian Messmate Pipefish |  |  |  | x |  |  |  |
| *Corythoichthys ocellatus* | Orange-Spotted Pipefish |  |  |  | x |  |  |  |
| *Corythoichthys paxtoni* | Paxton’s Pipefish |  |  |  | x |  |  |  |
| *Corythoichthys schultzi* | Schultz’s Pipefish |  |  |  | x |  |  |  |
| *Craterocephalus stercusmuscarum* | Flyspecked Hardyhead |  |  |  | x |  |  |  |
| *Cynoglossus maccullochi* | Mcculloch's Tongue Sole |  |  |  | x |  |  |  |
| *Dasyatis fluviorum* | Brown Stingray |  |  | 2 |  |  |  |  |
| *Diodor nichthemerus* | Porcupine Fish |  |  | 1 |  |  |  |  |
| *Doryrhamphus excisus* | Bluestripe Pipefish |  |  |  | x |  |  |  |
| *Drepane punctata* | Spotted Sickelfish |  |  | 12 | x |  |  |  |
| *Eleutheronema tetradactylum* | Blue Threadfin |  | x | 1 | x | 1 |  |  |
| N/A | Threadfin | 212.0 |  |  |  |  |  |  |
| *Elops hawaiensis* | Ladyfish Or Herring |  |  |  | x |  |  |  |
| *Engraulis australis* | Anchovy- Australian |  |  |  |  |  | 3 |  |
| *Epinephelus coioides* | Goldspotted Rockcod |  | x | 1 | x |  |  |  |
| *Epinephelus maculatus* | High Fin Grouper Or Rock Cod |  |  |  | x |  |  |  |
| *Epinephelus malabaricus* | Malabar Or Greasy Grouper |  |  |  | x |  |  |  |
| *Epinephelus quoyanus* | Longfin Rockcod |  | x |  |  |  |  |  |
| *Erosa erosa* | Pacific Monkey Fish |  |  |  | x |  |  |  |
| *Favonigobius exquisitus* | Sand Goby |  |  |  | x |  |  |  |
| *Festucalex cinctus* | Girdled Pipefish |  |  |  | x |  |  |  |
| *Filicampus tigris* | Tiger Pipefish |  |  |  | x |  |  |  |
| *Fraudella carassiops* | Carp Prettyfin |  |  |  | x |  |  |  |
| *Gerres filamentosus* | Threadfin Silverbelly |  |  | 23 |  |  | 3 |  |
| *Gerres oyena* | Oceanic Silverbelly |  |  | 20 | x |  |  |  |
| *Gerres subfasciatus* | Common Silverbelly |  |  | 65 | x |  | 32 |  |
| *Glossamia aprion* | Mouth Almighty |  |  |  | x |  | 1 |  |
| *Gnathanodon speciosus* | Golden Trevally |  |  |  | x |  |  |  |
| *Gymnothorax favagineus* | Laced Moray |  |  |  | x |  |  |  |
| *Halicampus dunckeri* | Red-Hair Pipefish |  |  |  | x |  |  |  |
| *Halicampus grayi* | Mud Pipefish |  |  |  | x |  |  |  |
| *Halicampus nitidus* | Glittering Pipefish |  |  |  | x |  |  |  |
| *Halicampus spinirostris* | Spiny-Snout Pipefish |  |  |  | x |  |  |  |
| *Harpadon translucens* | Lizard Fish |  |  |  | x |  |  |  |
| *Herklotsichthys castelnaui* | Southern Herring |  |  | 489 | x |  | 96 |  |
| *Hippichthys cyanospilos* | Blue-Speckled Pipefish |  |  |  | x |  |  |  |
| *Hippichthys heptagonus* | Madura Pipefish |  |  |  | x |  |  |  |
| *Hippichthys penicillus* | Beady Pipefish |  |  |  | x |  |  |  |
| *Hippocampus bargibanti* | Pygmy Seahorse |  |  |  | x |  |  |  |
| *Hippocampus hendriki* | Eastern Spiny Seahorse |  |  |  | x |  |  |  |
| *Hippocampus kuda* | Spotted Seahorse |  |  |  | x |  |  |  |
| *Hippocampus multispinus* | Northern Spiny Seahorse |  |  |  | x |  |  |  |
| *Hippocampus planifrons* | Flat-Faced Seahorse |  |  |  | x |  |  |  |
| *Hippocampus zebra* | Zebra Seahorse |  |  |  | x |  |  |  |
| *Hyperolophus translucidus* | Glassy Sprat |  |  | 2 |  |  |  |  |
| *Hyporhamphus australis* | Sea Garfish |  |  | 1 |  |  |  |  |
| *Hyporhamphus quoyi* | Short-Nosed Garfish |  |  | 6 | x |  |  |  |
| *Hypseleotris compressa* | Empire Gudgeon |  |  |  | x |  |  |  |
| *Jaydia argyrogaster* | Cardinal Or Siphon Fish |  |  |  | x |  |  |  |
| *Johnius borneensis* | Croaker/River Dew Fish |  |  |  | x |  |  |  |
| *Kuhlia rupestris* | Jungle Perch |  |  |  | x |  |  |  |
| *Kyphosus bigibbus* | Brown Chub |  |  |  | x |  |  |  |
| *Lates calcarifer* | Barramundi | 415.6 | x |  |  |  | 6 |  |
| *Leiognathus equulus* | Common Ponyfish |  |  | 1030 | x | 10 | 195 |  |
| *Leiopotherapon unicolor* | Spangled Perch |  |  |  | x |  | 4 |  |
| *Leptobrama muelleri* | Salmon |  |  | 30 | x | 4 |  |  |
| *Lethrinus genivittatus* | Threadfin Emperor |  |  |  | x |  |  |  |
| *Lethrinus laticaudis* | Grass Emperor |  | x |  | x |  |  |  |
| *Lethrinus nebulosus* | Spangled Emperor |  |  |  | x |  |  |  |
| *Liocranium praepositum* | Waspfish |  |  |  | x |  |  |  |
| *Lissocampus runa* | Javelin Pipefish |  |  |  | x |  |  |  |
| *Liza argentea* | Goldspot Mullet |  |  |  |  |  | 454 |  |
| *Liza dussumieri* | Flat-Tail Mullet |  |  | 27 |  |  |  |  |
| *Liza subviridis* | Greenback Mullet |  |  |  | x | 51 |  |  |
| *Liza tade* | Tade Mullet |  |  |  | x |  |  |  |
| *Liza vaigiensis* | Diamond Scale Mullet |  |  |  | x | 2 |  |  |
| *Lubricogobius ornatus* | Ornate Slippery Goby |  |  |  | x |  |  |  |
| *Lutjanus argentimaculatus* | Mangrove Jack |  | x |  |  |  | 1 |  |
| *Lutjanus carponotatus* | Stripey Snapper |  | x |  |  |  |  |  |
| *Lutjanus gibbus* | Humpback Red Snapper |  |  |  | x |  |  |  |
| *Lutjanus russellii* | Russell's Snapper |  |  |  | x |  | 5 |  |
| *Marilyna pleurosticta* | Banded Toadfish |  |  | 8 |  |  |  |  |
| *Megalaspis cordyla* | Finny Scad |  |  | 121 |  |  |  |  |
| *Megalops cyprinoides* | Indo-Pacific Tarpon Or Herring |  |  |  | x |  |  |  |
| *Meiacanthus luteus* | Yellow Fangbelly |  |  |  | x |  |  |  |
| *Melanotoenia splendida* | Eastern Rainbow Fish |  |  |  |  |  | 2 |  |
| *Meuschenia sp2* | Leatherjacket |  |  | 42 |  |  |  |  |
| *Microcanthus strigatus* | Stripey |  |  |  | x |  |  |  |
| *Micrognathus andersonii* | Anderson’s Pipefish |  |  |  | x |  |  |  |
| *Micrognathus brevirostris* | Thorntail Pipefish |  |  |  | x |  |  |  |
| *Monodactylus argenteus* | Diamond Fish |  |  |  |  |  | 1 |  |
| *Mugil cephalus* | Sea Mullet |  |  |  | x |  | 243 |  |
| N/A | Mullet | 147.6 |  |  |  |  |  |  |
| *Mugilogobius stigmaticus* | Blackspot Mangrove Goby |  |  |  | x |  |  |  |
| *Muraenesox cinevus* | Pike Eel |  |  | 1 |  |  |  |  |
| *Nannocampus pictus* | Painted Pipefish |  |  |  | x |  |  |  |
| *Naso annulatus* | Whitemargin Unicornfish |  |  |  | x |  |  |  |
| *Naso tonganus* | Bulbnose Unicornfish |  |  |  | x |  |  |  |
| *Naso unicornis* | Bluespine Unicornfish |  |  |  | x |  |  |  |
| *Nematalosa come* | Bony Bream |  |  | 18 |  |  |  |  |
| *Nematalosa erebi* | Bony Bream |  |  |  |  |  | 83 |  |
| *Nemipterus theodorei* | Yellow-Lip Butterfly-Bream |  |  | 2 |  |  |  |  |
| *Notesthes robusta* | Bull Rout |  |  |  | x |  | 1 |  |
| *Omobranchus rotundiceps* | Rotund Blenny |  |  |  | x |  |  |  |
| *Ophiocara porocephala* | Spangled Gudgeon |  |  |  | x |  |  |  |
| *Opistognathus eximius* | Harlequin Smiler |  |  |  | x |  |  |  |
| *Orbonymus rameus* | White Spotted Dragonet |  |  |  | x |  |  |  |
| *Otolithes ruber* | Tiger Tooth Croaker |  |  |  | x |  |  |  |
| *Paracentropogon vespa* | Wasp Roguefish |  |  |  | x |  |  |  |
| *Parachaetodon ocellatus* | Ocellate Coralfish |  |  | 2 |  |  |  |  |
| *Paradicula setifer* | Sole |  |  |  | x |  |  |  |
| *Paramugil georgii* | Goldspot Mullet |  |  |  | x |  |  |  |
| *Paraploactis trachyderma* | Mosback Velvetfish |  |  |  | x |  |  |  |
| *Parapriacanthus ransonneti* | Pigmy Sweeper |  |  |  | x |  |  |  |
| *Parascorpaena mossambica* | Mozambique Scorpionfish |  |  |  | x |  |  |  |
| *Pelates quadrilineatus* | Trumpeter |  |  | 53 |  |  |  |  |
| *Pelates sexlineatus* | Eastern Striped Trumpeter |  |  | 17 |  |  |  |  |
| *Pentapodus paradiseus* | Paradise Threadfin Bream |  |  | 1 | x |  |  |  |
| *Periopthalmus koelreuteri* | Mud-Skipper |  |  | 1 |  |  |  |  |
| *Petroscirtes variabilis* | Sabre Tooth Blenny |  |  |  | x |  |  |  |
| *Platycephalus arenarius* | Sand Flathead |  |  | 1 |  |  |  |  |
| *Platycephalus fuscus* | Dusky Flathead |  | x |  |  |  | 4 |  |
| *Platycephalus indicus* | Bartail Flathead |  |  |  |  |  |  |  |
| N/A | Flathead | 11.3 | x |  |  |  |  |  |
| *Plectorhinchus gibbosus* | Brown Sweetlips |  |  |  |  |  | 1 |  |
| *Polydactylus multiradiatus* | Australian Threadfin |  |  |  | x |  |  |  |
| *Pomacanthus semicirculatus* | Blue Angel Fish |  |  |  | x |  |  |  |
| *Pomadasys argenteus* | Speckled Javelin |  |  |  |  |  | 1 |  |
| *Pomadasys kaakan* | Barred Javelin |  | x | 77 | x |  | 1 |  |
| *Pomadasys maculatus* | Saddle Grunt |  |  |  | x |  |  |  |
| N/A | Grunter | 24.1 |  |  |  |  |  |  |
| *Priacanthus macracanthus* | Red Big Eye |  |  |  | x |  |  |  |
| *Pseudomonacanthus elongatus* | Fourband Leatherjack / File Fish |  |  |  | x |  |  |  |
| *Pseudomugil signifer* | Pacific Blue Eye |  |  |  | x |  |  |  |
| *Pseudorhombus argent* | Flounder |  |  | 1 |  |  |  |  |
| *Pseudorhombus arsius* | Large-Toothed Flounder |  |  | 23 | x |  |  |  |
| *Pteragogus flagellifera* | Cocktail Fish |  |  |  | x |  |  |  |
| *Sardinella gibbosa* | Goldstripe Sardinella |  |  |  | x |  |  |  |
| *Saurida undosquamis* | Large-Scaled Grinner |  |  | 65 |  |  |  |  |
| *Scatophagus argus* | Spotted Scat |  |  |  |  |  | 54 |  |
| *Scolopsis monogramma* | Rainbow Monocle Bream |  |  |  | x |  |  |  |
| *Scomberoides commersonnianus* | Talang Queenfish |  |  | 10 | x | 32 | 2 |  |
| *Scomberoides lysan* | Doublespotted Queenfish |  |  |  | x |  |  |  |
| *Scomberomorus queenslandicus* | School Mackerel |  | x | 1 |  |  |  |  |
| N/A | Mackerel | 319.8 |  |  |  |  |  |  |
| *Selaroides leptolepis* | Smooth-Tailed Trevally |  |  | 15 |  |  |  |  |
| *Selenotoca multifasciata* | Striped Butterfish |  |  | 3 |  |  | 72 |  |
| *Seriola lalandi* | Yellowtail Kingfish |  |  |  | x |  |  |  |
| *Siganus lineatus* | Gold Lined Rabbitfish |  |  |  |  |  | 5 |  |
| *Siganus rivulatus* | Happy Moments |  |  | 83 |  |  |  |  |
| *Sillago analis* | Whiting |  |  | 3 | x |  |  |  |
| *Sillago ciliata* | Sand Whiting |  |  |  | x | 2 | 30 |  |
| *Sillago maculata maculata* | Winter Whiting |  |  | 15 |  |  |  |  |
| N/A | Whiting | 20.2 |  |  |  |  |  |  |
| *Solegnathus hardwickii* | Pallid Pipehorse |  |  |  | x |  |  |  |
| *Soleichthys heterorhinos* | Tiger Sole |  |  |  | x |  |  |  |
| *Solenostomus cyanopterus* | Robust Ghostpipefish |  |  |  | x |  |  |  |
| *Solenostomus paradoxus* | Ornate Ghostpipefish |  |  |  | x |  |  |  |
| *Sphyraena jello* | Pick Handle Barracuda |  |  |  | x |  |  |  |
| *Sphyraena obtusata* | Yellowtail Barracuda |  |  |  | x |  |  |  |
| *Strongylura strongylura* | Spottail Needlefish |  |  |  | x |  |  |  |
| *Synanceia horrida* | Estuarine Stonefish |  |  |  | x |  |  |  |
| *Syngnathoides biaculeatus* | Double-End Pipehorse |  |  |  | x |  |  |  |
| *Synodus lobeli* | Lobel's Lizardfish |  |  |  | x |  |  |  |
| *Tathicarpus butleri* | Blackspot Angerlfish |  |  |  | x |  |  |  |
| *Terapon jarbua* | Crescent Grunter |  |  |  |  |  | 11 |  |
| *Terapon puta* | Spinycheek Grunter |  |  |  | x |  |  |  |
| *Tetractenos hamiltoni* | Common Toadfish |  |  | 9 |  | 4 | 7 |  |
| *Tetradontidae* | Puffer Fish |  |  |  |  |  |  |  |
| *Thryssa aestuaria* | Southern Anchovy |  |  | 10 |  | 1 |  |  |
| *Thunnus albacares* | Yellowfin Tuna |  |  |  | x |  |  |  |
| *Thunnus tonggol* | Longtail Tuna |  |  |  | x |  |  |  |
| *Trachinocephalus myops* | Painted Grinner |  |  |  | x |  |  |  |
| *Trachyrhamphus bicoarctatus* | Bentstick Pipefish |  |  |  | x |  |  |  |
| *Trepaon jarbua* | Crescent Perch |  |  | 14 |  |  |  |  |
| *Triacanthus brevirostris* | Short-Nosed Tripod-Fish |  |  | 7 |  |  |  |  |
| *Tripodichthys angustifrons* | Yellow-Fin Tripod-Fish |  |  | 26 |  |  |  |  |
| *Tylosurus crocodilus* | Crocodile Long-Tom |  |  | 2 |  |  |  |  |
| *Tylosurus gavialoides* | Stout Longtom |  |  |  |  |  | 2 |  |
| *Ulua mentalis* | Cale Trevally |  |  | 1 |  |  |  |  |
| *Upeneus australiae* | Australian Goatfish |  |  |  | x |  |  |  |
| *Upeneus tragula* | Bar-Tailed Goatfish |  |  | 7 |  |  |  |  |
| *Valamugil cunnesius* | Longarm Mullet |  |  |  | x |  |  |  |
| *Valamugil georgii* | Fantail Mullet |  |  | 80 |  |  |  |  |
| *Zenarchopterus buffonis* | Buffon's River Garfish |  |  |  | x |  |  |  |

# References

1. Sawynok B, Platten JR, Parsons W, Sawynok S. Gladfish 2012. Assessing trends in recreational fishing in Gladstone Harbour and adjacent waterways. Frenchville, Australia: Infofish Australia, 2012.
2. Sawynok B, Platten JR, Parsons W, Sawynok S. Gladfish 2014. Assessing trends in recreational fishing in Gladstone Harbour and adjacent waterways. Frenchville, Australia: Infofish Australia, 2014.
3. Department of Agriculture Fisheries and Forestry. Queensland Fishing (QFish) Brisbane, Australia: Queensland Government; 2016 [cited 2016 April]. Available from: <http://qfish.fisheries.qld.gov.au/>
4. Department of Agriculture Fisheries and Forestry. Commercial Catch of Key Species. Gladstone. 2006-2011. Brisbane, Australia: Fisheries Queensland, 2012.
5. Queensland Government. Commercial fishing logbook maps of Queensland Brisbane, Australia: Queensland Government; 2016 [cited 2016 April]. Available from: <https://www.business.qld.gov.au/industry/fisheries/commercial-fishing/monitoring-and-reporting/reporting-commercial-fishers/queensland-logbook-maps>
6. Sawynok B, Parsons W, Sawynok S. Calliope River Fish Recruitment. Frenchville, Australia: Infofish Australia, 2015.
7. Connolly RM, Currie DR, Danaher KF, Dunning M, Melzer A, Platten JR, et al. Intertidal wetlands of Port Curtis: Ecological patterns and processes and their implications. CRC for Coastal Zone, Estuary and Waterway Management, 2006.
8. Gladstone Port Corporation. Curtis Coast Coastal and Marine Resource Inventory Report. Gladstone, Australia: Gladstone Ports Corporation, 2012.
9. Wilson S, Anastasi A, Hansler M, Stoneham B, Payne M, Charlesworth D. Arrow LNG Plant Supplementary Report to the EIS. Part B: Marine & Estuarine Ecology Report. Gladstone, Australia: Central Queensland University, 2012.
10. Department of Agriculture and Fisheries. Shark catch numbers Brisbane, Australia: Queensland Government; 2016 [cited 2016 April]. Available from: <https://www.daf.qld.gov.au/fisheries/services/shark-control-program/catch-numbers>
