# Supplementary material for: A protocol for identifying suitable biomarkers to assess fish health: A systematic review
Source: PLoS One. 2017 Apr 12;12(4):e0174762. doi: 10.1371/journal.pone.0174762 (PMC5389625; doi:10.1371/journal.pone.0174762)
Supplement: S13 Table — (DOCX) [file pone.0174762.s013.docx]

**S13 Table. Bioaccumulation studies conducted in the (a) field and (b) laboratory for contaminants of concern on coastal and marine fish.** For contaminants measured in fish, only those contaminants that were identified as of concern to Gladstone Harbour (Al, Cd, Cu, Ga, Pb, Se, Zn) are presented; most studies measured additional contaminants in the environment which are also presented.

(a)

| **Species** | **Contaminants measured** | | **Tissue** | | | | | **Method** | **Exposure pathway** | | | **Life history stage** | | | **Reference** |
| --- | --- | --- | --- | --- | --- | --- | --- | --- | --- | --- | --- | --- | --- | --- | --- |
|  | **Fish** | **Environment** | **G** | **L** | **M** | **Wf** | **O** |  | **W** | **S** | **F** | **J** | **A** | **U** |  |
| *Acanthogobius hasta, Chaeturichthys sitgmatias, Cyprinus carpio* Linnaeus*, Hypophthalmichthys molitrix, Lateolabras japonicus, Liza haematocheila* | Cd, Cu, Pb, Zn | As, Cr, Hg, Mn, Ni |  |  |  |  |  | ICP, AFS | x | x |  |  |  | x | [1] |
| *Acanthopagrus australis, Arrhamphus sclerolepis, Mugil cephalus* | Cu, Pb, Zn | As, Cr, Co, Fe, Mn, Ni | x | x | x |  |  | ICP-MS | x | x |  |  |  | x | [2] |
| *Acanthopagrus australis, Mugil cephalus, Platycephalus fuscus* | Se | - |  |  | x |  |  | AAS |  | x |  |  |  |  | [3] |
| *Acanthopagrus berda, Elops machnata, Gerres abbreviatus, G. filamentosus, Leiognathus equulus, Liza affinis, L. macrolepis, L. subviridis, Mugil cephalus, Nematalosa come, Pelates quadrilineatus, Platycephalus indicus, Pseudorhombus dupliciocellatus, Scatophagus argus, Sillago sihama, Sphyraena putnamae, Terapon jarbua, Valamugil cunnesius* | Cd, Cu, Pb, Se, Zn | Ag, As, Fe, Hg, Mn, Ni, |  | x | x |  |  | AAS, ICP-MS |  | x |  | x | x |  | [4] |
| *Acanthopagrus latus, Cynoglossus arel* | Cd, Cu, Pb, Zn | As, Cr, Hg, Ni, V | x | x |  |  |  | ICP-MS, ICP-OES |  | x |  |  |  | x | [5] |
| *Aldrichetta forsteri, Sillago schomburgkii* | Cd, Cu, Pb | - |  |  | x |  |  | PDV |  | x |  |  | x |  | [6] |
| *Alosa bulgarica, Dicentrarchus labrax, Engraulis encrasiocolus, Merlangius euxinus* | Cd, Cu, Pb, Zn | Co, Cr, Fe, Mn, Ni |  |  | x |  |  | AAS |  | x | x |  |  | x | [7] |
| *Amphilophus robertsoni, Astyanax aeneus, Batrachoides goldmani, Centropomus undecimalis, Cichlasoma salvini, Cichlasoma urophthalmus, Gobiomorus dormitor, Gobiomorus dormitor* larvae*, Oreochromis niloticusa, Parachromis managuensisa, Paraneetroplus bifasciatusa, Paraneetroplus synspilusa, Petenia splendidaa, Pterygoplichthys pardalis, Rhamdia quelen, Rocio octofasciata, Theraps heterospilusa, Thorichthys helleri, Thorichthys meeki, Thorichthys pasionis* | Cd, Pb, Zn | Cr, Ni, V |  |  | x |  |  | ICP-AES |  | x | x |  |  | x | [8] |
| *Anguilla anguilla* | Cd, Cu, Pb | As, Cr, Fe, Hg, Mn, Ni, V, aliphatic hydrocarbons, PAHs | x | x |  |  |  | AAS |  | x |  | x |  |  | [9] |
| *Apogon kiensis, Argyrosomus argentatus, Brachirus, Cynoglossus sinicus, Eleotriodes Bleeker, Harpodon nehereus, Johnius belengeri, Lagocephalus lunaris, Leiognathus rivulatus, Nibea soldado, Setipinna taty, Siganus oramin, Trypauchen vagina* | Cd, Cu, Pb, Zn | As, Cr |  |  | x |  |  | AAS | x | x |  |  |  | x | [10] |
| *Argyrosomus japonicus, Gilchristella aestuaria, Lichia amia, Mugil cephalus, Pomadasys commersonnii, Psammogobius knysnaensis* | Al, Cd, Cu, Pb, Se, Zn | As, Ag, Au, B, Ba, Be, Bi, Co, Cr, Fe, Hg, Mn, Mo, Ni, Pd, Pt, Rb, Sb, Sr, Th, Ti, Tl, U, V, PCBs, PAHs, OCPs |  | x | x |  | x | ICP-MS |  | x |  | x |  |  | [11] |
| *Arius parkii, Etroplus suratensis, Gerres oyena, Liza parsia, Oreochromis mossambicus, Sillago sihama* | Cd, Cu, Pb, Zn | Fe, Mn, Cr, Ni, Co | x | x | x |  | x | AAS | x | x |  |  |  | x | [12] |
| *Atherina boyeri, Alosa pontica, Mullus barbatus, Sprattus sprattus* | Cd, Cu, Pb | Cr, Ni |  |  | x |  |  | AAS | x | x |  |  |  | x | [13] |
| *Boops boops, Eutrigla gurnardus, Micromesistius poutassou, Mugil cephalus,Mullus surmuletus, Serranus cabrilla* | Cd, Cu, Pb, Se, Zn | As, Co, Cr, Cs, Fe, Ga, Ge, Hg, Li, Mo, Ni, Rb, Sr, Tl, Th, U, V, Y | x | x | x |  | x | ICP-MS |  | x | x |  |  | x | [14] |
| *Cathorops spixii* | Al, Cd, Cu, Pb, Zn | As, Cr, Fe, Hg, Ni |  |  |  | x |  | ICP-MS, AAS |  | x |  |  |  | x | [15] |
| *Centropomus parallelus* | Al, Cd, Cu, Pb, Se, Zn | As, Ag, Cr, Fe, Hg, Mn, Ni |  |  | x |  |  | ICP-MS | x | x |  | x |  |  | [16] |
| *Champsocephalus gunnari, Trematomus scotti* | Al, Cd, Cu, Pb, Se, Zn | Ag, As, Cr, Fe, Mn, Ni, Sr, Ti, V | x | x | x |  | x | ICP-AES | x | x |  |  |  | x | [17] |
| *Channa asiatica, Oreochromis mossambicus* | Cd, Cu, Pb, Zn | Cr, Mn, Ni |  |  | x |  | x | ICP-OES |  | x |  |  |  | x | [18] |
| *Chanos chanos* | Cd, Cu, Pb, Zn | Fe, Mn | x | x | x |  | x | AAS | x | x |  |  |  | x | [19] |
| *Cichla ocellaris, Geophagus brasiliensis, Hoplias malabaricus, Hoplosternum littorale, Mugil liza, Rhamdia quelen, Tilapia rendalli, Trachelyopterus striatulus* | Al, Cd, Cu, Pb, Zn | As, Cr, Fe, Hg, Mn, Ni | x | x | x |  |  | ICP-MS |  | x | x |  |  | x | [20] |
| *Coris julis* | Cd, Cu, Pb, Zn | Co, Cr, Ni, Sn | x |  |  |  |  | AAS |  | x |  |  | x |  | [21] |
| *Coris julis* | Cd, Pb | As, Cr, Hg, PAHs, PCBs, pesticides |  |  | x |  |  | ICP-MS | x | x |  |  | x |  | [22] |
| *Dentex* spp.*, Chloroscombrus chrysurus, Galeodes decadactylus, Trichurus lepturus* | Al, Cd, Cu, Pb, Se, Zn | Ba, Cr, Fe, Mn, Ni, Sr, Ti, U, V, Zr, PAHs |  |  | x |  |  | ICP-AES |  | x |  |  |  | x | [23] |
| *Dicentrarchus labrax* | Cd, Cu, Pb, Se, Zn | - |  | x | x |  |  | dPSA, dCSP | x |  | x |  |  | x | [24] |
| *Dicentrarchus labrax* | Cd, Cu, Pb, Se, Zn | As, Cr, Hg |  |  | x |  |  | ICP-OES |  |  | x |  |  | x | [25] |
| *Dicentrarchus labrax, Scophtalmus maximus* | Cd, Cu, Pb, Zn | Al, Cr, Hg, Mn, Ni, V, PAHs, PCBs | x |  |  |  |  | ICP-MS |  | x |  | x |  |  | [26] |
| *Dicentrarchus labrax, Sparus auratus* | Al, Cd, Cu, Pb, Se, Zn | As, Ba, Co, Cr, Cs, Fe, Ge, Hg, Li, Mo, Ni, Pd, Rb, Sr, V, U, Y | x | x | x |  | x | ICP-MS |  |  | x |  |  | x | [27] |
| *Diplodus sargus* | Cd, Cu, Pb | As |  | x | x |  |  | ICP-MS |  |  |  |  |  | x | [28] |
| *Diplodus vulgaris, Engraulis encrasicholus, Engraulis encrasicholus, Merluccius merluccius, Mullus surmuletus, Pagellus erythrinus, Sardina pilchardus, Scomber scombrus, Sparus aurata, Solea vulgaris, Trachurus trachurus, Thunnus thynnus, Xiphias gladius* | Cd, Cu, Pb, Zn | Ni, Hg |  |  | x |  |  | AAS | x |  |  |  |  | x | [29] |
| *Engraulis encrasicolus, Sardina pilchardus* | Pb | - |  | x | x |  | x | Alpha spectrometer | x |  | x |  |  | x | [30] |
| *Epinephelus coioides, Lethrinus nebulosus* | Cd, Cu, Pb, Zn | Ag, Al, As, Co, Cr, Fe, Hg, Mn, Ni, Sb, V |  | x | x |  |  | ICP-MS |  | x |  |  |  | x | [31] |
| *Fundulus heteroclitus* | Cd, Cu, Pb, Zn | - |  |  |  | x |  | ICP-MS | x | x |  |  |  | x | [32] |
| *Fundulus heteroclitus* | Cd, Cu, Pb, Zn | - |  |  |  | x |  | AAS |  | x | x |  | x |  | [33] |
| *Fundulus heteroclitus* | Cd, Cu, Pb, Zn | Hg, PCBs, PAHs |  | x |  |  | x | AAS |  | x |  |  |  | x | [34] |
| *Gadus morhua* L.*, Platichthys jlesus* L. | Cd, Cu, Zn | Hg, Ti, PAHs, PCBs |  | x |  |  |  | AAS |  | x |  |  |  | x | [35] |
| *Genyonemus lineatus, Hippoglossoides elassodon, Pleuronectes vetulus* | Cd, Pb | Hg | x | x | x |  | x | AAS |  | x |  |  |  | x | [36] |
| *Gerres cinereus, Haemulopsis leuciscus, Lutjanus argentiventris, Mugil cephalus* | Cd, Cu, Pb, Zn |  |  |  |  | x | x | AAS | x | x | x | x | x |  | [37] |
| *Halobatrachus didactylus* | Cd, Cu, Pb, Zn | Co, Cr, Ni |  | x |  |  |  | AAS |  | x |  |  | x |  | [38] |
| *Helicolenus dactylopterus, Lepidotrigla cavillone, Merluccius merluccius, Micromesistius poutassou, Scyliorhinus canicula, Trachurus trachurus, Trisopterus luscus* | Cd, Pb, Zn | - |  |  | x |  |  | IPC-MS | x |  |  |  |  | x | [39] |
| *Liza aurata* | Cd, Cu, Zn | Ni |  |  |  |  | x | PIXE, RBS, STIM |  | x |  |  |  | x | [40] |
| *Liza aurata* | Cd, Cu, Pb, Zn | Cr, Ni, Mn |  | x |  |  | x | ICP-MS | x | x |  | x |  |  | [41] |
| *Liza aurata* | Cd, Cu, Pb, Zn | Cr, Ni, Mn | x |  |  |  |  | AAS | x |  |  | x |  |  | [42] |
| *Liza aurata* | Cd, Cu, Pb, Zn | As, Hg |  |  |  |  | x | ICP-MS | x | x |  | x |  |  | [43] |
| *Liza dumerelii* | Al, Cu, Pb, Zn | Cr, Fe, Mn | x | x | x |  |  | AAS | x | x |  |  |  | x | [44] |
| *Liza klunzingeri* | Cd, Cu, Pb, Zn | - |  |  |  |  |  | AAS |  | x |  |  |  | x | [45] |
| *Liza ramada* | Cd, Cu, Pb, Zn | Fe, Mn, Ni |  | x | x |  |  | AAS | x |  |  |  |  | x | [46] |
| *Liza saliens* | Cu, Zn | - | x | x | x |  |  | AAS |  | x |  |  |  | x | [47] |
| *Lutjanus malabaricus, Saurida undosquamis, Stromateoides argenteus, Trachinotus blochii* | Cd, Cu, Pb, Zn | - |  |  | x | x |  | AAS | x | x | x |  |  | x | [48] |
| *Lutjanus malabaricus, Trachinotus blochii* | Cd, Cu, Pb, Zn | As, Cr, Hg |  |  | x | x |  | AAS | x | x | x |  |  | x | [49] |
| *Mugil cephalus* | Cd, Cu, Pb, Se, Zn | Cr, Fe, Hg, Mn, Ni |  |  |  |  | x | AAS | x |  |  |  |  | x | [50] |
| *Mugil cephalus* | Pb | - |  |  | x |  |  | ICP-MS | x |  | x | x | x |  | [51] |
| *Mullus barbatus* | Al, Cd, Cu, Pb, Zn | Cr, Hg, Mn, Ni |  |  | x |  |  | AAS |  | x |  |  |  | x | [52] |
| *Mullus barbatus* | Cd, Cu, Pb, Zn | As, Hg, PAHs, PCBs, DDTs, pesticides |  |  | x |  |  | AAS |  | x |  |  | x |  | [53] |
| *Mullus barbatus, Solea vulgaris* | Cd, Pb | Cr, Hg, PAHs |  |  |  |  |  | AAS |  | x |  |  |  | x | [52] |
| *Notothenia* spp.*, Trematomus newnesi* | Zn | Fe, Hg |  |  |  | x |  | AAS |  | x |  |  |  | x | [54] |
| *Parablennius sanguinolentus* | Cd, Pb | Cr, Hg, PAHs, PCBs, pesticides |  |  | x |  |  | AAS | x | x |  |  |  | x | [55] |
| *Platichthys flesus* | Cd, Cu, Pb, Se, Zn | As, Cr, Mn, Ni, V |  |  |  | x |  | ICP-MS |  | x |  | x |  |  | [56] |
| *Platichthys flesus* | Cd, Cu, Pb, Zn | Al, Cr, Hg, Mn, Ni, V, PAHs | x | x |  |  |  | ICP-MS |  | x |  | x |  |  | [57] |
| *Platichthys flesus* | Cd, Zn | As, Cr, Cu, Hg, Ni, Pb, OCPs, PAHs, PCBs, organotonins |  | x |  |  |  | Unknown | x | x |  |  | x |  | [58] |
| *Platichthys flesus* | Cd, Cu, Pb | Al, Cr, Fe, Hg, Mn, Ni, Zn, PCBs |  |  | x |  |  | Unknown |  | x |  |  |  | x | [59] |
| *Platichthys flesus* | Cd, Cu, Pb, Zn | - |  | x | x |  |  | AAS |  | x |  |  |  | x | [60] |
| *Platycephalus bassensis* | Se | Fe, Hg |  |  | x |  |  | HG-AFS |  | x |  |  |  | x | [61] |
| *Pseudorhombus jenynsii* | Cu, Se, Zn | As, Fe, Hg, Mn |  |  | x |  |  | ICP-MS, ICP-AES |  | x |  |  |  | x | [62] |
| *Sardinella brasiliensis* | Se | Hg |  |  | x |  |  | AAS |  |  | x |  |  | x | [63] |
| *Sciaenops ocellatus* | Al, Cd, Cu, Pb, Se, Zn | Ag, As, Fe, Hg, Mn, Ni, Sn |  |  |  |  |  | AAS |  | x |  |  |  | x | [64] |
| *Solea senegalensis* | Cd, Cu, Pb, Zn | As, Fe, PAHs |  |  | x |  |  | ICP-MS, ICP-AES | x | x |  | x |  |  | [65] |
| *Solea senegalensis* | Cd, Cu, Pb, Zn | As, Fe, PAHs | x | x |  |  |  | ICP-MS, ICP-AES | x | x |  | x |  |  | [66] |
| *Solea senegalensis* | Cd, Cu, Pb, Zn | As, Fe |  | x |  |  |  | ICP-MS, ICP-AES | x | x |  | x |  |  | [67] |
| *Solea senegalensis, Sparus aurata* | Cd, Cu, Pb, Zn | As | x | x | x |  |  | ICP-MS, ICP-AES | x | x |  |  | x |  | [68] |
| *Solea solea, Solea senegalensis* | Cd, Cu, Pb, Se, Zn, Se | Cr, Fe, Hg |  |  |  |  | x | ICP-MS |  | x |  |  |  | x | [69] |
| *Tetractenos glaber* | Al, Cd, Cu, Pb, Se, Zn | As, Co, Fe, Cr, Ni | x | x | x |  | x | ICP-MS, ICP-AES |  | x |  |  | x |  | [70] |
| *Trematomus bernacchii* | Cd | Hg | x | x | x |  | x | AAS | x | x |  |  |  | x | [71] |

Abbreviations: G=gill, L=liver, M=muscle, Wf=whole fish, O=other; W=water, S=sediment, F=food; J=juvenile, A=adult, U=unknown.

(b)

| **Species** | **Contaminants measured** | | **Tissue** | | | | | **Method** | **Exposure pathway** | | | **Life history stage** | | | **Reference** |
| --- | --- | --- | --- | --- | --- | --- | --- | --- | --- | --- | --- | --- | --- | --- | --- |
|  | **Fish** | **Environment** | **G** | **L** | **M** | **Wf** | **O** |  | **W** | **S** | **F** | **J** | **A** | **U** |  |
| *Acanthopagrus schlegeli* | Cd | - | x |  | x | x | x | ICP-MS | x |  | x | x |  |  | [72] |
| *Acanthopagrus schlegeli, Terapon jarbua* | Cd, Zn | - | x |  |  | x | x | ICP-MS | x |  | x |  |  | x | [73] |
| *Acanthopagrus schlegelii schlegelii* | Cu | - | x | x |  |  | x | ICP-OES | x |  | x | x |  |  | [74] |
| *Ambassis jacksoniensis* | Cd, Se, Zn | - |  |  |  | x |  | Gamma spectrometer | x |  | x |  |  | x | [75] |
| *Anguilla anguilla* | Cd, Cu, Pb, Zn | As, Cr, CrIV, Hg, Ni, V, PAHs | x | x |  |  | x | AAS |  | x |  | x |  |  | [76] |
| *Atherinops affinis* | Cu, Zn | - | x | x |  |  | x | ICP-MS | x |  |  | x |  |  | [77] |
| *Centropomus parallelus* | Cu | - | x |  |  |  |  | AAS | x |  |  | x |  |  | [78] |
| *Dicentrarchus labrax* | Cu | - |  | x | x |  |  | AAS | x |  |  |  |  | x | [79] |
| *Dicentrarchus labrax* | Cd | - | x |  |  |  | x | AAS | x |  |  |  |  | x | [80] |
| *Dicentrarchus labrax, Psetta maxima, Raja undulata, Scyliorhinus canicula, Sparus aurata, Torpedo marmorata* | Cd, Se, Zn | Ag, Am, Co, Cr, Cs, Mn |  |  |  |  |  | Gamma spectrometry | x |  |  |  |  | x | [81] |
| *Dicentrarchus labrax, Sparus auratus* | Cd, Se, Zn | Am, Co, Cs, Mn |  |  |  | x |  | Gamma spectrometer |  |  | x | x |  |  | [82], |
| *Fundulus heteroclitus* | Cd | As, Cr, Hg |  |  |  | x |  | Gamma dectector |  |  | x | x |  |  | [83] |
| *Fundulus heteroclitus* | Zn | - |  |  |  |  | x | AAS | x |  |  |  | x |  | [84] |
| *Fundulus heteroclitus* | Cu | - | x | x |  |  | x | AAS | x |  |  |  | x |  | [85] |
| *Galaxias maculatus* | Cu, Pb, Zn | - |  |  |  |  | x | ICP–MS | x | x |  | x |  |  | [86] |
| *Gobius niger* | Cd | - |  |  |  |  | x | dPASV | x |  |  |  | x |  | [87] |
| *Liza microlepis* | Cd, Cu, Zn | Fe, Mn, Ni | x | x | x | x | x | AAS |  | x | x |  |  | x | [88] |
| *Menidia beryllina* | Cd | - |  |  |  | x |  | AAS | x |  |  |  |  | x | [89] |
| *Menidia menidia* | Cd, Cu, Zn | Am, Hg |  |  |  |  | x | Gamma dectector | x |  | x | x |  |  | [90] |
| *Mugil cephalus, Terapon jarbua* | Pb | - |  |  |  |  |  | AAS | x |  |  | x | x |  | [91] |
| *Oryzias melastigma* | Cu | - |  |  |  | x |  | ICP-MS | x |  | x | x |  |  | [92] |
| *Paralichthys olivaceus* | Cd | - |  |  |  | x |  | AAS | x |  |  | x |  |  | [93] |
| *Paralichthys olivaceus* | Cd | - | x | x | x |  | x | AAS | x |  |  | x |  |  | [94] |
| *Platichthys flesus* | Cd, Cu, Pb, Se, Zn | As, Cr, Hg, Ni, CBs, PAHs, PBDEs, pesticides, organotonins |  | x | x |  |  | ICP-MS |  | x |  | x |  |  | [95] |
| *Platichthys flesus* | Cd, Pb | Ni | x | x | x |  | x | ICP-OES, AAS, ICP-MS | x |  |  | x |  |  | [96] |
| *Platichthys flesus* | Cd, Pb, Zn | Hg, PAHs, PCBs |  | x |  |  |  | AAS |  | x |  | x |  |  | [97] |
| *Platichthys flesus* | Cd, Cu, Pb, Se, Zn | As, Cr, Hg, Ni, CBs, PAHs, PBDEs, pesticides, organotonins |  | x | x |  |  | ICP-MS |  | x |  | x |  |  | [98] |
| *Poecilia vivipara* | Cu | - | x | x | x | x |  | n/a | x |  |  |  | x |  | [99] |
| *Pogonichthys macrolepidotus* | Se | Hg |  |  |  | x |  | ICP-MS |  |  | x | x |  |  | [100] |
| *Pogonichthys macrolepidotus* | Se | - |  | x | x |  |  | ICP-MS |  |  | x | x |  |  | [100] |
| *Rachycentron canadum* | Cd | - | x | x | x |  | x | AAS |  |  | x | x |  |  | [101] |
| *Scophthalmus maximus* | Cd, Cu, Pb, Se | Al, Cr, Mn, Ni, V, Zn, PAHs, PCBs | x |  |  |  |  | ICP-MS |  | x |  | x |  |  | [102] |
| *Scyliorhinus canicula* | Cd, Zn | Am, Co, Cs, Mn |  |  |  |  | x | Gamma spectrometry | x |  |  | x |  |  | [103] |
| *Scyliorhinus canicula, Psetta maxima* | Cd, Zn | Am, Co, Cr, Cs, Mn |  | x | x |  | x | Gamma spectrometry | x |  |  | x |  |  | [104] |
| *Seriola lalandi* | Se | - |  | x | x |  |  | AAS |  |  | x | x |  |  | [105] |
| *Siganus oramin* | Cu | - | x |  | x |  | x | ICP-MS | x |  | x | x |  |  | [72] |
| *Solea senegalensis* | Cd, Cu, Pb, Zn | As, Cr, Ni, PAHs, PCBs, DDTs |  | x |  |  |  | ICP-MS |  | x |  | x |  |  | [106] |
| *Sparus aurata* | Cd, Cu, Pb, Zn | As, Fe |  | x | x |  | x | AAS | x | x |  |  |  | x | [107] |
| *Sparus aurata* | Cu, Cd, Pb | As |  | x |  |  |  | ICP-AES |  | x |  | x |  |  | [108] |
| *Sparus aurata* | Cu | - |  | x |  |  | x | AAS | x |  |  | x |  |  | [109] |
| *Sparus aurata* | Cd, Cu, Zn | - | x | x | x |  | x | AAS | x |  |  |  |  | x | [110] |
| *Sparus aurata* | Cu, Zn | - | x | x |  |  | x | AAS | x |  | x | x |  |  | [111] |
| *Squalus acanthias* | Pb | - | x | x |  |  | x | ICP-MS | x |  |  |  |  | x | [112] |
| *Synechogobius hasta* | Cd | - |  | x | x | x | x | ICP-MS | x |  |  | x |  |  | [113] |
| *Terapon jarbua* | Cd | - | x | x |  |  | x | AAS | x |  | x | x |  |  | [114] |
| *Tetractenos glaber* | Cd, Se | - | x | x | x |  | x | Gamma spectrometer | x |  | x |  | x |  | [115] |

Abbreviations: G=gill, L=liver, M=muscle, Wf=whole fish, O=other; W=water, S=sediment, F=food; J=juvenile, A=adult, U=unknown.

# References

1. Cui B, Zhang Q, Zhang K, Liu X, Zhang H. Analyzing trophic transfer of heavy metals for food webs in the newly-formed wetlands of the Yellow River Delta, China. Environ Pollut. 2011; 159: 1297-306. doi: 10.1016/j.envpol.2011.01.024 PMID: 000290192400037
2. Waltham NJ, Teasdale PR, Connolly RM. Contaminants in water, sediment and fish biomonitor species from natural and artificial estuarine habitats along the urbanized Gold Coast, Queensland. J Environ Monit. 2011; 13: 3409-19. doi: 10.1039/c1em10664c PMID: 000297558200012
3. Peters GM, Maher WA, Krikowa F, Roach AC, Jeswani HK, Barford JP, et al. Selenium in sediments, pore waters and benthic infauna of Lake Macquarie, New South Wales, Australia. Mar Environ Res. 1999; 47: 491-508.
4. Chen MH. Baseline metal concentrations in sediments and fish, and the determination of bioindicators in the subtropical Chi-ku Lagoon, SW Taiwan. Mar Pollut Bull. 2002; 44: 703-14. doi: 10.1016/s0025-326x(02)00066-8 PMID: 000177488800028
5. Beg MU, Al-Jandal N, Al-Subiai S, Karam Q, Husain S, Butt SA, et al. Metallothionein, oxidative stress and trace metals in gills and liver of demersal and pelagic fish species from Kuwaits’ marine area. Mar Pollut Bull. 2015; 100: 662-72. doi: 10.1016/j.marpolbul.2015.07.058
6. Edwards JW, Edyvane KS, Boxall VA, Hamann M, Soole KL. Metal levels in seston and marine fish flesh near industrial and metropolitan centres in South Australia. Mar Pollut Bull. 2001; 42: 389-96. doi: 10.1016/s0025-326x(00)00168-5 PMID: 000169401400018
7. Topcuoglu S, Kirbasoglu C, Gungor N. Heavy metals in organisms and sediments from Turkish Coast of the Black Sea, 1997-1998. Environ Int. 2002; 27: 521-6. doi: 10.1016/s0160-4120(01)00099-x PMID: 000173840500001
8. Mendoza-Carranza M, Sepulveda-Lozada A, Dias-Ferreira C, Geissen V. Distribution and bioconcentration of heavy metals in a tropical aquatic food web: A case study of a tropical estuarine lagoon in SE Mexico. Environ Pollut. 2016; 210: 155-65. doi: 10.1016/j.envpol.2015.12.014 PMID: 000376703600019
9. Piva F, Ciaprini F, Onorati F, Benedetti M, Fattorini D, Ausili A, et al. Assessing sediment hazard through a weight of evidence approach with bioindicator organisms: a practical model to elaborate data from sediment chemistry, bioavailability, biomarkers and ecotoxicological bioassays. Chemosphere. 2011; 83: 475-85. doi: 10.1016/j.chemosphere.2010.12.064 PMID: 21239037
10. Zhang L, Shi Z, Jiang Z, Zhang J, Wang F, Huang X. Distribution and bioaccumulation of heavy metals in marine organisms in east and west Guangdong coastal regions, South China. Mar Pollut Bull. 2015; 101: 930-7. doi: 10.1016/j.marpolbul.2015.10.041 PMID: 000367107600056
11. Nel L, Strydom NA, Bouwman H. Preliminary assessment of contaminants in the sediment and organisms of the Swartkops Estuary, South Africa. Mar Pollut Bull. 2015; 101: 878-85. doi: 10.1016/j.marpolbul.2015.11.015 PMID: 000367107600049
12. Jayaprakash M, Kumar RS, Giridharan L, Sujitha SB, Sarkar SK, Jonathan MP. Bioaccumulation of metals in fish species from water and sediments in macrotidal Ennore creek, Chennai, SE coast of India: A metropolitan city effect. Ecotoxicol Environ Saf. 2015; 120: 243-55. doi: 10.1016/j.ecoenv.2015.05.042 PMID: 000359029000033
13. Jitar O, Teodosiu C, Oros A, Plavan G, Nicoara M. Bioaccumulation of heavy metals in marine organisms from the Romanian sector of the Black Sea. New Biotechnol. 2015; 32: 369-78. doi: 10.1016/j.nbt.2014.11.004 PMID: 000353346000007
14. Kalantzi I, Papageorgiou N, Sevastou K, Black KD, Pergantis SA, Karakassis I. Metals in benthic macrofauna and biogeochemical factors affecting their trophic transfer to wild fish around fish farm cages. Sci Total Environ. 2014; 470: 742-53. doi: 10.1016/j.scitotenv.2013.10.020 PMID: 000331415600081
15. Mohammed A, May T, Echols K, Walther M, Manoo A, Maraj D, et al. Metals in sediments and fish from Sea Lots and Point Lisas Harbors, Trinidad and Tobago. Mar Pollut Bull. 2012; 64: 169-73. doi: 10.1016/j.marpolbul.2011.10.036 PMID: 000299981100035
16. Souza IC, Duarte ID, Pimentel NQ, Rocha LD, Morozesk M, Bonomo MM, et al. Matching metal pollution with bioavailability, bioaccumulation and biomarkers response in fish (*Centropomus parallelus*) resident in neotropical estuaries. Environ Pollut. 2013; 180: 136-44. doi: 10.1016/j.envpol.2013.05.017 PMID: 000322425300019
17. Deheyn DD, Gendreau P, Baldwin RJ, Latz MI. Evidence for enhanced bioavailability of trace elements in the marine ecosystem of Deception Island, a volcano in Antarctica. Mar Environ Res. 2005; 60: 1-33. doi: 10.1016/j.marenvres.2004.08.001 PMID: 000227597000001
18. Kwok CK, Liang Y, Wang H, Dong YH, Leung SY, Wong MH. Bioaccumulation of heavy metals in fish and Ardeid at Pearl River Estuary, China. Ecotoxicol Environ Saf. 2014; 106: 62-7. doi: 10.1016/j.ecoenv.2014.04.016 PMID: 000337643800010
19. Rajeshkumar S, Munuswamy N. Impact of metals on histopathology and expression of HSP 70 in different tissues of Milk fish (*Chanos chanos*) of Kaattuppalli Island, South East Coast, India. Chemosphere. 2011; 83: 415-21. doi: 10.1016/j.chemosphere.2010.12.086 PMID: 21257190
20. Pereira AA, van Hattum B, de Boer J, van Bodegom PM, Rezende CE, Salomons W. Trace Elements and Carbon and Nitrogen Stable Isotopes in Organisms from a Tropical Coastal Lagoon. Arch Environ Contam Toxicol. 2010; 59: 464-77. doi: 10.1007/s00244-010-9489-2 PMID: 000281376500012
21. Fasulo S, Mauceri A, Maisano M, Giannetto A, Parrino V, Gennuso F, et al. Immunohistochemical and molecular biomarkers in *Coris julis* exposed to environmental contaminants. Ecotoxicol Environ Saf. 2010; 73: 873-82. doi: 10.1016/j.ecoenv.2009.12.025 PMID: 000279623800023
22. Tomasello B, Copat C, Pulvirenti V, Ferrito V, Ferrante M, Renis M, et al. Biochemical and bioaccumulation approaches for investigating marine pollution using Mediterranean rainbow wrasse, *Coris julis* (Linneaus 1798). Ecotoxicol Environ Saf. 2012; 86: 168-75. doi: 10.1016/j.ecoenv.2012.09.012 PMID: 000311064800024
23. Gnandi K, Bandowe BAM, Deheyn DD, Porrachia M, Kersten M, Wilcke W. Polycyclic aromatic hydrocarbons and trace metal contamination of coastal sediment and biota from Togo. J Environ Monit. 2011; 13: 2033-41. doi: 10.1039/c1em10063g PMID: 000292984100020
24. Dugo G, La Pera L, Bruzzese A, Pellicano TM, Lo Turco V. Concentration of Cd (II), Cu (II), Pb (II), Se (IV) and Zn (II) in cultured sea bass (*Dicentrarchus labrax*) tissues from Tyrrhenian Sea and Sicilian Sea by derivative stripping potentiometry. Food Control. 2006; 17: 146-52. doi: 10.1016/j.foodcont.2004.09.014 PMID: 000232013400010
25. Trocino A, Xiccato G, Majolini D, Tazzoli M, Tulli F, Tibaldi E, et al. Levels of dioxin-like polychlorinated biphenyls (DL-PCBs) and metals in European sea bass from fish farms in Italy. Food Chem. 2012; 134: 333-8. doi: 10.1016/j.foodchem.2012.02.153 PMID: 000304291400045
26. Kerambrun E, Henry F, Courcot L, Gevaert F, Amara R. Biological responses of caged juvenile sea bass (*Dicentrarchus labrax*) and turbot (*Scophtalmus maximus*) in a polluted harbour. Ecol Indic. 2012; 19: 161-71. doi: 10.1016/j.ecolind.2011.06.035
27. Kalantzi I, Pergantis SA, Black KD, Shimmield TM, Papageorgiou N, Tsapakis M, et al. Metals in tissues of seabass and seabream reared in sites with oxic and anoxic substrata and risk assessment for consumers. Food Chem. 2016; 194: 659-70. doi: 10.1016/j.foodchem.2015.08.072 PMID: 000364248900087
28. Ferreira M, Caetano M, Costa J, Pousao-Ferreira P, Vale C, Reis-Henriques MA. Metal accumulation and oxidative stress responses in, cultured and wild, white seabream from Northwest Atlantic. Sci Total Environ. 2008; 407: 638-46. doi: 10.1016/j.scitotenv.2008.07.058 PMID: 000261877900060
29. Papetti P, Rossi G. Heavy metals in the fishery products of low Lazio and the use of metallothionein as a biomarker of contamination. Environ Monit Ass. 2009; 159: 589-98. doi: 10.1007/s10661-008-0725-4 PMID: 000271530400046
30. Strady E, Harmelin-Vivien M, Chiffoleau JF, Veron A, Tronczynski J, Radakovitch O. Po-210 and Pb-210 trophic transfer within the phytoplankton-zooplankton -anchovy/sardine food web: a case study from the Gulf of Lion (NW Mediterranean Sea). J Environ Radioactiv. 2015; 143: 141-51. doi: 10.1016/j.jenvrad.2015.02.019 PMID: 000353096600019
31. de Mora S, Fowler SW, Wyse E, Azemard S. Distribution of heavy metals in marine bivalves, fish and coastal sediments in the Gulf and Gulf of Oman. Mar Pollut Bull. 2004; 49: 410-24. doi: 10.1016/j.marpolbul.2004.02.029 PMID: 000223817200019
32. Broadley HJ, Buckman KL, Bugge DM, Chen CY. Spatial Variability of Metal Bioaccumulation in Estuarine Killifish (*Fundulus heteroclitus*) at the Callahan Mine Superfund Site, Brooksville, ME. Arch Environ Contam Toxicol. 2013; 65: 765-78. doi: 10.1007/s00244-013-9952-y PMID: 000327101700015
33. Goto D, Wallace WG. Relevance of intracellular partitioning of metals in prey to differential metal bioaccumulation among populations of mummichogs (*Fundulus heteroclitus*). Mar Environ Res. 2009; 68: 257-67. doi: 10.1016/j.marenvres.2009.06.015 PMID: 000270631300006
34. Weis JS, Samson J, Zhou T, Skurnick J, Weis P. Prey capture ability of mummichogs (*Fundulus heteroclitus*) as a behavioral biomarker for contaminants in estuarine systems. Can J Fish Aquat Sci. 2001; 58: 1442-52. doi: 10.1139/cjfas-58-7-1442 PMID: 000169776600017
35. Beyer J, Sandvik M, Hylland K, Fjeld E, Egaas E, Aas E, et al. Contaminant accumulation and biomarker responses in flounder (*Platichthys flesus* L) and Atlantic cod (*Gadus morhua* L) exposed by caging to polluted sediments in Sorfjorden, Norway. Aquat Toxicol. 1996; 36: 75-98. doi: 10.1016/s0166-445x(96)00798-9 PMID: A1996VY98200005
36. Meador JP, Ernest DW, Kagley AN. A comparison of the non-essential elements cadmium, mercury, and lead found in fish and sediment from Alaska and California. Sci Total Environ. 2005; 339: 189-205. doi: 10.1016/j.scitotenv.2004.07.028 PMID: 000227915700016
37. Jara-Marini ME, Soto-Jimenez MF, Paez-Osuna F. Trophic relationships and transference of cadmium, copper, lead and zinc in a subtropical coastal lagoon food web from SE Gulf of California. Chemosphere. 2009; 77: 1366-73. doi: 10.1016/j.chemosphere.2009.09.025 PMID: 000272519900015
38. Pedro S, Duarte B, Castro N, Almeida PR, Cacador I, Costa JL. The Lusitanian toadfish as bioindicator of estuarine sediment metal burden: The influence of gender and reproductive metabolism. Ecol Indic. 2015; 48: 370-9. doi: 10.1016/j.ecolind.2014.08.041 PMID: 000347495100039
39. Raimundo J, Pereira P, Caetano M, Cabrita MT, Vale C. Decrease of Zn, Cd and Pb concentrations in marine fish species over a decade as response to reduction of anthropogenic inputs: The example of Tagus estuary. Mar Pollut Bull. 2011; 62: 2854-8. doi: 10.1016/j.marpolbul.2011.09.020 PMID: 000298520400047
40. Godinho RM, Pereira P, Raimundo J, Pacheco M, Pinheiro T. Elemental mapping inventory of the fish *Liza aurata* brain: a biomarker of metal pollution vulnerability. Metallomics. 2015; 7: 277-82. doi: 10.1039/c4mt00281d PMID: 000349470000009
41. Pereira P, de Pablo H, Pacheco M, Vale C. The relevance of temporal and organ specific factors on metals accumulation and biochemical effects in feral fish (*Liza aurata*) under a moderate contamination scenario. Ecotoxicol Environ Saf. 2010; 73: 805-16. doi: 10.1016/j.ecoenv.2010.02.020 PMID: 000279623800015
42. Pereira P, de Pablo H, Vale C, Pacheco M. Combined use of environmental data and biomarkers in fish (*Liza aurata*) inhabiting a eutrophic and metal-contaminated coastal system - Gills reflect environmental contamination. Mar Environ Res. 2010; 69: 53-62. doi: 10.1016/j.marenvres.2009.08.003 PMID: 000274773800001
43. Pereira P, Raimundo J, Canario J, Almeida A, Pacheco M. Looking at the aquatic contamination through fish eyes - A faithful picture based on metals burden. Mar Pollut Bull. 2013; 77: 375-9. doi: 10.1016/j.marpolbul.2013.10.009 PMID: 000329888600059
44. Mzimela HM, Wepener V, Cyrus DP. Seasonal variation of selected metals in sediments, water and tissues of the groovy mullet, *Liza dumerelii* (Mugilidae) from the Mhlathuze Estuary, South Africa. Mar Pollut Bull. 2003; 46: 659-64. doi: 10.1016/s0025-326x(03)00088-2 PMID: 000183139000026
45. Bastami KD, Afkhami M, Mohammadizadeh M, Ehsanpour M, Chambari S, Aghaei S, et al. Bioaccumulation and ecological risk assessment of heavy metals in the sediments and mullet *Liza klunzingeri* in the northern part of the Persian Gulf. Mar Pollut Bull. 2015; 94: 329-34. doi: 10.1016/j.marpolbul.2015.01.019 PMID: 000355357400046
46. Blasco J, Arias AM, Saenz V. Heavy metals in organisms of the River Guadalquivir estuary: possible incidence of the Aznalcollar disaster. Sci Total Environ. 1999; 242: 249-59. doi: 10.1016/s0048-9697(99)00394-0 PMID: 000084565400018
47. Fernandes C, Fontainhas-Fernandes A, Monteiro SM, Salgado MA. Histopathological gill changes in wild leaping grey mullet (*Liza saliens*) from the esmoriz-paramos coastal Lagoon, Portugal. Environ Toxicol. 2007; 22: 443-8. doi: 10.1002/tox.20269 PMID: 000248189800012
48. Qiu Y-W. Bioaccumulation of heavy metals both in wild and mariculture food chains in Daya Bay, South China. Estuar Coast Shelf S. 2015; 163: 7-14. doi: 10.1016/j.ecss.2015.05.036 PMID: 000362604800002
49. Qiu Y-W, Lin D, Liu J-Q, Zeng EY. Bioaccumulation of trace metals in farmed fish from South China and potential risk assessment. Ecotoxicol Environ Saf. 2011; 74: 284-93. doi: 10.1016/j.ecoenv.2010.10.008 PMID: 000288226700011
50. Padmini E, Rani MU. Evaluation of oxidative stress biomarkers in hepatocytes of grey mullet inhabiting natural and polluted estuaries. Sci Total Environ. 2009; 407: 4533-41. doi: 10.1016/j.scitotenv.2009.04.005 PMID: 000267631700019
51. Soto-Jimenez MF, Paez-Osuna F, Scelfo G, Hibdon S, Franks R, Aggarawl J, et al. Lead pollution in subtropical ecosystems on the SE Gulf of California Coast: A study of concentrations and isotopic composition. Mar Environ Res. 2008; 66: 451-8. doi: 10.1016/j.marenvres.2008.07.009 PMID: 000260640400008
52. Kucuksezgin F, Kontas A, Altay O, Uluturhan E, Darilmaz E. Assessment of marine pollution in Izmir Bay: Nutrient, heavy metal and total hydrocarbon concentrations. Environ Internat. 2006; 32: 41-51. doi: 10.1016/j.envint.2005.04.007 PMID: 000234475000006
53. Martinez-Gomez C, Fernandez B, Benedicto J, Valdes J, Campillo JA, Leon VM, et al. Health status of red mullets from polluted areas of the Spanish Mediterranean coast, with special reference to Portman (SE Spain). Mar Environ Res. 2012; 77: 50-9. doi: 10.1016/j.marenvres.2012.02.002 PMID: 000304296700008
54. dos Santos IR, Silva EV, Schaefer C, Sella SM, Silva CA, Gomes V, et al. Baseline mercury and zinc concentrations in terrestrial and coastal organisms of Admiralty Bay, Antarctica. Environ Pollut. 2006; 140: 304-11. doi: 10.1016/j.envpol.2005.07.007 PMID: 000235133700012
55. Tigano C, Tomasello B, Pulvirenti V, Ferrito V, Copat C, Carpinteri G, et al. Assessment of environmental stress in *Parablennius sanguinolentus* (Pallas, 1814) of the Sicilian Ionian coast. Ecotoxicol Environ Saf. 2009; 72: 1278-86. doi: 10.1016/j.ecoenv.2008.09.028 PMID: 000265767900037
56. Henry F, Filipuci I, Billon G, Courcot L, Kerambrun E, Amara R. Metal concentrations, growth and condition indices in European juvenile flounder (*Platichthys flesus*) relative to sediment contamination levels in four Eastern English Channel estuaries. J Environ Monitor. 2012; 14: 3211-9. doi: 10.1039/c2em30765k PMID: 000312655800017
57. Kerambrun E, Henry F, Cornille V, Courcot L, Amara R. A combined measurement of metal bioaccumulation and condition indices in juvenile European flounder, *Platichthys flesus*, from European estuaries. Chemosphere. 2013; 91: 498-505. doi: 10.1016/j.chemosphere.2012.12.010 PMID: 000317325700010
58. Schipper CA, Lahr J, van den Brink PJ, George SG, Hansen P-D, de Assis HCdS, et al. A retrospective analysis to explore the applicability of fish biomarkers and sediment bioassays along contaminated salinity transects. Ices J Mar Sci. 2009; 66: 2089-105. doi: 10.1093/icesjms/fsp194 PMID: 000272080600003
59. Schmidt V, Zander S, Korting W, Broeg K, von Westernhagen H, Dizer H, et al. Parasites of flounder (*Platichthys flesus* L.) from the German Bight, North Sea, and their potential use in biological effects monitoring - C. Pollution effects on the parasite community and a comparison to biomarker responses. Helgoland Mar Res. 2003; 57: 262-71. doi: 10.1007/s10152-003-0159-x PMID: 000186604600015
60. Vinagre C, Franca S, Costa MJ, Cabral HN. Accumulation of heavy metals by flounder, *Platichthys flesus* (Linnaeus 1758), in a heterogeneously contaminated nursery area. Mar Pollut Bull. 2004; 49: 1109-13. doi: 10.1016/j.marpolbul.2004.08.021 PMID: 000225926700039
61. Jones HJ, Swadling KM, Butler ECV, Macleod CK. Complex patterns in fish - sediment mercury concentrations in a contaminated estuary: The influence of selenium co-contamination? Estuar Coast Shelf Sci. 2014; 137: 14-22. doi: 10.1016/j.ecss.2013.11.024 PMID: 000331507000002
62. McKinley AC, Taylor MD, Johnston EL. Relationships between body burdens of trace metals (As, Cu, Fe, Hg, Mn, Se, and Zn) and the relative body size of small tooth flounder (*Pseudorhombus jenynsii*). Sci Total Environ. 2012; 423: 84-94. doi: 10.1016/j.scitotenv.2012.02.007 PMID: 000303231200011
63. Seixas TG, Moreira I, Kehrig HA. Mercury and selenium in seston, marine plankton and fish (*Sardinella brasiliensis*) as a tool for understanding a tropical food web. Mar Pollut Bull. 2015; 101: 366-9. doi: 10.1016/j.marpolbul.2015.10.016 PMID: 000367630700053
64. Park J, Presley BJ. Trace metals contamination of sediments and organisms from the Swan Lake area of Galveston Bay. Environ Pollut. 1997; 98: 209-21. doi: 10.1016/s0269-7491(97)00137-1 PMID: 000072110500009
65. Oliva M, Antonio Perales J, Gravato C, Guilhermino L, Dolores Galindo-Riano M. Biomarkers responses in muscle of Senegal sole (*Solea senegalensis*) from a heavy metals and PAHs polluted estuary. Mar Pollut Bull. 2012; 64: 2097-108. doi: 10.1016/j.marpolbul.2012.07.017 PMID: 000310929500028
66. Oliva M, Gravato C, Guilhermino L, Dolores Galindo-Riano M, Antonio Perales J. EROD activity and cytochrome P4501A induction in liver and gills of Senegal sole *Solea senegalensis* from a polluted Huelva Estuary (SW Spain). Comp Biochem Phys C. 2014; 166: 134-44. doi: 10.1016/j.cbpc.2014.07.010 PMID: 000342532000015
67. Oliva M, Jose Vicente J, Gravato C, Guilhermino L, Dolores Galindo-Riano M. Oxidative stress biomarkers in Senegal sole, *Solea senegalensis*, to assess the impact of heavy metal pollution in a Huelva estuary (SW Spain): Seasonal and spatial variation. Ecotoxicol Environ Saf. 2012; 75: 151-62. doi: 10.1016/j.ecoenv.2011.08.017 PMID: 000297088500020
68. Vicente-Martorell JJ, Galindo-Riano MD, Garcia-Vargas M, Granado-Castro MD. Bioavailability of heavy metals monitoring water, sediments and fish species from a polluted estuary. J Hazard Mat. 2009; 162: 823-36. doi: 10.1016/j.jhazmat.2008.05.106 PMID: 000263370200030
69. Siscar R, Torreblanca A, Palanques A, Sole M. Metal concentrations and detoxification mechanisms in *Solea solea* and *Solea senegalensis* from NW Mediterranean fishing grounds. Mar Pollut Bull. 2013; 77: 90-9. doi: 10.1016/j.marpolbul.2013.10.026 PMID: 000329888600025
70. Alquezar R, Markich SJ, Booth DJ. Metal accumulation in the smooth toadfish, *Tetractenos glaber*, in estuaries around Sydney, Australia. Environ Pollut. 2006; 142: 123-31. doi: 10.1016/j.envpol.2005.09.010 PMID: 000237644600016
71. Riva SD, Abelmoschi ML, Magi E, Soggia F. The utilization of the antarctic environmental specimen bank (BCAA) in monitoring Cd and Hg in an antarctic coastal area in Terra Nova Bay (Ross Sea-Northern Victoria Land). Chemosphere. 2004; 56: 59-69. doi: 10.1016/j.chemosphere.2003.12.026 PMID: 000221727700008
72. Guo Z, Zhang W, Du S, Zhou Y, Gao N, Zhang L, et al. Feeding reduces waterborne Cu bioaccumulation in a marine rabbitfish *Siganus oramin*. Environ Pollut. 2016; 208: 580-9. doi: 10.1016/j.envpol.2015.10.032 PMID: 000368306500033
73. Zhang L, Wang WX. Effects of Zn pre-exposure on Cd and Zn bioaccumulation and metallothionein levels in two species of marine fish. Aquat Toxicol. 2005; 73: 353-69. doi: 10.1016/j.aquatox.2005.04.001 PMID: 000230798000003
74. Dang F, Wang W-X, Rainbow PS. Unifying Prolonged Copper Exposure, Accumulation, and Toxicity from Food and Water in a Marine Fish. Environ Sci Technol. 2012; 46: 3465-71. doi: 10.1021/es203951z PMID: 000301630200055
75. Creighton N, Twining J. Bioaccumulation from food and water of cadmium, selenium and zinc in an estuarine fish, *Ambassis jacksoniensis*. Mar Pollut Bull. 2010; 60: 1815-21. doi: 10.1016/j.marpolbul.2010.05.025 PMID: 000283899500023
76. Benedetti M, Ciaprini F, Piva F, Onorati F, Fattorini D, Notti A, et al. A multidisciplinary weight of evidence approach for classifying polluted sediments: Integrating sediment chemistry, bioavailability, biomarkers responses and bioassays. Environ Int. 2012; 38: 17-28. doi: 10.1016/j.envint.2011.08.003 PMID: 21982029
77. Rose WL, Nisbet RM, Green PG, Norris S, Fan T, Smith EH, et al. Using an integrated approach to link biomarker responses and physiological stress to growth impairment of cadmium-exposed larval topsmelt. Aquat Toxicol. 2006; 80: 298-308. doi: 10.1016/j.aquatox.2006.09.007 PMID: 000242776900010
78. Oliveira BL, Loureiro Fernandes LF, Bianchini A, Chippari-Gomes AR, Silva BF, Brandao GP, et al. Acute copper toxicity in juvenile fat snook *Centropomus parallelus* (Teleostei: Centropomidae) in sea water. Neotrop Ichthyol. 2014; 12: 845-52. doi: 10.1590/1982-0224-20140040 PMID: 000347909800020
79. Cotou E, Henry M, Zeri C, Rigos G, Torreblanca A, Catsiki V-A. Short-term exposure of the European sea bass *Dicentrarchus labrax* to copper-based antifouling treated nets: Copper bioavailability and biomarkers responses. Chemosphere. 2012; 89: 1091-7. doi:10.1016/j.chemosphere.2012.05.075
80. Faucher K, Fichet D, Miramand P, Lagardere J-P. Impact of chronic cadmium exposure at environmental dose on escape behaviour in sea bass (*Dicentrarchus labrax* L.; Teleostei, Moronidae). Environ Pollut. 2008; 151: 148-57. doi: 10.1016/j.envpol.2007.02.017 PMID: 000252766500017
81. Jeffree RA, Oberhansli F, Teyssie J-L. Phylogenetic consistencies among chondrichthyan and teleost fishes in their bioaccumulation of multiple trace elements from seawater. Sci Total Environ. 2010; 408: 3200-10. doi: 10.1016/j.scitotenv.2010.04.015 PMID: 000279773200020
82. Mathews T, Fisher NS. Trophic transfer of seven trace metals in a four-step marine food chain. Mar Ecol-Prog Ser. 2008; 367: 23-33. doi: 10.3354/meps07536 PMID: 000260017900003
83. Dutton J, Fisher NS. Bioaccumulation of As, Cd, Cr, Hg(II), and MeHg in killifish (*Fundulus heteroclitus*) from amphipod and worm prey. Sci Total Environ. 2011; 409: 3438-47. doi: 10.1016/j.scitotenv.2011.05.022 PMID: 000293260100021
84. Loro VL, Nogueira L, Nadella SR, Wood CM. Zinc bioaccumulation and ionoregulatory impacts in *Fundulus heteroclitus* exposed to sublethal waterborne zinc at different salinities. Comp Biochem Phys C. 2014; 166: 96-104. doi: 10.1016/j.cbpc.2014.07.004 PMID: 000342532000011
85. Ransberry VE, Morash AJ, Blewett TA, Wood CM, McClelland GB. Oxidative stress and metabolic responses to copper in freshwater- and seawater-acclimated killifish, *Fundulus heteroclitus*. Aquat Toxicol. 2015; 161: 242-52. doi: 10.1016/j.aquatox.2015.02.013 PMID: 000352177500026
86. Barbee NC, Ganio K, Swearer SE. Integrating multiple bioassays to detect and assess impacts of sublethal exposure to metal mixtures in an estuarine fish. Aquat Toxicol. 2014; 152: 244-55. doi: 10.1016/j.aquatox.2014.04.012 PMID: 000338607300027
87. Migliarini B, Campisi AM, Maradonna F, Truzzi C, Annibaldi A, Scarponi G, et al. Effects of cadmium exposure on testis apoptosis in the marine teleost *Gobius niger*. Gen Comp Endocrinol. 2005; 142: 241-7. doi: 10.1016/j.ygcen.2004.12.012 PMID: 15862569
88. Chen MH, Chen CY. Bioaccumulation of sediment-bound heavy metals in grey mullet, *Liza macrolepis*. Mar Pollut Bull. 1999; 39: 239-44. doi: 10.1016/s0025-326x(99)00027-2 PMID: 000083356500034
89. Jackson CS, Sneddon J, Heagler MG, Lindow AG, Beck JN. Use of flame atomic absorption spectrometry and the effect of water chemistry for the study of the bioaccumulation of cadmium in *Menidia beryllina* (cope), the tidewater silverside. Microchem J. 2003; 75: 23-8. doi: 10.1016/s0026-265x(03)00048-1 PMID: 000184766600003
90. Dutton J, Fisher NS. Intraspecific comparisons of metal bioaccumulation in the juvenile Atlantic silverside *Menidia menidia*. Aquat Biol. 2010; 10: 211-26. doi: 10.3354/ab00276 PMID: 000283445200002
91. Hariharan G, Purvaja R, Ramesh R. Environmental safety level of lead (Pb) pertaining to toxic effects on grey mullet (*Mugil cephalus*) and Tiger perch (*Terapon jarbua*). Environ Toxicol. 2016; 31: 24-43. doi: 10.1002/tox.22019 PMID: 000366585300003
92. Guo Z, Zhang W, Du S, Green I, Tan Q, Zhang L. Developmental patterns of copper bioaccumulation in a marine fish model *Oryzias melastigma*. Aquat Toxicol. 2016; 170: 216-22. doi: 10.1016/j.aquatox.2015.11.026 PMID: 000368564500022
93. Cao L, Huang W, Liu J, Yin X, Dou S. Accumulation and oxidative stress biomarkers in Japanese flounder larvae and juveniles under chronic cadmium exposure. Comp Biochem Phys C. 2010; 151: 386-92. doi: 10.1016/j.cbpc.2010.01.004 PMID: 000275627400016
94. Cao L, Huang W, Shan X, Ye Z, Dou S. Tissue-specific accumulation of cadmium and its effects on antioxidative responses in Japanese flounder juveniles. Environ Toxicol Pharmacol. 2012; 33: 16-25. doi: 10.1016/j.etap.2011.10.003 PMID: 000301876600003
95. Leaver MJ, Diab A, Boukouvala E, Williams TD, Chipman JK, Moffat CF, et al. Hepatic gene expression in flounder chronically exposed to multiply polluted estuarine sediment: Absence of classical exposure 'biomarker' signals and induction of inflammatory, innate immune and apoptotic pathways. Aquat Toxicol. 2010; 96: 234-45. doi: 10.1016/j.aquatox.2009.10.025 PMID: 000274950000007
96. Selleslagh J, Echard A, Pecheyran C, Baudrimont M, Lobry J, Daverat F. Can analysis of *Platichthys flesus* otoliths provide relevant data on historical metal pollution in estuaries? Experimental and in situ approaches. Sci Total Environ. 2016; 557: 20-30. doi: 10.1016/j.scitotenv.2016.03.014 PMID: 000375136200003
97. Vethaak AD, Jol JG, Meijboom A, Eggens ML, apRheinallt T, Wester PW, et al. Skin and liver diseases induced in flounder (*Platichthys flesus*) after long-term exposure to contaminated sediments in large-scale mesocosms. Environ Health Persp. 1996; 104: 1218-29. doi: 10.2307/3432916 PMID: A1996VX74000021
98. Williams TD, Davies IM, Wu H, Diab AM, Webster L, Viant MR, et al. Molecular responses of European flounder (*Platichthys flesus*) chronically exposed to contaminated estuarine sediments. Chemosphere. 2014; 108: 152-8. doi: 10.1016/j.chemosphere.2014.01.028 PMID: 000337881600020
99. de Souza Machado AA, Mueller Hoff ML, Klein RD, Cardozo JG, Giacomin MM, Ledes Pinho GL, et al. Biomarkers of waterborne copper exposure in the guppy *Poecilia vivipara* acclimated to salt water. Aquat Toxicol. 2013; 138: 60-9. doi: 10.1016/j.aquatox.2013.04.009. PMID: 000322293600007
100. Deng DF, Teh FC, Teh SJ. Effect of dietary methylmercury and seleno-methionine on Sacramento splittail larvae. Sci Total Environ. 2008; 407: 197-203. doi: 10.1016/j.scitotenv.2008.08.028 PMID: 000261877900019
101. Liu K, Chi S, Liu H, Dong X, Yang Q, Zhang S, et al. Toxic effects of two sources of dietborne cadmium on the juvenile cobia, *Rachycentron canadum* L. and tissue-specific accumulation of related minerals. Aquat Toxicol. 2015; 165: 120-8. doi: 10.1016/j.aquatox.2015.05.013 PMID: 000359030300013
102. Kerambrun E, Henry F, Perrichon P, Courcot L, Meziane T, Spilmont N, et al. Growth and condition indices of juvenile turbot, *Scophthalmus maximus*, exposed to contaminated sediments: Effects of metallic and organic compounds. Aquat Toxicol. 2012; 108: 130-40. doi:10.1016/j.aquatox.2011.07.016
103. Jeffree RA, Warnau M, Oberhansli F, Teyssie J-L. Bioaccumulation of heavy metals and radionuclides from seawater by encased embryos of the spotted dogfish *Scyliorhinus canicula*. Mar Pollut Bull. 2006; 52: 1278-86. doi: 10.1016/j.marpolbul.2006.03.015 PMID: 000241461700025
104. Jeffree RA, Warnau M, Teyssie J-L, Markich SJ. Comparison of the bioaccumulation from seawater and depuration of heavy metals and radionuclides in the spotted dogfish *Scyliorhinus canicula* (Chondrichthys) and the turbot *Psetta maxima* (Actinopterygii: Teleostei). Sci Total Environ. 2006; 368: 839-52. doi: 10.1016/j.scitotenv.2006.03.026 PMID: 000240270700035
105. Ky Trung L, Fotedar R. Toxic effects of excessive levels of dietary selenium in juvenile yellowtail kingfish (*Seriola lalandi*). Aquacult. 2014; 433: 229-34. doi: 10.1016/j.aquaculture.2014.06.021 PMID: 000342529400033
106. Costa PM, Caeiro S, Diniz MS, Lobo J, Martins M, Ferreira AM, et al. Biochemical endpoints on juvenile *Solea senegalensis* exposed to estuarine sediments: the effect of contaminant mixtures on metallothionein and CYP1A induction. Ecotoxicol. 2009; 18: 988-1000. doi: 10.1007/s10646-009-0373-7 PMID: 000269917200004
107. Benhamed S, Guardiola FA, Martínez S, Martínez-Sánchez MJ, Pérez-Sirvent C, Mars M, et al. Exposure of the gilthead seabream (*Sparus aurata*) to sediments contaminated with heavy metals down-regulates the gene expression of stress biomarkers. Toxicol Rep. 2016; 3: 364-72. doi:10.1016/j.toxrep.2016.02.006
108. Costa PM, Repolho T, Caeiro S, Diniz ME, Moura I, Costa MH. Modelling metallothionein induction in the liver of *Sparus aurata* exposed to metal-contaminated sediments. Ecotoxicol Environ Saf. 2008; 71: 117-24. doi: 10.1016/j.ecoenv.2007.05.012 PMID: 000258550400014
109. Isani G, Andreani G, Carpene E, Di Molfetta S, Eletto D, Spisni E. Effects of waterborne Cu exposure in gilthead sea bream (*Sparus aurata*): A proteomic approach. Fish Shellfish Immun. 2011; 31: 1051-8. doi: 10.1016/j.fsi.2011.09.005 PMID: 000298569700041
110. Isani G, Andreani G, Cocchioni F, Fedeli D, Carpene E, Falcioni G. Cadmium accumulation and biochemical responses in *Sparus aurata* following sub-lethal Cd exposure. Ecotoxicol Environ Saf. 2009; 72: 224-30. doi: 10.1016/j.ecoenv.2008.04.015 PMID: 000260660100028
111. Minghetti M, Leaver MJ, Carpene E, George SG. Copper transporter 1, metallothionein and glutathione reductase genes are differentially expressed in tissues of sea bream (*Sparus aurata*) after exposure to dietary or waterborne copper. Comp Biochem Phys C. 2008; 147: 450-9. doi: 10.1016/j.cbpc.2008.01.014 PMID: 18304880
112. Eyckmans M, Lardon I, Wood CM, De Boeck G. Physiological effects of waterborne lead exposure in spiny dogfish (*Squalus acanthias*). Aquat Toxicol. 2013; 126: 373-81. doi: 10.1016/j.aquatox.2012.09.004 PMID: 000315125600040
113. Liu XJ, Luo Z, Li CH, Xiong BX, Zhao YH, Li XD. Antioxidant responses, hepatic intermediary metabolism, histology and ultrastructure in *Synechogobius hasta* exposed to waterborne cadmium. Ecotoxicol Environ Saf. 2011; 74: 1156-63. doi: 10.1016/j.ecoenv.2011.02.015 PMID: 000291960600007
114. Dang F, Wang W-X. Assessment of tissue-specific accumulation and effects of cadmium in a marine fish fed contaminated commercially produced diet. Aquat Toxicol. 2009; 95: 248-55. doi: 10.1016/j.aquatox.2009.09.013 PMID: 000272784900009
115. Alquezar R, Markich SJ, Twining JR. Comparative accumulation of Cd-109 and Se-75 from water and food by an estuarine fish (*Tetractenos glaber*). J Environ Radioactiv. 2008; 99: 167-80. doi: 10.1016/j.jenvrad.2007.07.012 PMID: 000253999900015
