# Supplementary material for: A protocol for identifying suitable biomarkers to assess fish health: A systematic review
Source: PLoS One. 2017 Apr 12;12(4):e0174762. doi: 10.1371/journal.pone.0174762 (PMC5389625; doi:10.1371/journal.pone.0174762)
Supplement: S14 Table — (DOCX) [file pone.0174762.s014.docx]

**S14 Table. Field and laboratory studies on responses of biomarkers of exposure in fish to metals and other contaminants: biotransformation enzymes, Phase I.** Most studies measured contaminants in the environment in addition to those identified as of concern for Gladstone Harbour (Al, Cd, Cu, Ga, Pb, Se, Zn); these are also presented for completeness.

| Species | LHS | Tissue | Method | Laboratory or Field | Metals | other contaminants | cyt P450 | CYP1A | EROD | Others | Reference |
| --- | --- | --- | --- | --- | --- | --- | --- | --- | --- | --- | --- |
| *Anguilla anguilla* | J | liver | Bioassay | Caged field sed | As, Cd, Cr , Cu, Fe, Hg, Mn, Ni, Pb, V, Zn | PAHs |  |  | + |  | [1] |
|  |  |  |  | Lab field sed | As, Cd, Cr, Cu, Fe, Hg, Mn, Ni, Pb, V, Zn | PAHs |  |  | + |  | [1] |
|  |  |  |  | Lab field sed | As, Cd, Cr, Cu, Hg, Ni, Pb, V, Zn | PAH |  |  | + |  | [2] |
| *Aphanius fasciatus* | A | gonads | Real time PCR | Field water and sed | Cd, Cu, Zn | PAHs |  | = |  |  | [3] |
| *Atherina presbyter* | A | liver | Bioassay | Field sed | Cd, Hg, Ni, Pb, Zn | PAHs |  |  | +/- |  | [4] |
| *Dicentrarchus labrax* | A | liver | Bioassay | Caged field sed | Cu, Pb, Zn | PAHs |  |  | = |  | [5] |
|  |  |  | Bioassay | Field sed | Cr, Cu, Ni, Pb, Zn | PAHs |  |  | +/- |  | [6] |
|  | J | liver | Bioassay | Caged field sed | Cd, Cr, Cu, Ni, Pb, Zn |  |  |  | + |  | [7] |
| *Gadus morhua L.* | A | liver | Bioassay | Cage field water | Cd, Cu, Hg, Pb, Zn | PAHs, PCBs |  | +/- | +/- |  | [8] |
| *Lates calcarifer* | A | liver | Bioassay | Field sed | Cd, Cr, Cu, Ni, Zn | Diuron, PAHs | = |  | + |  | [9] |
| *Mullus barbatus* | A | liver | Bioassay | Field sed | As, Cr, Cu, Ni, Pb, Zn | PAHs, CBs, DDT, HCB, trans-nonachlor, Lindane, Dieldrin |  |  | + |  | [10] |
| *Plastichthys flesus* | A | liver | Bioassay | Cage field water | Cd, Cu, Hg, Pb, Zn | PAHs, PCBs |  | + | +/- |  | [8] |
|  |  |  |  | Field sed | Cd, Hg, Pb, Zn | PAHs, PCBs |  | = | = |  | [11] |
|  |  |  |  | Field sed | Cd, Cu, Hg, Pb | PCBs |  |  | + |  | [12] |
|  |  |  |  | Field water and sed | As, Cd, Cr, Cu, Hg, Ni, Pb, Zn | PAHs, PCBs, OCPs |  | +/- |  |  | [13] |
|  |  |  | mRNA absorbance | Field sed | Cd, Hg, Pb, Zn | PAHs, PCBs |  | + F |  |  | [11] |
| *Pomatoschistus microps* | A | liver | Bioassay | Field sed | Cd, Cr, Cu, Hg, Ni, Pb, Zn | PAHs |  |  | + |  | [14] |
|  |  |  | Bioassay | Field sed | Cd, Hg, Ni, Pb, Zn |  |  |  | +/- |  | [4] |
|  |  |  | Bioassay | Field sed | Cr, Cu, Ni, Pb, Zn | PAHs |  |  | = |  | [6] |
| *Scophthalmus maximus* | J | liver | Bioassay | Caged field sed | Cd, Cr, Cu, Ni, Pb, Zn |  |  |  | + |  | [7] |
|  |  |  | Bioassay | Lab field sed | Cd, Cd, Cr, Cu, Hg, Ni, Pb, Zn |  |  |  | +/- |  | [15] |
|  |  |  | western blot | Lab field sed | Cu, Pb, Zn |  |  | + | + | BROD =; MROD +; PROD + | [16] |
| *Solea senegalensis* | A | gills | Immunohistochemical /bioassay | Field water and sed | As, Cd, Cu, Fe, Pb, Zn | PAHs |  | = | - |  | [17] |
|  | A | liver | Bioassay | Field sed | Cr, Cu, Ni, Pb, Zn | PAHs |  |  | = |  | [6] |
|  |  |  | Immunohistochemical / bioassay | Field water and sed | As, Cd, Cu, Fe, Pb, Zn | PAHs |  | = | + |  | [17] |
|  | J | liver | Bioassay | Field sed | Cd, Cr, Cu, Ni, Pb, Zn | PAHs |  |  | = |  | [18] |
|  |  |  |  | Lab and field sed | As, Cu, Zn | PAHs, PCBs, DDT |  | + |  |  | [19] |
|  |  |  |  | Lab field sed | As, Cd, Cr, Cu, Hg, Ni, Pb, Zn | PAHs |  |  | + |  | [20] |
|  |  |  |  | Lab field sed | As, Cd, Cr, Cu, Ni, Pb, Zn | PAHs, PCBs, DDT |  | - |  |  | [21] |
| *Sparus aurata* | A | liver | Real time PCR | Lab and field sed | As, Cd, Cu, Pb, Zn, |  |  | + |  |  | [22] |
|  | A | skin | Real time PCR | Lab and field sed | As, Cd, Cu, Pb, Zn, |  |  | = |  |  | [22] |
|  | J | liver | Bioassay | Lab field sed | As, Cd, Cr, Cu, Hg, Ni, Pb, Zn | PAHs |  |  | + |  | [20] |
|  | J | liver | Real time PCR | Lab field sed | As, Cd, Cr, Cu, Hg, Ni, Pb, Se, V, Zn | PAHs |  |  |  | CYP3A + | [23] |
| *Symphodus melops* | A | liver | Bioassay | Field water and sed | Fe, Pb, Zn |  |  |  | = |  | [24] |

Abbreviations: LHS: life history stage; J: juveniles, A: adults; Lab: laboratory; Sed : sediment; HCB: hexachlorobenzene; OCP: total organochlorine pesticides; CB: chlorinated biphenyls; naph: naphthalenes; PAHs: total polycyclic aromatic hydrocarbons; PCBS: polychlorinated biphenyl; HCH:hexachlorcyclohexane; DDT: dichlorodiphenyltrichloroethane; HCB: hexachlorobenzene; cyt P450: total cytochrome P450; CYP1A: Cytochrome P450 family 1 subfamily A; EROD: 7-ethoxyresorufin O-deethoxylase; + induction; - inhibition; +/- mixed response; = no significant induction; F: Female only; BROD: 7-benzyloxyresorufin O-debenzylase; MROD: 7-methooxyresorufin O-methoxyresorufin; PROD: 7-pentooxyresorufin O-depentylase; AHH: aryl hydrocarbon hydroxylase; CYP3A: Cytochrome P450 family 3 subfamily A.

# References

1. Piva F, Ciaprini F, Onorati F, Benedetti M, Fattorini D, Ausili A, et al. Assessing sediment hazard through a weight of evidence approach with bioindicator organisms: a practical model to elaborate data from sediment chemistry, bioavailability, biomarkers and ecotoxicological bioassays. Chemosphere. 2011; 83: 475-85. doi: 10.1016/j.chemosphere.2010.12.064 PMID: 21239037
2. Benedetti M, Ciaprini F, Piva F, Onorati F, Fattorini D, Notti A, et al. A multidisciplinary weight of evidence approach for classifying polluted sediments: Integrating sediment chemistry, bioavailability, biomarkers responses and bioassays. Environ Int. 2012; 38: 17-28. doi: 10.1016/j.envint.2011.08.003 PMID: 21982029
3. Annabi A, Kessabi K, Navarro A, Said K, Messaoudi I, Pina B. Assessment of reproductive stress in natural populations of the fish *Aphanius fasciatus* using quantitative mRNA markers. Aquat Biol. 2012; 17: 285-+. doi: 10.3354/ab00482 PMID: 000312247800008
4. Fonseca VF, Vasconcelos RP, Franca S, Serafim A, Lopes B, Company R, et al. Modeling fish biological responses to contaminants and natural variability in estuaries. Mar Environ Res. 2014; 96: 45-55. doi: 10.1016/j.marenvres.2013.10.011 PMID: 000334981600007
5. Traven L, Micovic V, Lusic DV, Smital T. The responses of the hepatosomatic index (HSI), 7-ethoxyresorufin-O-deethylase (EROD) activity and glutathione-S-transferase (GST) activity in sea bass (*Dicentrarchus labrax*, Linnaeus 1758) caged at a polluted site: implications for their use in environmental risk assessment. Environ Monitor Ass. 2013; 185: 9009-18. doi: 10.1007/s10661-013-3230-3 PMID: 000325116500018
6. Fonseca VF, Franca S, Serafim A, Company R, Lopes B, Bebianno MJ, et al. Multi-biomarker responses to estuarine habitat contamination in three fish species: *Dicentrarchus labrax*, *Solea senegalensis* and *Pomatoschistus microps*. Aquat Toxicol. 2011; 102: 216-27. doi: 10.1016/j.aquatox.2011.01.018 PMID: 21356184
7. Kerambrun E, Sanchez W, Henry F, Amara R. Are biochemical biomarker responses related to physiological performance of juvenile sea bass (*Dicentrarchus labrax*) and turbot (*Scophthalmus maximus*) caged in a polluted harbour? Comp Biochem Phys C. 2011; 154: 187-95. doi: 10.1016/j.cbpc.2011.05.006 PMID: 000293994200007
8. Beyer J, Sandvik M, Hylland K, Fjeld E, Egaas E, Aas E, et al. Contaminant accumulation and biomarker responses in flounder (*Platichthys flesus* L) and Atlantic cod (*Gadus morhua* L) exposed by caging to polluted sediments in Sorfjorden, Norway. Aquat Toxicol. 1996; 36: 75-98. doi: 10.1016/s0166-445x(96)00798-9 PMID: A1996VY98200005
9. Humphrey CA, King SC, Klumpp DW. A multibiomarker approach in barramundi (*Lates calcarifer*) to measure exposure to contaminants in estuaries of tropical North Queensland. Mar Pollut Bull. 2007; 54: 1569-81. doi: 10.1016/j.marpolbul.2007.06.004 PMID: 000250599700014
10. Martinez-Gomez C, Fernandez B, Benedicto J, Valdes J, Campillo JA, Leon VM, et al. Health status of red mullets from polluted areas of the Spanish Mediterranean coast, with special reference to Portman (SE Spain). Mar Environ Res. 2012; 77: 50-9. doi: 10.1016/j.marenvres.2012.02.002 PMID: 000304296700008
11. Vethaak AD, Jol JG, Meijboom A, Eggens ML, apRheinallt T, Wester PW, et al. Skin and liver diseases induced in flounder (*Platichthys flesus*) after long-term exposure to contaminated sediments in large-scale mesocosms. Environ Health Persp. 1996; 104: 1218-29. doi: 10.2307/3432916 PMID: A1996VX74000021
12. Schmidt V, Zander S, Korting W, Broeg K, von Westernhagen H, Dizer H, et al. Parasites of flounder (*Platichthys flesus* L.) from the German Bight, North Sea, and their potential use in biological effects monitoring - C. Pollution effects on the parasite community and a comparison to biomarker responses. Helgoland Mar Res. 2003; 57: 262-71. doi: 10.1007/s10152-003-0159-x PMID: 000186604600015
13. Schipper CA, Lahr J, van den Brink PJ, George SG, Hansen P-D, de Assis HCdS, et al. A retrospective analysis to explore the applicability of fish biomarkers and sediment bioassays along contaminated salinity transects. Ices J Mar Sci. 2009; 66: 2089-105. doi: 10.1093/icesjms/fsp194 PMID: 000272080600003
14. Serafim A, Company R, Lopes B, Fonseca VF, Franca S, Vasconcelos RP, et al. Application of an integrated biomarker response index (IBR) to assess temporal variation of environmental quality in two Portuguese aquatic systems. Ecol Indic. 2012; 19: 215-25. doi: 10.1016/j.ecolind.2011.08.009 PMID: 000302891100022
15. Kerambrun E, Henry F, Marechal A, Sanchez W, Minier C, Filipuci I, et al. A multibiomarker approach in juvenile turbot, *Scophthalmus maximus*, exposed to contaminated sediments. Ecotoxicol Environ Saf. 2012; 80: 45-53. doi: 10.1016/j.ecoenv.2012.02.010 PMID: 000304337300007
16. Hartl MGJ, Kilemade M, Sheehan D, Mothersill C, O'Halloran J, O'Brien NM, et al. Hepatic biomarkers of sediment-associated pollution in juvenile turbot, *Scophthalmus maximus* L. Mar Environ Res. 2007; 64: 191-208. doi: 10.1016/j.marenvres.2007.01.002 PMID: 000248488500007
17. Oliva M, Gravato C, Guilhermino L, Dolores Galindo-Riano M, Antonio Perales J. EROD activity and cytochrome P4501A induction in liver and gills of Senegal sole *Solea senegalensis* from a polluted Huelva Estuary (SW Spain). Comp Biochem Phys C. 2014; 166: 134-44. doi: 10.1016/j.cbpc.2014.07.010 PMID: 000342532000015
18. Fonseca VF, Vasconcelos RP, Tanner SE, Franca S, Serafim A, Lopes B, et al. Habitat quality of estuarine nursery grounds: Integrating non-biological indicators and multilevel biological responses in *Solea senegalensis*. Ecol Indic. 2015; 58: 335-45. doi: 10.1016/j.ecolind.2015.05.064 PMID: 000360776100035
19. Costa PM, Caeiro S, Vale C, Angel DelValls T, Costa MH. Can the integration of multiple biomarkers and sediment geochemistry aid solving the complexity of sediment risk assessment? A case study with a benthic fish. Environ Pollut. 2012; 161: 107-20. doi: 10.1016/j.envpol.2011.10.010 PMID: 000300539300016
20. Jimenez-Tenorio N, Morales-Caselles C, Kalman J, Salamanca MJ, Luisa Gonzalez de Canales M, Sarasquete C, et al. Determining sediment quality for regulatory proposes using fish chronic bioassays. Environ Internat. 2007; 33: 474-80. doi: 10.1016/j.envint.2006.11.009 PMID: 000246315800008
21. Costa PM, Caeiro S, Diniz MS, Lobo J, Martins M, Ferreira AM, et al. Biochemical endpoints on juvenile *Solea senegalensis* exposed to estuarine sediments: the effect of contaminant mixtures on metallothionein and CYP1A induction. Ecotoxicol. 2009; 18: 988-1000. doi: 10.1007/s10646-009-0373-7 PMID: 000269917200004
22. Benhamed S, Guardiola FA, Martínez S, Martínez-Sánchez MJ, Pérez-Sirvent C, Mars M, et al. Exposure of the gilthead seabream (*Sparus aurata*) to sediments contaminated with heavy metals down-regulates the gene expression of stress biomarkers. Toxicol Rep. 2016; 3: 364-72. doi:10.1016/j.toxrep.2016.02.006
23. Ribecco C, Baker ME, Sasik R, Zuo Y, Hardiman G, Carnevali O. Biological effects of marine contaminated sediments on *Sparus aurata* juveniles. Aquat Toxicol. 2011; 104: 308-16. doi: 10.1016/j.aquatox.2011.05.005 PMID: 000293042100017
24. Almroth BC, Sturve J, Stephensen E, Holth TF, Forlin L. Protein carbonyls and antioxidant defenses in corkwing wrasse (*Symphodus melops*) from a heavy metal polluted and a PAH polluted site. Mar Environ Res. 2008; 66: 271-7. doi: 10.1016/j.marenvres.2008.04.002 PMID: 000257817100006
