# Supplementary material for: A protocol for identifying suitable biomarkers to assess fish health: A systematic review
Source: PLoS One. 2017 Apr 12;12(4):e0174762. doi: 10.1371/journal.pone.0174762 (PMC5389625; doi:10.1371/journal.pone.0174762)
Supplement: S15 Table — (DOCX) [file pone.0174762.s015.docx]

**S15 Table. Field and laboratory studies on responses of biomarkers of exposure in fish to metals and other contaminants: biotransformation enzymes, Phase II.** Most studies measured contaminants in the environment in addition to those identified as of concern for Gladstone Harbour (Al, Cd, Cu, Ga, Pb, Se, Zn); these are also presented for completeness.

| Species | LHS | Tissue | Method | Laboratory or Field | Metals | other contaminants | GSH / GSSH | GST | Total Glutathione | Reference |
| --- | --- | --- | --- | --- | --- | --- | --- | --- | --- | --- |
| *Acanthopagrus latus* | J | gills | Bio-assay | Field sed | As, Cr, Cu, Ni, Pb, V, Zn | PAHs |  | = |  | [1] |
|  |  | liver | Bio-assay | Field sed | As, Cr, Cu, Ni, Pb, V, Zn | PAHs |  | = |  | [1] |
| *Anguilla anguilla* | Glass eels | liver | Bio-assay | Field sed | Cd, Cr, Cu, Hg, Ni, Pb, V, Zn | PAH | = | - | - | [2] |
|  | J | liver | Bio-assay | Caged field sed | As, Cd, Cr, Cu, Fe, Hg, Mn, Ni, Pb, V, Zn | PAHs |  | + | + | [3] |
|  |  |  | Bio-assay | Lab field sed | As, Cd, Cr, Cu, Fe, Hg, Mn, Ni, Pb, V, Zn | PAHs |  | = | - | [3] |
|  |  |  | Bio-assay | Lab field sed toxicity | As, Cd, Cr, Cu, Hg, Ni, Pb, V, Zn | PAH |  | + | - | [4] |
|  | Yellow eels | liver | Bio-assay | Field sed | Cd, Cr, Cu, Hg, Ni, Pb, V, Zn | PAH | - | + | - | [2] |
| *Atherina presbyter* | J | liver | Bio-assay | Field sed | Cd, Hg, Ni, Pb, Zn | PAHs |  | +/- |  | [5] |
| *Centropomus parallelus* | J | gills | Bio-assay | Field sed and water | Ag, Al, As, Cd, Cr, Cu, Fe, Hg, Mn, Ni, Pb, Se, Zn |  |  | - |  | [6] |
|  |  | liver | Bio-assay | Field sed and water | Ag, Al, As, Cd, Cr, Cu, Fe, Hg, Mn, Ni, Pb, Se, Zn |  |  | - |  | [6] |
| *Cynoglossus arel* | J | gills | Bio-assay | Field sed | As, Cr, Cu, Ni, Pb, V, Zn | PAHs |  | = |  | [1] |
|  |  | liver | Bio-assay | Field sed | As, Cr, Cu, Ni, Pb, V, Zn | PAHs |  | + |  | [1] |
| *Dicentrarchus labrax* | J | liver | Bio-assay | Caged field sed | Cu, Pb, Zn | PAHs |  | - |  | [7] |
|  |  |  | Bio-assay | Field sed | Cr, Cu, Ni, Pb, Zn | PAHs |  | = |  | [8] |
|  |  |  | Bio-assay | Lab water toxicity test | Cu antifouling |  |  | = |  | [9] |
|  | J | liver | Bio-assay | Caged field sed | Cd, Cr, Cu, Ni, Pb, Zn |  |  | + |  | [10] |
| *Gadus morhua L.* | J | liver | Bio-assay | Cage field water | Cd, Cu, Hg, Pb, Zn | PAHs, PCBs |  | +/- |  | [11] |
| *Liza aurata* | J | gills | Bio-assay | Field water | Cd, Cr, Cu, Mn, Ni, Pb |  |  | + | + | [12] |
|  |  | kidney | Bio-assay | Field water | Cd, Cr, Cu, Mn, Ni, Pb |  |  | + | = | [13] |
|  |  | liver | Bio-assay | Field water | Cd, Cr, Cu, Mn, Ni, Pb |  |  | = | - | [13] |
| *Lutjanus russellii* | J | blood | Bio-assay | Field sed and water | Cd, Cu, Fe, Pb, Zn |  |  | + |  | [14] |
| *Mugil cephalus* | J | liver | Bio-assay | Field water | Cd, Cr, Cu, Fe, Hg, Mn, Ni, Pb, Se, Zn |  | - | - |  | [15] |
|  | J | gill | Bio-assay | Lab water toxicity test | Pb |  | + | - |  | [16] |
|  |  | whole | Bio-assay | Lab water toxicity test | Pb |  | + | - |  | [16] |
|  |  | liver | Bio-assay | Field sed and water | Cd, Cu, Mn, Ni, Pb | AHCs, PAHs, PCBs, DDTs, TBT |  | +/- |  | [17] |
| *Paralichthys olivaceus* | J | gill | Bio-assay | Lab water toxicity test | Cd |  | - | - |  | [18] |
|  |  | kidney | Bio-assay | Lab water toxicity test | Cd |  | = | + |  | [18] |
|  |  | liver | Bio-assay | Lab water toxicity test | Cd |  | - | - |  | [18] |
|  |  | whole | Bio-assay | Lab water toxicity test | Cd |  | = | - |  | [19] |
|  | L meta | whole | Bio-assay | Lab water toxicity test | Cd (≥12µg L-) |  | + | = |  | [19] |
|  | L set | whole | Bio-assay | Lab water toxicity test | Cd (48 µg L-) |  | + | - |  | [19] |
| *Plastichthys flesus* | J | liver | Bio-assay | Cage field water | Cd, Cu, Hg, Pb, Zn | PAHs, PCBs |  | +/- |  | [11] |
|  |  |  | Bio-assay | Field water and sed | As, Cd, Cr, Cu, Hg, Ni, Pb, Zn | PAHs, PCBs, OCPs |  | +/- |  | [20] |
| *Poecilia vivipara acclimated to saltwater* | J | gills | Bio-assay | Lab water toxicity test | Cu |  |  | = |  | [21] |
|  |  | liver | Bio-assay | Lab water toxicity test | Cu |  |  | +/- |  | [21] |
|  |  | muscle | Bio-assay | Lab water toxicity test | Cu |  |  | +/- |  | [21] |
| *Pomadasys hasta* | J | blood | Bio-assay | Field sed and water | Cd, Cu, Fe, Pb, Zn |  |  | + |  | [14] |
| *Pomatoschistus microps* | J | liver | Bio-assay | Field sed | Cd, Cr, Cu, Hg, Ni, Pb, Zn | PAHs |  | = |  | [22] |
|  |  |  | Bio-assay | Field sed | Cd, Hg, Ni, Pb, Zn | PAHS |  | +/- |  | [5] |
|  |  |  | Bio-assay | Field sed | Cr, Cu, Ni, Pb, Zn | PAHs |  | = |  | [8] |
| *Scophthalmus maximus* | J | liver | Bio-assay | Caged field sed | Cd, Cr, Cu, Ni, Pb, Zn |  |  | + |  | [10] |
|  |  |  | Bio-assay | Lab field sed toxicity | Cd, Cr, Cu, Mn, Ni, Pb, V, Zn |  |  | +/- |  | [23] |
| *Solea senegalensis* | J | liver | Bio-assay | Field sed | Cr, Cu, Ni, Pb, Zn | PAHs |  | - |  | [8] |
|  |  |  | Bio-assay | Field water and sed | As, Cd, Cu, Fe, Pb, Zn | PAHs |  | = |  | [24] |
|  | J | liver | Bio-assay | Field sed | Cd, Cr, Cu, Ni, Pb, Zn | PAHs |  | = |  | [25] |
| *Symphodus melops* | J | liver/blood | Bio-assay | Field water and sed | Fe, Pb, Zn |  | = | = |  | [26] |
| *Terapon jarbua* | J | gill | Bio-assay | Lab water toxicity test | Pb |  | + | - |  | [16] |
|  |  | whole | Bio-assay | Lab water toxicity test | Pb |  | + | - |  | [16] |

Abbreviations: LHS: life history stage; A: adult, J = juvenile, L = larvae; Lab: laboratory; meta: metamorphosing; set: settling; Sed: sediment; AHCs: aliphatic hydrocarbons; OCP: total organochlorine pesticides; PAHs: total polycyclic aromatic hydrocarbons; PCBS: polychlorinated biphenyl; TBT: tributyltin; DDT: dichlorodiphenyltrichloroethane; GSH/GSSH: reduced and oxidised glutathione GST: Glutathione S-transferases; + induction; - inhibition; = no significant induction; +/- mixed response.

# References

1. Beg MU, Al-Jandal N, Al-Subiai S, Karam Q, Husain S, Butt SA, et al. Metallothionein, oxidative stress and trace metals in gills and liver of demersal and pelagic fish species from Kuwaits’ marine area. Mar Pollut Bull. 2015; 100: 662-72. doi: 10.1016/j.marpolbul.2015.07.058
2. Gravato C, Guimaraes L, Santos J, Faria M, Alves A, Guilhermino L. Comparative study about the effects of pollution on glass and yellow eels (*Anguilla anguilla*) from the estuaries of Minho, Lima and Douro Rivers (NW Portugal). Ecotoxicol Environ Saf. 2010; 73: 524-33. doi: 10.1016/j.ecoenv.2009.11.009 PMID: 000277103600009
3. Piva F, Ciaprini F, Onorati F, Benedetti M, Fattorini D, Ausili A, et al. Assessing sediment hazard through a weight of evidence approach with bioindicator organisms: a practical model to elaborate data from sediment chemistry, bioavailability, biomarkers and ecotoxicological bioassays. Chemosphere. 2011; 83: 475-85. doi: 10.1016/j.chemosphere.2010.12.064 PMID: 21239037
4. Benedetti M, Ciaprini F, Piva F, Onorati F, Fattorini D, Notti A, et al. A multidisciplinary weight of evidence approach for classifying polluted sediments: Integrating sediment chemistry, bioavailability, biomarkers responses and bioassays. Environ Int. 2012; 38: 17-28. doi: 10.1016/j.envint.2011.08.003 PMID: 21982029
5. Fonseca VF, Vasconcelos RP, Franca S, Serafim A, Lopes B, Company R, et al. Modeling fish biological responses to contaminants and natural variability in estuaries. Mar Environ Res. 2014; 96: 45-55. doi: 10.1016/j.marenvres.2013.10.011 PMID: 000334981600007
6. Souza IC, Duarte ID, Pimentel NQ, Rocha LD, Morozesk M, Bonomo MM, et al. Matching metal pollution with bioavailability, bioaccumulation and biomarkers response in fish (*Centropomus parallelus*) resident in neotropical estuaries. Environ Pollut. 2013; 180: 136-44. doi: 10.1016/j.envpol.2013.05.017 PMID: 000322425300019
7. Traven L, Micovic V, Lusic DV, Smital T. The responses of the hepatosomatic index (HSI), 7-ethoxyresorufin-O-deethylase (EROD) activity and glutathione-S-transferase (GST) activity in sea bass (*Dicentrarchus labrax*, Linnaeus 1758) caged at a polluted site: implications for their use in environmental risk assessment. Environ Monitor Ass. 2013; 185: 9009-18. doi: 10.1007/s10661-013-3230-3 PMID: 000325116500018
8. Fonseca VF, Franca S, Serafim A, Company R, Lopes B, Bebianno MJ, et al. Multi-biomarker responses to estuarine habitat contamination in three fish species: *Dicentrarchus labrax*, *Solea senegalensis* and *Pomatoschistus microps*. Aquat Toxicol. 2011; 102: 216-27. doi: 10.1016/j.aquatox.2011.01.018 PMID: 21356184
9. Cotou E, Henry M, Zeri C, Rigos G, Torreblanca A, Catsiki V-A. Short-term exposure of the European sea bass *Dicentrarchus labrax* to copper-based antifouling treated nets: Copper bioavailability and biomarkers responses. Chemosphere. 2012; 89: 1091-7. doi:10.1016/j.chemosphere.2012.05.075
10. Kerambrun E, Sanchez W, Henry F, Amara R. Are biochemical biomarker responses related to physiological performance of juvenile sea bass (*Dicentrarchus labrax*) and turbot (*Scophthalmus maximus*) caged in a polluted harbour? Comp Biochem Phys C. 2011; 154: 187-95. doi: 10.1016/j.cbpc.2011.05.006 PMID: 000293994200007
11. Beyer J, Sandvik M, Hylland K, Fjeld E, Egaas E, Aas E, et al. Contaminant accumulation and biomarker responses in flounder (*Platichthys flesus* L) and Atlantic cod (*Gadus morhua* L) exposed by caging to polluted sediments in Sorfjorden, Norway. Aquat Toxicol. 1996; 36: 75-98. doi: 10.1016/s0166-445x(96)00798-9 PMID: A1996VY98200005
12. Pereira P, de Pablo H, Vale C, Pacheco M. Combined use of environmental data and biomarkers in fish (*Liza aurata*) inhabiting a eutrophic and metal-contaminated coastal system - Gills reflect environmental contamination. Mar Environ Res. 2010; 69: 53-62. doi: 10.1016/j.marenvres.2009.08.003 PMID: 000274773800001
13. Pereira P, de Pablo H, Pacheco M, Vale C. The relevance of temporal and organ specific factors on metals accumulation and biochemical effects in feral fish (*Liza aurata*) under a moderate contamination scenario. Ecotoxicol Environ Saf. 2010; 73: 805-16. doi: 10.1016/j.ecoenv.2010.02.020 PMID: 000279623800015
14. Omar WA, Saleh YS, Marie M-AS. The use of biotic and abiotic components of Red Sea coastal areas as indicators of ecosystem health. Ecotoxicol. 2016; 25: 253-66. doi: 10.1007/s10646-015-1584-8 PMID: 000370716000001
15. Padmini E, Rani MU. Evaluation of oxidative stress biomarkers in hepatocytes of grey mullet inhabiting natural and polluted estuaries. Sci Total Environ. 2009; 407: 4533-41. doi: 10.1016/j.scitotenv.2009.04.005 PMID: 000267631700019
16. Hariharan G, Purvaja R, Ramesh R. Environmental safety level of lead (Pb) pertaining to toxic effects on grey mullet (*Mugil cephalus*) and Tiger perch (*Terapon jarbua*). Environ Toxicol. 2016; 31: 24-43. doi: 10.1002/tox.22019 PMID: 000366585300003
17. Tsangaris C, Vergolyas M, Fountoulaki E, Nizheradze K. Oxidative Stress and Genotoxicity Biomarker Responses in Grey Mullet (*Mugil cephalus*) From a Polluted Environment in Saronikos Gulf, Greece. Arch Environ Con Tox. 2011; 61: 482-90. doi: 10.1007/s00244-010-9629-8 PMID: 000298500400013
18. Cao L, Huang W, Shan X, Ye Z, Dou S. Tissue-specific accumulation of cadmium and its effects on antioxidative responses in Japanese flounder juveniles. Environ Toxicol Pharmacol. 2012; 33: 16-25. doi: 10.1016/j.etap.2011.10.003 PMID: 000301876600003
19. Cao L, Huang W, Liu J, Yin X, Dou S. Accumulation and oxidative stress biomarkers in Japanese flounder larvae and juveniles under chronic cadmium exposure. Comp Biochem Phys C. 2010; 151: 386-92. doi: 10.1016/j.cbpc.2010.01.004 PMID: 000275627400016
20. Schipper CA, Lahr J, van den Brink PJ, George SG, Hansen P-D, de Assis HCdS, et al. A retrospective analysis to explore the applicability of fish biomarkers and sediment bioassays along contaminated salinity transects. Ices J Mar Sci. 2009; 66: 2089-105. doi: 10.1093/icesjms/fsp194 PMID: 000272080600003
21. de Souza Machado AA, Mueller Hoff ML, Klein RD, Cardozo JG, Giacomin MM, Ledes Pinho GL, et al. Biomarkers of waterborne copper exposure in the guppy *Poecilia vivipara* acclimated to salt water. Aquat Toxicol. 2013; 138: 60-9. doi: 10.1016/j.aquatox.2013.04.009. PMID: 000322293600007
22. Serafim A, Company R, Lopes B, Fonseca VF, Franca S, Vasconcelos RP, et al. Application of an integrated biomarker response index (IBR) to assess temporal variation of environmental quality in two Portuguese aquatic systems. Ecol Indic. 2012; 19: 215-25. doi: 10.1016/j.ecolind.2011.08.009 PMID: 000302891100022
23. Kerambrun E, Henry F, Marechal A, Sanchez W, Minier C, Filipuci I, et al. A multibiomarker approach in juvenile turbot, *Scophthalmus maximus*, exposed to contaminated sediments. Ecotoxicol Environ Saf. 2012; 80: 45-53. doi: 10.1016/j.ecoenv.2012.02.010 PMID: 000304337300007
24. Oliva M, Jose Vicente J, Gravato C, Guilhermino L, Dolores Galindo-Riano M. Oxidative stress biomarkers in Senegal sole, *Solea senegalensis*, to assess the impact of heavy metal pollution in a Huelva estuary (SW Spain): Seasonal and spatial variation. Ecotoxicol Environ Saf. 2012; 75: 151-62. doi: 10.1016/j.ecoenv.2011.08.017 PMID: 000297088500020
25. Fonseca VF, Vasconcelos RP, Tanner SE, Franca S, Serafim A, Lopes B, et al. Habitat quality of estuarine nursery grounds: Integrating non-biological indicators and multilevel biological responses in *Solea senegalensis*. Ecol Indic. 2015; 58: 335-45. doi: 10.1016/j.ecolind.2015.05.064 PMID: 000360776100035
26. Almroth BC, Sturve J, Stephensen E, Holth TF, Forlin L. Protein carbonyls and antioxidant defenses in corkwing wrasse (*Symphodus melops*) from a heavy metal polluted and a PAH polluted site. Mar Environ Res. 2008; 66: 271-7. doi: 10.1016/j.marenvres.2008.04.002 PMID: 000257817100006
