# Supplementary material for: A protocol for identifying suitable biomarkers to assess fish health: A systematic review
Source: PLoS One. 2017 Apr 12;12(4):e0174762. doi: 10.1371/journal.pone.0174762 (PMC5389625; doi:10.1371/journal.pone.0174762)
Supplement: S16 Table — (DOCX) [file pone.0174762.s016.docx]

**S16 Table. Field and laboratory studies on responses of biomarkers of exposure in fish to metals and other contaminants: oxidative stress parameters.** Most studies measured contaminants in the environment in addition to those identified as of concern for Gladstone Harbour (Al, Cd, Cu, Ga, Pb, Se, Zn); these are also presented for completeness.

| Species | LHS | Tissue | Method | Laboratory or Field | Metals | Other contami-nants | SOD | CAT | GPOX | GRED | LPOX | Se-GPx | ROS | TOSC | Others | Reference |
| --- | --- | --- | --- | --- | --- | --- | --- | --- | --- | --- | --- | --- | --- | --- | --- | --- |
| *Acanthopagrus latus* | A | gills | Bioassay | Field sed | As , Cr , Cu, Ni, Pb, V, Zn | PAHs |  | = | +/- | +/- | + |  |  |  |  | [1] |
|  |  | liver | Bioassay | Field sed | As, Cr, Cu, Ni, Pb, V, Zn | PAHs |  | +/- | +/- | +/- | +/- |  |  |  |  | [1] |
| *Anguilla anguilla* | Glass eels | liver | Bioassay | Field sed | Cd, Cr, Cu, Hg, Ni, Pb, V, Zn | PAH | - | - | = | = | = |  |  |  |  | [2] |
|  | J | liver | Bioassay | Caged field sed | As, Cd, Cr, Cu, Fe, Hg, Mn, Ni, Pb, V, Zn | PAHs |  | + | - |  |  | - |  | = |  | [3] |
|  |  |  |  | Lab field sed | As, Cd, Cr, Cu, Fe, Hg, Mn, Ni, Pb, V, Zn | PAHs |  | - | + |  |  | + |  | +/- | AOX + | [3] |
|  |  |  |  | Lab field sed toxicity | As, Cd, Cr, Cu, Hg, Ni, Pb, V, Zn | PAH |  | + |  | - | + | + |  | - | AOX = | [4] |
|  | Yellow eels | liver | Bioassay | Field sed | Cd, Cr, Cu, Hg, Ni, Pb, V, Zn | PAH | = | - | = | = | + |  |  |  |  | [2] |
| *Aphanius fasciatus* | A | gonads | Real time PCR | Field water and sed | Cd, Cu, Zn | PAHs | = |  |  |  |  |  |  |  |  | [5] |
| *Atherina presbyter* | A | liver | Bioassay | Field sed | Cd, Hg, Ni, Pb, Zn | PAHs | +/- | +/- |  |  | +/- |  |  |  |  | [6] |
| *Centropomus parallelus* | J | gills | Bioassay | Field sed and water | Ag, Al, As, Cd, Cr, Cu, Fe, Hg, Mn, Ni, Pb, Se, Zn |  | - |  |  |  |  |  |  |  |  | [7] |
|  |  | liver | Bioassay | Field sed and water | Ag, Al, As, Cd, Cr, Cu, Fe, Hg, Mn, Ni, Pb, Se, Zn |  | + | +/- |  |  |  |  |  |  |  | [7] |
| *Coris julis* | A | gills | Immuno-histochemical | Field sed | Cd, Co, Cr, Cu, Ni, Pb, Sb, Zn |  |  |  |  |  |  |  |  |  | NOS - | [8] |
| *Cynoglossus arel* | A | gills | Bioassay | Field sed | As, Cr, Cu, Ni, Pb, V, Zn | PAHs |  | = | = | = | +/- |  |  |  |  | [1] |
|  |  | liver | Bioassay | Field sed | As, Cr, Cu, Ni, Pb, V, Zn | PAHs |  | = | +/- | +/- | = |  |  |  |  | [1] |
| *Dicentrarchus labrax* | A | liver | Bioassay | Field sed | Cr, Cu, Ni, Pb, Zn | PAHs | +/- | = | +/- |  | = | = |  |  |  | [9] |
|  |  |  |  | Lab water toxicity test | Cu antifouling |  |  |  |  |  | = |  |  |  |  | [10] |
|  | J | gills | Immuno-histochemical | Lab field sed toxicity | As, Cd, Co, Cr, Cu, Fe, Hg, Mn, Ni, Pb, Sb, V, Zn |  |  |  |  |  |  |  |  |  | NOS + | [11] |
|  |  |  | Real time PCR | Lab field sed toxicity | As, Cd, Cr, Cu, Hg, Ni, Pb, Zn | PAHs and PCBs |  |  |  |  |  |  |  |  | HIF-1 + | [12] |
|  |  | liver | Bioassay | Caged field sed | Cd, Cr, Cu, Ni, Pb, Zn |  |  | - |  |  |  |  |  |  |  | [13] |
| *Diplodus sargus* | A | liver | Bioassay | Field and aquaculture | As, Cd, Cu, Pb |  | + | + |  |  | = |  |  |  |  | [14] |
|  |  | muscle | Bioassay | Field and aquaculture | As, Cd, Cu, Pb |  | + | + |  |  | = |  |  |  |  | [14] |
| *Fundulus heteroclitus* | A | gill | Bioassay | Lab water toxicity test | Cu |  | - | = |  |  |  |  |  |  | CP = | [15] |
|  |  | intestine | Bioassay | Lab water toxicity test | Cu |  | - | + |  |  |  |  |  |  | CP = | [15] |
|  |  | liver | Bioassay | Lab water toxicity test | Cu |  | = | + |  |  |  |  |  |  | CP = | [15] |
| *Limanda limanda* | A | liver | Bioassay | Field sed | Cu, Hg | hydrocarbons | +/- | +/- | - |  |  |  |  |  | DT-diphorase = | [16] |
| *Liza aurata* | J | gills | Bioassay | Field water | Cd, Cr, Cu, Mn, Ni, Pb |  |  | + | + | + | + |  |  |  |  | [17] |
|  |  | kidney | Bioassay | Field water | Cd, Cr, Cu, Mn, Ni, Pb |  |  | + | = | + | = |  |  |  |  | [18] |
|  |  | liver | Bioassay | Field water | Cd, Cr, Cu, Mn, Ni, Pb |  |  | + | + | = | + |  |  |  |  | [18] |
| *Lutjanus russellii* | A | blood | Bioassay | Field sed and water | Cd, Cu, Fe, Pb, Zn |  |  | - |  |  | + |  |  |  |  | [19] |
| *Mugil cephalus* | A | liver | Bioassay | Field water | Cd, Cr, Cu, Fe, Hg, Mn, Ni, Pb, Se, Zn |  | - | - | - | - | + |  |  |  | LHP +;  CD +;  CP + | [20] |
| *Mugil cephalus* | J | gill | Bioassay | Lab water toxicity test | Pb |  |  | - |  |  | + |  |  |  |  | [21] |
|  |  | whole | Bioassay | Lab water toxicity test | Pb |  |  | - |  |  | + |  |  |  |  | [21] |
|  |  | liver | Bioassay | Field sed and water | Cd, Cu, Mn, Ni, Pb, | AHCs, PAHs, PCBs, DDTs, TBT |  | +/- |  |  | + | +/- |  |  |  | [22] |
| *Paralichthys olivaceus* | J | gill | Bioassay | Lab water toxicity test | Cd |  | - | = | - |  | + |  |  |  |  | [23] |
|  |  | kidney | Bioassay | Lab water toxicity test | Cd |  | - | = | + |  | = |  |  |  |  | [23] |
|  |  | liver | Bioassay | Lab water toxicity test | Cd |  | + | = | - |  | + |  |  |  |  | [23] |
|  |  | whole | Bioassay | Lab water toxicity test | Cd (≤12 µg L-) |  | + | = |  |  | + |  |  |  |  | [24] |
|  | L meta | whole | Bioassay | Lab water toxicity test | Cd (48 µg L-) |  | - | +/- |  |  | +/- |  |  |  |  | [24] |
|  | L set | whole | Bioassay | Lab water toxicity test | Cd |  | = | +/- |  |  | = |  |  |  |  | [24] |
| *Platichthys flesus L.* | A | liver | Bioassay | Field water and sed | As, Cd, Cr, Cu, Hg, Ni, Pb, Zn | PAHs, PCBs, OCPs | +/- | +/- |  |  | +/- |  |  |  | KMBA +/-; OP =; | [25] |
| *Poecilia vivipara* acclimated to saltwater | A | gills | Bioassay | Lab water toxicity test | Cu |  | - | = |  | = | + |  | + |  | ACAP = | [26] |
|  |  | liver | Bioassay | Lab water toxicity test | Cu |  | - | + |  | +/- | + |  | + |  | ACAP + | [26] |
|  |  | muscle | Bioassay | Lab water toxicity test | Cu |  | = | = |  | = | +/- |  | = |  | ACAP - | [26] |
| *Pomadasys hasta* | A | blood | Bioassay | Field sed and water | Cd, Cu, Fe, Pb, Zn |  |  | - |  |  | + |  |  |  |  | [19] |
| *Pomatoschistus microps* | A | liver | Bioassay | Field sed | Cd, Cr, Cu, Hg, Ni, Pb, Zn | PAHs | = |  |  |  | = |  |  |  |  | [27] |
|  |  |  |  | Field sed | Cd, Hg, Ni, Pb, Zn | PAHS | +/- | +/- |  |  | +/- |  |  |  |  | [6] |
|  |  |  |  | Field sed | Cr, Cu, Ni, Pb, Zn | PAHs | +/- | +/- | = |  | = | +/- |  |  |  | [9] |
| *Rachycentron canadum* | J | liver | Bioassay | Lab food | Cd |  | - |  |  |  |  |  |  |  |  | [28] |
| *Scophthalmus maximus* | J | liver | Bioassay | Caged field sed | Cd, Cr, Cu, Ni, Pb, Zn |  |  | - |  |  |  |  |  |  |  | [13] |
|  |  |  |  | Lab field sed toxicity | Cd, Cr, Cu, Mn, Ni, Pb, V, Zn |  |  | +/- |  |  |  |  |  |  |  | [29] |
| *Seriola lalandi* | J | RBC | Bioassay | Lab food | Se |  |  |  | + |  |  |  |  |  |  | [30] |
| *Solea senegalensis* | A | gill | Bioassay | Field sed | Cd, Cr, Cu, Fe, Hg, Pb, Zn |  |  |  |  |  | - |  |  |  |  | [31] |
|  |  | liver | Bioassay | Field sed | Cr, Cu, Ni, Pb, Zn | PAHs | +/- | +/- | = |  | = | = |  |  |  | [9] |
|  |  |  |  | Field water and sed | As, Cd, Cu, Fe, Pb, Zn | PAHs |  | +/- | = | +/- | +/- |  |  |  |  | [32] |
|  |  | muscle | Bioassay | Field sed | Cd, Cr, Cu, Fe, Hg, Pb, Zn |  |  |  |  |  | + |  |  |  |  | [31] |
|  | J | blood | Bioassay | Caged field sed | Cd, Cr, Cu, Ni, Pb, Zn | PAHs, PCBs, DDT |  |  |  |  | + |  |  |  |  | [33] |
|  |  |  |  | Lab field sed toxicity | Cd, Cr, Cu, Ni, Pb, Zn | PAHs, PCBs, DDT |  |  |  |  | - |  |  |  |  | [33] |
|  |  | liver | Bioassay | Field sed | Cd, Cr, Cu, Ni, Pb, Zn | PAHs | = | = |  |  | = |  |  |  |  | [34] |
|  |  |  | Real time PCR | Lab and field sed | As, Cu, Zn | PAHs, PCBs, DDT |  | +/- | +/- |  |  |  |  |  |  | [35] |
| *Solea solea* | A | gill | Bioassay | Field sed | Cd, Cr, Cu, Fe, Hg, Pb, Zn |  |  |  |  |  | = |  |  |  |  | [31] |
|  |  | muscle | Bioassay | Field sed | Cd, Cr, Cu, Fe, Hg, Pb, Zn |  |  |  |  |  | = |  |  |  |  | [31] |
| *Sparus aurata* | J | liver | Real time PCR | Lab field sed toxicity | As, Cd, Cr, Cu, Hg, Ni, Pb, Se, V, Zn | PAHs |  |  |  | +/- |  |  |  |  |  | [36] |
|  |  | liver, gill and kidney | Real time PCR | Food and water lab | Cu |  |  |  |  | +/- |  |  |  |  |  | [37] |
| *Symphodus melops* | A | blood | Bioassay | Field water and sed | Fe, Pb, Zn |  | = |  |  |  |  |  |  |  | G6PHD -M; metHB =; CP +; | [38] |
| *Symphodus melops* | A | liver | Bioassay | Field water and sed | Fe, Pb, Zn |  | = | = |  | = |  |  |  |  |  | [38] |
| *Synechogobius hasta* | J | gill | Bioassay | Lab water toxicity test | Cd |  | - | - | - |  | + |  |  |  |  | [39] |
|  |  | liver | Bioassay | Lab water toxicity test | Cd |  | - | - | - |  | + |  |  |  |  | [39] |
|  |  | spleen | Bioassay | Lab water toxicity test | Cd |  | - | - | - |  | + |  |  |  |  | [39] |
| *Terapon jarbua* | J | gill | Bioassay | Lab water toxicity test | Pb |  |  | - |  |  | + |  |  |  |  | [21] |
|  |  | liver | Bioassay | Lab food | Cd |  | = |  |  |  |  |  |  |  |  | [40] |
|  |  | whole | Bioassay | Lab water toxicity test | Pb |  |  | + |  |  | + |  |  |  |  | [21] |

Abbreviations: LHS: life history stage; A: adult, J: juvenile, L: larvae; RBC: red blood cells; meta : metamorphosing; set: settling; Lab: laboratory; Sed : Sediment; SOD: Superoxidase mutase; CAT: Catalase; GP_X_ : glutathione peroxidases; GR: glutathione reductase; LPO: lipid peroxidation; Se-GPx: selenium-dependent glutathione peroxidase; ROS: reactive oxygen species; TOSC: Total oxyradical scavenging capacity; + induction; - inhibition; = no significant induction; +/- mixed response; AHCs: aliphatic hydrocarbons; OCP: total organochlorine pesticides ; PAHs : total polycyclic aromatic hydrocarbons; PCBS: polychlorinated biphenyl; TBT: tributyltin; DDT: dichlorodiphenyltrichloroethane; AOX: Acyl-CoA oxidase (peroxisomal proliferation); G6PD: Glucose -6-phosphate dehydrogenase; metHB: methemoglobin; NOS: nitric oxide synthase; HIF-1: Hypoxia inducible factor ; ACAP: antioxidant capacity against peroxyls radicals; LHP: lipid hydroperoxide; CD: conjugated diene; CP: carbonyl proteins; KMBA: 2-Keto-4-methiolbutyric acid; OP: oxidised proteins; M: males only.

# References

1. Beg MU, Al-Jandal N, Al-Subiai S, Karam Q, Husain S, Butt SA, et al. Metallothionein, oxidative stress and trace metals in gills and liver of demersal and pelagic fish species from Kuwaits’ marine area. Mar Pollut Bull. 2015; 100: 662-72. doi: 10.1016/j.marpolbul.2015.07.058
2. Gravato C, Guimaraes L, Santos J, Faria M, Alves A, Guilhermino L. Comparative study about the effects of pollution on glass and yellow eels (*Anguilla anguilla*) from the estuaries of Minho, Lima and Douro Rivers (NW Portugal). Ecotoxicol Environ Saf. 2010; 73: 524-33. doi: 10.1016/j.ecoenv.2009.11.009 PMID: 000277103600009
3. Piva F, Ciaprini F, Onorati F, Benedetti M, Fattorini D, Ausili A, et al. Assessing sediment hazard through a weight of evidence approach with bioindicator organisms: a practical model to elaborate data from sediment chemistry, bioavailability, biomarkers and ecotoxicological bioassays. Chemosphere. 2011; 83: 475-85. doi: 10.1016/j.chemosphere.2010.12.064 PMID: 21239037
4. Benedetti M, Ciaprini F, Piva F, Onorati F, Fattorini D, Notti A, et al. A multidisciplinary weight of evidence approach for classifying polluted sediments: Integrating sediment chemistry, bioavailability, biomarkers responses and bioassays. Environ Int. 2012; 38: 17-28. doi: 10.1016/j.envint.2011.08.003 PMID: 21982029
5. Annabi A, Kessabi K, Navarro A, Said K, Messaoudi I, Pina B. Assessment of reproductive stress in natural populations of the fish *Aphanius fasciatus* using quantitative mRNA markers. Aquat Biol. 2012; 17: 285-+. doi: 10.3354/ab00482 PMID: 000312247800008
6. Fonseca VF, Vasconcelos RP, Franca S, Serafim A, Lopes B, Company R, et al. Modeling fish biological responses to contaminants and natural variability in estuaries. Mar Environ Res. 2014; 96: 45-55. doi: 10.1016/j.marenvres.2013.10.011 PMID: 000334981600007
7. Souza IC, Duarte ID, Pimentel NQ, Rocha LD, Morozesk M, Bonomo MM, et al. Matching metal pollution with bioavailability, bioaccumulation and biomarkers response in fish (*Centropomus parallelus*) resident in neotropical estuaries. Environ Pollut. 2013; 180: 136-44. doi: 10.1016/j.envpol.2013.05.017 PMID: 000322425300019
8. Fasulo S, Mauceri A, Maisano M, Giannetto A, Parrino V, Gennuso F, et al. Immunohistochemical and molecular biomarkers in *Coris julis* exposed to environmental contaminants. Ecotoxicol Environ Saf. 2010; 73: 873-82. doi: 10.1016/j.ecoenv.2009.12.025 PMID: 000279623800023
9. Fonseca VF, Franca S, Serafim A, Company R, Lopes B, Bebianno MJ, et al. Multi-biomarker responses to estuarine habitat contamination in three fish species: *Dicentrarchus labrax*, *Solea senegalensis* and *Pomatoschistus microps*. Aquat Toxicol. 2011; 102: 216-27. doi: 10.1016/j.aquatox.2011.01.018 PMID: 21356184
10. Cotou E, Henry M, Zeri C, Rigos G, Torreblanca A, Catsiki V-A. Short-term exposure of the European sea bass *Dicentrarchus labrax* to copper-based antifouling treated nets: Copper bioavailability and biomarkers responses. Chemosphere. 2012; 89: 1091-7. doi:10.1016/j.chemosphere.2012.05.075
11. De Domenico E, Mauceri A, Giordano D, Maisano M, Gioffre G, Natalotto A, et al. Effects of "in vivo" exposure to toxic sediments on juveniles of sea bass (*Dicentrarchus labrax*). Aquat Toxicol. 2011; 105: 688-97. doi: 10.1016/j.aquatox.2011.08.026 PMID: 000298120600055
12. De Domenico E, Mauceri A, Giordano D, Maisano M, Giannetto A, Parrino V, et al. Biological responses of juvenile European sea bass (*Dicentrarchus labrax*) exposed to contaminated sediments. Ecotoxicol Environ Saf. 2013; 97: 114-23. doi: 10.1016/j.ecoenv.2013.07.015 PMID: 000325039400015
13. Kerambrun E, Sanchez W, Henry F, Amara R. Are biochemical biomarker responses related to physiological performance of juvenile sea bass (*Dicentrarchus labrax*) and turbot (*Scophthalmus maximus*) caged in a polluted harbour? Comp Biochem Phys C. 2011; 154: 187-95. doi: 10.1016/j.cbpc.2011.05.006 PMID: 000293994200007
14. Ferreira M, Caetano M, Costa J, Pousao-Ferreira P, Vale C, Reis-Henriques MA. Metal accumulation and oxidative stress responses in, cultured and wild, white seabream from Northwest Atlantic. Sci Total Environ. 2008; 407: 638-46. doi: 10.1016/j.scitotenv.2008.07.058 PMID: 000261877900060
15. Ransberry VE, Morash AJ, Blewett TA, Wood CM, McClelland GB. Oxidative stress and metabolic responses to copper in freshwater- and seawater-acclimated killifish, *Fundulus heteroclitus*. Aquat Toxicol. 2015; 161: 242-52. doi: 10.1016/j.aquatox.2015.02.013 PMID: 000352177500026
16. Livingstone DR, Archibald S, Chipman JK, Marsh JW. Antioxidant enzymes in liver of dab *Limanda limanda* from the North Sea. Mar Ecol-Prog Ser. 1992; 91: 97-104. doi: 10.3354/meps091097 PMID: A1992LL06400013
17. Pereira P, de Pablo H, Vale C, Pacheco M. Combined use of environmental data and biomarkers in fish (*Liza aurata*) inhabiting a eutrophic and metal-contaminated coastal system - Gills reflect environmental contamination. Mar Environ Res. 2010; 69: 53-62. doi: 10.1016/j.marenvres.2009.08.003 PMID: 000274773800001
18. Pereira P, de Pablo H, Pacheco M, Vale C. The relevance of temporal and organ specific factors on metals accumulation and biochemical effects in feral fish (*Liza aurata*) under a moderate contamination scenario. Ecotoxicol Environ Saf. 2010; 73: 805-16. doi: 10.1016/j.ecoenv.2010.02.020 PMID: 000279623800015
19. Omar WA, Saleh YS, Marie M-AS. The use of biotic and abiotic components of Red Sea coastal areas as indicators of ecosystem health. Ecotoxicol. 2016; 25: 253-66. doi: 10.1007/s10646-015-1584-8 PMID: 000370716000001
20. Padmini E, Rani MU. Evaluation of oxidative stress biomarkers in hepatocytes of grey mullet inhabiting natural and polluted estuaries. Sci Total Environ. 2009; 407: 4533-41. doi: 10.1016/j.scitotenv.2009.04.005 PMID: 000267631700019
21. Hariharan G, Purvaja R, Ramesh R. Environmental safety level of lead (Pb) pertaining to toxic effects on grey mullet (*Mugil cephalus*) and Tiger perch (*Terapon jarbua*). Environ Toxicol. 2016; 31: 24-43. doi: 10.1002/tox.22019 PMID: 000366585300003
22. Tsangaris C, Vergolyas M, Fountoulaki E, Nizheradze K. Oxidative Stress and Genotoxicity Biomarker Responses in Grey Mullet (*Mugil cephalus*) From a Polluted Environment in Saronikos Gulf, Greece. Arch Environ Con Tox. 2011; 61: 482-90. doi: 10.1007/s00244-010-9629-8 PMID: 000298500400013
23. Cao L, Huang W, Shan X, Ye Z, Dou S. Tissue-specific accumulation of cadmium and its effects on antioxidative responses in Japanese flounder juveniles. Environ Toxicol Pharmacol. 2012; 33: 16-25. doi: 10.1016/j.etap.2011.10.003 PMID: 000301876600003
24. Cao L, Huang W, Liu J, Yin X, Dou S. Accumulation and oxidative stress biomarkers in Japanese flounder larvae and juveniles under chronic cadmium exposure. Comp Biochem Phys C. 2010; 151: 386-92. doi: 10.1016/j.cbpc.2010.01.004 PMID: 000275627400016
25. Schipper CA, Lahr J, van den Brink PJ, George SG, Hansen P-D, de Assis HCdS, et al. A retrospective analysis to explore the applicability of fish biomarkers and sediment bioassays along contaminated salinity transects. Ices J Mar Sci. 2009; 66: 2089-105. doi: 10.1093/icesjms/fsp194 PMID: 000272080600003
26. de Souza Machado AA, Mueller Hoff ML, Klein RD, Cardozo JG, Giacomin MM, Ledes Pinho GL, et al. Biomarkers of waterborne copper exposure in the guppy *Poecilia vivipara* acclimated to salt water. Aquat Toxicol. 2013; 138: 60-9. doi: 10.1016/j.aquatox.2013.04.009. PMID: 000322293600007
27. Serafim A, Company R, Lopes B, Fonseca VF, Franca S, Vasconcelos RP, et al. Application of an integrated biomarker response index (IBR) to assess temporal variation of environmental quality in two Portuguese aquatic systems. Ecol Indic. 2012; 19: 215-25. doi: 10.1016/j.ecolind.2011.08.009 PMID: 000302891100022
28. Liu K, Chi S, Liu H, Dong X, Yang Q, Zhang S, et al. Toxic effects of two sources of dietborne cadmium on the juvenile cobia, *Rachycentron canadum* L. and tissue-specific accumulation of related minerals. Aquat Toxicol. 2015; 165: 120-8. doi: 10.1016/j.aquatox.2015.05.013 PMID: 000359030300013
29. Kerambrun E, Henry F, Marechal A, Sanchez W, Minier C, Filipuci I, et al. A multibiomarker approach in juvenile turbot, *Scophthalmus maximus*, exposed to contaminated sediments. Ecotoxicol Environ Saf. 2012; 80: 45-53. doi: 10.1016/j.ecoenv.2012.02.010 PMID: 000304337300007
30. Ky Trung L, Fotedar R. Toxic effects of excessive levels of dietary selenium in juvenile yellowtail kingfish (*Seriola lalandi*). Aquacult. 2014; 433: 229-34. doi: 10.1016/j.aquaculture.2014.06.021 PMID: 000342529400033
31. Siscar R, Torreblanca A, Palanques A, Sole M. Metal concentrations and detoxification mechanisms in *Solea solea* and *Solea senegalensis* from NW Mediterranean fishing grounds. Mar Pollut Bull. 2013; 77: 90-9. doi: 10.1016/j.marpolbul.2013.10.026 PMID: 000329888600025
32. Oliva M, Jose Vicente J, Gravato C, Guilhermino L, Dolores Galindo-Riano M. Oxidative stress biomarkers in Senegal sole, *Solea senegalensis*, to assess the impact of heavy metal pollution in a Huelva estuary (SW Spain): Seasonal and spatial variation. Ecotoxicol Environ Saf. 2012; 75: 151-62. doi: 10.1016/j.ecoenv.2011.08.017 PMID: 000297088500020
33. Costa PM, Neuparth TS, Caeiro S, Lobo J, Martins M, Ferreira AM, et al. Assessment of the genotoxic potential of contaminated estuarine sediments in fish peripheral blood: Laboratory versus in situ studies. Environ Res. 2011; 111: 25-36. doi: 10.1016/j.envres.2010.09.011 PMID: 000286715300005
34. Fonseca VF, Vasconcelos RP, Tanner SE, Franca S, Serafim A, Lopes B, et al. Habitat quality of estuarine nursery grounds: Integrating non-biological indicators and multilevel biological responses in *Solea senegalensis*. Ecol Indic. 2015; 58: 335-45. doi: 10.1016/j.ecolind.2015.05.064 PMID: 000360776100035
35. Costa PM, Caeiro S, Vale C, Angel DelValls T, Costa MH. Can the integration of multiple biomarkers and sediment geochemistry aid solving the complexity of sediment risk assessment? A case study with a benthic fish. Environ Pollut. 2012; 161: 107-20. doi: 10.1016/j.envpol.2011.10.010 PMID: 000300539300016
36. Ribecco C, Baker ME, Sasik R, Zuo Y, Hardiman G, Carnevali O. Biological effects of marine contaminated sediments on *Sparus aurata* juveniles. Aquat Toxicol. 2011; 104: 308-16. doi: 10.1016/j.aquatox.2011.05.005 PMID: 000293042100017
37. Minghetti M, Leaver MJ, Carpene E, George SG. Copper transporter 1, metallothionein and glutathione reductase genes are differentially expressed in tissues of sea bream (*Sparus aurata*) after exposure to dietary or waterborne copper. Comp Biochem Phys C. 2008; 147: 450-9. doi: 10.1016/j.cbpc.2008.01.014 PMID: 18304880
38. Almroth BC, Sturve J, Stephensen E, Holth TF, Forlin L. Protein carbonyls and antioxidant defenses in corkwing wrasse (*Symphodus melops*) from a heavy metal polluted and a PAH polluted site. Mar Environ Res. 2008; 66: 271-7. doi: 10.1016/j.marenvres.2008.04.002 PMID: 000257817100006
39. Liu XJ, Luo Z, Li CH, Xiong BX, Zhao YH, Li XD. Antioxidant responses, hepatic intermediary metabolism, histology and ultrastructure in *Synechogobius hasta* exposed to waterborne cadmium. Ecotoxicol Environ Saf. 2011; 74: 1156-63. doi: 10.1016/j.ecoenv.2011.02.015 PMID: 000291960600007
40. Dang F, Wang W-X. Assessment of tissue-specific accumulation and effects of cadmium in a marine fish fed contaminated commercially produced diet. Aquat Toxicol. 2009; 95: 248-55. doi: 10.1016/j.aquatox.2009.09.013 PMID: 000272784900009
