# Supplementary material for: A protocol for identifying suitable biomarkers to assess fish health: A systematic review
Source: PLoS One. 2017 Apr 12;12(4):e0174762. doi: 10.1371/journal.pone.0174762 (PMC5389625; doi:10.1371/journal.pone.0174762)
Supplement: S17 Table — (DOCX) [file pone.0174762.s017.docx]

**S17 Table. Field and laboratory studies on responses of biomarkers of exposure in fish to metals and other contaminants: bio transformational products, stress proteins, metallothioneins and metal proteins.** Most studies measured contaminants in the environment in addition to those identified as of concern for Gladstone Harbour (Al, Cd, Cu, Ga, Pb, Se, Zn); these are also presented for completeness.

| Species | LHS | Tissue | Method | Laboratory or Field | Metals | Other contaminants | HSP70 | HSP90 | MT | Others | Reference |
| --- | --- | --- | --- | --- | --- | --- | --- | --- | --- | --- | --- |
| *Acanthopagrus latus* | A | gills | Bioassay | Field sed | As, Cr, Cu, Ni, Pb, V, Zn | PAHs |  |  | = |  | [1] |
|  |  | liver | Bioassay | Field sed | As, Cr, Cu, Ni, Pb, V, Zn | PAHs |  |  | +/- |  | [1] |
|  | J | whole | Ag-saturation | Food lab | Zn |  |  |  | + |  | [2] |
|  |  |  | Ag-saturation | Lab water toxicity test | Zn |  |  |  | + |  | [2] |
| *Anguilla anguilla* | J | liver | Bioassay | Caged field sed | As, Cd, Cr, Cu, Fe, Hg, Mn, Ni, Pb, V, Zn | PAHs |  |  | = |  | [3] |
|  |  |  |  | Lab field sed | As, Cd, Cr, Cu, Fe, Hg, Mn, Ni, Pb, V, Zn | PAHs |  |  | = |  | [3] |
|  |  |  |  | Lab field sed toxicity | As, Cd, Cr, Cu, Hg, Ni, Pb, V, Zn | PAH |  |  | = |  | [4] |
| *Aphanius fasciatus* | A | gonads/liver | Real time PCR | Field water and sed | Cd, Cu, Zn | PAHs | = |  | = |  | [5] |
|  | J | bone tissue | Real time PCR | Lab water toxicity test | Cd |  |  |  | +/- |  | [6] |
| *Atherina presbyter* | A | liver | Bioassay | Field sed | Cd, Hg, Ni, Pb, Zn | PAHs |  |  | +/- |  | [7] |
| *Atherinops affnis* | L | whole | Bioassay | Lab water toxicity test | Cd |  |  |  | + |  | [8] |
| *Chanos chanos* | A | muscle, liver and gills | Immuno-histochemical | Field water and sed | Cd, Cu, Fe, Mn, Pb, Zn |  | + |  |  |  | [9] |
| *Coris julis* | A | gills | Immuno-histochemical | Field sed | Cd, Co, Cr, Cu, Ni, Pb, Sb, Zn |  | + |  | + |  | [10] |
|  |  |  | *In situ* hybridisaton | Field sed | Cd, Co, Cr, Cu, Ni, Pb, Sb, Zn |  | + |  | + |  | [10] |
|  |  |  | Real time PCR | Field sed | Cd, Co, Cr, Cu, Ni, Pb, Sb, Zn |  | + |  | + |  | [10] |
|  |  | liver | Western blot | Field water and sed | As, Cd, Cr, Cu, Hg, Pb, Zn | PAHs, PCBs | + |  | +/- |  | [11] |
|  |  | muscle | Western blot | Field water and sed | As, Cd, Cr, Cu, Hg, Pb, Zn | PAHs, PCBs | + |  | + |  | [11] |
| *Cynoglossus arel* | A | gills | Bioassay | Field sed | As, Cr, Cu, Ni, Pb, V, Zn | PAHs |  |  | = |  | [1] |
|  |  | liver | Bioassay | Field sed | As, Cr, Cu, Ni, Pb, V, Zn | PAHs |  |  | +/- |  | [1] |
| *Dicentrarchus labrax* | A | liver | Bioassay | Field sed | Cr, Cu, Ni, Pb, Zn | PAHs |  |  | = |  | [12] |
|  | J | gills | Immuno-histochemical | Lab field sed toxicity | As, Cd, Co, Cr, Cu, Fe, Hg, Mn, Ni, Pb, Sb, V, Zn |  |  |  | + |  | [13] |
|  |  |  | Real time PCR | Lab field sed toxicity | As, Cd, Co, Cr, Cu, Fe, Hg, Mn, Ni, Pb, Sb, V, Zn |  |  |  | + |  | [13] |
| *Gadus morhua L.* | A | liver | Bioassay | Cage field water | Cd, Cu, Hg, Pb, Zn | PAHs, PCBs |  |  | = |  | [14] |
| *Gobius niger* | A | liver | Bioassay | Lab water toxicity test | Cd |  |  |  | + |  | [15] |
|  |  |  | Southern blot | Lab water toxicity test | Cd |  | + |  | + |  | [15] |
|  |  | testis | Bioassay | Lab water toxicity test | Cd |  |  |  | + |  | [15] |
|  |  |  | Southern blot | Lab water toxicity test | Cd |  | + |  | + |  | [15] |
| *Liza aurata* | J | liver | Bioassay | Field water and sed | Cd, Cu, Hg, Zn |  |  |  | + |  | [16] |
| *Mullus barbatus* | A | liver | Bioassay | Field sed | As, Cd, Cu, Hg, Pb, Zn | PAHs, CBs, DDT, HCB, trans-nonachlor, Lindane, Dieldrin |  |  | = |  | [17] |
| *Parablennius sanguinolentus* | A | liver | Western blot | Field water and sed | Cr, Pb |  | +/- |  |  |  | [18] |
| *Plastichthys flesus* | A | liver | Bioassay | Cage field water | Cd, Cu, Hg, Pb, Zn | PAHs, PCBs |  |  | = |  | [14] |
|  |  |  |  | Field water and sed | As, Cd, Cr, Cu, Hg, Ni, PbZn, | PAHs, PCBs, OCPs |  |  | +/- |  | [19] |
| *Poecilia vivipara* acclimated to saltwater | A | gills | Bioassay | Lab water toxicity test | Cu |  |  |  | +/- |  | [20] |
|  |  | liver | Bioassay | Lab water toxicity test | Cu |  |  |  | - |  | [20] |
| *Pomatoschistus microps* | A | liver | Bioassay | Field sed | Cd, Cr, Cu, Hg, Ni, Pb, Zn, | PAHs |  |  | - |  | [21] |
|  |  |  |  |  | Cd, Hg, Ni, Pb, Zn | PAHS |  |  | +/- |  | [7] |
|  |  |  |  |  | Cr, Cu, Ni, Pb, Zn | PAHs |  |  | = |  | [12] |
| *Scomber scombrus* | A | liver | Bioassay | Field water | Cd, Cu, Hg, Ni, Pb, Zn |  |  |  | = |  | [22] |
| *Solea senegalensis* | A | liver | Bioassay | Field sed | Cr, Cu, Ni, Pb, Zn | PAHs |  |  | + |  | [12] |
|  | J | liver | Bioassay | Field sed | Cd, Cr, Cu, Ni, Pb, Zn | PAHs |  |  | = |  | [23] |
|  |  |  |  | Lab field sed | As, Cd, Cr, Cu, Ni, Pb, Zn | PAHs, PCBs, DDT |  |  | = |  | [24] |
|  |  |  |  | Lab field sed toxicity | As, Cd, Cr, Cu, Hg, Ni, Pb, Zn | PAHs |  |  | = |  | [25] |
|  |  |  | Real time PCR | Lab and field sed | As, Cu, Zn | PAHs, PCBs, DDT |  | +/- | +/- |  | [26] |
|  |  |  | Electrophoretic | Caged field sed | Cd, Cr, Cu, Ni, Pb, Zn | PAHs, PCBs, DDT |  |  | = |  | [27] |
| *Solea solea* | A | gill | Bioassay | Field sed | Cd, Cr, Cu, Fe, Hg, Pb, Zn |  |  |  | + |  | [28] |
|  |  | muscle | Bioassay | Field sed | Cd, Cr, Cu, Fe, Hg, Pb, Zn |  |  |  | + |  | [28] |
| *Sparus aurata* | A | liver | Bioassay | Field water | Cd, Cu, Hg, Ni, Pb, Zn |  |  |  | + |  | [22] |
|  |  |  | Real time PCR | Lab and field sed | As, Cd, Cu, Pb, Zn |  | = | = | = |  | [29] |
|  |  | liver/kidney | Chromatography | Lab water toxicity test | Cd |  |  |  | + |  | [30] |
|  |  | skin | Real time PCR | Lab and field sed | As, Cd, Cu, Pb, Zn |  | = | - | +/- |  | [29] |
|  | J | liver | Bioassay | Lab field sed toxicity | As, Cd, Cr, Cu, Hg, Ni, Pb, Zn | PAHs |  |  | = |  | [25] |
|  |  |  | Real time PCR | Lab field sed toxicity | As, Cd, Cr, Cu, Hg, Ni, Pb, Se, V, Zn, | PAHs | + |  | +/- |  | [31] |
|  |  | liver, gill and kidney | Real time PCR | Food and water lab | Cu |  |  |  | +/- | Ctr1 +/- | [32] |
| *Symphodus melops* | A | liver/blood | Bioassay | Field water and sed | Fe, Pb, Zn |  |  |  | = |  | [33] |
| *Terapon jarbua* | J | carcass | Ag-saturation | Food lab | Zn |  |  |  | + |  | [2] |
|  |  |  |  | Lab water toxicity test | Zn |  |  |  | + |  | [2] |
|  |  | digestive tract | Bioassay | Lab water toxicity test | Cd |  |  |  | = |  | [34] |
|  |  | gills | Ag-saturation | Food lab | Zn |  |  |  | = |  | [2] |
|  |  |  |  | Lab water toxicity test | Zn |  |  |  | = |  | [2] |
|  |  |  | Bioassay | Lab water toxicity test | Cd |  |  |  | = |  | [34] |
|  |  | liver | Bioassay | Lab water toxicity test | Cd |  |  |  | + |  | [34] |
|  |  | viscera | Ag-saturation | Food lab | Zn |  |  |  | + |  | [2] |
|  |  |  |  | Lab water toxicity test | Zn |  |  |  | + |  | [2] |
|  |  | whole | Ag-saturation | Food lab | Zn |  |  |  | + |  | [2] |
|  |  |  |  | Lab water toxicity test | Zn |  |  |  | + |  | [2] |
| *Thunnus thynnus* | A | liver | Bioassay | Field water | Cd, Cu, Hg, Ni, Pb, Zn |  |  |  | + |  | [22] |

Abbreviations: LHS: life history stage; A: adult, J: juveniles; Lab: laboratory; Sed : Sediment; AHCs: aliphatic hydrocarbons; HCB - hexachlorobenzene ; OCP: total organochlorine pesticides; CB: chlorinated biphenyls; naph: naphthalenes; PAHs: total polycyclic aromatic hydrocarbons ; PCBS: polychlorinated biphenyl; TBT: tributyltin; DBT: dibutytin; DDD: 1,1-dichloro-2.2-bis(p-chlorophenyl) ethane; DDE : 1,1-dichloro-2.2-bis(p-chlorophenyl) ethylene; HCH: hexachlorcyclohexane; DDT: dichlorodiphenyltrichloroethane;HCB: hexachlorobenzene; HSP70: Heat shock protein 70; HSP90: Heat shock protein 90; MT: Metallothionein; + induction; - inhibition; = no significant induction; +/- mixed response; Ctr1: copper transporter.

# References

1. Beg MU, Al-Jandal N, Al-Subiai S, Karam Q, Husain S, Butt SA, et al. Metallothionein, oxidative stress and trace metals in gills and liver of demersal and pelagic fish species from Kuwaits’ marine area. Mar Pollut Bull. 2015; 100: 662-72. doi: 10.1016/j.marpolbul.2015.07.058
2. Zhang L, Wang WX. Effects of Zn pre-exposure on Cd and Zn bioaccumulation and metallothionein levels in two species of marine fish. Aquat Toxicol. 2005; 73: 353-69. doi: 10.1016/j.aquatox.2005.04.001 PMID: 000230798000003
3. Piva F, Ciaprini F, Onorati F, Benedetti M, Fattorini D, Ausili A, et al. Assessing sediment hazard through a weight of evidence approach with bioindicator organisms: a practical model to elaborate data from sediment chemistry, bioavailability, biomarkers and ecotoxicological bioassays. Chemosphere. 2011; 83: 475-85. doi: 10.1016/j.chemosphere.2010.12.064 PMID: 21239037
4. Benedetti M, Ciaprini F, Piva F, Onorati F, Fattorini D, Notti A, et al. A multidisciplinary weight of evidence approach for classifying polluted sediments: Integrating sediment chemistry, bioavailability, biomarkers responses and bioassays. Environ Int. 2012; 38: 17-28. doi: 10.1016/j.envint.2011.08.003 PMID: 21982029
5. Annabi A, Kessabi K, Navarro A, Said K, Messaoudi I, Pina B. Assessment of reproductive stress in natural populations of the fish *Aphanius fasciatus* using quantitative mRNA markers. Aquat Biol. 2012; 17: 285-+. doi: 10.3354/ab00482 PMID: 000312247800008
6. Kessabi K, Annabi A, Navarro A, Casado M, Hwas Z, Said K, et al. Structural and molecular analysis of pollution-linked deformities in a natural *Aphanius fasciatus* (Valenciennes, 1821) population from the Tunisian coast. J Environ Monitor. 2012; 14: 2254-60. doi: 10.1039/c2em30329a PMID: 000306852100028
7. Fonseca VF, Vasconcelos RP, Franca S, Serafim A, Lopes B, Company R, et al. Modeling fish biological responses to contaminants and natural variability in estuaries. Mar Environ Res. 2014; 96: 45-55. doi: 10.1016/j.marenvres.2013.10.011 PMID: 000334981600007
8. Rose WL, Nisbet RM, Green PG, Norris S, Fan T, Smith EH, et al. Using an integrated approach to link biomarker responses and physiological stress to growth impairment of cadmium-exposed larval topsmelt. Aquat Toxicol. 2006; 80: 298-308. doi: 10.1016/j.aquatox.2006.09.007 PMID: 000242776900010
9. Rajeshkumar S, Munuswamy N. Impact of metals on histopathology and expression of HSP 70 in different tissues of Milk fish (*Chanos chanos*) of Kaattuppalli Island, South East Coast, India. Chemosphere. 2011; 83: 415-21. doi: 10.1016/j.chemosphere.2010.12.086 PMID: 21257190
10. Fasulo S, Mauceri A, Maisano M, Giannetto A, Parrino V, Gennuso F, et al. Immunohistochemical and molecular biomarkers in *Coris julis* exposed to environmental contaminants. Ecotoxicol Environ Saf. 2010; 73: 873-82. doi: 10.1016/j.ecoenv.2009.12.025 PMID: 000279623800023
11. Tomasello B, Copat C, Pulvirenti V, Ferrito V, Ferrante M, Renis M, et al. Biochemical and bioaccumulation approaches for investigating marine pollution using Mediterranean rainbow wrasse, *Coris julis* (Linneaus 1798). Ecotoxicol Environ Saf. 2012; 86: 168-75. doi: 10.1016/j.ecoenv.2012.09.012 PMID: 000311064800024
12. Fonseca VF, Franca S, Serafim A, Company R, Lopes B, Bebianno MJ, et al. Multi-biomarker responses to estuarine habitat contamination in three fish species: *Dicentrarchus labrax*, *Solea senegalensis* and *Pomatoschistus microps*. Aquat Toxicol. 2011; 102: 216-27. doi: 10.1016/j.aquatox.2011.01.018 PMID: 21356184
13. De Domenico E, Mauceri A, Giordano D, Maisano M, Gioffre G, Natalotto A, et al. Effects of "in vivo" exposure to toxic sediments on juveniles of sea bass (*Dicentrarchus labrax*). Aquat Toxicol. 2011; 105: 688-97. doi: 10.1016/j.aquatox.2011.08.026 PMID: 000298120600055
14. Beyer J, Sandvik M, Hylland K, Fjeld E, Egaas E, Aas E, et al. Contaminant accumulation and biomarker responses in flounder (*Platichthys flesus* L) and Atlantic cod (*Gadus morhua* L) exposed by caging to polluted sediments in Sorfjorden, Norway. Aquat Toxicol. 1996; 36: 75-98. doi: 10.1016/s0166-445x(96)00798-9 PMID: A1996VY98200005
15. Migliarini B, Campisi AM, Maradonna F, Truzzi C, Annibaldi A, Scarponi G, et al. Effects of cadmium exposure on testis apoptosis in the marine teleost *Gobius niger*. Gen Comp Endocrinol. 2005; 142: 241-7. doi: 10.1016/j.ygcen.2004.12.012 PMID: 15862569
16. Oliveira M, Ahmad I, Maria VL, Serafim A, Bebianno MJ, Pacheco M, et al. Hepatic metallothionein concentrations in the golden grey mullet (*Liza aurata*) - Relationship with environmental metal concentrations in a metal-contaminated coastal system in Portugal. Mar Environ Res. 2010; 69: 227-33. doi: 10.1016/j.marenvres.2009.10.012 PMID: 000275342300003
17. Martinez-Gomez C, Fernandez B, Benedicto J, Valdes J, Campillo JA, Leon VM, et al. Health status of red mullets from polluted areas of the Spanish Mediterranean coast, with special reference to Portman (SE Spain). Mar Environ Res. 2012; 77: 50-9. doi: 10.1016/j.marenvres.2012.02.002 PMID: 000304296700008
18. Tigano C, Tomasello B, Pulvirenti V, Ferrito V, Copat C, Carpinteri G, et al. Assessment of environmental stress in *Parablennius sanguinolentus* (Pallas, 1814) of the Sicilian Ionian coast. Ecotoxicol Environ Saf. 2009; 72: 1278-86. doi: 10.1016/j.ecoenv.2008.09.028 PMID: 000265767900037
19. Schipper CA, Lahr J, van den Brink PJ, George SG, Hansen P-D, de Assis HCdS, et al. A retrospective analysis to explore the applicability of fish biomarkers and sediment bioassays along contaminated salinity transects. Ices J Mar Sci. 2009; 66: 2089-105. doi: 10.1093/icesjms/fsp194 PMID: 000272080600003
20. de Souza Machado AA, Mueller Hoff ML, Klein RD, Cardozo JG, Giacomin MM, Ledes Pinho GL, et al. Biomarkers of waterborne copper exposure in the guppy *Poecilia vivipara* acclimated to salt water. Aquat Toxicol. 2013; 138: 60-9. doi: 10.1016/j.aquatox.2013.04.009. PMID: 000322293600007
21. Serafim A, Company R, Lopes B, Fonseca VF, Franca S, Vasconcelos RP, et al. Application of an integrated biomarker response index (IBR) to assess temporal variation of environmental quality in two Portuguese aquatic systems. Ecol Indic. 2012; 19: 215-25. doi: 10.1016/j.ecolind.2011.08.009 PMID: 000302891100022
22. Papetti P, Rossi G. Heavy metals in the fishery products of low Lazio and the use of metallothionein as a biomarker of contamination. Environ Monit Ass. 2009; 159: 589-98. doi: 10.1007/s10661-008-0725-4 PMID: 000271530400046
23. Fonseca VF, Vasconcelos RP, Tanner SE, Franca S, Serafim A, Lopes B, et al. Habitat quality of estuarine nursery grounds: Integrating non-biological indicators and multilevel biological responses in *Solea senegalensis*. Ecol Indic. 2015; 58: 335-45. doi: 10.1016/j.ecolind.2015.05.064 PMID: 000360776100035
24. Costa PM, Caeiro S, Diniz MS, Lobo J, Martins M, Ferreira AM, et al. Biochemical endpoints on juvenile *Solea senegalensis* exposed to estuarine sediments: the effect of contaminant mixtures on metallothionein and CYP1A induction. Ecotoxicol. 2009; 18: 988-1000. doi: 10.1007/s10646-009-0373-7 PMID: 000269917200004
25. Jimenez-Tenorio N, Morales-Caselles C, Kalman J, Salamanca MJ, Luisa Gonzalez de Canales M, Sarasquete C, et al. Determining sediment quality for regulatory proposes using fish chronic bioassays. Environ Internat. 2007; 33: 474-80. doi: 10.1016/j.envint.2006.11.009 PMID: 000246315800008
26. Costa PM, Caeiro S, Vale C, Angel DelValls T, Costa MH. Can the integration of multiple biomarkers and sediment geochemistry aid solving the complexity of sediment risk assessment? A case study with a benthic fish. Environ Pollut. 2012; 161: 107-20. doi: 10.1016/j.envpol.2011.10.010 PMID: 000300539300016
27. Costa PM, Repolho T, Caeiro S, Diniz ME, Moura I, Costa MH. Modelling metallothionein induction in the liver of *Sparus aurata* exposed to metal-contaminated sediments. Ecotoxicol Environ Saf. 2008; 71: 117-24. doi: 10.1016/j.ecoenv.2007.05.012 PMID: 000258550400014
28. Siscar R, Torreblanca A, Palanques A, Sole M. Metal concentrations and detoxification mechanisms in *Solea solea* and *Solea senegalensis* from NW Mediterranean fishing grounds. Mar Pollut Bull. 2013; 77: 90-9. doi: 10.1016/j.marpolbul.2013.10.026 PMID: 000329888600025
29. Benhamed S, Guardiola FA, Martínez S, Martínez-Sánchez MJ, Pérez-Sirvent C, Mars M, et al. Exposure of the gilthead seabream (*Sparus aurata*) to sediments contaminated with heavy metals down-regulates the gene expression of stress biomarkers. Toxicol Rep. 2016; 3: 364-72. doi:10.1016/j.toxrep.2016.02.006
30. Isani G, Andreani G, Cocchioni F, Fedeli D, Carpene E, Falcioni G. Cadmium accumulation and biochemical responses in *Sparus aurata* following sub-lethal Cd exposure. Ecotoxicol Environ Saf. 2009; 72: 224-30. doi: 10.1016/j.ecoenv.2008.04.015 PMID: 000260660100028
31. Ribecco C, Baker ME, Sasik R, Zuo Y, Hardiman G, Carnevali O. Biological effects of marine contaminated sediments on *Sparus aurata* juveniles. Aquat Toxicol. 2011; 104: 308-16. doi: 10.1016/j.aquatox.2011.05.005 PMID: 000293042100017
32. Minghetti M, Leaver MJ, Carpene E, George SG. Copper transporter 1, metallothionein and glutathione reductase genes are differentially expressed in tissues of sea bream (*Sparus aurata*) after exposure to dietary or waterborne copper. Comp Biochem Phys C. 2008; 147: 450-9. doi: 10.1016/j.cbpc.2008.01.014 PMID: 18304880
33. Almroth BC, Sturve J, Stephensen E, Holth TF, Forlin L. Protein carbonyls and antioxidant defenses in corkwing wrasse (*Symphodus melops*) from a heavy metal polluted and a PAH polluted site. Mar Environ Res. 2008; 66: 271-7. doi: 10.1016/j.marenvres.2008.04.002 PMID: 000257817100006
34. Dang F, Wang W-X. Assessment of tissue-specific accumulation and effects of cadmium in a marine fish fed contaminated commercially produced diet. Aquat Toxicol. 2009; 95: 248-55. doi: 10.1016/j.aquatox.2009.09.013 PMID: 000272784900009
