# Supplementary material for: A protocol for identifying suitable biomarkers to assess fish health: A systematic review
Source: PLoS One. 2017 Apr 12;12(4):e0174762. doi: 10.1371/journal.pone.0174762 (PMC5389625; doi:10.1371/journal.pone.0174762)
Supplement: S18 Table — (DOCX) [file pone.0174762.s018.docx]

**S18 Table. Field and laboratory studies on responses of biomarkers of exposure in fish to metals and other contaminants: haematological and immunological parameters.** Most studies measured contaminants in the environment in addition to those identified as of concern for Gladstone Harbour (Al, Cd, Cu, Ga, Pb, Se, Zn); these are also presented for completeness.

| Species | LHS | Laboratory or Field | Metals | Other contaminants | ALT / AST | Haematocrit | EC | ALP | Total protein | Albumin | Albumin /Ʃprotein | PCNA | Others | Reference |
| --- | --- | --- | --- | --- | --- | --- | --- | --- | --- | --- | --- | --- | --- | --- |
| *Anguilla anguilla* | J | Caged field sed | As, Cd, Cr, Cu, Fe, Hg, Mn, Ni, Pb, V, Zn | PAHs |  |  |  |  |  |  |  |  | Lys - | [1] |
|  |  | Lab field sed | As, Cd, Cr, Cu, Fe, Hg, Mn, Ni, Pb, V, Zn | PAHs |  |  |  |  |  |  |  |  | Lys - | [1] |
|  |  | Lab field sed toxicity | As, Cd, Cr, Cu, Hg, Ni, Pb, V, Zn | PAH |  |  |  |  |  |  |  |  | LY 1and2 - | [2] |
| *Coris julis* | A | Field sed | Cd, Co, Cr, Cu, Ni, Pb, Sb, |  |  |  |  |  |  |  |  | + | CBP - | [3] |
| *Dicentrarchus labrax* | A | Lab water toxicity test | Cu antifouling |  |  |  |  |  |  |  |  |  | TCAS =;  Haemoglobin +; RB = | [4] |
|  | J | Lab field sed toxicity | As, Cd, Cr, Cu, Hg, Ni, Pb, Zn | PAHs and PCBs |  |  |  |  |  |  |  | + | PCNA RT + | [5] |
|  |  | Lab field sed toxicity | As, Cd, Cr, Cu, Hg, Ni, Pb, Zn | PAHs and PCBs |  |  |  |  |  |  |  |  |  | [6] |
| *Gadus morhua L.* | A | Cage field water | Cd, Cu, Hg, Pb, Zn | PAHs, PCBs | = | = | = | +/- | +/- | = | +/- |  | % leukocytes = ;  % thrombocytes +/-; Glucose = | [7] |
| *Lutjanus russellii* | A | Field sed and water | Cd, Cu, Fe, Pb, Zn |  |  |  |  |  | - | - | = | - |  | [8] |
| *Mugil cephalus* | A | Field water | Cd, Cr, Cu, Fe, Hg, Mn, Ni, Pb, Se, Zn |  |  |  |  |  |  |  |  |  | CV - | [9] |
| *Plastichthys flesus* | A | Cage field water | Cd, Cu, Hg, Pb, Zn | PAHs, PCBs | +/- | = |  | +/- | +/- | +/- | = |  | Glucose = | [7] |
|  |  | Field sed | Al, Cd, Cr, Cu, Fe, Hg, Mn, Pb, Zn, | PCB,DDD,DDE, HCH |  |  |  |  |  |  |  |  | MAM -;  LY1and2 - | [10] |
|  |  |  | Cd, Hg, Pb, Zn | PAHs, PCBs |  |  |  |  |  |  |  |  | Lys +;  MMC + | [11] |
| *Pomadasys hasta* | A | Field sed and water | Cd, Cu, Fe, Pb, Zn |  |  |  |  |  | - | - | = | - |  | [8] |
| *Rachycentron canadum* | J | Lab food | Cd |  | + | + | + | + |  |  |  |  | Glucose + | [12] |
| *Scophthalmus maximus* | J | Lab field sed toxicity | Cd, Cr, Cu, Mn, Ni, Pb, V, Zn |  |  |  |  |  |  |  |  |  | TV =;  TCF-b1 +/-;  Lys +/-;  MMC =/- | [13] |
| *Seriola lalandi* | J | Lab food | Se |  |  | - |  |  |  |  |  |  | MA + | [14] |
| *Solea senegalensis* | J | Caged field sed | Cd, Cr, Cu, Ni, Pb, Zn | PAHs, PCBs, DDT |  |  | = |  |  |  |  |  | % leukocytes -;  % thrombocytes = | [15] |
| *Solea senegalensis* | J | Lab field sed toxicity | Cd, Cr, Cu, Ni, Pb, Zn | PAHs, PCBs, DDT |  |  | = |  |  |  |  |  | % leukocytes - ;  % thrombocytes = | [15] |
| *Sparus aurata* | J | Lab water toxicity test | Cu |  |  |  |  |  |  |  |  |  | Pro-S + | [16] |
| *Squalus acanthias* | A | Lab water toxicity test | Pb |  |  |  |  |  |  |  |  |  | Na -;  Cl - | [17] |
| *Symphodus melops* | A | Field water and sed | Fe, Pb, Zn, |  |  | + |  |  |  |  |  |  |  | [18] |
| *Synechogobius hasta* | J | Lab water toxicity test | Cd |  |  |  |  |  |  |  |  |  | MMC + | [19] |

Abbreviations: LHS: life history stage; J: juvenile, A: adult: Lab: laboratory; Sed : Sediment; PAHs - total polycyclic aromatic hydrocarbons; PCBS: polychlorinated biphenyl; DDD - 1,1-dichloro-2.2-bis(p-chlorophenyl) ethane; DDE - 1,1-dichloro-2.2-bis(p-chlorophenyl) ethylene; HCH - hexachlorcyclohexane; DDT: dichlorodiphenyltrichloroethane; ALP: Alkaline phosphatase; AST aspartate aminotransferase; EC: Erthrocyte count; PCNA - proliferating cell nuclear antigen; + induction; - inhibition; = no significant induction; +/- mixed response; CBP - calcium binding proteins; RB - respiratory burst ; TCAS - total complementary activity of serum; LY1 and 2 - Stability of lysosomes 1 and 2; Lys - Lysozyme activity; MAM - macrophage aggregate activity ; Pro-S: Proteomics serum; TCF-b1: cytokine transforming growth; factor - β1; MMC: melanomacrophage centres; TV: Thymus volume; CV: cell viability.

# References

1. Piva F, Ciaprini F, Onorati F, Benedetti M, Fattorini D, Ausili A, et al. Assessing sediment hazard through a weight of evidence approach with bioindicator organisms: a practical model to elaborate data from sediment chemistry, bioavailability, biomarkers and ecotoxicological bioassays. Chemosphere. 2011; 83: 475-85. doi: 10.1016/j.chemosphere.2010.12.064 PMID: 21239037
2. Benedetti M, Ciaprini F, Piva F, Onorati F, Fattorini D, Notti A, et al. A multidisciplinary weight of evidence approach for classifying polluted sediments: Integrating sediment chemistry, bioavailability, biomarkers responses and bioassays. Environ Int. 2012; 38: 17-28. doi: 10.1016/j.envint.2011.08.003 PMID: 21982029
3. Fasulo S, Mauceri A, Maisano M, Giannetto A, Parrino V, Gennuso F, et al. Immunohistochemical and molecular biomarkers in *Coris julis* exposed to environmental contaminants. Ecotoxicol Environ Saf. 2010; 73: 873-82. doi: 10.1016/j.ecoenv.2009.12.025 PMID: 000279623800023
4. Cotou E, Henry M, Zeri C, Rigos G, Torreblanca A, Catsiki V-A. Short-term exposure of the European sea bass *Dicentrarchus labrax* to copper-based antifouling treated nets: Copper bioavailability and biomarkers responses. Chemosphere. 2012; 89: 1091-7. doi:10.1016/j.chemosphere.2012.05.075
5. De Domenico E, Mauceri A, Giordano D, Maisano M, Giannetto A, Parrino V, et al. Biological responses of juvenile European sea bass (*Dicentrarchus labrax*) exposed to contaminated sediments. Ecotoxicol Environ Saf. 2013; 97: 114-23. doi: 10.1016/j.ecoenv.2013.07.015 PMID: 000325039400015
6. De Domenico E, Mauceri A, Giordano D, Maisano M, Gioffre G, Natalotto A, et al. Effects of "in vivo" exposure to toxic sediments on juveniles of sea bass (*Dicentrarchus labrax*). Aquat Toxicol. 2011; 105: 688-97. doi: 10.1016/j.aquatox.2011.08.026 PMID: 000298120600055
7. Beyer J, Sandvik M, Hylland K, Fjeld E, Egaas E, Aas E, et al. Contaminant accumulation and biomarker responses in flounder (*Platichthys flesus* L) and Atlantic cod (*Gadus morhua* L) exposed by caging to polluted sediments in Sorfjorden, Norway. Aquat Toxicol. 1996; 36: 75-98. doi: 10.1016/s0166-445x(96)00798-9 PMID: A1996VY98200005
8. Omar WA, Saleh YS, Marie M-AS. The use of biotic and abiotic components of Red Sea coastal areas as indicators of ecosystem health. Ecotoxicol. 2016; 25: 253-66. doi: 10.1007/s10646-015-1584-8 PMID: 000370716000001
9. Padmini E, Rani MU. Evaluation of oxidative stress biomarkers in hepatocytes of grey mullet inhabiting natural and polluted estuaries. Sci Total Environ. 2009; 407: 4533-41. doi: 10.1016/j.scitotenv.2009.04.005 PMID: 000267631700019
10. Schmidt V, Zander S, Korting W, Broeg K, von Westernhagen H, Dizer H, et al. Parasites of flounder (*Platichthys flesus* L.) from the German Bight, North Sea, and their potential use in biological effects monitoring - C. Pollution effects on the parasite community and a comparison to biomarker responses. Helgoland Mar Res. 2003; 57: 262-71. doi: 10.1007/s10152-003-0159-x PMID: 000186604600015
11. Vethaak AD, Jol JG, Meijboom A, Eggens ML, apRheinallt T, Wester PW, et al. Skin and liver diseases induced in flounder (*Platichthys flesus*) after long-term exposure to contaminated sediments in large-scale mesocosms. Environ Health Persp. 1996; 104: 1218-29. doi: 10.2307/3432916 PMID: A1996VX74000021
12. Liu K, Chi S, Liu H, Dong X, Yang Q, Zhang S, et al. Toxic effects of two sources of dietborne cadmium on the juvenile cobia, *Rachycentron canadum* L. and tissue-specific accumulation of related minerals. Aquat Toxicol. 2015; 165: 120-8. doi: 10.1016/j.aquatox.2015.05.013 PMID: 000359030300013
13. Kerambrun E, Henry F, Marechal A, Sanchez W, Minier C, Filipuci I, et al. A multibiomarker approach in juvenile turbot, *Scophthalmus maximus*, exposed to contaminated sediments. Ecotoxicol Environ Saf. 2012; 80: 45-53. doi: 10.1016/j.ecoenv.2012.02.010 PMID: 000304337300007
14. Ky Trung L, Fotedar R. Toxic effects of excessive levels of dietary selenium in juvenile yellowtail kingfish (*Seriola lalandi*). Aquacult. 2014; 433: 229-34. doi: 10.1016/j.aquaculture.2014.06.021 PMID: 000342529400033
15. Costa PM, Neuparth TS, Caeiro S, Lobo J, Martins M, Ferreira AM, et al. Assessment of the genotoxic potential of contaminated estuarine sediments in fish peripheral blood: Laboratory versus in situ studies. Environ Res. 2011; 111: 25-36. doi: 10.1016/j.envres.2010.09.011 PMID: 000286715300005
16. Isani G, Andreani G, Carpene E, Di Molfetta S, Eletto D, Spisni E. Effects of waterborne Cu exposure in gilthead sea bream (*Sparus aurata*): A proteomic approach. Fish Shellfish Immun. 2011; 31: 1051-8. doi: 10.1016/j.fsi.2011.09.005 PMID: 000298569700041
17. Eyckmans M, Lardon I, Wood CM, De Boeck G. Physiological effects of waterborne lead exposure in spiny dogfish (*Squalus acanthias*). Aquat Toxicol. 2013; 126: 373-81. doi: 10.1016/j.aquatox.2012.09.004 PMID: 000315125600040
18. Almroth BC, Sturve J, Stephensen E, Holth TF, Forlin L. Protein carbonyls and antioxidant defenses in corkwing wrasse (*Symphodus melops*) from a heavy metal polluted and a PAH polluted site. Mar Environ Res. 2008; 66: 271-7. doi: 10.1016/j.marenvres.2008.04.002 PMID: 000257817100006
19. Liu XJ, Luo Z, Li CH, Xiong BX, Zhao YH, Li XD. Antioxidant responses, hepatic intermediary metabolism, histology and ultrastructure in *Synechogobius hasta* exposed to waterborne cadmium. Ecotoxicol Environ Saf. 2011; 74: 1156-63. doi: 10.1016/j.ecoenv.2011.02.015 PMID: 000291960600007
