# Supplementary material for: A protocol for identifying suitable biomarkers to assess fish health: A systematic review
Source: PLoS One. 2017 Apr 12;12(4):e0174762. doi: 10.1371/journal.pone.0174762 (PMC5389625; doi:10.1371/journal.pone.0174762)
Supplement: S19 Table — (DOCX) [file pone.0174762.s019.docx]

**S19 Table. Field and laboratory studies on responses of biomarkers of exposure in fish to metals and other contaminants: reproductive, endocrine and neurotoxic parameters.** Most studies measured contaminants in the environment in addition to those identified as of concern for Gladstone Harbour (Al, Cd, Cu, Ga, Pb, Se, Zn); these are also presented for completeness.

| Species | LHS | Tissue | Method | Laboratory or Field | Metals | Other contaminants | ChE | Other | Reference |
| --- | --- | --- | --- | --- | --- | --- | --- | --- | --- |
| *Anguilla anguilla* | Glass eels | liver | Bioassay | Field sed | Cd, Cr, Cu, Hg, Ni, Pb, V, Zn, | PAH | - |  | [1] |
|  | J | liver | Bioassay | Caged field sed | As, Cd, Cr, Cu, Fe, Hg, Mn, Ni, Pb, V, Zn | PAHs | = |  | [2] |
|  |  |  |  | Lab field sed | As, Cd, Cr, Cu, Fe, Hg, Mn, Ni, Pb, V, Zn | PAHs | = |  | [2] |
|  |  |  |  | Lab field sed toxicity | As, Cd, Cr, Cu, Hg, Ni, Pb, V, Zn | PAH | = |  | [3] |
|  | Yellow eels | liver | Bioassay | Field sed | Cd, Cr, Cu, Hg, Ni, Pb, V, Zn, | PAH | - |  | [1] |
| *Aphanius fasciatus* | A | liver | Real time PCR | Field water and sed | Cd, Cu, Zn | PAHs |  | VTG + | [4] |
| *Coris julis* | A | gills | Immuno-histochemical | Field sed | Cd, Co, Cr, Cu, Ni, Pb, Sb, Zn |  |  | 5-HT3 +;  5HT - | [5] |
| *Dicentrarchus labrax* | J | gills | Immuno-histochemical | Lab field sed toxicity | As, Cd, Cr, Cu, Hg, Ni, Pb, Zn | PAHs and PCBs | - | ChAT -;  TH +;  5-HT - | [6] |
|  |  |  | Real time PCR | Lab field sed toxicity | As, Cd, Cr, Cu, Hg, Ni, Pb, Zn | PAHs and PCBs | - | 5-HT3 = | [6] |
| *Lates calcarifer* | A | muscle | Bioassay | Field sed | Cd, Cr, Cu, Ni, Zn | Diuron, PAHs | - |  | [7] |
| *Platichthys flesus L.* | A | liver | Bioassay | Field sed | Cd, Cu, Hg, Pb | PCBs | = |  | [8]) |
|  |  |  |  | Field water and sed | As, Cd, Cr, Cu, Hg, Ni, Pb, Zn | PAHs, PCBs, OCPs | +/- | VTG +/- | [9] |
| *Solea senegalensis* | A | brain | Bioassay | Field sed | Cd, Cr, Cu, Fe, Hg, Pb, Zn |  | = |  | [10] |
|  |  | gill | Bioassay | Field sed | Cd, Cr, Cu, Fe, Hg, Pb, Zn |  | + |  | [10] |
|  |  | muscle | Bioassay | Field sed | Cd, Cr, Cu, Fe, Hg, Pb, Zn |  | + |  | [10] |
|  |  |  |  | Field water and sed | As, Cd, Cu, Fe, Pb, Zn | PAHs | +/- |  | [11] |
| *Solea solea* | A | brain | Bioassay | Field sed | Cd, Cr, Cu, Fe, Hg, Pb, Zn |  | + |  | [10] |
|  |  | gill | Bioassay | Field sed | Cd, Cr, Cu, Fe, Hg, Pb, Zn |  | = |  | [10] |
|  |  | muscle | Bioassay | Field sed | Cd, Cr, Cu, Fe, Hg, Pb, Zn |  | - |  | [10] |
| *Sparus aurata* | J | liver | Real time PCR | Lab field sed toxicity | As, Cd, Cr, Cu, Hg, Ni, Pb, Se, V, Zn | PAHs |  | TRα + | [12] |

Abbreviations: LHS: life history stage; J: juvenile, A: adult: Lab: laboratory; Sed : Sediment; PAHs - total polycyclic aromatic hydrocarbons; PCBS: polychlorinated biphenyl; OCP: total organochlorine pesticides; ChE: choline esterase; + induction; - inhibition; = no significant induction; +/- mixed response; VTG: Vitellogenin; TH: tyrosine hydroxylase; ChAT: Choline AcetylTransferase; ChE: AcetylCholineEsterase; 5-HT: Serotonin; 5-HT3: Serotonin receptor; TRα: thyroid receptor alpha.

# References

1. Gravato C, Guimaraes L, Santos J, Faria M, Alves A, Guilhermino L. Comparative study about the effects of pollution on glass and yellow eels (*Anguilla anguilla*) from the estuaries of Minho, Lima and Douro Rivers (NW Portugal). Ecotoxicol Environ Saf. 2010; 73: 524-33. doi: 10.1016/j.ecoenv.2009.11.009 PMID: 000277103600009
2. Piva F, Ciaprini F, Onorati F, Benedetti M, Fattorini D, Ausili A, et al. Assessing sediment hazard through a weight of evidence approach with bioindicator organisms: a practical model to elaborate data from sediment chemistry, bioavailability, biomarkers and ecotoxicological bioassays. Chemosphere. 2011; 83: 475-85. doi: 10.1016/j.chemosphere.2010.12.064 PMID: 21239037
3. Benedetti M, Ciaprini F, Piva F, Onorati F, Fattorini D, Notti A, et al. A multidisciplinary weight of evidence approach for classifying polluted sediments: Integrating sediment chemistry, bioavailability, biomarkers responses and bioassays. Environ Int. 2012; 38: 17-28. doi: 10.1016/j.envint.2011.08.003 PMID: 21982029
4. Annabi A, Kessabi K, Navarro A, Said K, Messaoudi I, Pina B. Assessment of reproductive stress in natural populations of the fish *Aphanius fasciatus* using quantitative mRNA markers. Aquat Biol. 2012; 17: 285-+. doi: 10.3354/ab00482 PMID: 000312247800008
5. Fasulo S, Mauceri A, Maisano M, Giannetto A, Parrino V, Gennuso F, et al. Immunohistochemical and molecular biomarkers in *Coris julis* exposed to environmental contaminants. Ecotoxicol Environ Saf. 2010; 73: 873-82. doi: 10.1016/j.ecoenv.2009.12.025 PMID: 000279623800023
6. De Domenico E, Mauceri A, Giordano D, Maisano M, Giannetto A, Parrino V, et al. Biological responses of juvenile European sea bass (*Dicentrarchus labrax*) exposed to contaminated sediments. Ecotoxicol Environ Saf. 2013; 97: 114-23. doi: 10.1016/j.ecoenv.2013.07.015 PMID: 000325039400015
7. Humphrey CA, King SC, Klumpp DW. A multibiomarker approach in barramundi (*Lates calcarifer*) to measure exposure to contaminants in estuaries of tropical North Queensland. Mar Pollut Bull. 2007; 54: 1569-81. doi: 10.1016/j.marpolbul.2007.06.004 PMID: 000250599700014
8. Schmidt V, Zander S, Korting W, Broeg K, von Westernhagen H, Dizer H, et al. Parasites of flounder (*Platichthys flesus* L.) from the German Bight, North Sea, and their potential use in biological effects monitoring - C. Pollution effects on the parasite community and a comparison to biomarker responses. Helgoland Mar Res. 2003; 57: 262-71. doi: 10.1007/s10152-003-0159-x PMID: 000186604600015
9. Schipper CA, Lahr J, van den Brink PJ, George SG, Hansen P-D, de Assis HCdS, et al. A retrospective analysis to explore the applicability of fish biomarkers and sediment bioassays along contaminated salinity transects. Ices J Mar Sci. 2009; 66: 2089-105. doi: 10.1093/icesjms/fsp194 PMID: 000272080600003
10. Siscar R, Torreblanca A, Palanques A, Sole M. Metal concentrations and detoxification mechanisms in *Solea solea* and *Solea senegalensis* from NW Mediterranean fishing grounds. Mar Pollut Bull. 2013; 77: 90-9. doi: 10.1016/j.marpolbul.2013.10.026 PMID: 000329888600025
11. Oliva M, Antonio Perales J, Gravato C, Guilhermino L, Dolores Galindo-Riano M. Biomarkers responses in muscle of Senegal sole (*Solea senegalensis*) from a heavy metals and PAHs polluted estuary. Mar Pollut Bull. 2012; 64: 2097-108. doi: 10.1016/j.marpolbul.2012.07.017 PMID: 000310929500028
12. Ribecco C, Baker ME, Sasik R, Zuo Y, Hardiman G, Carnevali O. Biological effects of marine contaminated sediments on *Sparus aurata* juveniles. Aquat Toxicol. 2011; 104: 308-16. doi: 10.1016/j.aquatox.2011.05.005 PMID: 000293042100017
