# Supplementary material for: A protocol for identifying suitable biomarkers to assess fish health: A systematic review
Source: PLoS One. 2017 Apr 12;12(4):e0174762. doi: 10.1371/journal.pone.0174762 (PMC5389625; doi:10.1371/journal.pone.0174762)
Supplement: S20 Table — (DOCX) [file pone.0174762.s020.docx]

**S20 Table. Field and laboratory studies on responses of biomarkers of exposure in fish to metals and other contaminants: genotoxic parameters.** Most studies measured contaminants in the environment in addition to those identified as of concern for Gladstone Harbour (Al, Cd, Cu, Ga, Pb, Se, Zn); these are also presented for completeness**.**

| Species | LHS | Laboratory or Field | Metals | Other contaminants | Apoptosis / Caspase | Comet | Micronucleus and nuclear abnormalities | Others | Reference |
| --- | --- | --- | --- | --- | --- | --- | --- | --- | --- |
| *Aldrichetta forsteri* | A | Field sed | Cd, Cu, Pb |  |  |  | + |  | [1] |
| *Anguilla anguilla* | J | Lab field sed | As, Cd, Cr, Cu, Fe, Hg, Mn, Ni, Pb, V, Zn | PAHs |  |  | + |  | [2] |
|  |  |  | As, Cd, Cr, Cu, Hg, Ni, Pb, V, Zn | PAH |  | - | + |  | [3] |
| *Atherinops affnis* | L | Lab water toxicity test | Cd |  | + |  |  |  | [4] |
| *Centropomus parallelus* | J | Field sed and water | Ag, Al, As, Cd, Cr, Cu, Fe, Hg, Mn, Ni, Pb, Se, Zn |  |  |  | = |  | [5] |
|  |  | Lab water toxicity test | Cu |  |  | - | + |  | [6] |
| *Coris julis* | A | Field sed | Cd, Co, Cr, Cu, Ni, Pb, Sb, Zn |  | + |  |  | Fas ligand + | [7] |
|  |  | Field water and sed | As, Cd, Cr, Cu, Hg, Pb, Zn | PAHs, PCBs |  | + | + | Fpg-comet + | [8] |
| *Dicentrarchus labrax* | J | Lab field sed toxicity | As, Cd, Cr, Cu, Hg, Ni, Pb, Zn | PAHs and PCBs |  |  |  | Fas ligand RT +;  Fas ligand - | [9] |
| *Epinephelus coioides* | J | Lab field sed | Cu | PAHs |  | + |  |  | [10] |
| *Gobius niger* | A | Lab water toxicity test | Cd |  | + |  |  |  | [11] |
| *Lates calcarifer* | A | Field sed | Cd, Cr, Cu, Ni, Zn | Diuron, PAHs |  |  |  | DNA unwinding + | [12] |
| *Mugil cephalus* | A | Field sed and water | Cu, Fe, Mn, Pb, Zn |  |  | + | + |  | [13] |
|  | J | Field sed and water | Cd, Cu, Mn, Ni, Pb | AHCs, PAHs, PCBs, DDTs, TBT |  |  | + |  | [14] |
| *Mullus barbatus* | A | Field sed | As, Cd, Cu, Hg, Pb, Zn | PAHs, CBs, DDT, HCB, trans-nonachlor, Lindane, Dieldrin |  | + |  |  | [15] |
| *Parablennius sanguinolentus* | A | Field water and sed | Cr, Pb | PAHs |  | +/- |  |  | [16] |
| *Plastichthys flesus* | A | Field sed | Al, Cd, Cr, Cu, Fe, Hg, Mn, Pb, Zn | PCB,DDD,DDE, HCH |  |  |  | DNA unwinding = | [17] |
|  |  | Field sed | Cd, Hg, Pb, Zn | PAHs, PCBs |  |  |  | DNA adducts + PAHs | [18] |
|  |  | Lab field sed | As, Cr, Cu, Hg, Ni, Pb, Zn | HCB, OCP, CB, naph, PAHs, TBT, DBT |  | + |  | Transcriptomics +; Metabolomics + | [19] |
|  |  | Field water and sed | As, Cd, Cr, Cu, Hg, Ni, Pb, Zn | PAHs, PCBs, OCPs |  |  |  | DNA adducts = | [20] |
|  | J | Lab field sed toxicity | As, Cr, Cu, Hg, Ni, Pb, Zn | PAHs, DDT, HCB, CB, TBT, DBT |  |  |  | Microarray +/- | [21] |
| *Poecilia vivipara acclimated to saltwater* | A | Lab water toxicity test | Cu |  |  | +/- | + |  | [22] |
| *Scophthalmus maximus* | J | Lab field sed | Cu, Pb, Zn |  |  | + |  |  | [23] |
| *Sillago schomburgkii,* | A | Field sed | Cd, Cu, Pb |  |  |  | + |  | [1] |
| *Solea senegalensis* | J | Caged field sed | Cd, Cr, Cu, Ni, Pb, Zn | PAHs, PCBs, DDT |  | + | + |  | [24] |
|  |  | Lab and field sed | As, Cu, Zn | PAHs, PCBs, DDT | +/- | +/- | + |  | [25] |
|  |  | Lab field sed | Cd, Cr, Cu, Ni, Pb, Zn | PAHs, PCBs, DDT |  | + | + |  | [26] |
|  |  |  | Cd, Cr, Cu, Ni, Pb, Zn | PAHs, PCBs, DDT |  | + | + |  | [24] |
| *Sparus aurata* | A | Lab water toxicity test | Cd |  |  | = |  |  | [27] |
|  | J | Lab field sed | As, Cd, Cr, Cu, Hg, Ni, Pb, Se, V, Zn | PAHs |  |  |  | microarray + | [28] |

Abbreviations: LHS: life history stage; J: juvenile, A: adult: Lab: laboratory; Sed: Sediment; HCB: hexachlorobenzene; OCP: total organochlorine pesticides; CB: chlorinated biphenyls; naph: naphthalenes; PAHs : total polycyclic aromatic hydrocarbons; PCBS: polychlorinated biphenyl; TBT: tributyltin; DBT: dibutytin; DDD: 1,1-dichloro-2.2-bis(p-chlorophenyl) ethane; DDE: 1,1-dichloro-2.2-bis(p-chlorophenyl) ethylene; HCH: hexachlorcyclohexane; DDT: dichlorodiphenyltrichloroethane; HCB: hexachlorobenzene; 8-OHdG: 8-hydroxy-2'-deoxyguanosine; + induction; - inhibition; = no significant induction; +/- mixed response; Fpg-comet: formamido pyrimidine glycosylase.

# References

1. Edwards JW, Edyvane KS, Boxall VA, Hamann M, Soole KL. Metal levels in seston and marine fish flesh near industrial and metropolitan centres in South Australia. Mar Pollut Bull. 2001; 42: 389-96. doi: 10.1016/s0025-326x(00)00168-5 PMID: 000169401400018
2. Piva F, Ciaprini F, Onorati F, Benedetti M, Fattorini D, Ausili A, et al. Assessing sediment hazard through a weight of evidence approach with bioindicator organisms: a practical model to elaborate data from sediment chemistry, bioavailability, biomarkers and ecotoxicological bioassays. Chemosphere. 2011; 83: 475-85. doi: 10.1016/j.chemosphere.2010.12.064 PMID: 21239037
3. Benedetti M, Ciaprini F, Piva F, Onorati F, Fattorini D, Notti A, et al. A multidisciplinary weight of evidence approach for classifying polluted sediments: Integrating sediment chemistry, bioavailability, biomarkers responses and bioassays. Environ Int. 2012; 38: 17-28. doi: 10.1016/j.envint.2011.08.003 PMID: 21982029
4. Rose WL, Nisbet RM, Green PG, Norris S, Fan T, Smith EH, et al. Using an integrated approach to link biomarker responses and physiological stress to growth impairment of cadmium-exposed larval topsmelt. Aquat Toxicol. 2006; 80: 298-308. doi: 10.1016/j.aquatox.2006.09.007 PMID: 000242776900010
5. Souza IC, Duarte ID, Pimentel NQ, Rocha LD, Morozesk M, Bonomo MM, et al. Matching metal pollution with bioavailability, bioaccumulation and biomarkers response in fish (*Centropomus parallelus*) resident in neotropical estuaries. Environ Pollut. 2013; 180: 136-44. doi: 10.1016/j.envpol.2013.05.017 PMID: 000322425300019
6. Oliveira BL, Loureiro Fernandes LF, Bianchini A, Chippari-Gomes AR, Silva BF, Brandao GP, et al. Acute copper toxicity in juvenile fat snook *Centropomus parallelus* (Teleostei: Centropomidae) in sea water. Neotrop Ichthyol. 2014; 12: 845-52. doi: 10.1590/1982-0224-20140040 PMID: 000347909800020
7. Fasulo S, Mauceri A, Maisano M, Giannetto A, Parrino V, Gennuso F, et al. Immunohistochemical and molecular biomarkers in *Coris julis* exposed to environmental contaminants. Ecotoxicol Environ Saf. 2010; 73: 873-82. doi: 10.1016/j.ecoenv.2009.12.025 PMID: 000279623800023
8. Tomasello B, Copat C, Pulvirenti V, Ferrito V, Ferrante M, Renis M, et al. Biochemical and bioaccumulation approaches for investigating marine pollution using Mediterranean rainbow wrasse, *Coris julis* (Linneaus 1798). Ecotoxicol Environ Saf. 2012; 86: 168-75. doi: 10.1016/j.ecoenv.2012.09.012 PMID: 000311064800024
9. De Domenico E, Mauceri A, Giordano D, Maisano M, Giannetto A, Parrino V, et al. Biological responses of juvenile European sea bass (*Dicentrarchus labrax*) exposed to contaminated sediments. Ecotoxicol Environ Saf. 2013; 97: 114-23. doi: 10.1016/j.ecoenv.2013.07.015 PMID: 000325039400015
10. Tse CY, Chan KM, Wong CK. DNA damage as a biomarker for assessing the effects of suspended solids on the orange-spotted grouper, *Epinephelus coioides*. Fish Physiol Biochem. 2010; 36: 141-6. doi: 10.1007/s10695-008-9243-0 PMID: 000277710800004
11. Migliarini B, Campisi AM, Maradonna F, Truzzi C, Annibaldi A, Scarponi G, et al. Effects of cadmium exposure on testis apoptosis in the marine teleost *Gobius niger*. Gen Comp Endocrinol. 2005; 142: 241-7. doi: 10.1016/j.ygcen.2004.12.012 PMID: 15862569
12. Humphrey CA, King SC, Klumpp DW. A multibiomarker approach in barramundi (*Lates calcarifer*) to measure exposure to contaminants in estuaries of tropical North Queensland. Mar Pollut Bull. 2007; 54: 1569-81. doi: 10.1016/j.marpolbul.2007.06.004 PMID: 000250599700014
13. Omar WA, Zaghloul KH, Abdel-Khalek AA, Abo-Hegab S. Genotoxic effects of metal pollution in two fish species, *Oreochromis niloticus* and *Mugil cephalus*, from highly degraded aquatic habitats. Mutat Res-Genet Tox En. 2012; 746: 7-14. doi:10.1016/j.mrgentox.2012.01.013
14. Tsangaris C, Vergolyas M, Fountoulaki E, Nizheradze K. Oxidative Stress and Genotoxicity Biomarker Responses in Grey Mullet (*Mugil cephalus*) From a Polluted Environment in Saronikos Gulf, Greece. Arch Environ Con Tox. 2011; 61: 482-90. doi: 10.1007/s00244-010-9629-8 PMID: 000298500400013
15. Martinez-Gomez C, Fernandez B, Benedicto J, Valdes J, Campillo JA, Leon VM, et al. Health status of red mullets from polluted areas of the Spanish Mediterranean coast, with special reference to Portman (SE Spain). Mar Environ Res. 2012; 77: 50-9. doi: 10.1016/j.marenvres.2012.02.002 PMID: 000304296700008
16. Tigano C, Tomasello B, Pulvirenti V, Ferrito V, Copat C, Carpinteri G, et al. Assessment of environmental stress in *Parablennius sanguinolentus* (Pallas, 1814) of the Sicilian Ionian coast. Ecotoxicol Environ Saf. 2009; 72: 1278-86. doi: 10.1016/j.ecoenv.2008.09.028 PMID: 000265767900037
17. Schmidt V, Zander S, Korting W, Broeg K, von Westernhagen H, Dizer H, et al. Parasites of flounder (*Platichthys flesus* L.) from the German Bight, North Sea, and their potential use in biological effects monitoring - C. Pollution effects on the parasite community and a comparison to biomarker responses. Helgoland Mar Res. 2003; 57: 262-71. doi: 10.1007/s10152-003-0159-x PMID: 000186604600015
18. Vethaak AD, Jol JG, Meijboom A, Eggens ML, apRheinallt T, Wester PW, et al. Skin and liver diseases induced in flounder (*Platichthys flesus*) after long-term exposure to contaminated sediments in large-scale mesocosms. Environ Health Persp. 1996; 104: 1218-29. doi: 10.2307/3432916 PMID: A1996VX74000021
19. Williams TD, Davies IM, Wu H, Diab AM, Webster L, Viant MR, et al. Molecular responses of European flounder (*Platichthys flesus*) chronically exposed to contaminated estuarine sediments. Chemosphere. 2014; 108: 152-8. doi: 10.1016/j.chemosphere.2014.01.028 PMID: 000337881600020
20. Schipper CA, Lahr J, van den Brink PJ, George SG, Hansen P-D, de Assis HCdS, et al. A retrospective analysis to explore the applicability of fish biomarkers and sediment bioassays along contaminated salinity transects. Ices J Mar Sci. 2009; 66: 2089-105. doi: 10.1093/icesjms/fsp194 PMID: 000272080600003
21. Leaver MJ, Diab A, Boukouvala E, Williams TD, Chipman JK, Moffat CF, et al. Hepatic gene expression in flounder chronically exposed to multiply polluted estuarine sediment: Absence of classical exposure 'biomarker' signals and induction of inflammatory, innate immune and apoptotic pathways. Aquat Toxicol. 2010; 96: 234-45. doi: 10.1016/j.aquatox.2009.10.025 PMID: 000274950000007
22. de Souza Machado AA, Mueller Hoff ML, Klein RD, Cardozo JG, Giacomin MM, Ledes Pinho GL, et al. Biomarkers of waterborne copper exposure in the guppy *Poecilia vivipara* acclimated to salt water. Aquat Toxicol. 2013; 138: 60-9. doi: 10.1016/j.aquatox.2013.04.009. PMID: 000322293600007
23. Hartl MGJ, Kilemade M, Sheehan D, Mothersill C, O'Halloran J, O'Brien NM, et al. Hepatic biomarkers of sediment-associated pollution in juvenile turbot, *Scophthalmus maximus* L. Mar Environ Res. 2007; 64: 191-208. doi: 10.1016/j.marenvres.2007.01.002 PMID: 000248488500007
24. Costa PM, Neuparth TS, Caeiro S, Lobo J, Martins M, Ferreira AM, et al. Assessment of the genotoxic potential of contaminated estuarine sediments in fish peripheral blood: Laboratory versus in situ studies. Environ Res. 2011; 111: 25-36. doi: 10.1016/j.envres.2010.09.011 PMID: 000286715300005
25. Costa PM, Caeiro S, Vale C, Angel DelValls T, Costa MH. Can the integration of multiple biomarkers and sediment geochemistry aid solving the complexity of sediment risk assessment? A case study with a benthic fish. Environ Pollut. 2012; 161: 107-20. doi: 10.1016/j.envpol.2011.10.010 PMID: 000300539300016
26. Costa PM, Lobo J, Caeiro S, Martins M, Ferreira AM, Caetano M, et al. Genotoxic damage in Solea senegalensis exposed to sediments from the Sado Estuary (Portugal): Effects of metallic and organic contaminants. Mutat Res-Genet Tox En. 2008; 654: 29-37. doi: 10.1016/j.mrgentox.2008.04.007 PMID: 000258049500005
27. Isani G, Andreani G, Cocchioni F, Fedeli D, Carpene E, Falcioni G. Cadmium accumulation and biochemical responses in *Sparus aurata* following sub-lethal Cd exposure. Ecotoxicol Environ Saf. 2009; 72: 224-30. doi: 10.1016/j.ecoenv.2008.04.015 PMID: 000260660100028
28. Ribecco C, Baker ME, Sasik R, Zuo Y, Hardiman G, Carnevali O. Biological effects of marine contaminated sediments on *Sparus aurata* juveniles. Aquat Toxicol. 2011; 104: 308-16. doi: 10.1016/j.aquatox.2011.05.005 PMID: 000293042100017
