# Supplementary material for: A protocol for identifying suitable biomarkers to assess fish health: A systematic review
Source: PLoS One. 2017 Apr 12;12(4):e0174762. doi: 10.1371/journal.pone.0174762 (PMC5389625; doi:10.1371/journal.pone.0174762)
Supplement: S21 Table — (DOCX) [file pone.0174762.s021.docx]

**S21 Table. Field and laboratory studies on responses of biomarkers of exposure in fish to metals and other contaminants: osmoregulatory and respiratory parameters.** Most studies measured contaminants in the environment in addition to those identified as of concern for Gladstone Harbour (Al, Cd, Cu, Ga, Pb, Se, Zn); these are also presented for completeness.

| Species | LHS | Laboratory or Field | Metals | other contaminants | lactate | LDH | Na+/K+ - ATPase | others | Reference |
| --- | --- | --- | --- | --- | --- | --- | --- | --- | --- |
| *Anguilla anguilla* | Glass eels | Field sed | Cd, Cr, Cu, Hg, Ni, Pb, V, Zn | PAH |  | - | + |  | [1] |
|  | Yellow eels | Field sed | Cd, Cr, Cu, Hg, Ni, Pb, V, Zn | PAH |  | + | + |  | [1] |
| *Centropomus parallelus* | J | Field sed and water | Ag, Al, As, Cd, Cr, Cu, Fe, Hg, Mn, Ni, Pb, Se, Zn |  |  |  | +/- |  | [2] |
| *Dicentrarchus labrax* | J | Lab field sed toxicity | As, Cd, Cr, Cu, Hg, Ni, Pb, Zn | PAHs and PCBs |  |  | = | AQPs - | [3] |
| *Fundulus heteroclitus* | A | Lab water toxicity test | Zn |  |  |  | + | Ca^2+^-ATPase + | [4] |
| *Fundulus heteroclitus* | A | Lab water toxicity test | Cu |  | = | = |  |  | [5] |
| *Solea senegalensis* | A | Field sed | Cd, Cr, Cu, Fe, Hg, Pb, Zn |  |  | = |  |  | [6] |
|  | A | Field water and sed | As, Cd, Cu, Fe, Pb, Zn | PAHs |  | = |  |  | [7] |
| *Solea solea* | A | Field sed | Cd, Cr, Cu, Fe, Hg, Pb, Zn |  |  | = |  |  | [6] |
| *Squalus acanthias* | A | Lab water toxicity test | Pb |  | = |  | - Gills  = rectal gland | TMAO =;  Urea +/-;  PaO_2_ =;  PaCO_2_ -;  arterial pH +/-;  K -;  Ca =;  NH_4_ +/-  Na -;  Cl - | [8] |
| *Synechogobius hasta* | J | Lab water toxicity test | Cd |  |  | = |  |  | [9] |

Abbreviations: LHS: life history stage; A: adult, J: juvenile, L: larvae: Lab: laboratory; Sed: Sediment; PAHs: total polycyclic aromatic hydrocarbons ; PCBS: polychlorinated biphenyl; + induction; - inhibition; = no significant induction; +/- mixed response; RB: Respiratory burst; AQPs: aquaporins; TMAO: trimethylamine oxide; LDH: lactate dehydrogenase.

# References

1. Gravato C, Guimaraes L, Santos J, Faria M, Alves A, Guilhermino L. Comparative study about the effects of pollution on glass and yellow eels (*Anguilla anguilla*) from the estuaries of Minho, Lima and Douro Rivers (NW Portugal). Ecotoxicol Environ Saf. 2010; 73: 524-33. doi: 10.1016/j.ecoenv.2009.11.009 PMID: 000277103600009
2. Souza IC, Duarte ID, Pimentel NQ, Rocha LD, Morozesk M, Bonomo MM, et al. Matching metal pollution with bioavailability, bioaccumulation and biomarkers response in fish (*Centropomus parallelus*) resident in neotropical estuaries. Environ Pollut. 2013; 180: 136-44. doi: 10.1016/j.envpol.2013.05.017 PMID: 000322425300019
3. De Domenico E, Mauceri A, Giordano D, Maisano M, Gioffre G, Natalotto A, et al. Effects of "in vivo" exposure to toxic sediments on juveniles of sea bass (*Dicentrarchus labrax*). Aquat Toxicol. 2011; 105: 688-97. doi: 10.1016/j.aquatox.2011.08.026 PMID: 000298120600055
4. Loro VL, Nogueira L, Nadella SR, Wood CM. Zinc bioaccumulation and ionoregulatory impacts in *Fundulus heteroclitus* exposed to sublethal waterborne zinc at different salinities. Comp Biochem Phys C. 2014; 166: 96-104. doi: 10.1016/j.cbpc.2014.07.004 PMID: 000342532000011
5. Ransberry VE, Morash AJ, Blewett TA, Wood CM, McClelland GB. Oxidative stress and metabolic responses to copper in freshwater- and seawater-acclimated killifish, *Fundulus heteroclitus*. Aquat Toxicol. 2015; 161: 242-52. doi: 10.1016/j.aquatox.2015.02.013 PMID: 000352177500026
6. Siscar R, Torreblanca A, Palanques A, Sole M. Metal concentrations and detoxification mechanisms in *Solea solea* and *Solea senegalensis* from NW Mediterranean fishing grounds. Mar Pollut Bull. 2013; 77: 90-9. doi: 10.1016/j.marpolbul.2013.10.026 PMID: 000329888600025
7. Oliva M, Antonio Perales J, Gravato C, Guilhermino L, Dolores Galindo-Riano M. Biomarkers responses in muscle of Senegal sole (*Solea senegalensis*) from a heavy metals and PAHs polluted estuary. Mar Pollut Bull. 2012; 64: 2097-108. doi: 10.1016/j.marpolbul.2012.07.017 PMID: 000310929500028
8. Eyckmans M, Lardon I, Wood CM, De Boeck G. Physiological effects of waterborne lead exposure in spiny dogfish (*Squalus acanthias*). Aquat Toxicol. 2013; 126: 373-81. doi: 10.1016/j.aquatox.2012.09.004 PMID: 000315125600040
9. Liu XJ, Luo Z, Li CH, Xiong BX, Zhao YH, Li XD. Antioxidant responses, hepatic intermediary metabolism, histology and ultrastructure in *Synechogobius hasta* exposed to waterborne cadmium. Ecotoxicol Environ Saf. 2011; 74: 1156-63. doi: 10.1016/j.ecoenv.2011.02.015 PMID: 000291960600007
