# Supplementary material for: A protocol for identifying suitable biomarkers to assess fish health: A systematic review
Source: PLoS One. 2017 Apr 12;12(4):e0174762. doi: 10.1371/journal.pone.0174762 (PMC5389625; doi:10.1371/journal.pone.0174762)
Supplement: S22 Table — (DOCX) [file pone.0174762.s022.docx]

**S22 Table. Field and laboratory studies on responses of biomarkers of effect in fish to metals and other contaminants: carbohydrate and aerobic metabolism.** Most studies measured contaminants in the environment in addition to those identified as of concern for Gladstone Harbour (Al, Cd, Cu, Ga, Pb, Se, Zn); these are also presented for completeness.

| Species | LHS | Tissue | Method | Laboratory or Field | Metals | Other contaminants | PK | HK | CS | COX | CS:COS ratio | Others | Reference |
| --- | --- | --- | --- | --- | --- | --- | --- | --- | --- | --- | --- | --- | --- |
| *Fundulus heteroclitus* | A | gill | Bioassay | Lab water toxicity test | Cu |  | - | = | = | - | = |  | [1] |
|  |  | intestine | Bioassay | Lab water toxicity test | Cu |  | + | = | + | + | = |  | [1] |
|  |  | liver | Bioassay | Lab water toxicity test | Cu |  | = | = | = | = | = |  | [1] |
| *Solea senegalensis* | A | muscle | Bioassay | Field water and sed | As, Cd, Cu, Fe, Pb, Zn | PAHs, |  |  |  |  |  | IDH +\- | [2] |
| *Sparus aurata* | J | liver | Real time PCR | Lab field sed toxicity | As, Cd, Cr, Cu, Hg, Ni, Pb, Se, V, Zn | PAHs |  |  |  |  |  | Erα +; RXRα + | [3] |
| *Synechogobius hasta* | J | liver | Bioassay | Lab water toxicity test | Cd |  | + |  |  |  |  | SDH -; MDH +\-; HL +;  LPL +; | [4] |

Abbreviations: Lab: laboratory; Sed: Sediment; PAHs : total polycyclic aromatic hydrocarbons; PK: pyruvate kinase; HK: hexokinase; CS: citrate synthase; COX: cytochrome c oxidase; + induction; - inhibition; = no significant induction; +/- mixed response; IDH:Isocitrate dehydrogenase; Erα: nuclear receptor binds xenochemical compounds termed 'estrogen like molecules; RXRα: 9-cis retinic acid receptor; SDH: succinate dehydrogenase; MDH: malic dehydrogenase; HL: hepatic lipase; LPL: lipoprotein lipase.

# References

1. Ransberry VE, Morash AJ, Blewett TA, Wood CM, McClelland GB. Oxidative stress and metabolic responses to copper in freshwater- and seawater-acclimated killifish, *Fundulus heteroclitus*. Aquat Toxicol. 2015; 161: 242-52. doi: 10.1016/j.aquatox.2015.02.013 PMID: 000352177500026
2. Oliva M, Antonio Perales J, Gravato C, Guilhermino L, Dolores Galindo-Riano M. Biomarkers responses in muscle of Senegal sole (*Solea senegalensis*) from a heavy metals and PAHs polluted estuary. Mar Pollut Bull. 2012; 64: 2097-108. doi: 10.1016/j.marpolbul.2012.07.017 PMID: 000310929500028
3. Ribecco C, Baker ME, Sasik R, Zuo Y, Hardiman G, Carnevali O. Biological effects of marine contaminated sediments on *Sparus aurata* juveniles. Aquat Toxicol. 2011; 104: 308-16. doi: 10.1016/j.aquatox.2011.05.005 PMID: 000293042100017
4. Liu XJ, Luo Z, Li CH, Xiong BX, Zhao YH, Li XD. Antioxidant responses, hepatic intermediary metabolism, histology and ultrastructure in *Synechogobius hasta* exposed to waterborne cadmium. Ecotoxicol Environ Saf. 2011; 74: 1156-63. doi: 10.1016/j.ecoenv.2011.02.015 PMID: 000291960600007
