# Supplementary material for: A protocol for identifying suitable biomarkers to assess fish health: A systematic review
Source: PLoS One. 2017 Apr 12;12(4):e0174762. doi: 10.1371/journal.pone.0174762 (PMC5389625; doi:10.1371/journal.pone.0174762)
Supplement: S23 Table — (DOCX) [file pone.0174762.s023.docx]

**S23 Table. Field and laboratory studies on responses of biomarkers of effect in fish to metals and other contaminants: histopathology and gross indices.** Most studies measured contaminants in the environment in addition to those identified as of concern for Gladstone Harbour (Al, Cd, Cu, Ga, Pb, Se, Zn); these are also presented for completeness.

| Species | LHS | Laboratory or Field | Metals | Other contaminants | HA | HIS/ LSI | GSI | CF | K index | RNA:DNA ratio | TLC | Other | Reference |
| --- | --- | --- | --- | --- | --- | --- | --- | --- | --- | --- | --- | --- | --- |
| *Acanthopagrus latus* | A | Field sed | As, Cr, Cu, Ni, Pb, V, Zn | PAHs |  | - |  | = |  |  |  |  | (1) |
| *Anguilla anguilla* | Glass eels | Field sed | Cd, Cr, Cu, Hg, Ni, Pb, V, Zn | PAH |  |  |  |  | = |  |  |  | (2) |
| *Anguilla anguilla* | Yellow eels | Field sed | Cd, Cr, Cu, Hg, Ni, Pb, V, Zn | PAH |  |  |  |  | - |  |  |  | (2) |
| *Aphanius fasciatus* | A | Field water and sed | Cd, Cu, Zn | PAHs |  |  |  | +/- |  |  |  |  | (3) |
| *Aphanius fasciatus* | J | Field sed | Cd, Cd, Cu, Cu, ZnZn | PAHs | + (Collagen) |  |  |  | = |  |  | COL1A2 +/- | (4) |
| *Aphanius fasciatus* | J | Lab water toxicity test | Cd |  |  |  |  |  |  |  |  | COl1A2 - | (4) |
| *Atherina presbyter* | A | Field sed | Cd, Hg, Ni, Pb, Zn | PAHs |  |  |  |  | = | +/- |  |  | (5) |
| *Atherinops affnis* | L | Lab water toxicity test | Cd |  |  |  |  |  |  |  |  | Food intake - | (6) |
| *Centropomus parallelus* | J | Field sed and water | Ag, Al, As, Cd, Cr, Cu, Fe, Hg, Mn, Ni, Pb, Se, Zn |  | = |  |  |  |  |  |  |  | (7) |
| *Chanos chanos* | A | Field sed and water | Cd, Cu, Fe, Mn, Pb, Zn |  | + |  |  |  |  |  |  |  | (8) |
| *Coris julis* | A | Field sed | Cd, Co, Cr, Cu, Ni, Pb, Sb, Zn |  | + |  |  |  |  |  |  |  | (9) |
| *Cynoglossus arel* | A | Field sed | As, Cr, Cu, Ni, Pb, V, Zn | PAHs |  | = |  | = |  |  |  |  | (1) |
| *Dicentrarchus labrax* | A | Caged field sed | Cu, Pb, Zn | PAHs |  | - |  |  |  |  |  |  | (10) |
| *Dicentrarchus labrax* | A | Field sed | Cr, Cu, Ni, Pb, Zn | PAHs |  |  |  |  |  | - | - | TPC -; | (11) |
| *Dicentrarchus labrax* | J | Caged field sed | Al, Cd, Cr, Cu, Mn, Ni, Pb, V, Zn |  |  |  |  |  | - | - |  | Growth index -; TAG:ST - | (12) |
| *Dicentrarchus labrax* | J | Lab field sed toxicity | As, Cd, Co, Cr, Cu, Fe, Hg, Mn, Ni, Pb, Sb, V, Zn |  | + |  |  |  |  |  |  |  | (13) |
| *Dicentrarchus labrax* | J | Lab water toxicity test | Cd |  |  |  |  |  |  |  |  | Lateral line tissue + | (14) |
| *Epinephelus coioides* | J | Lab water toxicity test | Cu |  | + |  |  |  |  |  | - | Digestive enzymes -; TSFA +; MFA +; TPFA -; | (15) |
| *Fundulus heteroclitus* | A | Field and lab sed | Cd, Cu, Hg, Pb, Zn | PAHs, PCBs |  |  |  |  |  |  |  | Prey capture -; | (16) |
| *Fundulus heteroclitus* | L | Field sed / lab | Cd, Cu, Hg, Pb, Zn | PAHs, PCBs |  |  |  |  |  |  |  | Prey capture -; | (17) |
| *Gadus morhua L.* | A | Cage field water | Cd, Cu, Hg, Pb, Zn | PAHs, PCBs |  | = | = | = |  |  |  | SSI = | (18) |
| *Gadus morhua L.* | J | Lab water toxicity test | As, Cd, Co, Cr, CuHg, Mn, Ni, Pb, Zn | PAHs | = |  |  |  |  |  |  |  | (19) |
| *Galaxias maculatus* | E | Lab water and sed toxicity test | Cu, Pb, Zn |  |  |  |  |  |  |  |  | Phototactic response - | (20) |
| *Lates calcarifer* | A | Field sed | Cd, Cr, Cu, Ni, Zn | Diuron, PAHs |  | = |  |  | = | - |  |  | (21) |
| *Lates calcarifer* |  | Lab water toxicity test | Cu |  | + |  |  |  |  |  |  |  | (22) |
| *Limanda limanda* | A | Field sed | Cd, Cr, Cu, Hg, Ni, Pb, Zn | PAHs, PCBs | = |  |  | + |  |  |  | Parasites =; | (23) |
| *Lutjanus russellii* | A | Field sed and water | Cd, Cu, Fe, Pb, Zn |  |  | - |  |  |  |  |  |  | (24) |
| *Mugil cephalus* | A | Field sed and water | Cu, Fe, Mn, Pb, Zn |  | + |  |  | - |  |  |  |  | (25) |
| *Mugil cephalus* | F | Lab water toxicity test | Pb |  | + |  |  |  |  |  |  |  | (26) |
| *Mugil cephaus* | J | Field sed and water | Cd, Cu, Mn, Ni, Pb | AHCs, PAHs, PCBs, DDTs, TBT |  |  |  | = |  |  |  |  | (27) |
| *Mullus barbatus* | A | Field sed | As, Cd, Cu, Hg, Pb, Zn | PAHs, CBs, DDT, HCB, trans-nonachlor, Lindane, Dieldrin |  | = | - | - |  |  | - |  | (28) |
| *Plastichthys flesus* | A | Cage field water | Cd, Cu, Hg, Pb, Zn | PAHs, PCBs |  | = |  | = |  |  |  |  | (18) |
| *Plastichthys flesus* | A | Field sed | Al, Cd, Cr, Cu, Fe, Hg, Mn, Pb, Zn | PCB,DDD,DDE, HCH |  |  |  |  |  |  |  | Parasites - | (29) |
| *Plastichthys flesus* | J | Field sed | Al, Cd, Cr, Cu, Hg, Mn, Ni, Pb, V, Zn | PAHs |  |  |  |  | - |  | - | TAG:ST - | (30) |
| *Plastichthys flesus* | J | Field sed | Al, Cd, Cr, Cu, Hg, Mn, Ni, Pb, V, Zn |  |  |  |  |  | - | - |  |  | (31) |
| *Plastichthys flesus* | J | Lab field sed toxicity | As, Cr, Cu, Hg, Ni, Pb, Zn | PAHs, DDT, HCB, CB, TBT, DBT |  |  | = |  |  |  |  |  | (32) |
| *Platichthys flesus* | A | Field sed | Cd, Hg, Pb, Zn | PAHs, PCBs | + lesions | = |  | +/- |  |  |  | Epidermal disease = | (33) |
| *Platichthys flesus L.* | A | Field water and sed | As, Cd, Cr, Cu, Hg, Ni, Pb, Zn | PAHs, PCBs, OCPs | = | = | = | - |  | +/- |  | TPC +\-; | (34) |
| *Pogpmichthys macrolepidotus* | L | Lab food | MeHgSe |  | + |  |  | = |  |  |  |  | (35) |
| *Pomadasys hasta* | A | Field sed and water | Cd, Cu, Fe, Pb, Zn |  |  | - |  |  |  |  |  |  | (24) |
| *Pomatoschistus microps* | A | Field sed | Cd, Cr, Cu, Hg, Ni, Pb, Zn | PAHs |  |  |  |  | = | = |  |  | (36) |
| *Pomatoschistus microps* | A | Field sed | Cd, Hg, Ni, Pb, Zn | PAHs |  |  |  |  | = | +/- |  |  | (5) |
| *Pomatoschistus microps* | A | Field sed | Cr, Cu, Ni, Pb, Zn | PAHs |  |  |  |  |  | - | = | TPC +/- | (11) |
| *Scophtalmus maximus* | J | Caged field sed | Al, Cd, Cr, Cu, Mn, Ni, Pb, V, Zn |  |  |  |  |  | - | = |  | Growth index =; TAG:ST - | (12) |
| *Scophtalmus maximus* | J | Lab field sed | Al, Cd, Cr, Cu, Mn, Ni, Pb, V, Zn |  |  |  |  |  | - | +/- |  | Growth index +\-; TAG:ST - | (37) |
| *Seriola lalandi* | J | Lab food | Se |  | = | - |  |  |  |  |  |  | (38) |
| *Solea senegalensis* | A | Field sed | Cr, Cu, Ni, Pb, Zn | PAHs |  |  |  |  |  | +/- | +/- | TPC +\- | (11) |
| *Solea senegalensis* | J | Field sed | Cd, Cr, Cu, Ni, Pb, Zn | PAHs |  |  |  |  | + | +/- |  |  | (39) |
| *Solea senegalensis* | J | Lab and field sed | As, Cu, Zn | PAHs, PCBs, DDT | + |  |  |  |  |  |  |  | (40) |
| *Solea senegalensis* | J | Lab field sed toxicity | As, Cd, Cr, Cu, Hg, Ni, Pb, Zn | PAHs | + |  |  | + |  |  |  |  | (41) |
| *Solea senegalensis* | J | Lab field sed toxicity | Cd, Cr, Cu, Ni, Pb, Zn | PAHs, PCBs, DDT | + |  |  |  |  |  |  |  | (42) |
| *Sparus aurata* | J | Lab field sed toxicity | As, Cd, Cr, Cu, Hg, Ni, Pb, Zn | PAHs | + |  |  | + |  |  |  |  | (41) |
| *Squalus acanthias* | A | Lab water toxicity test | Pb |  |  |  |  |  |  |  |  | TPC liver +/-; glycogen muscle - | (43) |
| *Symphodus melops* | A | Field water and sed | Fe, Pb, Zn |  |  | = | - | = |  |  |  | SSI - | (44) |
| *Synechogobius hasta* | J | Lab water toxicity test | Cd |  | + | + |  | = |  |  | + | VSI = | (45) |
| *Terapon jarbua* | F | Lab water toxicity test | Pb |  | + |  |  |  |  |  |  |  | (26) |
| *Terapon jarbua* | J | Lab food | Cd |  |  |  |  | = |  |  |  |  | (46) |

Abbreviations: LHS: life history stage; A: adult, E: eggs, F: fingerlings, J: juvenile, L: larvae: Lab: laboratory; Sed: Sediment; HCB: hexachlorobenzene; OCP: total organochlorine pesticides; CB: chlorinated biphenyls; naph: naphthalenes; PAHs : total polycyclic aromatic hydrocarbons; PCBS: polychlorinated biphenyl; TBT: tributyltin; DBT: dibutytin; DDD: 1,1-dichloro-2.2-bis(p-chlorophenyl) ethane; DDE: 1,1-dichloro-2.2-bis(p-chlorophenyl) ethylene; HCH: hexachlorcyclohexane; DDT: dichlorodiphenyltrichloroethane; HCB: hexachlorobenzene; HA: histological alteration; HSI: hepatosomatic index; LSI: liver somatic index; GSI: gonadosomatic index; CF: condition factor; + induction; - inhibition; = no significant induction; +/- mixed response; TAG:ST - lipid storage index; TLC - total lipid content; TPC - total protein content; TPFA - total polyunsaturated fatty acids; MFA - monounsaturated fatty acids; TSFA - total saturated fatty acids; COL1A2: Collagen 1A2; VSI: viscerosomatic index; Vac: Vacuolation; Fibr: Fibrillar structures.

# References

1. Beg MU, Al-Jandal N, Al-Subiai S, Karam Q, Husain S, Butt SA, et al. Metallothionein, oxidative stress and trace metals in gills and liver of demersal and pelagic fish species from Kuwaits’ marine area. Mar Pollut Bull. 2015; 100: 662-72. doi: 10.1016/j.marpolbul.2015.07.058
2. Gravato C, Guimaraes L, Santos J, Faria M, Alves A, Guilhermino L. Comparative study about the effects of pollution on glass and yellow eels (*Anguilla anguilla*) from the estuaries of Minho, Lima and Douro Rivers (NW Portugal). Ecotoxicol Environ Saf. 2010; 73: 524-33. doi: 10.1016/j.ecoenv.2009.11.009 PMID: 000277103600009
3. Annabi A, Kessabi K, Navarro A, Said K, Messaoudi I, Pina B. Assessment of reproductive stress in natural populations of the fish *Aphanius fasciatus* using quantitative mRNA markers. Aquat Biol. 2012; 17: 285-+. doi: 10.3354/ab00482 PMID: 000312247800008
4. Kessabi K, Annabi A, Navarro A, Casado M, Hwas Z, Said K, et al. Structural and molecular analysis of pollution-linked deformities in a natural *Aphanius fasciatus* (Valenciennes, 1821) population from the Tunisian coast. J Environ Monitor. 2012; 14: 2254-60. doi: 10.1039/c2em30329a PMID: 000306852100028
5. Fonseca VF, Vasconcelos RP, Franca S, Serafim A, Lopes B, Company R, et al. Modeling fish biological responses to contaminants and natural variability in estuaries. Mar Environ Res. 2014; 96: 45-55. doi: 10.1016/j.marenvres.2013.10.011 PMID: 000334981600007
6. Rose WL, Nisbet RM, Green PG, Norris S, Fan T, Smith EH, et al. Using an integrated approach to link biomarker responses and physiological stress to growth impairment of cadmium-exposed larval topsmelt. Aquat Toxicol. 2006; 80: 298-308. doi: 10.1016/j.aquatox.2006.09.007 PMID: 000242776900010
7. Souza IC, Duarte ID, Pimentel NQ, Rocha LD, Morozesk M, Bonomo MM, et al. Matching metal pollution with bioavailability, bioaccumulation and biomarkers response in fish (*Centropomus parallelus*) resident in neotropical estuaries. Environ Pollut. 2013; 180: 136-44. doi: 10.1016/j.envpol.2013.05.017 PMID: 000322425300019
8. Rajeshkumar S, Munuswamy N. Impact of metals on histopathology and expression of HSP 70 in different tissues of Milk fish (*Chanos chanos*) of Kaattuppalli Island, South East Coast, India. Chemosphere. 2011; 83: 415-21. doi: 10.1016/j.chemosphere.2010.12.086 PMID: 21257190
9. Fasulo S, Mauceri A, Maisano M, Giannetto A, Parrino V, Gennuso F, et al. Immunohistochemical and molecular biomarkers in *Coris julis* exposed to environmental contaminants. Ecotoxicol Environ Saf. 2010; 73: 873-82. doi: 10.1016/j.ecoenv.2009.12.025 PMID: 000279623800023
10. Traven L, Micovic V, Lusic DV, Smital T. The responses of the hepatosomatic index (HSI), 7-ethoxyresorufin-O-deethylase (EROD) activity and glutathione-S-transferase (GST) activity in sea bass (*Dicentrarchus labrax*, Linnaeus 1758) caged at a polluted site: implications for their use in environmental risk assessment. Environ Monitor Ass. 2013; 185: 9009-18. doi: 10.1007/s10661-013-3230-3 PMID: 000325116500018
11. Fonseca VF, Franca S, Serafim A, Company R, Lopes B, Bebianno MJ, et al. Multi-biomarker responses to estuarine habitat contamination in three fish species: *Dicentrarchus labrax*, *Solea senegalensis* and *Pomatoschistus microps*. Aquat Toxicol. 2011; 102: 216-27. doi: 10.1016/j.aquatox.2011.01.018 PMID: 21356184
12. Kerambrun E, Henry F, Courcot L, Gevaert F, Amara R. Biological responses of caged juvenile sea bass (*Dicentrarchus labrax*) and turbot (*Scophtalmus maximus*) in a polluted harbour. Ecol Indic. 2012; 19: 161-71. doi: 10.1016/j.ecolind.2011.06.035
13. De Domenico E, Mauceri A, Giordano D, Maisano M, Gioffre G, Natalotto A, et al. Effects of "in vivo" exposure to toxic sediments on juveniles of sea bass (*Dicentrarchus labrax*). Aquat Toxicol. 2011; 105: 688-97. doi: 10.1016/j.aquatox.2011.08.026 PMID: 000298120600055
14. Faucher K, Fichet D, Miramand P, Lagardere J-P. Impact of chronic cadmium exposure at environmental dose on escape behaviour in sea bass (*Dicentrarchus labrax* L.; Teleostei, Moronidae). Environ Pollut. 2008; 151: 148-57. doi: 10.1016/j.envpol.2007.02.017 PMID: 000252766500017
15. Wang T, Long X, Cheng Y, Liu Z, Yan S. A Comparison Effect of Copper Nanoparticles versus Copper Sulphate on Juvenile *Epinephelus coioides*: Growth Parameters, Digestive Enzymes, Body Composition, and Histology as Biomarkers. Internat J Genom. 2015; 2015: 783021-. doi: 10.1155/2015/783021 PMID: 26527479
16. Weis JS, Samson J, Zhou T, Skurnick J, Weis P. Prey capture ability of mummichogs (*Fundulus heteroclitus*) as a behavioral biomarker for contaminants in estuarine systems. Can J Fish Aquat Sci. 2001; 58: 1442-52. doi: 10.1139/cjfas-58-7-1442 PMID: 000169776600017
17. Weis JS, Samson J, Zhou T, Skurnick J, Weis P. Evaluating prey capture by larval mummichogs (*Fundulus heteroclitus*) as a potential biomarker for contaminants. Mar Environ Res. 2003; 55: 27-38. doi: 10.1016/s0141-1136(02)00204-0 PMID: 000179347600002
18. Beyer J, Sandvik M, Hylland K, Fjeld E, Egaas E, Aas E, et al. Contaminant accumulation and biomarker responses in flounder (*Platichthys flesus* L) and Atlantic cod (*Gadus morhua* L) exposed by caging to polluted sediments in Sorfjorden, Norway. Aquat Toxicol. 1996; 36: 75-98. doi: 10.1016/s0166-445x(96)00798-9 PMID: A1996VY98200005
19. Beyer J, Aarab N, Tandberg AH, Ingvarsdottir A, Bamber S, Borseth JF, et al. Environmental harm assessment of a wastewater discharge from Hammerfest LNG: A study with biomarkers in mussels (*Mytilus* sp.) and Atlantic cod (*Gadus morhua*). Mar Pollut Bull. 2013; 69: 28-37. doi: 10.1016/j.marpolbul.2013.01.001 PMID: 000318377400017
20. Barbee NC, Ganio K, Swearer SE. Integrating multiple bioassays to detect and assess impacts of sublethal exposure to metal mixtures in an estuarine fish. Aquat Toxicol. 2014; 152: 244-55. doi: 10.1016/j.aquatox.2014.04.012 PMID: 000338607300027
21. Humphrey CA, King SC, Klumpp DW. A multibiomarker approach in barramundi (*Lates calcarifer*) to measure exposure to contaminants in estuaries of tropical North Queensland. Mar Pollut Bull. 2007; 54: 1569-81. doi: 10.1016/j.marpolbul.2007.06.004 PMID: 000250599700014
22. Maharajan A, Kitto MR, Paruruckumani PS, Ganapiriya V. Histopathology biomarker responses in Asian sea bass, *Lates calcarifer* (Bloch) exposed to copper. J Basic App Zool. 2016; 77: 21-30. doi: 10.1016/j.jobaz.2016.02.001
23. Stronkhorst J, Ariese F, van Hattum B, Postma JF, de Kluijver M, Den Besten PJ, et al. Environmental impact and recovery at two dumping sites for dredged material in the North Sea. Environ Pollut. 2003; 124: 17-31. doi: 10.1016/s0269-7491(02)00430-x PMID: 000182868900003
24. Omar WA, Saleh YS, Marie M-AS. The use of biotic and abiotic components of Red Sea coastal areas as indicators of ecosystem health. Ecotoxicol. 2016; 25: 253-66. doi: 10.1007/s10646-015-1584-8 PMID: 000370716000001
25. Omar WA, Zaghloul KH, Abdel-Khalek AA, Abo-Hegab S. Genotoxic effects of metal pollution in two fish species, *Oreochromis niloticus* and *Mugil cephalus*, from highly degraded aquatic habitats. Mutat Res-Genet Tox En. 2012; 746: 7-14. doi:10.1016/j.mrgentox.2012.01.013
26. Hariharan G, Purvaja R, Ramesh R. Environmental safety level of lead (Pb) pertaining to toxic effects on grey mullet (*Mugil cephalus*) and Tiger perch (*Terapon jarbua*). Environ Toxicol. 2016; 31: 24-43. doi: 10.1002/tox.22019 PMID: 000366585300003
27. Tsangaris C, Vergolyas M, Fountoulaki E, Nizheradze K. Oxidative Stress and Genotoxicity Biomarker Responses in Grey Mullet (*Mugil cephalus*) From a Polluted Environment in Saronikos Gulf, Greece. Arch Environ Con Tox. 2011; 61: 482-90. doi: 10.1007/s00244-010-9629-8 PMID: 000298500400013
28. Martinez-Gomez C, Fernandez B, Benedicto J, Valdes J, Campillo JA, Leon VM, et al. Health status of red mullets from polluted areas of the Spanish Mediterranean coast, with special reference to Portman (SE Spain). Mar Environ Res. 2012; 77: 50-9. doi: 10.1016/j.marenvres.2012.02.002 PMID: 000304296700008
29. Schmidt V, Zander S, Korting W, Broeg K, von Westernhagen H, Dizer H, et al. Parasites of flounder (*Platichthys flesus* L.) from the German Bight, North Sea, and their potential use in biological effects monitoring - C. Pollution effects on the parasite community and a comparison to biomarker responses. Helgoland Mar Res. 2003; 57: 262-71. doi: 10.1007/s10152-003-0159-x PMID: 000186604600015
30. Kerambrun E, Henry F, Cornille V, Courcot L, Amara R. A combined measurement of metal bioaccumulation and condition indices in juvenile European flounder, *Platichthys flesus*, from European estuaries. Chemosphere. 2013; 91: 498-505. doi: 10.1016/j.chemosphere.2012.12.010 PMID: 000317325700010
31. Henry F, Filipuci I, Billon G, Courcot L, Kerambrun E, Amara R. Metal concentrations, growth and condition indices in European juvenile flounder (*Platichthys flesus*) relative to sediment contamination levels in four Eastern English Channel estuaries. J Environ Monitor. 2012; 14: 3211-9. doi: 10.1039/c2em30765k PMID: 000312655800017
32. Leaver MJ, Diab A, Boukouvala E, Williams TD, Chipman JK, Moffat CF, et al. Hepatic gene expression in flounder chronically exposed to multiply polluted estuarine sediment: Absence of classical exposure 'biomarker' signals and induction of inflammatory, innate immune and apoptotic pathways. Aquat Toxicol. 2010; 96: 234-45. doi: 10.1016/j.aquatox.2009.10.025 PMID: 000274950000007
33. Vethaak AD, Jol JG, Meijboom A, Eggens ML, apRheinallt T, Wester PW, et al. Skin and liver diseases induced in flounder (*Platichthys flesus*) after long-term exposure to contaminated sediments in large-scale mesocosms. Environ Health Persp. 1996; 104: 1218-29. doi: 10.2307/3432916 PMID: A1996VX74000021
34. Schipper CA, Lahr J, van den Brink PJ, George SG, Hansen P-D, de Assis HCdS, et al. A retrospective analysis to explore the applicability of fish biomarkers and sediment bioassays along contaminated salinity transects. Ices J Mar Sci. 2009; 66: 2089-105. doi: 10.1093/icesjms/fsp194 PMID: 000272080600003
35. Deng DF, Teh FC, Teh SJ. Effect of dietary methylmercury and seleno-methionine on Sacramento splittail larvae. Sci Total Environ. 2008; 407: 197-203. doi: 10.1016/j.scitotenv.2008.08.028 PMID: 000261877900019
36. Serafim A, Company R, Lopes B, Fonseca VF, Franca S, Vasconcelos RP, et al. Application of an integrated biomarker response index (IBR) to assess temporal variation of environmental quality in two Portuguese aquatic systems. Ecol Indic. 2012; 19: 215-25. doi: 10.1016/j.ecolind.2011.08.009 PMID: 000302891100022
37. Kerambrun E, Henry F, Perrichon P, Courcot L, Meziane T, Spilmont N, et al. Growth and condition indices of juvenile turbot, *Scophthalmus maximus*, exposed to contaminated sediments: Effects of metallic and organic compounds. Aquat Toxicol. 2012; 108: 130-40. doi:10.1016/j.aquatox.2011.07.016
38. Ky Trung L, Fotedar R. Toxic effects of excessive levels of dietary selenium in juvenile yellowtail kingfish (*Seriola lalandi*). Aquacult. 2014; 433: 229-34. doi: 10.1016/j.aquaculture.2014.06.021 PMID: 000342529400033
39. Fonseca VF, Vasconcelos RP, Tanner SE, Franca S, Serafim A, Lopes B, et al. Habitat quality of estuarine nursery grounds: Integrating non-biological indicators and multilevel biological responses in *Solea senegalensis*. Ecol Indic. 2015; 58: 335-45. doi: 10.1016/j.ecolind.2015.05.064 PMID: 000360776100035
40. Costa PM, Caeiro S, Vale C, Angel DelValls T, Costa MH. Can the integration of multiple biomarkers and sediment geochemistry aid solving the complexity of sediment risk assessment? A case study with a benthic fish. Environ Pollut. 2012; 161: 107-20. doi: 10.1016/j.envpol.2011.10.010 PMID: 000300539300016
41. Jimenez-Tenorio N, Morales-Caselles C, Kalman J, Salamanca MJ, Luisa Gonzalez de Canales M, Sarasquete C, et al. Determining sediment quality for regulatory proposes using fish chronic bioassays. Environ Internat. 2007; 33: 474-80. doi: 10.1016/j.envint.2006.11.009 PMID: 000246315800008
42. Costa PM, Diniz MS, Caeiro S, Lobo J, Martins M, Ferreira AM, et al. Histological biomarkers in liver and gills of juvenile *Solea senegalensis* exposed to contaminated estuarine sediments: A weighted indices approach. Aquat Toxicol. 2009; 92: 202-12. doi: 10.1016/j.aquatox.2008.12.009 PMID: 000266184200012
43. Eyckmans M, Lardon I, Wood CM, De Boeck G. Physiological effects of waterborne lead exposure in spiny dogfish (*Squalus acanthias*). Aquat Toxicol. 2013; 126: 373-81. doi: 10.1016/j.aquatox.2012.09.004 PMID: 000315125600040
44. Almroth BC, Sturve J, Stephensen E, Holth TF, Forlin L. Protein carbonyls and antioxidant defenses in corkwing wrasse (*Symphodus melops*) from a heavy metal polluted and a PAH polluted site. Mar Environ Res. 2008; 66: 271-7. doi: 10.1016/j.marenvres.2008.04.002 PMID: 000257817100006
45. Liu XJ, Luo Z, Li CH, Xiong BX, Zhao YH, Li XD. Antioxidant responses, hepatic intermediary metabolism, histology and ultrastructure in *Synechogobius hasta* exposed to waterborne cadmium. Ecotoxicol Environ Saf. 2011; 74: 1156-63. doi: 10.1016/j.ecoenv.2011.02.015 PMID: 000291960600007
46. Dang F, Wang W-X. Assessment of tissue-specific accumulation and effects of cadmium in a marine fish fed contaminated commercially produced diet. Aquat Toxicol. 2009; 95: 248-55. doi: 10.1016/j.aquatox.2009.09.013 PMID: 000272784900009
